# Structural and Biophysical Analyses of Human MEK2 in Complex with Two Inhibitors Reveal the Determinants of Isoform-Dependent Inhibitor Binding

**DOI:** 10.3390/ijms27135992

**Published:** 2026-07-03

**Authors:** Sang Won Cheon, Eunmi Hwang, Gi Baek Lee, Yoonyoung Heo, Hyoun Sook Kim, Byung Woo Han

**Affiliations:** 1 Research Institute of Pharmaceutical Sciences & Natural Products Research Institute, College of Pharmacy, Seoul National University, Seoul 08826, Republic of Korea; cjstkddnjs33@snu.ac.kr (S.W.C.); bhwangb@gmail.com (E.H.); spacekevin@snu.ac.kr (G.B.L.); 2 Research Institute, National Cancer Center, Goyang 10408, Republic of Korea; hey1066@ncc.re.kr (Y.H.); hskim@ncc.re.kr (H.S.K.)

**Keywords:** MEK1/2, non-competitive inhibitor, allosteric inhibitor, molecular dynamics, oligomeric state difference

## Abstract

Selective inhibition of MEK isoforms remains a central challenge in MAPK-targeted drug discovery, largely due to the structural similarity between MEK1 and MEK2. While MEK1 has been extensively characterized, the structural basis of MEK2-specific ligand recognition is not fully understood. Here, we present crystal structures of human MEK2 in complex with the noncompetitive inhibitor U0126 and the allosteric inhibitor refametinib at resolutions of 3.15 Å and 3.30 Å, respectively. Despite a conserved kinase fold, MEK2 exhibits isoform-specific features within the N-lobe β-sheet. Additional differences are observed in the relative orientation of the helix C and activation segment, and the helix F-supported regulatory spine. Structural differences are reflected in micromolar binding affinities for U0126 (*K*_d_ = 9.8 μM) and refametinib (*K*_d_ = 7.4 μM). Notably, a single N-lobe substitution (Thr87 in MEK2 versus Phe83 in MEK1) selectively enhanced U0126 binding. The MEK2 T87F mutant exhibited an approximately twofold increase in affinity, while refametinib binding remained largely unchanged. SEC–MALS analysis demonstrated that MEK2 predominantly exists as a monomer in solution, contrasting with the reported homodimeric behavior of MEK1. Molecular dynamics simulations supported these findings by revealing isoform-specific differences in oligomeric state-dependent flexibility and inhibitor-induced dynamics. Collectively, our findings define the structural basis underlying the differential inhibitor recognition of MEK2 and MEK1, providing mechanistic insight into isoform-selective MEK-targeted drug design.

## 1. Introduction

Mitogen-activated protein kinase kinases (MEKs), particularly human MEK1 and MEK2, are essential components of the highly conserved Ras-Raf-MEK-ERK signaling cascade. This pathway regulates fundamental cellular processes, such as proliferation, differentiation, and apoptosis [1]. MEK1 and MEK2 function as dual-specificity kinases that phosphorylate tyrosine and threonine residues in ERK1/2, thereby promoting signal propagation through the MAPK cascade [2,3,4]. The oncogenic relevance of the MAPK cascade and development of MEK-targeted therapeutics have been extensively reviewed, demonstrating the potential of MEK inhibition in clinical oncology [5]. Therefore, MEK1 and MEK2 have emerged as promising therapeutic targets. Multiple inhibitory strategies are being explored both as monotherapies and in combination with other targeted agents and conventional chemotherapeutic approaches [6].

A detailed structural comparison of MEK1 and MEK2 is essential to elucidate their functional similarities and differences, thereby enabling the rational design of selective and potent inhibitors. MEK1 and MEK2 share approximately 80% sequence identity, with the highest degree of conservation located within the kinase domain responsible for catalytic activity [7]. Historically, the development of MEK inhibitors has relied predominantly on the crystal structure of human MEK1, largely due to the embryonic lethality observed in MEK1 knockout mouse models. MEK inhibitors can be classified into three major categories [8]. Type I MEK inhibitors are non-ATP competitive inhibitors that achieve high selectivity by binding to an allosteric site adjacent to the ATP-binding pocket [9]. Type II MEK inhibitors are ATP-competitive inhibitors that directly bind to the active sites and interfere with ATP binding. Type III MEK inhibitors are also non-ATP-competitive inhibitors but induce conformational changes in MEK that allosterically inhibit substrate phosphorylation [10]. Despite the extensive structural characterization of MEK1, the structural information on MEK2 remains limited, with only one crystal structure of human MEK2 bound to the inhibitor 5EA and ATP-Mg reported to date [7]. Nevertheless, recent studies have begun to identify biological and functional features that distinguish MEK2 from MEK1 [11].

Exploiting the structural differences between MEK2 and MEK1 may facilitate the development of isoform-specific inhibitors with improved efficacy and reduced off-target toxicity [12]. In this study, we report the crystal structures of human MEK2 in complex with inhibitors that were previously co-crystallized with MEK1. Furthermore, this study identifies the distinct structural features of MEK2 and characterizes its oligomeric state, which together provide a structural basis for the differential binding behaviors of MEK1 and MEK2 toward the same inhibitors.

## 2. Results

### 2.1. Structural Comparison of Inhibitor-Bound MEK2 and MEK1 with Isoform-Specific Flexibility Analysis

**Figure 1:**
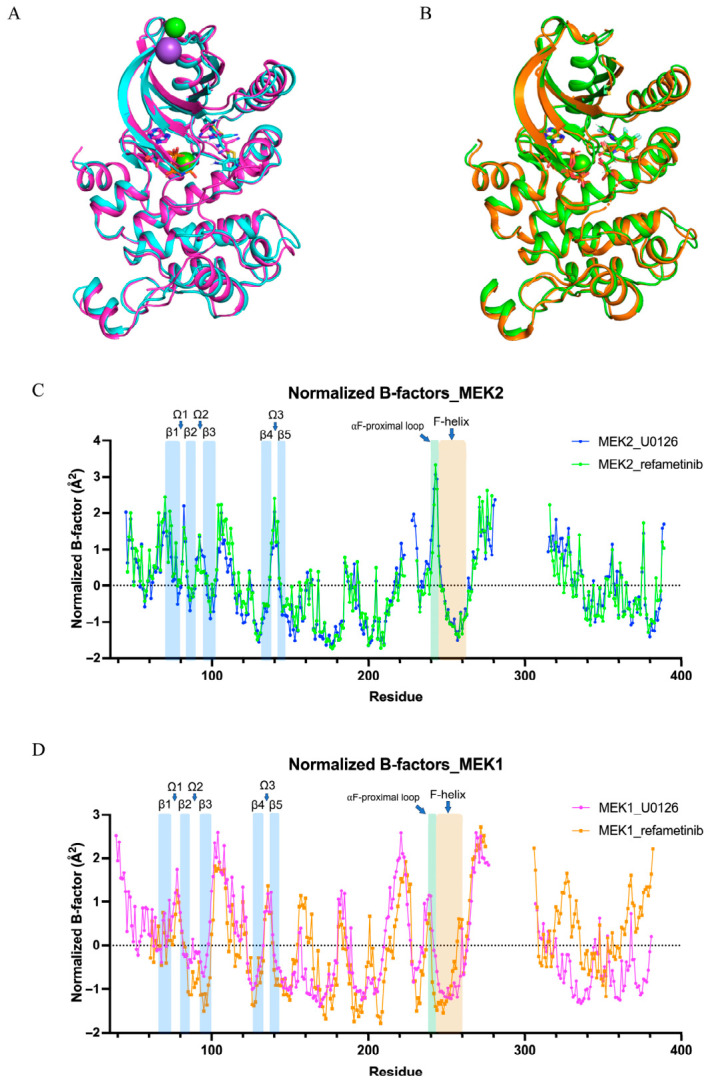
Comparisons of overall structures of human MEK2 and MEK1 bound to U0126 and refametinib, and normalized B-factors. (**A**) Structural alignment of U0126-bound MEK2 with U0126-bound MEK1 (PDB ID: 3EQH). MEK2 is illustrated in cyan and MEK1 in magenta. (**B**) Structural alignment of refametinib-bound MEK2 with refametinib-bound MEK1 (PDB ID: 3E8N). MEK2 is illustrated in green and MEK1 in orange. (**C**,**D**) Comparison of normalized B-factors between the U0126- and refametinib-bound structures of MEK2 and MEK1. The lines representing the normalized B-factors of MEK2–U0126 and MEK2–refametinib complexes are colored in blue and green, respectively. The lines representing the normalized B-factors of MEK1–U0126 and MEK1–refametinib complexes are colored in magenta and orange. The β-strands in the N-lobe are highlighted with light blue boxes, and locations of omega loops are indicated between corresponding β-strands. The loop located at the ⍺F-proximal loop is highlighted with light green boxes, and F-helix is highlighted with beige boxes.

To define the structural determinants of inhibitor recognition in human MEK2, the crystal structures of human MEK2 in complex with U0126 and refametinib were determined at resolutions of 3.15 Å and 3.30 Å, respectively (Figure 1A,B). Both inhibitor-bound MEK2 structures displayed global folds that were highly similar to the previously reported MEK2 structure (PDB ID: 1S9I), with no substantial deviations in the overall architecture (Figure S1A). Structural superposition of the U0126-bound and refametinib-bound MEK2 structures with the corresponding MEK1 complexes revealed a high degree of conformational similarity. The all-atom RMSD values were 0.624 Å for U0126-bound MEK2 versus U0126-bound MEK1 and 0.568 Å for refametinib-bound MEK2 versus refametinib-bound MEK1 (Figure 1A,B).

Crystal structure alignment further confirmed that human MEK2 shares the same secondary structural elements as MEK1 and is arranged in an essentially identical manner. In the inhibitor-bound structures, both MEK2 and MEK1 adopted the DFG-in conformation corresponding to the activated state. Despite the overall structural similarity, regional differences were observed in the normalized B-factor values. In the N-lobe, obvious differences were detected around the β3 strand, comprising residues Ile97–His104 in MEK2 and Leu92–His100 in MEK1. The β3 strand contains the conserved catalytic lysine residue (Lys101 in MEK2 and Lys97 in MEK1), which is essential for kinase activity [13]. In MEK2, both U0126- and refametinib-bound structures exhibited similar normalized B-factor values ranging from approximately −1 to 1 Å in the β3 strand region, indicating comparable local stability (Figure 1C). In contrast, MEK1 exhibited lower normalized B-factor values in the refametinib-bound structure than in the U0126-bound structure within the β3 strand region, consistent with greater stabilization of this region by refametinib (Figure 1D). A direct comparison between MEK2 and MEK1 further showed that MEK1 consistently exhibited lower normalized B-factor values than MEK2 along the Ω2 loop in both inhibitor-bound structures, indicating increased flexibility of the N-lobe β sheet region of MEK2.

### 2.2. Conserved Binding Poses Are Observed but Subtle Pocket-Contact Differences Distinguish MEK2 from MEK1 for U0126

**Figure 2:**
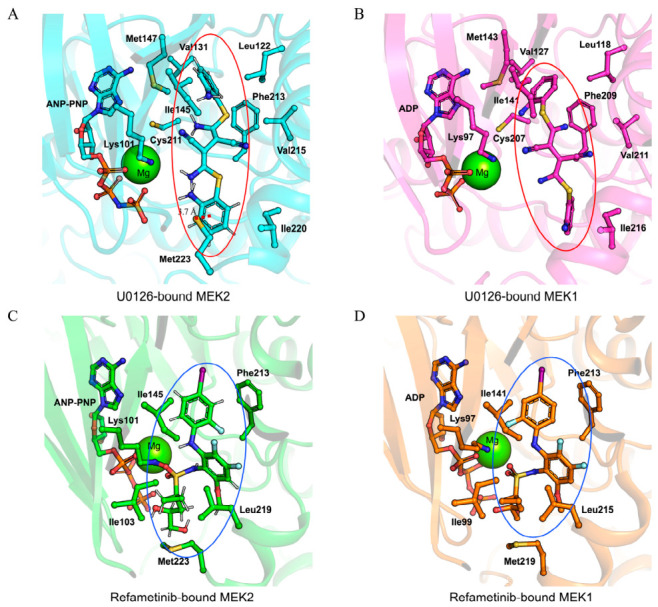
Comparison of residues interacting with U0126 and refametinib in MEK2 and MEK1. (**A**,**B**) Comparison of the binding modes and interacting residues of U0126 in MEK2 and MEK1. U0126 is indicated with red circles. Nitrogen, oxygen, and sulfur atoms are colored blue, red, and yellow, respectively. Carbon atoms are colored cyan for MEK2 and magenta for MEK1. (**C**,**D**) Comparison of the binding modes and interacting residues of refametinib in MEK2 and MEK1. Refametinib is indicated by blue circles. Nitrogen, oxygen, and sulfur atoms are colored blue, red, and yellow, respectively. Carbon atoms are colored green for MEK2 and orange for MEK1.

In the MEK2–U0126 complex, U0126 forms an electrostatic interaction with the conserved catalytic lysine Lys101 and hydrophobic effects with Leu122, Val131, Ile145, Cys211, Phe213, and Val215 (Figure 2A and Figure S2A). The corresponding interacting residues in MEK1 are Lys97, Leu118, Val127, Ile141, Cys207, Phe209, and Val211 (Figure 2B and Figure S2A). Notably, Met223 in MEK2 is located within 3.7 Å of the phenyl ring of U0126, providing an additional hydrophobic contact. This contact is not observed in MEK1, where the equivalent residue Met219 is positioned 6.3 Å away from the phenyl ring of U0126 (Figure 2A,B). In a similar manner, in the MEK2–refametinib complex, refametinib also interacts with Lys101 and forms hydrophobic contacts with Ile103, Ile145, Phe213, Leu219, and Met223 (Figure 2C and Figure S2B). The corresponding residues in MEK1 are Lys97, Ile99, Ile141, Phe209, Leu215, and Met219 (Figure 2D and Figure S2B). In contrast to U0126, refametinib exhibited a highly similar interaction pattern in MEK2 and MEK1, with no significant differences in the residues involved.

Altogether, MEK2 and MEK1 adopt highly conserved binding interfaces for both U0126 and refametinib. While U0126 shows a minor isoform-specific difference, most notably involving Met223 in MEK2 and Met219 in MEK1, the overall ligand-recognition surfaces remain similar. Although U0126 exhibits minor residue-specific differences, most notably at Met223 in MEK2 and the corresponding Met219 in MEK1, the overall ligand-recognition surfaces remain highly similar. Thus, structural elements may contribute to isoform-specific inhibitory responses, potentially through differences in the surrounding structural regions or dynamic conformational properties.

### 2.3. Comparative Analyses of Ligand-Binding Pockets and Helix C–Activation Segment Angles in Human MEK1 and Human MEK2

**Figure 3:**
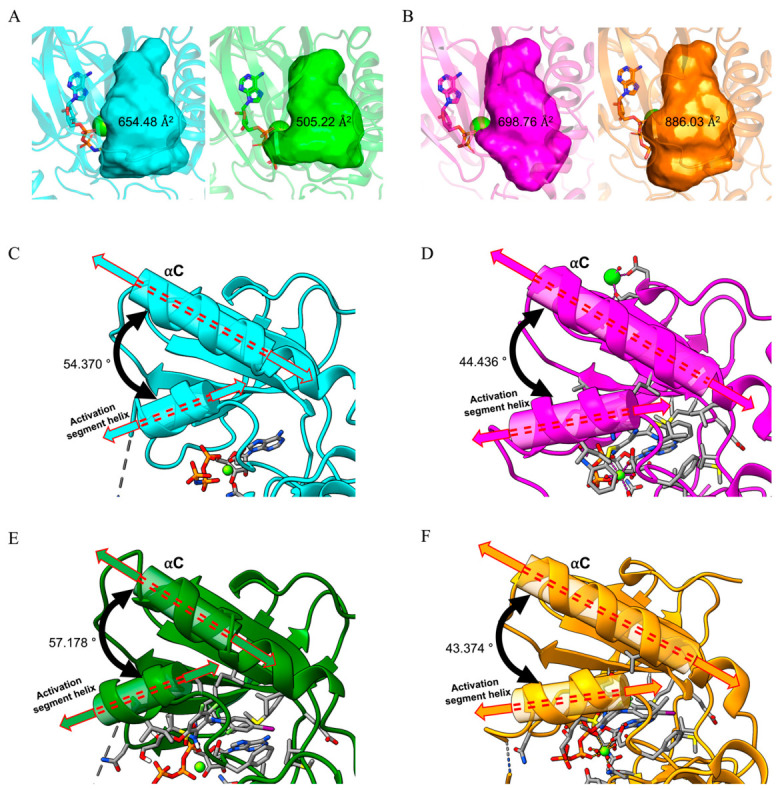
Structural comparison of ligand-binding pocket volumes and the relative orientation between helix C and the activation segment helix in MEK2 and MEK1. (**A**) Ligand-binding pocket volumes of MEK2–U0126 and MEK2–refametinib complexes. The binding pockets identified from the MEK2–U0126 and MEK2–refametinib complexes are illustrated in cyan and green, respectively. (**B**) Ligand-binding pocket volumes of the MEK1–U0126 and MEK1–refametinib complexes. The binding pockets identified from the MEK1–U0126 and MEK1–refametinib complexes are illustrated in magenta and orange, respectively. All ligand-binding pocket volumes were calculated using KVFinder-web v1.1.1 [14]. (**C**,**E**) Helix–helix architecture of MEK2–U0126 and MEK2–refametinib complexes, illustrated in cyan and green, respectively, in ChimeraX. (**D**,**F**) Helix–helix architecture of MEK1–U0126 and MEK1–refametinib complexes, illustrated in magenta and yellow, respectively, in ChimeraX. Helix identification, inter-helical angle measurements, and structural visualization were performed using built-in tools in UCSF ChimeraX (v.1.8) [15].

The volumes of the ligand-binding pockets in the determined human MEK2 and MEK1 structures were analyzed with KVFinder [14]. The binding pocket volumes of the MEK2 in complex with U0126 and refametinib were 654.48 Å3 and 505.22 Å3, respectively (Figure 3A). In contrast, the corresponding pocket volumes in MEK1 were 698.76 Å3 and 886.03 Å3, respectively (Figure 3B), indicating that MEK1 possesses a larger binding pocket than MEK2 in both complexes. The differences in cavity volume are influenced by the degree of structural ordering in the activation segment–proximal loop region. In the MEK2–U0126 and –refametinib complex structures, the activation loop (A-loop) spanning Met223 to Thr230 is not visible in the crystal structure. Conversely, the corresponding region Met219–Thr226 is clearly defined in the MEK1–U0126 or –refametinib complex structures (Figure S3). As a result, the ligand-binding pocket of MEK1 extends outward relative to that of MEK2, resulting in a larger binding pocket volume.

Furthermore, the angle between helix-C (αC) in the N-lobe and the activation segment helix was examined. All inhibitor-bound MEK2 and MEK1 structures adopt a DFG-in conformation corresponding to an activated kinase state, wherein αC and A-loop contribute to shaping the ligand-binding pocket. In MEK2, the angles between αC and the activation segment helix were 54.370° and 57.178° in the U0126-bound and refametinib-bound structures, respectively (Figure 3C,E). For MEK1, the corresponding angles were 44.436° and 43.374°, respectively (Figure 3D,F). These angle differences indicate that the relative positioning of αC and the activation segment helix differs in MEK2 and in MEK1, even under the same DFG-in conformation. Furthermore, despite their high sequence similarity, the region immediately following the A-loop in MEK2 exhibits reduced structural stability compared to the corresponding region in MEK1. Collectively, these structural differences suggest that MEK1 maintains a more rigid and pre-organized binding pocket geometry, whereas MEK2 exhibits consistently larger αC–activation segment angles than MEK1. Such differences may influence the shape and geometry of the binding pocket, thereby affecting inhibitor recognition.

### 2.4. R-Spine-Associated Structural Differences in MEK2 and MEK1 in Complex with U0126 and with Refametinib

**Figure 4:**
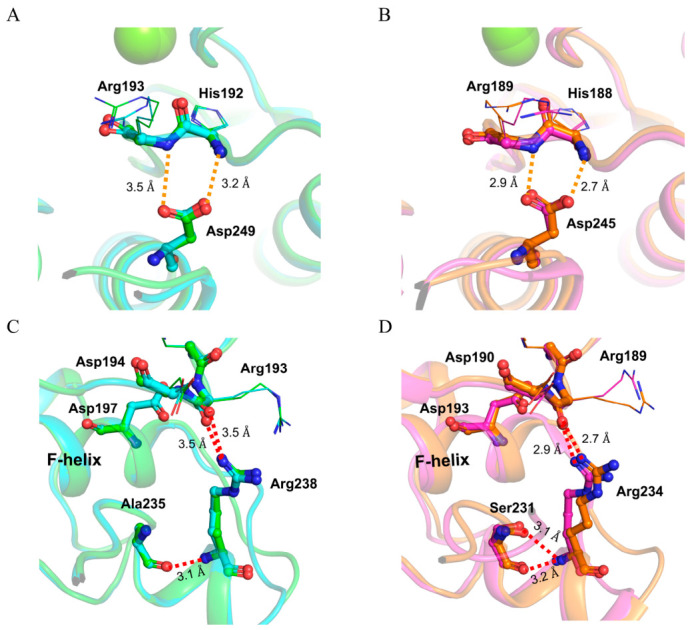
Structural comparison of the ⍺F region and R-spine between MEK2 and MEK1. U0126-bound MEK2, refametinib-bound MEK2, U0126-bound MEK1, and refametinib-bound MEK1 structures are shown in cyan, green, magenta, and orange, respectively. (**A**,**B**) Backbone stabilization of the ⍺F mediated by Asp197 of the HRD motif in MEK2 and MEK1 through bifurcated hydrogen-bond formation in the U0126- and refametinib-bound structures. (**C**,**D**) Comparison of the local structural environments surrounding Arg238 in MEK2 and Arg234 in MEK1, shaped by the presence of Ala235 in MEK2 and Ser231 in MEK1. Distances between the backbone carboxyl groups of Arg193 (MEK2) or Arg189 (MEK1) and the side chains of Arg238 (MEK2) or Arg234 (MEK1) are shown.

The kinase activities of MEK2 and MEK1 are regulated by the assembly of the regulatory spine (R-spine) [16]. The R-spine is composed of four conserved residues: Phe129/Phe134 on the β4 strand, Leu118/Leu122 on the αC, Phe209/Phe213 from the DFG motif, and His188/His192 from the HRD motif in MEK1 and MEK2, respectively [17]. Furthermore, conserved hydrophobic residues on the F-helix (αF) directly interact with R-spine components and function as structural supports that stabilize the spine [18]. In MEK2, the backbone amide groups of His192 and Arg193 form bifurcated hydrogen bonds with the carboxyl group of Asp249 on the αF. A comparable interaction was observed in MEK1, wherein the backbone amide groups of His188 and Arg189 form bifurcated hydrogen bonds with Asp245 (Figure 4A,B). The interaction distances are 3.2–3.5 Å in MEK2 and 2.7–2.9 Å in MEK1. A notable structural difference is observed in the loop at the N-terminal region of αF (Gln240–Val246 in MEK2 and Gln236–Val242 in MEK1). In this region, MEK2 exhibited normalized B-factor values more than 2 Å^2^ higher than those of MEK1, suggesting increased local flexibility (Figure 1C,D). The preceding short helix contains Ala235 in MEK2 and Ser231 in MEK1, representing the only residual difference at this position (Figure S4). In MEK1, the hydroxyl group of Ser231 forms an additional stabilizing hydrogen bond with the backbone carbonyl and guanidinium group of Arg234, with interaction distances of 3.1–3.2 Å. In contrast, Ala235 in MEK2 does not elicit a comparable stabilizing interaction with Arg238 (Figure 4C,D). The guanidinium groups of Arg238 in MEK2 and Arg234 in MEK1 interact with the backbone carboxyl groups of Arg193 and Arg189, respectively. In MEK1, Arg234 forms a hydrogen bond with the backbone carbonyl group of Arg189 at a distance of 2.7–2.9 Å. In contrast, the corresponding interaction in MEK2 occurs at a longer distance (~3.5 Å), indicating a weaker interaction (Figure 4C,D).

### 2.5. Binding Affinities of U0126 and Refametinib to Human MEK2 and the T87F MEK2 Mutant

**Figure 5:**
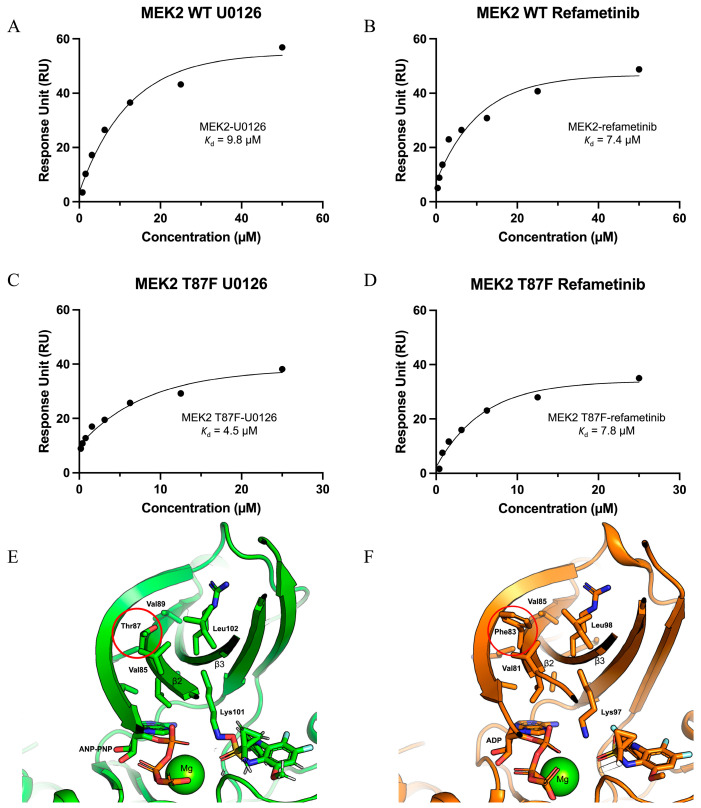
SPR analysis of ligand binding and structural comparison of hydrophobic residues in the N-lobe β-sheet of MEK2 and MEK1. (**A**,**B**) Binding affinity curves of human MEK2 for U0126 and refametinib determined by SPR, together with the calculated dissociation equilibrium constants. (**C**,**D**) Binding affinity curves of the MEK2 T87F mutant for U0126 and refametinib determined by SPR, together with the calculated dissociation equilibrium constants. (**E**,**F**) Structural comparison of hydrophobic residues forming the N-lobe β-sheet in MEK2 (green) and MEK1 (orange). The key residues that differ between MEK2 and MEK1, Thr87 in MEK2 and Phe83 in MEK1, are indicated by red circles.

Surface plasmon resonance (SPR) experiments were conducted to quantify the binding affinities of human MEK2 to U0126 and refametinib. The dissociation constants (*K*_d_) of MEK2 for U0126 and Refametinib were 9.8 μM and 7.4 μM, respectively (Figure 5A,B). To assess the role of this residue in inhibitor binding, SPR experiments were performed using a MEK2 T87F mutant. The MEK2 T87F mutant exhibited *K*_d_ values of 4.5 μM for U0126 and 7.8 μM for refametinib (Figure 5C,D). Despite the high sequence similarity between MEK2 and MEK1, a key difference in the N-lobe is observed at Thr87 in MEK2 and the corresponding Phe83 in MEK1 (Figure S4). These residues are located on the β2 strand of the N-lobe and are surrounded by hydrophobic residues (Figure 5E). The β3 strand adjacent to β2 contains the catalytic lysine residues (Lys101 in MEK2 and Lys97 in MEK1), which are located within 4 Å of Thr87 in MEK2 and Phe83 in MEK1 (Figure 5E,F). This region is also proximal to C-spine residues, including Val86 in MEK2 and Val82 in MEK1. In MEK1, the hydrophobic Phe83 contributes to stabilization of the N-lobe β-sheet. In contrast, the less hydrophobic Thr87 in MEK2 may weaken the local packing of this β-sheet structure. Compared with MEK2 WT, U0126 exhibited an approximately twofold increase in binding affinity for the MEK2 T87F mutant, indicating that the N-lobe β-sheet environment contributes to U0126 binding. In contrast, refametinib exhibited no significant difference in binding affinity between MEK2 WT and MEK2 T87F, suggesting that its binding is less dependent on the N-lobe β-sheet environment.

### 2.6. Experimental Determination of the Oligomeric State of MEK2 and Comparative MD Simulations of U0126- and Refametinib-Bound MEK1/2

**Figure 6:**
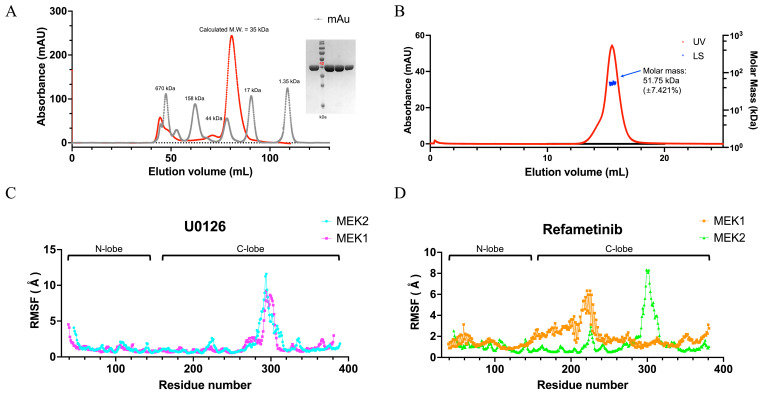
Experimental determination of the oligomeric state of MEK2 and MD simulation analysis of conformational dynamics upon inhibitor binding. (**A**) Size-exclusion chromatography profile of human MEK2. MEK2 is indicated by red dots, and the molecular-weight standards (Bio-Rad #1511901) are shown in gray. The corresponding SDS-PAGE gel is shown on the right. (**B**) Determination of the average molecular weight of MEK2 using SEC–MALS. The red line represents UV absorbance, and the blue line indicates molecular weight during elution. (**C**) Backbone RMSF values per residue from MD simulations of U0126-bound monomeric MEK2 and homodimeric MEK1. MEK2 is shown in cyan, and MEK1 in magenta. (**D**) Backbone RMSF values per residue from MD simulations of refametinib-bound monomeric MEK2 and homodimeric MEK1. MEK2 is shown in green, and MEK1 in orange.

Human MEK2 has been predicted to form a homodimer based on its high sequence identity with MEK1 and by analogy to the first reported MEK1 structure [7]. However, during the purification of human MEK2 (residues L41–Q391), size-exclusion chromatography indicated that MEK2 was eluted at a position corresponding to a molecular weight slightly below 44 kDa, consistent with a monomeric state in solution (Figure 6A). SEC-MALS analysis further supported this observation, yielding an estimated molecular weight of approximately 51.75 kDa in agreement with a monomeric state of human MEK2 (Figure 6B). Size-exclusion chromatography analysis of MEK1 yielded an estimated molecular weight of 84.77 kDa, consistent with its homodimeric state (Figure S5). To investigate the relationship between oligomeric state and conformational dynamics, molecular dynamics simulations were initially performed using monomeric MEK2 and homodimeric MEK1. In the U0126-bound state, the backbone RMSF profiles of monomeric MEK2 and homodimeric MEK1 were largely comparable, with no significant differences in the N-lobe region. Both complexes exhibited elevated RMSF values at residues 280–310 (Figure 6C). In contrast, in the refametinib-bound state, MEK2 exhibited elevated RMSF values at residues 280–310, similar to the pattern observed for U0126. In MEK1, RMSF values remained below 2 Å in residues 280–310. In addition, unlike the other complexes, the refametinib-bound MEK1 structure showed fluctuations of 3–6 Å in residues 150–240 (Figure 6D). This region spans from the hinge region to elements forming the ligand- and ATP-binding pocket. These observations suggest that the structural dynamics of MEK1 and MEK2 differ depending on the bound inhibitors, and that oligomeric state may contribute to these differences. To further distinguish the effects of the oligomeric state from the isoform-specific structural features, additional MD simulations were performed using reciprocal oligomeric models, namely monomeric MEK1 and homodimeric MEK2 (Figure S6). In the U0126-bound state, monomeric MEK1 exhibited increased fluctuations in the distal C-terminal region compared with homodimeric MEK1 (Figure S6A). In the refametinib-bound state, monomeric MEK1 showed a pronounced RMSF increase around residues 290–310, whereas the corresponding region remained relatively restrained in the homodimeric simulation (Figure 6D and Figure S6A). In contrast, homodimeric MEK2 did not exhibit a comparable reduction in flexibility (Figure S6B). Instead, both U0126- and refametinib-bound homodimeric MEK2 displayed elevated RMSF values around residues 285–315, with the largest fluctuations observed in the refametinib-bound complex (Figure S6B). Ligand RMSD values relative to the protein were analyzed for both inhibitors. U0126 exhibited comparable RMSD values in MEK1 and MEK2 following equilibration. In contrast, refametinib showed lower RMSD values in MEK2 than in MEK1 throughout the simulations, indicating reduced positional fluctuations of the bound ligand in MEK2 (Figure S7). These results indicate that the dynamic differences between MEK1 and MEK2 cannot be explained solely by oligomeric state and suggest that the two isoforms respond differently to oligomerization.

## 3. Discussion

This study determined two ligand-bound crystal structures of human MEK2 and provided structural insights into the factors that contribute to the differential binding affinities of MEK2 and MEK1 toward U0126 and refametinib. Structural superposition with previously reported MEK1 complexes revealed that MEK2 binds both allosteric inhibitor refametinib and noncompetitive inhibitor U0126 in highly similar conformations. Among the residues surrounding the active site between the N- and C-lobes, the most prominent difference was at Thr87 in MEK2, corresponding to Phe83 in MEK1. Substitution of phenylalanine for Thr87 in MEK2 enhanced the binding affinity of U0126, whereas refametinib binding remained largely unaffected. This differential sensitivity can be rationalized by the distinct binding characteristics of U0126 and refametinib. U0126 engages a relatively confined hydrophobic pocket near the DFG motif and interacts with fewer residues, making its binding more sensitive to alterations in local β-sheet stability. In contrast, refametinib establishes additional contacts extending toward the Ω-loop between the β1 and β2 strands and interacts with a broader binding surface, rendering it less dependent on the Thr87/Phe83 position (Figure 2). Analysis of the ligand-binding pocket volumes further revealed that MEK2 possesses a smaller pocket than MEK1 (Figure 3A,B). This difference is influenced by the structural organization of the activation segment helix, which interacts primarily with the αC. Structural comparison revealed that the activation segment helix in MEK2 adopted a more open configuration, with an angle approximately 10° wider than that of MEK1 (Figure 3C–F). While the N-terminal region of αC near the DFG motif is positioned similarly, the C-terminal region of αC in MEK2 is displaced outward. This displacement likely reduces stabilization of the subsequent loop region, increases pocket flexibility, and contributes to the observed differences in inhibitor binding affinity between MEK2 and MEK1. Another important structural difference between MEK2 and MEK1 involves the R-spine, which is essential for catalytic activity and strongly influences allosteric inhibitor binding. Among the structural elements that affect R-spine stability, the F-helix (αF) plays a central role. Normalized B-factor analysis revealed that the αF of MEK2 is less stable than that of MEK1 (Figure 1C,D). This reduced stability appears to originate from a short, single-turn helix immediately preceding the αF, where MEK2 contains Ala235 instead of Ser231 in MEK1. This substitution diminishes stabilization of Arg238 in MEK2 (corresponding to Arg234 in MEK1), leading to destabilization of both the loop connecting the activation segment to αF and the N-terminal region of αF itself. Consequently, the interaction between Asp249 in MEK2 and the HRD motif backbone is weakened due to an increased interaction distance. Although Arg238 in MEK2 and Arg234 in MEK1 both interact with the HRD motif, these interactions occur at longer distances in MEK2. Collectively, MEK2 adopts a less well-organized R-spine architecture than MEK1, which may alter the configuration of the allosteric site and contribute to differences in allosteric inhibitor binding between MEK2 and MEK1.

Besides these structural features, our biophysical analyses demonstrated that highly purified human MEK2 exists as a monomer in solution. Despite its high sequence identity with homodimeric MEK1, the oligomeric state of MEK2 has not been determined experimentally. Consistent with previous reports, a corresponding human MEK1 construct analyzed under similar conditions exhibited an apparent molecular weight of approximately 84.77 kDa by size-exclusion chromatography, supporting a homodimeric state in solution (Figure S5). The MEK2 construct used in this study contains a longer sequence than that of the dimeric MEK1 structure, suggesting that MEK2 likely forms a dimer under identical conditions if such an assembly is favorable. Therefore, the observed monomeric behavior of MEK2 therefore represents a fundamental difference between MEK2 and MEK1, and may influence how allosteric inhibitors interact with regulatory proteins such as BRAF, CRAF, KSR1, and KSR2. Additional MD simulations using reciprocal oligomeric models suggest that the distinct dynamic properties of MEK1 and MEK2 arise from intrinsic isoform-specific structural features in addition to differences in oligomeric state. Consistent with these observations, ligand RMSD analysis revealed that refametinib exhibited lower positional fluctuations in MEK2 than in MEK1, whereas U0126 displayed comparable stability in both isoforms (Figure S7). These findings further support the existence of isoform-dependent differences in inhibitor recognition.

A limitation of this study is that the structures of MEK2 in complex with a broader range of inhibitors could not be determined. Although crystallization with several additional inhibitors was attempted to determine the MEK1 structures, the resulting diffraction qualities were insufficient for structure determination. Structural characterization using additional inhibitors would enable a more comprehensive comparison with MEK1 and further clarify the determinants of isoform-selective inhibition. While recent studies have largely focused on MEK1 to elucidate the mechanistic basis of MAPK signaling [19,20], MEK2 remains comparatively underexplored. Our findings suggest that MEK2 may play regulatory roles distinct from those of MEK1 in the MAPK pathway. Given the fact that differential inhibition of MEK2 and MEK1 influences neural stem cell differentiation, selective targeting of each kinase may provide opportunities for the development of novel therapeutic agents with improved specificity [21].

Further studies defining the MEK2 interaction partners and signaling roles are essential to fully elucidate its regulatory functions. Collectively, this study provides a comprehensive structural comparison of MEK2 and MEK1 in complex with clinically relevant inhibitors and establishes a foundation for the structure-guided development of next-generation MEK-targeted therapies.

## 4. Materials and Methods

### 4.1. Cloning, Expression, and Purification

The full length of cDNA for human MEK2 was synthesized for the *E. coli* expression system. The construct for purification was optimized for better expression and crystallization with the XtalPred server, and the result range of human MEK2 was L41-Q391. The sequence of L41-Q391 of human MEK2 was amplified by PCR and inserted into the expression vectors, including maltose binding protein or SUMO to increase the expression level and its solubility. The cloned vector was validated through sequencing and subsequently transformed into BL21(DE3) *E. coli* strain competent cells. The transformed cells were incubated at 37 °C in Luria Broth medium until the optimal density at 600 nm (OD600) reached 0.8–1.0, followed by induction with 1 mM IPTG (1-thio-β-D-galactopyranoside) for 18–21 h at 22 °C. The induced cells were harvested by centrifugation at 6000× *g* for 10 min at 4 °C and sonicated after adding buffer A (20 mM Tris-HCl pH 8.0, 500 mM NaCl, 20 mM imidazole, 10% (*v*/*v*) glycerol, and 0.5 mM TCEP). The lysate was centrifuged at 30,000× *g* for 1 h at 4 °C to eliminate cellular debris. The supernatant was filtered through a 0.45 µm syringe filter, and the filtrate was applied to a Ni^2+^-charged HiTrap chelating HP column (GE Healthcare, Chicago, IL, USA). The protein-loaded column was equilibrated with buffer A, and the bound human MEK2 was eluted using buffer B (20 mM Tris-HCl pH 8.0, 500 mM NaCl, 300 mM imidazole, 10% (*v*/*v*) glycerol, and 0.5 mM TCEP) with a linear gradient. The bound human MEK2 was eluted with 100–200 mM imidazole, then the buffer was changed to buffer C (20 mM Tris-HCl pH 8.0, 150 mM NaCl, 10% (*v*/*v*) glycerol, and 0.5 mM TCEP) using a HiPrep Desalting 26.10 column (GE Healthcare, Chicago, IL, USA) for cleaving solubilization tags with TEV protease. The TEV protease-treated human MEK2 was incubated at 4 °C for 18 h. The cleaved human MEK2 was loaded onto the pre-equilibrated Ni^2+^ HiTrap chelating HP column with buffer A, and the loading-through was collected to exclude solubilization tags. The loading-through protein was further purified by size-exclusion chromatography using HiLoad 16/600 Superdex 200 pg (GE Healthcare, Chicago, IL, USA), pre-equilibrated with a storage buffer consisting of 20 mM HEPES-NaOH pH 7.5, 200 mM NaCl, 2 mM MgCl_2_ hexahydrate, and 0.5 mM TCEP. Each step of purification was validated by SDS-PAGE analysis of the fractions. For surface plasmon resonance (SPR) and size-exclusion chromatography coupled with multi-angle light scattering (SEC-MALS) experiments, the sequence of L41-Q391 of human MEK2 was amplified by PCR and inserted into the pET28a expression vectors. The cloned vector was used to make the human MEK2 T87F mutant using the site-directed mutagenesis protocol. The cloned vectors were transformed into BL21(DE3) *E. coli* strain competent cells and induced with IPTG in the same way as described above. The expressed protein was purified using the same procedure as described above up to the affinity chromatography step. Subsequently, ion exchange chromatography was performed on a HiTrap Q HP column (GE Healthcare, Chicago, IL, USA) using buffer D (20 mM Tris-HCl, pH 7.4, 50 mM NaCl) and buffer E (20 mM Tris-HCl, pH 8.4, 1 M NaCl). Finally, the protein was further purified by size-exclusion chromatography using HBS buffer (10 mM HEPES-NaOH, pH 7.4, 150 mM NaCl). Human MEK1 (residues L37–Q383) was expressed and purified using the same procedure as described for human MEK2. This construct was selected to correspond to the region most comparable to the MEK2 construct (L41–Q391). Size-exclusion chromatography was performed using a Superdex 200 Increase 10/300 GL column (GE Healthcare, Chicago, IL, USA).

### 4.2. Size-Exclusion Chromatography–Multi-Angle Light Scattering

The absolute molecular weight and oligomeric state of human MEK2 were determined using size-exclusion chromatography coupled with multi-angle light scattering (SEC-MALS). The highly purified MEK2 was loaded onto a Superdex 200 10/300 GL column (Cytiva, Marlborough, MA, USA) pre-equilibrated with HBS buffer (10 mM HEPES-NaOH, pH 7.4, 150 mM NaCl). Chromatographic separation was performed at a flow rate of 0.5 mL/min using an ÄKTA pure system (Cytiva, Marlborough, MA, USA). The SEC column was connected in-line to miniDAWN (Wyatt Technology, Santa Barbara, CA, USA), which was pre-equilibrated and stabilized with HBS buffer. Absolute molecular weight values were calculated by combining light-scattering data with concentration information derived from the chromatographic elution profile. The refractive index increment (Δn/Δc) was set to 0.185 mL/g, assuming a standard value for globular proteins. Data acquisition and analysis were performed using ASTRA software (v.8.2.0) (Wyatt Technology, Santa Barbara, CA, USA). Molecular weight distributions were calculated using the Zimm model, and reported molecular weights represent averages across the main elution peak. All measurements were conducted at room temperature (25 °C).

### 4.3. Crystallization and X-Ray Diffraction Data Collection

The crystallization conditions for human MEK2 preincubated with U0126 and Refametinib were screened with commercial screening kits (Hampton Research, Aliso Viejo, CA, USA). Before crystallization, U0126 and refametinib were pre-incubated with highly purified human MEK2 in a molar ratio of 1:5 for more than 1 h at 4 °C. The pre-incubated MEK2–inhibitor mixtures were diluted to 10 mg/mL–12 mg/mL for crystallization. For human MEK2 binding U0126, initial crystals were grown in a condition containing 0.2 M ammonium acetate, 0.1 M Tris-HCl pH 8.5, and 25% (*w*/*v*) polyethylene glycol 3350. For refametinib-bound human MEK2, initial crystals were grown in a condition containing 0.2 M sodium phosphate dibasic dihydrate, 0.1 M Tris-HCl pH 8.5, and 20% (*w*/*v*) polyethylene glycol 3350. Crystals were grown at 22 °C using the hanging-drop vapor diffusion method. Prior to collecting diffraction data, all crystals were cryo-protected with 20% (*w*/*v*) glycerol with reservoir solution and flash-frozen in liquid nitrogen. The X-ray diffraction data of human MEK2 binding U0126 and Refametinib were collected on the BL-5C at the Pohang Accelerator Laboratory, Pohang, Republic of Korea. The collected data were indexed, integrated, and scaled using the HKL2000 package (v721.3) [22].

### 4.4. Structure Determination with Phasing and Refinement

For both U0126 and refametinib binding human MEK2, 5% of the total data were randomly excluded to serve as the R*_free_* set, and the remaining data were employed to calculate R*_work_*. The phasing problem for both inhibitor-bound human MEK2 was solved by molecular replacement by MolRep in the CCP4i suite (v8.0.019) and Phaser in Phenix (v1.21.2) [23,24]. Then the primary models were refined manually by fitting them into the electron density map using COOT (v0.9.8.93) [25]. The manually refined model was then further refined with Phenix until the resulting structure satisfied validation criteria from MolProbity (v4.5.2) [26]. After fitting the protein model into the electron density map, ligands generated from the electron Ligand Builder and Optimization Workbench (eLBOW) were fitted into the omit map [27]. The final models were refined using phenix.refine implemented in the PHENIX software package (v.2.0). The statistics of the data collection and structure refinement are summarized in Table 1.

### 4.5. Molecular Dynamics Simulations

The MD simulations were implemented using the Desmond simulation package (v.2024-4) distributed by Schrodinger LLC. All simulations were carried out using the OPLS4 force field [28]. Because the missing region could not be fully modeled by homologous loop modeling, the unresolved portions were reconstructed using loop fragments generated from both the AlphaFold3 (v.3.0.2) and SWISS-MODEL predictions [29,30]. The resulting models were evaluated based on their geometries, and the model with the most favorable geometry was selected for subsequent simulations. The refined MEK2 structures and already solved structures of human MEK1 (PDB ID: 3E8N, 3EQH) bound with U0126 and refametinib were prepared by using the Protein Preparation Workflow (PDB ID: 21PA and 21OA) [31]. The homodimeric MEK1 model was reconstructed from the biological assembly corresponding to the dimeric arrangement previously described by Ohren et al. using PDB entries 3E8N and 3EQH. Although only one MEK1 molecule is present in the asymmetric unit of these structures, the biologically relevant homodimer was generated by applying the crystallographic symmetry operators provided in the deposited coordinates. The monomeric MEK1 model was generated by extracting a single protomer from the reconstructed homodimer. A hypothetical homodimeric MEK2 model was generated by superimposing the experimentally determined MEK2 crystal structure onto both protomers of the MEK1 homodimer using Cα atoms, thereby preserving the dimer arrangement observed in MEK1. This model was used solely for comparative MD simulations and does not represent an experimentally validated oligomeric state of MEK2. The prepared systems were solvated in an orthorhombic TIP4P water box with a 10 Å buffer distance from the protein surface. Counterions were added to neutralize the system, and NaCl was added to a final concentration of 0.15 M. Following energy minimization, each system was equilibrated for 20 ns prior to production simulations. Each simulation was performed three times for 300 ns under the NPT ensemble conditions at 300 K and 1 bar. Simulations were performed under NPT conditions at 300 K and 1 bar using the Nose–Hoover thermostat and Martyna–Tobias–Klein barostat.

### 4.6. Surface Plasmon Resonance

Surface plasmon resonance (SPR) experiments were performed on a Biacore T200 system (Cytiva, Marlborough, MA, USA) using a Sensor Chip NTA (Cytiva, Uppsala, Sweden). High-purity human MEK2 and its T87F mutant were immobilized on the chip via their N-terminal hexahistidine tags. The concentration of each immobilized protein was standardized to 30 µg/mL with HBS buffer. The analytes U0126 and refametinib were prepared in running buffer consisting of 10 mM HEPES-NaOH pH 7.4, 150 mM NaCl, and 2% (*v*/*v*) DMSO and serially diluted to obtain the desired concentrations. Each measurement cycle was conducted independently. Prior to immobilization, the NTA surface was charged with Ni^2+^ by injecting 0.5 mM NiCl_2_ at a flow rate of 5 µL/min for 2 min, followed by washing with 3 mM EDTA at the same flow rate for 30 s. The ligand proteins were then immobilized at 5 µL/min for 90 s and allowed to stabilize for 240 s. During the association phase, analytes were injected at a flow rate of 30 µL/min for 120 s, followed by a 90 s dissociation phase. At the end of each cycle, the surface was regenerated using a solution containing 350 mM EDTA, 6 M urea, and 50 mM NaOH to completely remove bound proteins and restore the Ni^2+^-NTA surface.

## Figures and Tables

**Table 1:** Crystallographic data collection and refinement statistics.

	MEK2–U0126 Complex	MEK2–Refametinib Complex
Data collection		
Beamline	PAL-5C	PAL-5C
Wavelength (Å)	0.9795	0.9795
Space group	P2_1_2_1_2	P2_1_2_1_2
a, b, c (Å)	149.7, 261.5, 75.9	149.3, 261.7, 75.4
⍺, β, γ (°)	90.0, 90.0. 90.0	90.0, 90.0, 90.0
Resolution (Å) ^a^	49.54–2.85 (2.93–2.85)	49.40–3.30 (3.42–3.30)
Unique reflections	56,175 (1831)	45,229 (2876)
Multiplicity	4.9 (3.9)	6.8 (5.8)
Completeness	80.30 (32.50)	98.44 (91.28)
I/σ (I)	12.0 (3.1)	13.2 (2.7)
*R_meas_* (%) ^b^	11.8 (101.5)	14.3 (72.9)
*R_pim_* (%) ^c^	5.0 (46.2)	5.5 (29.9)
CC_1/2_	99.5 (62.2)	99.1 (86.7)
Refinement		
Resolution (Å) ^a^	49.54–3.15 (3.26–3.15)	49.40–3.30 (3.39–3.30)
Reflections used in refinement	47,190 (3827)	44,524 (2878)
Reflections used for R*_free_*	1487 (135)	2000 (130)
*R_work_*/*R_free_* (%) ^d^	19.58/24.53	21.36/24.66
Bond lengths (Å)	0.004	0.005
Bond angles (°)	0.66	0.74
Number of non-hydrogen atoms	14,874	14,850
Protein	14,441	14,431
Ligand	348	378
Water	85	41
Average B-factor (Å^2^)	48.69	56.08
Protein	48.75	56.07
Ligand	50.48	58.15
Water	31.68	39.61
Ramachandran plot (%)		
Favored	98.21	98.26
Allowed	1.74	1.69
Outliers	0.06	0.06
Poor rotamers (%)	0.00	0.00
PDB ID	21PA	21OA

^a^ Values in parentheses refer to the highest resolution shell. ^b^ $R_{meas}=\frac{\sum_{h}\left(\frac{N_{h}}{N_{h}-1}\sum_{i}\left|I_{h,i}-\left\langle I_{h}\right\rangle \right|\right)}{\sum_{h}\sum_{i}I_{h,i}},$ ^c^ $R_{pim}=\frac{\sum_{h}\left(\frac{1}{\sqrt{N_{h}-1}}\sum_{i}\left|I_{h,i}-\left\langle I_{h}\right\rangle \right|\right)}{\sum_{h}\sum_{i}I_{h,i}}$, $I_{h,i}$ is the intensity of the *i*-th observation of reflection *h*, and *N*_*h* is the multiplicity of reflection *h*. ^d^ $R_{work}=\frac{\sum_{h\in \mathrm{work}}\left|\left|F_{o}\right|-\left|F_{c}\right|\right|}{\sum_{h\in \mathrm{work}}\left|F_{o}\right|},R_{free}=\frac{\sum_{h\in \mathrm{free}}\left|\left|F_{o}\right|-\left|F_{c}\right|\right|}{\sum_{h\in \mathrm{free}}\left|F_{o}\right|}$, where the free set comprises ~5% of the reflections excluded from refinement. Validation statistics were calculated using MolProbity.

## Data Availability

The coordinates and structure factors of MEK2 in complex with U0126 and refametinib have been deposited in the Protein Data Bank (PDB) under the accession IDs 21PA and 21OA, respectively.

## Supplementary Materials

The following supporting information can be downloaded at: https://www.mdpi.com/article/10.3390/ijms27135992/s1. References [32,33] are cited in the supplementary materials.

## Abbreviations

The following abbreviations are used in this manuscript:

MEK2	Mitogen-activated protein kinase kinase 2
MEK1	Mitogen-activated protein kinase kinase 1
TCEP	Tris(2-carboxyethyl) phosphine
RMSF	Root mean square fluctuation
SPR	Surface plasmon Resonance
αC	Helix-C
αF	Helix-F
A-loop	Activation loop
