# Supplementary material for: Structural and Biophysical Analyses of Human MEK2 in Complex with Two Inhibitors Reveal the Determinants of Isoform-Dependent Inhibitor Binding
Source: Int J Mol Sci. 2026 Jul 3;27(13):5992. doi: 10.3390/ijms27135992 (PMC13360787; doi:10.3390/ijms27135992)
Supplement: Supplementary file 1 [file ijms-27-05992-s001.zip › validation_report_21PA.pdf]

Full wwPDB X-ray Structure Validation Report (i)

Jun 19, 2026 – 04:58 PM JST

PDB ID	:	21PA / pdb_000021pa
Title	:	Crystal structure of human MEK2 in complex with U0126
Deposited on	:	2025-12-22
Resolution	:	3.15 Å(reported)

This wwPDB validation report is for manuscript review

For Manuscript Review

This is a Full wwPDB X-ray Structure Validation Report.

This report is produced by the wwPDB biocuration pipeline after annotation of the structure.

We welcome your comments at *validation@mail.wwpdb.org*
A user guide is available at
https://www.wwpdb.org/validation/2017/XrayValidationReportHelp
with specific help available everywhere you see the (i) symbol.

The types of validation reports are described at
http://www.wwpdb.org/validation/2017/FAQs#types.

The following versions of software and data (see references (i)) were used in the production of this report:

MolProbity	:	4-5-2 with Phenix2.0
Mogul	:	1.8.5 (274361), CSD as541be (2020)
Xtriage (Phenix)	:	2.0
EDS	:	3.0
Buster-report	:	wwPDB partial adaption of 1.1.7 (2018)
Percentile statistics	:	20250101.v01 (using entries in the PDB archive January 1st 2025)
CCP4	:	9.0.010 (Gargrove)
Density-Fitness	:	1.0.12
Ideal geometry (proteins)	:	Engh & Huber (2001)
Ideal geometry (DNA, RNA)	:	Parkinson et al. (1996)

1 Overall quality at a glance (i)

The following experimental techniques were used to determine the structure:
X-RAY DIFFRACTION

The reported resolution of this entry is 3.15 Å.

Percentile scores (ranging between 0-100) for global validation metrics of the entry are shown in the following graphic. The table shows the number of entries on which the scores are based.

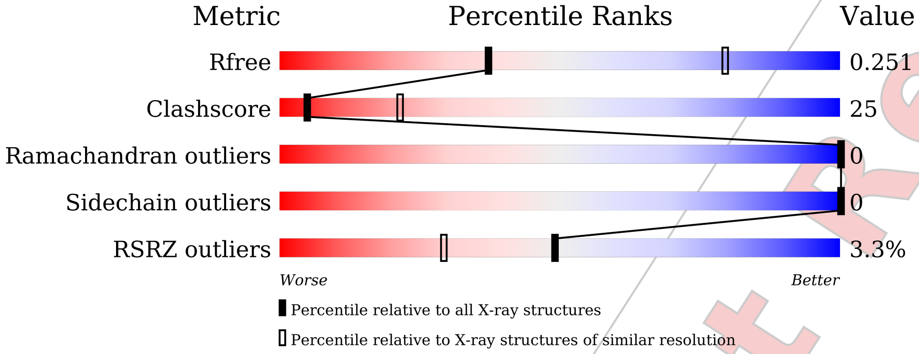

Metric	Whole archive (#Entries)	Similar resolution (#Entries, resolution range(Å))
R_{free}	180053	2361 (3.20-3.12)
Clashscore	190562	2486 (3.20-3.12)
Ramachandran outliers	187476	2405 (3.20-3.12)
Sidechain outliers	187428	2404 (3.20-3.12)
RSRZ outliers	180081	2361 (3.20-3.12)

The table below summarises the geometric issues observed across the polymeric chains and their fit to the electron density. The red, orange, yellow and green segments of the lower bar indicate the fraction of residues that contain outliers for >=3, 2, 1 and 0 types of geometric quality criteria respectively. A grey segment represents the fraction of residues that are not modelled. The numeric value for each fraction is indicated below the corresponding segment, with a dot representing fractions <=5% The upper red bar (where present) indicates the fraction of residues that have poor fit to the electron density. The numeric value is given above the bar.

Mol	Chain	Length	Quality of chain
1	A	345	2% / 52% 36% 12%
1	B	345	3% / 50% 39% 12%
1	C	345	2% / 53% 35% 11%
1	D	345	% / 52% 36% 11%

Continued on next page...

For Manuscript Review

Continued from previous page...

Mol	Chain	Length	Quality of chain
1	E	345	5% 46% 42% 12%
1	F	345	5% 45% 41% 14%

The following table lists non-polymeric compounds, carbohydrate monomers and non-standard residues in protein, DNA, RNA chains that are outliers for geometric or electron-density-fit criteria:

Mol	Type	Chain	Res	Chirality	Geometry	Clashes	Electron density
4	5BM	A	403	-	-	X	-
4	5BM	F	403	-	-	X	-

For Manuscript Review

2 Entry composition (i)

There are 5 unique types of molecules in this entry. The entry contains 15048 atoms, of which 174 are hydrogens and 0 are deuteriums.

In the tables below, the ZeroOcc column contains the number of atoms modelled with zero occupancy, the AltConf column contains the number of residues with at least one atom in alternate conformation and the Trace column contains the number of residues modelled with at most 2 atoms.

- Molecule 1 is a protein called Dual specificity mitogen-activated protein kinase kinase 2.

Mol	Chain	Residues	Atoms	ZeroOcc	AltConf	Trace
1	A	303	Total C N O S<br>2405 1532 415 444 14	0	0	0
1	B	304	Total C N O S<br>2412 1537 416 445 14	0	0	0
1	C	306	Total C N O S<br>2423 1544 418 447 14	0	0	0
1	D	306	Total C N O S<br>2421 1542 418 447 14	0	0	0
1	E	304	Total C N O S<br>2415 1537 419 445 14	0	0	0
1	F	298	Total C N O S<br>2365 1507 407 438 13	0	0	0

- Molecule 2 is MAGNESIUM ION (CCD ID: MG) (formula: Mg).

Mol	Chain	Residues	Atoms	ZeroOcc	AltConf
2	A	1	Total Mg<br>1 1	0	0
2	B	1	Total Mg<br>1 1	0	0
2	C	1	Total Mg<br>1 1	0	0
2	D	1	Total Mg<br>1 1	0	0
2	E	1	Total Mg<br>1 1	0	0
2	F	1	Total Mg<br>1 1	0	0

- Molecule 3 is PHOSPHOAMINOPHOSPHONIC ACID-ADENYLATE ESTER (CCD ID: ANP) (formula: $C_{10}H_{17}N_6O_{12}P_3$) (labeled as "Ligand of Interest" by depositor).

For Manuscript Review

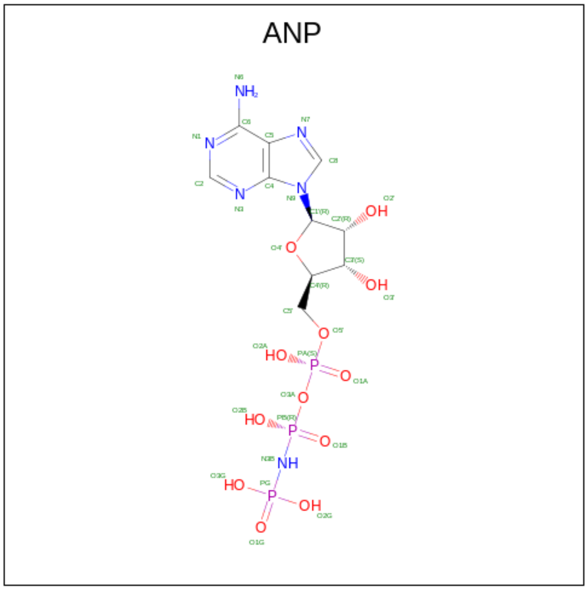

Mol	Chain	Residues	Atoms						ZeroOcc	AltConf
3	A	1	Total 44	C 10	H 13	N 6	O 12	P 3	0	0
3	B	1	Total 44	C 10	H 13	N 6	O 12	P 3	0	0
3	C	1	Total 44	C 10	H 13	N 6	O 12	P 3	0	0
3	D	1	Total 44	C 10	H 13	N 6	O 12	P 3	0	0
3	E	1	Total 44	C 10	H 13	N 6	O 12	P 3	0	0
3	F	1	Total 44	C 10	H 13	N 6	O 12	P 3	0	0

- Molecule 4 is (2Z)-bis{amino[(2-aminophenyl)sulfanyl]methylidene}butanedinitrile (CCD ID: 5BM) (formula: $C_{18}H_{16}N_6S_2$) (labeled as "Ligand of Interest" by depositor).

For Manuscript Review

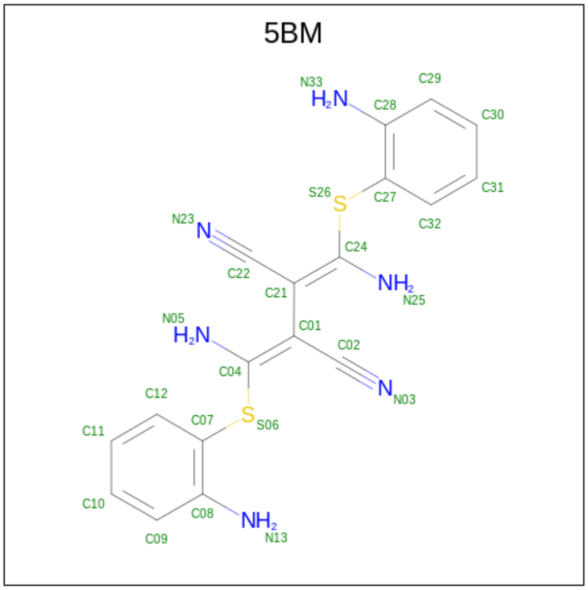

Mol	Chain	Residues	Atoms					ZeroOcc	AltConf
			Total	C	H	N	S		
4	A	1	42	18	16	6	2	0	0
4	B	1	42	18	16	6	2	0	0
4	C	1	42	18	16	6	2	0	0
4	D	1	42	18	16	6	2	0	0
4	E	1	42	18	16	6	2	0	0
4	F	1	42	18	16	6	2	0	0

- Molecule 5 is water.

Mol	Chain	Residues	Atoms		ZeroOcc	AltConf
			Total	O		
5	A	14	14	14	0	0
5	B	16	16	16	0	0
5	C	12	12	12	0	0
5	D	13	13	13	0	0
5	E	11	11	11	0	0
5	F	19	19	19	0	0

For Manuscript Review

3 Residue-property plots (i)

These plots are drawn for all protein, RNA, DNA and oligosaccharide chains in the entry. The first graphic for a chain summarises the proportions of the various outlier classes displayed in the second graphic. The second graphic shows the sequence view annotated by issues in geometry and electron density. Residues are color-coded according to the number of geometric quality criteria for which they contain at least one outlier: green = 0, yellow = 1, orange = 2 and red = 3 or more. A red dot above a residue indicates a poor fit to the electron density (RSRZ > 2). Stretches of 2 or more consecutive residues without any outlier are shown as a green connector. Residues present in the sample, but not in the model, are shown in grey.

• Molecule 1: Dual specificity mitogen-activated protein kinase kinase 2

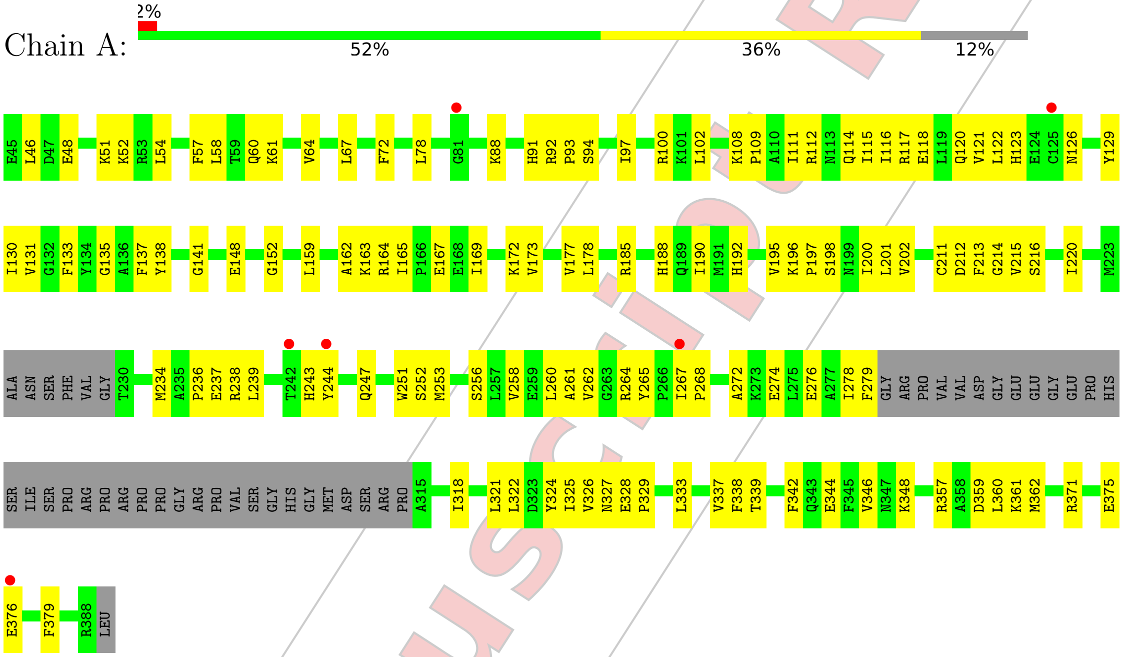

• Molecule 1: Dual specificity mitogen-activated protein kinase kinase 2

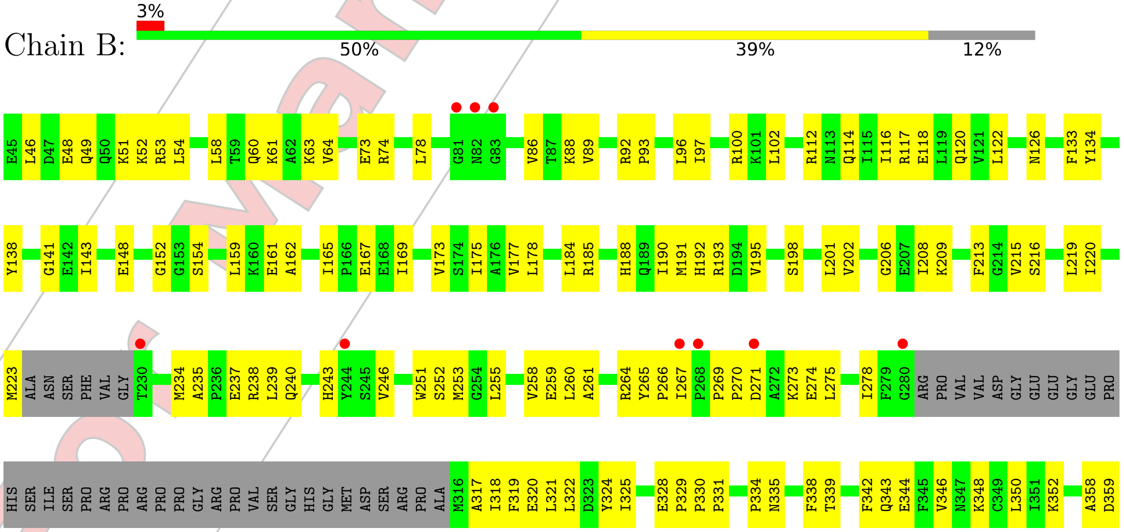

For Manuscript Review

M362 L363 I369 K370 R371 S372 E373 V374 E375 F379 T386 L389

• Molecule 1: Dual specificity mitogen-activated protein kinase kinase 2

Chain C: 2% 53% 35% 11%

E45 L46 Q49 Q50 R53 L54 F57 L58 V64 L67 D71 R74 L78 G81 N82 V86 T87 K88 V89 Q90 H91 R92 P93 S94 G95 L96 R100 K101 L102 I103 H104 L105 E106 I107 K108 P109 A110 I111 R112 N113 Q114 I115 E118 L122 N126 S127

P128 Y129 I130 V131 G132 F133 Y134 G135 Y138 G141 E142 I143 G152 G153 S154 Q157 R164 I165 P166 E167 E168 I169 K172 V173 S174 I175 A176 V177 L178 H188 Q189 I190 M191 H192 R193 D194 N199 V202 G206 E207 L210 C211 D212 F213 G214 V215 Q218 L219

M223 ALA ASN SER PHE V228 G229 T230 M234 E237 R238 L239 Q240 H243 Y244 I250 W251 S252 M253 G254 L255 L260 A261 R264 Y265 P266 I267 P268 P269 P270 D271 E274 I278 F279 G280 ARG PRO VAL VAL ASP GLY GLU GLU GLY GLU PRO HIS SER ILE SER PRO

ARG PRO ARG PRO PRO GLY ARG PRO VAL SER GLY HIS GLY MET ASP SER ARG PRO ALA M316 L322 D323 Y324 I325 V326 N327 E328 P329 P330 P334 V337 F338 T339 F342 K352 D359 L360 K361 M362 I369 E373 D378 F379 A380 G381 K385 R388 L389

• Molecule 1: Dual specificity mitogen-activated protein kinase kinase 2

Chain D: % 52% 36% 11%

E45 L46 D47 E48 Q49 Q50 K51 K52 E55 A56 F57 L58 T59 Q60 K61 A62 K63 V64 L67 E73 R74 L78 G79 K88 V89 Q90 H91 R92 P93 S94 G95 L96 I97 M98 L102 I103 H104 L105 I111 R112 N113 Q114 I115 I116 R117 E118 L119 Q120 V121 L122 N126 S127

P128 Y129 G132 F133 Y134 G135 Y138 S139 D140 G141 E142 I143 C146 M147 E148 G152 D156 Q157 V158 L159 K160 E161 A162 K163 R164 I165 P166 E167 E168 I169 V173 S174 I175 K187 H188 Q189 I190 M191 H192 R193 V202 N203 I208 F213 G214 V215 S216 I220

M223 ALA ASN SER PHE VAL G229 T230 R231 M234 A235 P236 E237 R238 Y244 I250 W251 L255 V258 E259 L260 A261 V262 G263 R264 Y265 P266 I267 P268 P269 P270 E274 I278 F279 G280 ARG PRO VAL VAL ASP GLY GLU GLU GLY GLU PRO HIS SER ILE SER PRO ARG PRO

ARG PRO PRO GLY ARG PRO VAL SER GLY HIS GLY MET ASP SER ARG PRO A315 M316 A317 I318 F319 E320 L321 L322 D323 Y324 E328 P329 N335 G336 V337 F342 Q343 L350 A358 M362 L363 T364 N365 I369 K370 E373 V377 D378 F379 T386 L389

• Molecule 1: Dual specificity mitogen-activated protein kinase kinase 2

Chain E: 5% 46% 42% 12%

E45 L46 D47 E48 Q49 Q50 K51 K52 R53 L54 E55 L58 T59 Q60 K61 L67 D71 F72 E73 R74 E77 L78 G79 A80 G81 N82 T87 K88 V89 Q90 H91 R92 P93 S94 G95 L96 I97 M98 A99 R100 I103 H104 L105 P109 A110 I111 R112 N113 Q114 I115 I116 R117 E118

L119 Q120 C125 V131 G132 F133 Y134 Y138 G141 E142 I143 S144 I145 C146 G152 G153 S154 L155 D156 L159 K160 E161 A162 E167 E168 I169 L170 G171 I175 A176 V177 L178 L184 R185 H188 Q189 I190 M191 H192 R193 D194 V195 S198 L201 V202 R205 G206

For Manuscript Review

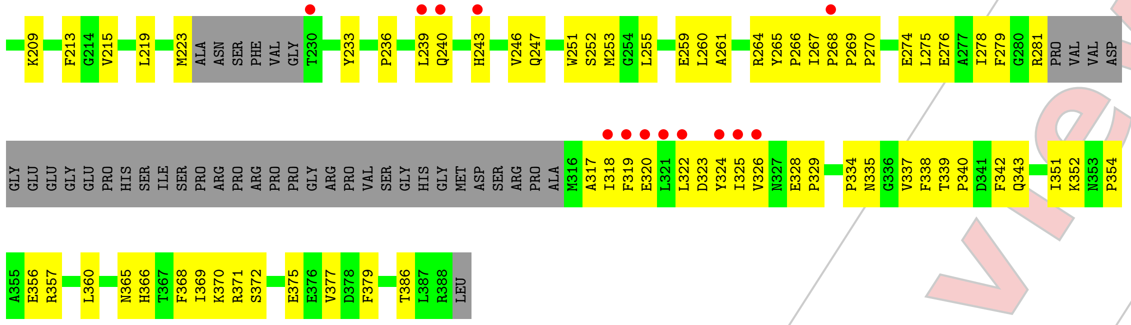

• Molecule 1: Dual specificity mitogen-activated protein kinase kinase 2

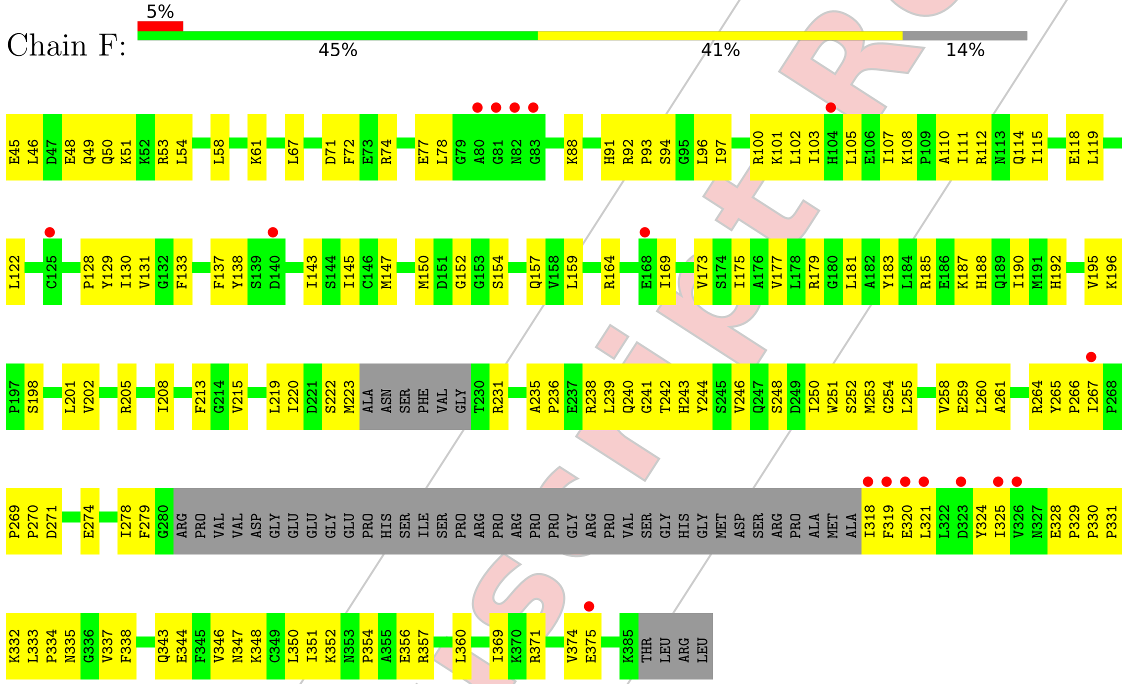

For Manuscript Review

4 Data and refinement statistics (i)

Property	Value	Source
Space group	P 21 21 2	Depositor
Cell constants<br>a, b, c, α, β, γ	149.68Å 261.54Å 75.89Å<br>90.00° 90.00° 90.00°	Depositor
Resolution (Å)	49.54 – 3.15<br>49.54 – 3.15	Depositor<br>EDS
% Data completeness<br>(in resolution range)	90.0 (49.54-3.15)<br>89.9 (49.54-3.15)	Depositor<br>EDS
R_{merge}	(Not available)	Depositor
R_{sym}	(Not available)	Depositor
$< I/\sigma(I) >$ [1]	3.29 (at 3.12Å)	Xtriage
Refinement program	PHENIX 2.0_5885	Depositor
R, R_{free}	0.202 , 0.248<br>0.206 , 0.251	Depositor<br>DCC
R_{free} test set	2000 reflections (3.26%)	wwPDB-VP
Wilson B-factor (Å^2)	51.9	Xtriage
Anisotropy	0.095	Xtriage
Bulk solvent k_{sol}(e/Å^3), B_{sol}(Å^2)	0.33 , 49.4	EDS
L-test for twinning[2]	$< \|L\| > = 0.47$, $< L^2 > = 0.30$	Xtriage
Estimated twinning fraction	No twinning to report.	Xtriage
F_o,F_c correlation	0.91	EDS
Total number of atoms	15048	wwPDB-VP
Average B, all atoms (Å^2)	50.0	wwPDB-VP

Xtriage's analysis on translational NCS is as follows: *The largest off-origin peak in the Patterson function is 14.16% of the height of the origin peak. No significant pseudotranslation is detected.*

[1] Intensities estimated from amplitudes.

[2] Theoretical values of $< |L| >$, $< L^2 >$ for acentric reflections are 0.5, 0.333 respectively for untwinned datasets, and 0.375, 0.2 for perfectly twinned datasets.

For Manuscript Review

5 Model quality (i)

5.1 Standard geometry (i)

Bond lengths and bond angles in the following residue types are not validated in this section: MG, ANP, 5BM

The Z score for a bond length (or angle) is the number of standard deviations the observed value is removed from the expected value. A bond length (or angle) with $|Z| > 5$ is considered an outlier worth inspection. RMSZ is the root-mean-square of all Z scores of the bond lengths (or angles).

Mol	Chain	Bond lengths		Bond angles	
		RMSZ	$\#\|Z\| >5$	RMSZ	$\#\|Z\| >5$
1	A	0.28	0/2450	0.40	0/3301
1	B	0.28	0/2457	0.40	0/3310
1	C	0.26	0/2468	0.39	0/3325
1	D	0.28	0/2466	0.40	0/3322
1	E	0.27	0/2460	0.39	0/3313
1	F	0.26	0/2410	0.38	0/3247
All	All	0.27	0/14711	0.39	0/19818

There are no bond length outliers.

There are no bond angle outliers.

There are no chirality outliers.

There are no planarity outliers.

5.2 Too-close contacts (i)

In the following table, the Non-H and H(model) columns list the number of non-hydrogen atoms and hydrogen atoms in the chain respectively. The H(added) column lists the number of hydrogen atoms added and optimized by MolProbity. The Clashes column lists the number of clashes within the asymmetric unit, whereas Symm-Clashes lists symmetry-related clashes.

Mol	Chain	Non-H	H(model)	H(added)	Clashes	Symm-Clashes
1	A	2405	0	2437	114	0
1	B	2412	0	2446	110	0
1	C	2423	0	2458	105	0
1	D	2421	0	2454	124	0
1	E	2415	0	2448	137	0
1	F	2365	0	2390	150	0
2	A	1	0	0	0	0

Continued on next page...

For Manuscript Review

Continued from previous page...

Mol	Chain	Non-H	H(model)	H(added)	Clashes	Symm-Clashes
2	B	1	0	0	0	0
2	C	1	0	0	0	0
2	D	1	0	0	0	0
2	E	1	0	0	0	0
2	F	1	0	0	0	0
3	A	31	13	13	1	0
3	B	31	13	13	2	0
3	C	31	13	13	1	0
3	D	31	13	13	2	0
3	E	31	13	13	0	0
3	F	31	13	13	4	0
4	A	26	16	16	9	0
4	B	26	16	16	4	0
4	C	26	16	16	3	0
4	D	26	16	16	7	0
4	E	26	16	16	5	0
4	F	26	16	16	10	0
5	A	14	0	0	1	0
5	B	16	0	0	0	0
5	C	12	0	0	1	0
5	D	13	0	0	0	0
5	E	11	0	0	0	0
5	F	19	0	0	0	0
All	All	14874	174	14807	746	0

The all-atom clashscore is defined as the number of clashes found per 1000 atoms (including hydrogen atoms). The all-atom clashscore for this structure is 25.

All (746) close contacts within the same asymmetric unit are listed below, sorted by their clash magnitude.

Atom-1	Atom-2	Interatomic distance (Å)	Clash overlap (Å)
1:C:268:PRO:HB3	1:C:316:MET:HE3	1.28	1.09
1:D:191:MET:HE3	1:D:193:ARG:HG2	1.38	1.05
1:F:333:LEU:HD13	1:F:346:VAL:HG21	1.38	1.04
1:A:78:LEU:HD21	1:A:88:LYS:HB2	1.38	1.04
1:C:122:LEU:HD21	1:C:215:VAL:HG21	1.38	1.03
1:A:328:GLU:HG3	1:A:329:PRO:HD2	1.41	1.00
1:D:268:PRO:HG3	1:D:316:MET:HE2	1.39	1.00
1:D:157:GLN:HA	1:D:160:LYS:HE2	1.44	1.00
1:E:279:PHE:HE2	1:E:334:PRO:HG2	1.28	0.99
1:B:122:LEU:HD21	1:B:215:VAL:HG21	1.46	0.98

Continued on next page...

For Manuscript Review

Continued from previous page...

Atom-1	Atom-2	Interatomic distance (Å)	Clash overlap (Å)
1:D:122:LEU:HD21	1:D:215:VAL:HG11	1.43	0.98
1:F:78:LEU:HD11	1:F:88:LYS:HB2	1.43	0.97
1:F:103:ILE:HG21	1:F:223:MET:HE2	1.44	0.96
1:E:239:LEU:HD22	1:E:322:LEU:HD22	1.47	0.96
1:E:98:MET:HE2	1:E:146:CYS:HB3	1.45	0.95
1:D:98:MET:HE2	1:D:146:CYS:HB3	1.49	0.95
1:E:178:LEU:HD23	1:E:253:MET:HE1	1.47	0.93
1:E:243:HIS:HE2	1:E:354:PRO:HG2	1.34	0.91
1:A:122:LEU:HD21	1:A:215:VAL:HG21	1.54	0.87
1:F:238:ARG:HH11	1:F:244:TYR:HB3	1.39	0.87
1:D:94:SER:HB2	1:D:96:LEU:HD13	1.56	0.85
1:E:259:GLU:HB2	1:E:266:PRO:HD3	1.59	0.84
1:A:328:GLU:CG	1:A:329:PRO:HD2	2.07	0.84
1:A:339:THR:HG23	1:A:342:PHE:H	1.46	0.81
1:F:74:ARG:HH21	1:F:77:GLU:HB2	1.46	0.81
1:E:319:PHE:HB3	1:F:239:LEU:O	1.82	0.80
1:F:159:LEU:HD22	1:F:260:LEU:HD23	1.64	0.79
1:D:74:ARG:HA	1:D:89:VAL:HG12	1.65	0.78
1:F:88:LYS:HE3	1:F:97:ILE:HG21	1.65	0.78
1:E:279:PHE:CE2	1:E:334:PRO:HG2	2.18	0.78
1:F:103:ILE:CG2	1:F:223:MET:HE2	2.14	0.77
1:B:177:VAL:CG1	1:B:253:MET:HE2	2.14	0.77
1:B:191:MET:HG3	1:B:193:ARG:HG3	1.65	0.77
1:F:192:HIS:CD2	1:F:213:PHE:HB3	2.20	0.77
1:E:239:LEU:HD11	1:E:325:ILE:HD12	1.67	0.76
1:D:73:GLU:HB2	1:D:92:ARG:HE	1.50	0.76
1:B:78:LEU:HD21	1:B:88:LYS:HB2	1.68	0.76
1:F:318:ILE:HG22	1:F:321:LEU:HG	1.68	0.76
1:E:236:PRO:HB3	1:E:325:ILE:HG22	1.67	0.75
1:E:279:PHE:CD2	1:E:337:VAL:HG21	2.20	0.75
1:A:265:TYR:CE2	1:A:267:ILE:HB	2.22	0.75
1:D:139:SER:O	1:D:142:GLU:HG2	1.87	0.75
1:B:328:GLU:HB3	1:B:329:PRO:HD2	1.69	0.74
1:D:45:GLU:HG2	1:D:46:LEU:H	1.52	0.74
1:D:98:MET:CE	1:D:146:CYS:HB3	2.17	0.74
1:E:167:GLU:OE2	1:E:339:THR:HG22	1.87	0.74
1:F:107:ILE:CG2	1:F:111:ILE:HB	2.17	0.74
1:F:333:LEU:CD1	1:F:346:VAL:HG21	2.14	0.74
1:F:335:ASN:HA	1:F:343:GLN:HE22	1.53	0.74
1:F:335:ASN:HA	1:F:343:GLN:NE2	2.02	0.74
1:A:185:ARG:HH12	1:A:360:LEU:HD12	1.52	0.74

Continued on next page...

For Manuscript Review

Continued from previous page...

Atom-1	Atom-2	Interatomic distance (Å)	Clash overlap (Å)
1:D:98:MET:HE3	1:D:134:TYR:CG	2.22	0.73
1:F:185:ARG:HE	1:F:246:VAL:HG21	1.53	0.73
1:B:167:GLU:OE2	1:B:342:PHE:HB2	1.89	0.73
1:C:269:PRO:HG2	1:C:324:TYR:OH	1.87	0.73
1:F:265:TYR:CE2	1:F:267:ILE:HB	2.24	0.73
1:F:251:TRP:HB2	1:F:357:ARG:HH11	1.52	0.72
1:D:114:GLN:O	1:D:118:GLU:HG3	1.89	0.72
1:D:191:MET:HE3	1:D:193:ARG:CG	2.17	0.72
1:A:333:LEU:HD11	1:A:346:VAL:HG11	1.71	0.72
1:D:267:ILE:HG21	1:D:321:LEU:HD12	1.72	0.72
1:B:219:LEU:HG	1:B:223:MET:HE2	1.73	0.71
1:C:107:ILE:HG22	1:C:111:ILE:HB	1.73	0.71
1:B:100:ARG:HE	1:B:102:LEU:HD21	1.55	0.71
1:E:328:GLU:HB3	1:E:329:PRO:HD2	1.73	0.71
1:B:265:TYR:CE2	1:B:267:ILE:HB	2.26	0.70
1:E:78:LEU:HD21	1:E:88:LYS:HB2	1.73	0.70
1:A:58:LEU:HD21	1:A:126:ASN:ND2	2.07	0.70
1:B:192:HIS:CD2	1:B:213:PHE:HB3	2.26	0.70
1:A:108:LYS:HE3	1:A:109:PRO:HD2	1.72	0.70
1:C:274:GLU:O	1:C:278:ILE:HG13	1.92	0.70
1:E:168:GLU:HB3	1:E:377:VAL:HG21	1.73	0.70
1:F:78:LEU:CD1	1:F:88:LYS:HB2	2.22	0.70
1:D:165:ILE:HD12	1:D:260:LEU:HB3	1.74	0.70
1:A:238:ARG:HG2	1:A:244:TYR:CD1	2.27	0.69
1:D:369:ILE:O	1:D:373:GLU:HG3	1.90	0.69
1:C:114:GLN:O	1:C:118:GLU:HG3	1.91	0.69
1:E:159:LEU:HD22	1:E:260:LEU:HD23	1.72	0.69
1:B:234:MET:CE	1:B:238:ARG:HB3	2.22	0.69
1:C:138:TYR:OH	1:C:141:GLY:HA2	1.92	0.69
1:B:184:LEU:HD22	1:B:190:ILE:HD11	1.73	0.69
1:F:267:ILE:HD12	1:F:325:ILE:HG12	1.74	0.69
1:D:46:LEU:HD11	1:D:128:PRO:HG3	1.75	0.69
1:D:60:GLN:HB3	1:D:96:LEU:HD21	1.73	0.69
1:E:239:LEU:CD2	1:E:322:LEU:HD22	2.19	0.69
1:E:178:LEU:CD2	1:E:253:MET:HE1	2.20	0.68
1:C:261:ALA:HB1	1:C:338:PHE:CZ	2.28	0.68
1:F:101:LYS:HE2	3:F:402:ANP:O1A	1.92	0.68
1:D:122:LEU:HD13	4:D:403:5BM:H12	1.76	0.68
1:F:103:ILE:HG22	1:F:105:LEU:HG	1.76	0.68
1:C:58:LEU:HD21	1:C:126:ASN:ND2	2.09	0.67
1:C:46:LEU:HD22	1:C:128:PRO:HG3	1.76	0.67

Continued on next page...

For Manuscript Review

Continued from previous page...

Atom-1	Atom-2	Interatomic distance (Å)	Clash overlap (Å)
1:B:265:TYR:HE2	1:B:321:LEU:HD11	1.58	0.67
1:E:351:ILE:HG21	1:E:356:GLU:HB2	1.75	0.67
1:E:317:ALA:HB2	1:F:241:GLY:HA3	1.76	0.67
1:C:107:ILE:CG2	1:C:111:ILE:HB	2.23	0.67
1:E:98:MET:CE	1:E:146:CYS:HB3	2.20	0.67
1:A:122:LEU:HB2	1:A:133:PHE:CD2	2.30	0.67
1:B:177:VAL:HG12	1:B:253:MET:HE2	1.77	0.66
1:F:107:ILE:HG22	1:F:111:ILE:HB	1.75	0.66
1:F:195:VAL:HB	1:F:252:SER:HB2	1.75	0.66
1:C:131:VAL:HG23	1:C:210:LEU:O	1.95	0.66
1:D:268:PRO:CG	1:D:316:MET:HE2	2.21	0.66
1:F:267:ILE:O	1:F:269:PRO:HD3	1.95	0.66
1:F:354:PRO:HA	1:F:357:ARG:HE	1.61	0.66
1:E:71:ASP:O	1:E:92:ARG:HB2	1.96	0.66
1:B:173:VAL:O	1:B:177:VAL:HG23	1.95	0.66
1:E:114:GLN:O	1:E:118:GLU:HG3	1.96	0.66
1:E:116:ILE:O	1:E:120:GLN:HG2	1.95	0.66
1:F:321:LEU:O	1:F:325:ILE:HG13	1.95	0.66
1:E:324:TYR:HA	1:E:328:GLU:CG	2.25	0.66
1:F:74:ARG:NH2	1:F:77:GLU:HB2	2.11	0.65
1:B:369:ILE:O	1:B:373:GLU:HG3	1.97	0.65
1:D:138:TYR:OH	1:D:141:GLY:HA2	1.97	0.65
1:F:354:PRO:HB3	1:F:357:ARG:HH21	1.61	0.65
1:A:247:GLN:HB3	1:A:357:ARG:HH11	1.60	0.65
1:E:239:LEU:HD13	1:E:322:LEU:HA	1.78	0.65
1:E:276:GLU:HG3	1:E:281:ARG:C	2.22	0.65
1:A:196:LYS:HE2	1:A:198:SER:HB3	1.77	0.65
1:C:268:PRO:CB	1:C:316:MET:HE3	2.17	0.65
1:F:100:ARG:NE	1:F:102:LEU:HD21	2.11	0.65
1:B:264:ARG:HH21	1:B:270:PRO:HA	1.61	0.65
1:E:50:GLN:O	1:E:54:LEU:HD23	1.97	0.64
1:D:191:MET:HE1	1:D:244:TYR:CE1	2.32	0.64
1:D:267:ILE:CG2	1:D:321:LEU:HD12	2.27	0.64
4:E:403:5BM:C04	4:E:403:5BM:HN13	2.10	0.64
1:C:192:HIS:CD2	1:C:213:PHE:HB3	2.32	0.64
1:D:265:TYR:HE2	1:D:321:LEU:HD11	1.62	0.64
1:E:318:ILE:O	1:E:322:LEU:HG	1.97	0.64
1:F:324:TYR:CE2	1:F:328:GLU:HG3	2.33	0.64
1:B:48:GLU:O	1:B:52:LYS:HG2	1.97	0.64
1:F:318:ILE:CG2	1:F:321:LEU:HG	2.28	0.64
1:E:92:ARG:HB3	1:E:93:PRO:HD3	1.78	0.64

Continued on next page...

For Manuscript Review

Continued from previous page...

Atom-1	Atom-2	Interatomic distance (Å)	Clash overlap (Å)
1:F:274:GLU:O	1:F:278:ILE:HG13	1.98	0.64
1:D:98:MET:HE3	1:D:134:TYR:CD2	2.33	0.64
1:E:54:LEU:O	1:E:58:LEU:HG	1.98	0.64
1:B:330:PRO:HD3	1:B:352:LYS:NZ	2.13	0.63
1:E:243:HIS:NE2	1:E:354:PRO:HG2	2.09	0.63
1:A:114:GLN:O	1:A:118:GLU:HG3	1.97	0.63
1:F:318:ILE:HG23	1:F:320:GLU:HG2	1.80	0.63
1:B:138:TYR:OH	1:B:141:GLY:HA2	1.99	0.63
1:A:195:VAL:HB	1:A:252:SER:HB2	1.81	0.63
1:C:78:LEU:HD21	1:C:88:LYS:HB2	1.79	0.63
1:E:261:ALA:HB1	1:E:338:PHE:CZ	2.34	0.63
1:F:45:GLU:HG3	1:F:46:LEU:HD12	1.81	0.63
1:A:185:ARG:NH1	1:A:360:LEU:HD12	2.14	0.62
1:D:98:MET:HE3	1:D:134:TYR:CB	2.28	0.62
1:B:273:LYS:HD3	1:B:273:LYS:C	2.24	0.62
1:A:279:PHE:CE1	1:A:337:VAL:HG11	2.34	0.62
1:B:237:GLU:HG3	1:B:243:HIS:HB2	1.81	0.62
1:D:78:LEU:HD21	1:D:88:LYS:HB2	1.81	0.62
1:B:159:LEU:HD22	1:B:260:LEU:HD23	1.80	0.62
1:E:177:VAL:CG1	1:E:253:MET:HE2	2.30	0.62
1:F:105:LEU:HD13	1:F:222:SER:HB3	1.82	0.62
1:C:164:ARG:HD3	1:C:278:ILE:HG22	1.82	0.62
1:A:159:LEU:HD22	1:A:260:LEU:HD23	1.82	0.62
1:A:274:GLU:O	1:A:278:ILE:HG13	2.00	0.62
1:E:191:MET:HG2	1:E:246:VAL:HG22	1.80	0.62
1:B:237:GLU:HB2	1:B:243:HIS:CG	2.35	0.61
1:D:45:GLU:HG2	1:D:46:LEU:N	2.14	0.61
1:F:242:THR:HG22	1:F:243:HIS:ND1	2.15	0.61
1:E:51:LYS:O	1:E:55:GLU:HG3	2.00	0.61
1:D:46:LEU:O	1:D:51:LYS:HE3	2.01	0.61
1:E:265:TYR:CD1	1:E:266:PRO:HD2	2.36	0.61
1:E:328:GLU:O	1:E:352:LYS:HD3	2.01	0.61
1:F:100:ARG:HE	1:F:102:LEU:HD21	1.66	0.60
1:C:167:GLU:HG3	1:C:342:PHE:CD2	2.35	0.60
1:C:237:GLU:HG3	1:C:243:HIS:HB2	1.83	0.60
1:D:88:LYS:HE3	1:D:97:ILE:HG21	1.83	0.60
1:B:178:LEU:HD23	1:B:253:MET:HE1	1.83	0.60
1:F:111:ILE:O	1:F:115:ILE:HG13	2.01	0.60
1:F:185:ARG:HH21	1:F:246:VAL:HG23	1.67	0.60
1:E:98:MET:HE3	1:E:134:TYR:CG	2.37	0.60
1:E:335:ASN:HA	1:E:343:GLN:OE1	2.01	0.60

Continued on next page...

For Manuscript Review

Continued from previous page...

Atom-1	Atom-2	Interatomic distance (Å)	Clash overlap (Å)
1:D:94:SER:CB	1:D:96:LEU:HD13	2.31	0.59
1:A:212:ASP:H	4:A:403:5BM:C11	2.14	0.59
1:F:78:LEU:HD13	1:F:88:LYS:N	2.17	0.59
1:E:351:ILE:HG21	1:E:356:GLU:CB	2.32	0.59
1:C:128:PRO:HG2	1:C:129:TYR:CE2	2.37	0.59
1:F:344:GLU:O	1:F:348:LYS:HG2	2.03	0.59
1:A:265:TYR:HE2	1:A:267:ILE:HB	1.66	0.59
1:C:264:ARG:HH21	1:C:270:PRO:HA	1.67	0.59
1:C:385:LYS:O	1:C:388:ARG:HD2	2.03	0.59
1:E:251:TRP:CZ2	1:E:325:ILE:HG23	2.37	0.59
1:B:344:GLU:HG2	1:B:348:LYS:HE2	1.85	0.59
1:D:91:HIS:HB3	1:D:94:SER:OG	2.03	0.59
1:F:332:LYS:HD2	1:F:347:ASN:OD1	2.02	0.59
1:E:138:TYR:OH	1:E:141:GLY:HA2	2.03	0.58
1:F:324:TYR:CZ	1:F:328:GLU:HG3	2.37	0.58
1:C:192:HIS:O	1:C:193:ARG:HB2	2.04	0.58
1:A:91:HIS:HD2	1:A:94:SER:H	1.48	0.58
1:A:188:HIS:O	1:A:190:ILE:HG13	2.03	0.58
1:A:324:TYR:CD2	1:A:328:GLU:HG2	2.37	0.58
1:C:268:PRO:HB3	1:C:316:MET:CE	2.19	0.58
1:F:71:ASP:O	1:F:92:ARG:HG3	2.04	0.58
1:A:138:TYR:OH	1:A:141:GLY:HA2	2.03	0.58
1:B:219:LEU:HD23	4:B:403:5BM:N03	2.18	0.58
1:B:335:ASN:HA	1:B:343:GLN:HE22	1.68	0.58
1:F:318:ILE:CG2	1:F:320:GLU:HG2	2.32	0.58
1:C:49:GLN:HA	1:C:49:GLN:HE21	1.67	0.58
1:C:91:HIS:HB3	1:C:94:SER:OG	2.03	0.58
1:C:111:ILE:HG23	1:C:218:GLN:CG	2.34	0.57
1:E:154:SER:HB2	1:E:198:SER:HA	1.86	0.57
1:C:172:LYS:HD3	1:C:379:PHE:HB2	1.87	0.57
1:D:265:TYR:CE2	1:D:267:ILE:HB	2.39	0.57
1:E:206:GLY:HA2	1:E:379:PHE:HD2	1.68	0.57
1:D:159:LEU:HD22	1:D:260:LEU:HD23	1.86	0.57
1:F:259:GLU:HB2	1:F:266:PRO:HD3	1.86	0.57
1:E:219:LEU:O	1:E:223:MET:HG2	2.03	0.57
1:A:164:ARG:HD3	1:A:278:ILE:HG22	1.87	0.57
1:D:46:LEU:CD1	1:D:128:PRO:HG3	2.33	0.57
1:D:78:LEU:HD13	3:D:402:ANP:C2	2.34	0.57
1:C:219:LEU:CD1	1:C:223:MET:HE2	2.34	0.57
1:E:61:LYS:HE3	1:E:133:PHE:O	2.05	0.57
1:A:359:ASP:OD2	1:A:361:LYS:HG2	2.04	0.57

Continued on next page...

For Manuscript Review

Continued from previous page...

Atom-1	Atom-2	Interatomic distance (Å)	Clash overlap (Å)
1:E:188:HIS:O	1:E:190:ILE:HG23	2.05	0.57
1:A:239:LEU:O	1:D:319:PHE:HB3	2.03	0.57
1:F:119:LEU:HD23	1:F:215:VAL:CG1	2.34	0.57
1:A:111:ILE:O	1:A:115:ILE:HG13	2.05	0.57
1:E:167:GLU:OE2	1:E:339:THR:CG2	2.52	0.57
1:E:175:ILE:HD11	1:E:372:SER:HB2	1.86	0.57
1:E:177:VAL:HG12	1:E:253:MET:HE2	1.87	0.57
1:B:223:MET:HE3	4:B:403:5BM:C31	2.35	0.56
1:E:168:GLU:HB3	1:E:377:VAL:CG2	2.35	0.56
1:F:344:GLU:HG3	1:F:348:LYS:NZ	2.19	0.56
1:D:267:ILE:HG22	1:D:316:MET:HE1	1.86	0.56
1:F:78:LEU:HD13	1:F:88:LYS:H	1.69	0.56
1:A:333:LEU:N	1:A:333:LEU:HD12	2.20	0.56
1:B:331:PRO:HD2	1:B:350:LEU:HD13	1.87	0.56
1:E:322:LEU:O	1:E:326:VAL:HG23	2.05	0.56
1:A:216:SER:O	1:A:220:ILE:HD13	2.06	0.56
1:E:233:TYR:HA	1:E:255:LEU:HD23	1.88	0.56
1:B:185:ARG:HD3	1:B:246:VAL:HG21	1.88	0.56
1:B:240:GLN:O	1:B:240:GLN:HG3	2.05	0.56
1:C:359:ASP:OD1	1:C:360:LEU:N	2.37	0.56
1:D:103:ILE:HD11	4:D:403:5BM:N23	2.19	0.56
1:E:366:HIS:O	1:E:370:LYS:HG2	2.06	0.56
1:E:185:ARG:HH21	1:E:246:VAL:CG2	2.18	0.56
1:D:358:ALA:CB	1:D:362:MET:HE3	2.36	0.56
1:E:98:MET:HE3	1:E:134:TYR:CD2	2.41	0.56
1:A:48:GLU:O	1:A:52:LYS:HG3	2.06	0.56
1:A:371:ARG:O	1:A:375:GLU:HG2	2.07	0.55
1:D:98:MET:HE3	1:D:134:TYR:HB2	1.88	0.55
1:D:164:ARG:HD2	1:D:278:ILE:HG22	1.88	0.55
1:D:264:ARG:HH21	1:D:270:PRO:HA	1.71	0.55
1:F:110:ALA:O	1:F:114:GLN:HG3	2.07	0.55
1:E:74:ARG:HA	1:E:89:VAL:HG12	1.88	0.55
1:E:178:LEU:HD22	1:E:360:LEU:HD22	1.87	0.55
1:C:54:LEU:O	1:C:58:LEU:HG	2.06	0.55
1:A:214:GLY:HA2	4:A:403:5BM:N25	2.22	0.55
1:E:60:GLN:HB3	1:E:96:LEU:CD1	2.37	0.55
1:F:251:TRP:HB2	1:F:357:ARG:NH1	2.20	0.55
1:C:165:ILE:HD12	1:C:260:LEU:HD22	1.87	0.55
1:C:178:LEU:HD21	1:C:253:MET:HE1	1.89	0.55
1:F:331:PRO:O	1:F:332:LYS:HD3	2.07	0.55
1:C:191:MET:HG3	1:C:193:ARG:HG3	1.87	0.55

Continued on next page...

For Manuscript Review

Continued from previous page...

Atom-1	Atom-2	Interatomic distance (Å)	Clash overlap (Å)
1:C:234:MET:HE3	1:C:239:LEU:CD2	2.36	0.55
1:B:114:GLN:O	1:B:118:GLU:HG3	2.07	0.55
1:E:169:ILE:HD13	1:E:377:VAL:HG11	1.88	0.55
1:A:60:GLN:OE1	1:A:60:GLN:HA	2.07	0.55
1:B:100:ARG:NE	1:B:102:LEU:HD21	2.20	0.55
1:C:74:ARG:HA	1:C:89:VAL:HG12	1.88	0.55
1:C:219:LEU:HD11	1:C:223:MET:HE2	1.89	0.55
1:E:317:ALA:CB	1:F:241:GLY:HA3	2.36	0.55
1:B:274:GLU:O	1:B:278:ILE:HG13	2.06	0.54
4:C:403:5BM:C22	4:C:403:5BM:S06	2.95	0.54
1:D:191:MET:HE1	1:D:244:TYR:HE1	1.71	0.54
1:A:48:GLU:HA	1:A:51:LYS:HE2	1.89	0.54
1:C:164:ARG:HD3	1:C:278:ILE:CG2	2.37	0.54
1:D:188:HIS:O	1:D:190:ILE:HG13	2.07	0.54
1:F:49:GLN:O	1:F:53:ARG:HG3	2.07	0.54
1:E:100:ARG:HD2	1:E:144:SER:HB3	1.89	0.54
1:F:238:ARG:HH11	1:F:244:TYR:CB	2.16	0.54
1:A:318:ILE:O	1:A:322:LEU:HD13	2.07	0.54
1:D:167:GLU:OE2	1:D:342:PHE:HB2	2.07	0.54
1:D:187:LYS:HG3	1:D:188:HIS:CE1	2.43	0.54
1:E:247:GLN:HB3	1:E:357:ARG:HE	1.72	0.54
1:D:116:ILE:O	1:D:120:GLN:HG2	2.06	0.54
1:A:152:GLY:HA3	1:A:202:VAL:HG23	1.90	0.54
1:B:116:ILE:O	1:B:120:GLN:HG2	2.08	0.54
1:E:138:TYR:HD2	1:E:143:ILE:HG12	1.72	0.54
1:E:171:GLY:O	1:E:175:ILE:HG13	2.08	0.54
1:F:334:PRO:HD2	1:F:338:PHE:CE2	2.43	0.54
1:C:133:PHE:CE2	1:C:135:GLY:HA2	2.43	0.54
1:D:164:ARG:HD2	1:D:278:ILE:CG2	2.38	0.54
1:D:192:HIS:CD2	1:D:213:PHE:HB3	2.43	0.54
1:E:324:TYR:HA	1:E:328:GLU:HG2	1.88	0.54
1:D:105:LEU:HD12	1:D:143:ILE:HD12	1.90	0.54
1:F:128:PRO:HG2	1:F:129:TYR:CD2	2.43	0.54
1:F:177:VAL:O	1:F:181:LEU:HG	2.08	0.54
1:F:279:PHE:CE1	1:F:337:VAL:HG11	2.43	0.54
1:F:78:LEU:HD23	3:F:402:ANP:C2	2.38	0.54
1:C:49:GLN:HA	1:C:49:GLN:NE2	2.23	0.53
1:C:128:PRO:HG2	1:C:129:TYR:CD2	2.43	0.53
1:D:122:LEU:CD2	1:D:215:VAL:HG11	2.28	0.53
1:E:184:LEU:HD22	1:E:190:ILE:HD11	1.88	0.53
1:D:128:PRO:HG2	1:D:129:TYR:CD2	2.43	0.53

Continued on next page...

For Manuscript Review

Continued from previous page...

Atom-1	Atom-2	Interatomic distance (Å)	Clash overlap (Å)
1:E:270:PRO:HB2	1:E:275:LEU:HD21	1.90	0.53
1:A:164:ARG:HD3	1:A:278:ILE:CG2	2.38	0.53
1:A:261:ALA:HB1	1:A:338:PHE:CZ	2.43	0.53
1:B:86:VAL:HG21	3:B:402:ANP:H5'1	1.90	0.53
1:D:58:LEU:HD21	1:D:126:ASN:ND2	2.23	0.53
1:D:234:MET:HG2	1:D:238:ARG:HB3	1.90	0.53
1:D:165:ILE:HD12	1:D:260:LEU:HD22	1.91	0.53
1:B:61:LYS:HE3	1:B:134:TYR:HA	1.88	0.53
1:F:147:MET:HE2	4:F:403:5BM:C32	2.39	0.53
1:F:103:ILE:HD13	1:F:223:MET:CE	2.39	0.53
1:F:215:VAL:HG12	4:F:403:5BM:HN25	1.73	0.53
1:F:351:ILE:HG21	1:F:356:GLU:HB2	1.90	0.53
1:C:103:ILE:HG21	1:C:223:MET:SD	2.49	0.53
1:D:79:GLY:HA3	3:D:402:ANP:H4'	1.89	0.53
1:B:188:HIS:O	1:B:190:ILE:HG23	2.10	0.52
1:B:235:ALA:HA	1:B:251:TRP:CD1	2.45	0.52
1:E:243:HIS:HE2	1:E:354:PRO:CG	2.15	0.52
1:B:261:ALA:HB1	1:B:338:PHE:CZ	2.45	0.52
1:E:324:TYR:O	1:E:328:GLU:HB2	2.08	0.52
1:F:50:GLN:HG2	1:F:53:ARG:NH2	2.24	0.52
1:D:59:THR:O	1:D:63:LYS:HD3	2.09	0.52
1:F:258:VAL:HG22	1:F:333:LEU:HD11	1.90	0.52
1:F:53:ARG:HD2	1:F:205:ARG:CD	2.40	0.52
1:F:145:ILE:HG21	4:F:403:5BM:H32	1.91	0.52
1:F:220:ILE:HA	4:F:403:5BM:C11	2.39	0.52
1:A:197:PRO:HG3	1:A:256:SER:HB3	1.92	0.52
1:C:46:LEU:CD2	1:C:128:PRO:HG3	2.39	0.52
1:D:318:ILE:O	1:D:322:LEU:HG	2.10	0.52
1:D:165:ILE:CD1	1:D:260:LEU:HD22	2.40	0.52
1:D:51:LYS:O	1:D:55:GLU:HG2	2.10	0.52
1:D:169:ILE:HG23	1:D:379:PHE:HD1	1.74	0.52
1:F:138:TYR:HD2	1:F:143:ILE:HG12	1.74	0.52
1:B:215:VAL:HB	4:B:403:5BM:HN05	1.75	0.51
1:B:335:ASN:HA	1:B:343:GLN:NE2	2.24	0.51
1:D:358:ALA:HA	1:D:362:MET:CE	2.40	0.51
1:A:54:LEU:O	1:A:58:LEU:HG	2.10	0.51
1:A:220:ILE:N	1:A:220:ILE:HD12	2.25	0.51
1:B:138:TYR:HD2	1:B:143:ILE:HG12	1.75	0.51
1:D:191:MET:CE	1:D:193:ARG:HG2	2.26	0.51
4:D:403:5BM:N13	4:D:403:5BM:N05	2.56	0.51
1:C:111:ILE:O	1:C:115:ILE:HG13	2.10	0.51

Continued on next page...

For Manuscript Review

Continued from previous page...

Atom-1	Atom-2	Interatomic distance (Å)	Clash overlap (Å)
1:E:162:ALA:HB2	1:E:386:THR:HG23	1.92	0.51
1:F:164:ARG:HD3	1:F:278:ILE:CG2	2.41	0.51
1:F:183:TYR:CE1	1:F:187:LYS:HG3	2.46	0.51
1:D:173:VAL:HG22	1:D:208:ILE:HD13	1.92	0.51
1:F:375:GLU:HG3	1:F:375:GLU:O	2.11	0.51
1:E:98:MET:HE3	1:E:134:TYR:CB	2.41	0.51
1:F:61:LYS:HE3	1:F:133:PHE:O	2.10	0.51
1:C:264:ARG:HH21	1:C:270:PRO:CA	2.24	0.51
1:F:371:ARG:O	1:F:375:GLU:HG2	2.10	0.51
1:D:62:ALA:HA	1:E:117:ARG:NH2	2.25	0.51
1:F:220:ILE:HA	4:F:403:5BM:H11	1.92	0.51
1:C:152:GLY:HA3	1:C:202:VAL:HG23	1.92	0.51
1:D:98:MET:CE	1:D:134:TYR:HB2	2.41	0.51
1:D:274:GLU:O	1:D:278:ILE:HG13	2.11	0.51
1:E:371:ARG:O	1:E:375:GLU:HG2	2.11	0.50
1:F:238:ARG:HG3	1:F:248:SER:OG	2.11	0.50
1:B:269:PRO:HG3	1:B:324:TYR:OH	2.11	0.50
1:C:50:GLN:O	1:C:54:LEU:HD13	2.11	0.50
1:C:64:VAL:CG1	1:C:67:LEU:HD21	2.42	0.50
1:D:169:ILE:HD13	1:D:377:VAL:HG11	1.93	0.50
1:E:236:PRO:CB	1:E:325:ILE:HG22	2.38	0.50
1:B:216:SER:O	1:B:220:ILE:HG13	2.12	0.50
1:D:128:PRO:HG2	1:D:129:TYR:CE2	2.46	0.50
1:E:77:GLU:HA	1:E:87:THR:HG22	1.93	0.50
1:E:185:ARG:HE	1:E:246:VAL:HG21	1.77	0.50
1:E:239:LEU:HD13	1:E:322:LEU:HD22	1.92	0.50
1:F:114:GLN:O	1:F:118:GLU:HG3	2.10	0.50
1:A:326:VAL:HG12	1:A:327:ASN:OD1	2.11	0.50
1:A:333:LEU:HD11	1:A:346:VAL:HG21	1.94	0.50
1:C:100:ARG:NE	1:C:102:LEU:HD21	2.26	0.50
1:C:330:PRO:HD3	1:C:352:LYS:NZ	2.26	0.50
1:D:112:ARG:O	1:D:116:ILE:HG12	2.12	0.50
1:D:115:ILE:O	1:D:119:LEU:HG	2.12	0.50
1:E:317:ALA:HB3	1:E:320:GLU:HB3	1.93	0.50
1:A:91:HIS:CD2	1:A:94:SER:H	2.28	0.50
1:A:328:GLU:HG3	1:A:329:PRO:CD	2.27	0.50
1:B:334:PRO:HD2	1:B:338:PHE:CE2	2.47	0.50
1:C:92:ARG:HB2	1:C:93:PRO:HD3	1.94	0.50
1:D:255:LEU:N	1:D:350:LEU:HD21	2.27	0.50
1:F:130:ILE:HG23	1:F:213:PHE:CE2	2.47	0.50
1:F:330:PRO:HD3	1:F:352:LYS:HE3	1.94	0.50

Continued on next page...

For Manuscript Review

Continued from previous page...

Atom-1	Atom-2	Interatomic distance (Å)	Clash overlap (Å)
1:C:104:HIS:O	1:C:105:LEU:HD23	2.12	0.50
1:D:64:VAL:CG1	1:D:67:LEU:HD21	2.40	0.50
1:F:92:ARG:HB2	1:F:93:PRO:HD3	1.93	0.50
1:C:361:LYS:HG3	1:C:362:MET:N	2.26	0.50
1:E:115:ILE:O	1:E:119:LEU:HG	2.12	0.50
4:E:403:5BM:S26	4:E:403:5BM:C02	3.00	0.50
1:A:165:ILE:HD12	1:A:260:LEU:HD22	1.94	0.50
1:A:272:ALA:O	1:A:276:GLU:HG3	2.11	0.50
1:E:152:GLY:HA3	1:E:202:VAL:HG23	1.93	0.50
1:B:191:MET:HG3	1:B:193:ARG:CG	2.40	0.49
1:A:129:TYR:O	1:A:130:ILE:HD13	2.11	0.49
1:B:162:ALA:HB2	1:B:386:THR:HG23	1.94	0.49
1:E:240:GLN:OE1	1:E:326:VAL:HG13	2.12	0.49
1:D:162:ALA:HB2	1:D:386:THR:HG23	1.95	0.49
1:F:265:TYR:OH	1:F:267:ILE:HG13	2.12	0.49
1:A:214:GLY:HA2	4:A:403:5BM:HN25	1.75	0.49
1:A:339:THR:CG2	1:A:342:PHE:H	2.20	0.49
4:A:403:5BM:S26	4:A:403:5BM:C02	3.00	0.49
1:B:267:ILE:HD12	1:B:325:ILE:HG12	1.94	0.49
1:C:324:TYR:HA	1:C:328:GLU:CG	2.42	0.49
1:D:47:ASP:HB2	1:D:50:GLN:HG3	1.94	0.49
1:F:270:PRO:HA	1:F:274:GLU:OE1	2.13	0.49
1:B:73:GLU:HB2	1:B:92:ARG:HE	1.78	0.49
1:E:48:GLU:HA	1:E:51:LYS:HZ3	1.78	0.49
1:F:175:ILE:HG12	1:F:369:ILE:HG23	1.94	0.49
1:C:138:TYR:HD2	1:C:143:ILE:CD1	2.25	0.49
1:C:167:GLU:OE1	1:C:339:THR:HG23	2.12	0.49
1:F:219:LEU:HD11	1:F:223:MET:HE3	1.95	0.49
1:B:206:GLY:HA2	1:B:379:PHE:HD2	1.77	0.49
1:C:234:MET:HE3	1:C:239:LEU:HD21	1.95	0.49
1:C:167:GLU:OE2	1:C:342:PHE:HB2	2.13	0.49
1:D:67:LEU:HD12	1:D:135:GLY:HA3	1.95	0.49
1:D:328:GLU:OE1	1:D:328:GLU:HA	2.12	0.49
1:B:202:VAL:HG11	1:B:379:PHE:HZ	1.76	0.48
1:D:279:PHE:CD2	1:D:337:VAL:HG11	2.48	0.48
1:F:147:MET:HB3	4:F:403:5BM:H31	1.94	0.48
1:A:211:CYS:HB2	4:A:403:5BM:C11	2.43	0.48
1:B:271:ASP:O	1:B:275:LEU:HG	2.14	0.48
1:A:78:LEU:CD2	1:A:88:LYS:HB2	2.26	0.48
1:B:173:VAL:HG22	1:B:208:ILE:HD13	1.95	0.48
1:B:371:ARG:HG2	1:B:371:ARG:HH11	1.78	0.48

Continued on next page...

For Manuscript Review

Continued from previous page...

Atom-1	Atom-2	Interatomic distance (Å)	Clash overlap (Å)
1:F:154:SER:HB2	1:F:157:GLN:HG3	1.95	0.48
1:A:376:GLU:O	1:A:376:GLU:HG3	2.14	0.48
1:B:273:LYS:HD3	1:B:273:LYS:O	2.14	0.48
1:A:238:ARG:HA	1:A:244:TYR:CE1	2.49	0.48
1:C:165:ILE:HD12	1:C:260:LEU:HB3	1.96	0.48
1:F:152:GLY:HA3	1:F:202:VAL:HG23	1.96	0.48
1:A:211:CYS:HB2	4:A:403:5BM:H11	1.96	0.48
1:A:234:MET:HE3	1:A:238:ARG:CZ	2.43	0.48
1:A:324:TYR:CE2	1:A:328:GLU:HG2	2.48	0.48
1:A:328:GLU:CD	1:A:329:PRO:HD2	2.37	0.48
1:E:215:VAL:HG12	4:E:403:5BM:HN05	1.79	0.48
1:D:122:LEU:CD1	4:D:403:5BM:H12	2.43	0.48
1:E:133:PHE:HE1	1:E:145:ILE:HG23	1.78	0.48
1:E:239:LEU:HD13	1:E:322:LEU:CD2	2.44	0.47
1:F:78:LEU:HD23	3:F:402:ANP:N3	2.29	0.47
1:F:195:VAL:HB	1:F:252:SER:CB	2.40	0.47
1:B:270:PRO:HB2	1:B:275:LEU:HD21	1.95	0.47
1:F:164:ARG:HD3	1:F:278:ILE:HG22	1.96	0.47
1:F:48:GLU:HA	1:F:51:LYS:HE2	1.96	0.47
1:B:359:ASP:OD1	1:B:362:MET:HE3	2.15	0.47
1:B:370:LYS:O	1:B:374:VAL:HG23	2.14	0.47
1:C:279:PHE:CE1	1:C:337:VAL:HG11	2.49	0.47
1:A:258:VAL:O	1:A:262:VAL:HG22	2.15	0.47
1:C:378:ASP:OD2	1:C:381:GLY:HA3	2.14	0.47
1:F:54:LEU:O	1:F:58:LEU:HG	2.14	0.47
1:E:339:THR:HG23	1:E:342:PHE:H	1.80	0.47
1:F:173:VAL:HG22	1:F:208:ILE:HD13	1.96	0.47
1:F:264:ARG:HD3	1:F:274:GLU:OE2	2.14	0.47
1:A:237:GLU:HB2	1:A:243:HIS:ND1	2.29	0.47
4:A:403:5BM:H12	4:A:403:5BM:N05	2.28	0.47
1:B:54:LEU:O	1:B:58:LEU:HG	2.14	0.47
1:B:344:GLU:CG	1:B:348:LYS:HE2	2.45	0.47
1:C:255:LEU:HD22	1:C:266:PRO:HG2	1.95	0.47
1:D:265:TYR:CE2	1:D:321:LEU:HD11	2.47	0.47
1:F:185:ARG:HH21	1:F:246:VAL:CG2	2.27	0.47
1:F:242:THR:HG22	1:F:243:HIS:CE1	2.49	0.47
1:F:253:MET:O	1:F:254:GLY:C	2.57	0.47
1:F:329:PRO:HA	1:F:352:LYS:HE3	1.95	0.47
1:A:61:LYS:HE3	1:A:133:PHE:O	2.15	0.47
1:E:171:GLY:HA2	1:E:368:PHE:CE2	2.50	0.47
1:C:206:GLY:HA2	1:C:379:PHE:HD2	1.79	0.47

Continued on next page...

For Manuscript Review

Continued from previous page...

Atom-1	Atom-2	Interatomic distance (Å)	Clash overlap (Å)
1:C:322:LEU:O	1:C:326:VAL:HG23	2.15	0.47
1:D:46:LEU:HD23	1:D:51:LYS:HG2	1.96	0.47
1:D:191:MET:HE1	1:D:244:TYR:CD1	2.50	0.47
1:F:150:MET:HG3	1:F:201:LEU:HB3	1.96	0.47
1:B:234:MET:HE2	1:B:238:ARG:HB3	1.97	0.47
1:B:255:LEU:HD11	1:B:266:PRO:HG2	1.97	0.47
1:C:107:ILE:HG21	1:C:111:ILE:CG2	2.45	0.47
1:E:67:LEU:HD22	1:E:72:PHE:HZ	1.80	0.47
1:E:109:PRO:HA	1:E:112:ARG:NE	2.29	0.47
1:E:185:ARG:HH21	1:E:246:VAL:HG21	1.79	0.47
1:F:48:GLU:HA	1:F:51:LYS:CE	2.44	0.47
1:A:165:ILE:HD12	1:A:260:LEU:HB3	1.97	0.46
1:E:78:LEU:CD2	1:E:88:LYS:HB2	2.43	0.46
1:E:104:HIS:HA	1:E:142:GLU:HG2	1.97	0.46
1:E:215:VAL:HG12	4:E:403:5BM:N23	2.30	0.46
1:A:64:VAL:CG1	1:A:67:LEU:HD21	2.46	0.46
1:A:167:GLU:HG3	1:A:342:PHE:CD2	2.50	0.46
1:F:333:LEU:N	1:F:333:LEU:HD12	2.31	0.46
1:D:111:ILE:O	1:D:115:ILE:HG13	2.16	0.46
1:D:324:TYR:O	1:D:328:GLU:HB2	2.15	0.46
1:E:156:ASP:O	1:E:160:LYS:HG3	2.15	0.46
1:F:45:GLU:C	1:F:46:LEU:HD12	2.41	0.46
1:B:154:SER:HB2	1:B:198:SER:HA	1.98	0.46
1:B:265:TYR:CE2	1:B:321:LEU:HD11	2.46	0.46
1:D:235:ALA:HA	1:D:251:TRP:CD1	2.51	0.46
1:C:71:ASP:O	1:C:92:ARG:HG3	2.16	0.46
1:D:92:ARG:HB2	1:D:93:PRO:HD3	1.97	0.46
1:F:354:PRO:CB	1:F:357:ARG:HH21	2.27	0.46
1:A:100:ARG:HG2	1:A:102:LEU:CD1	2.46	0.46
1:A:178:LEU:HD21	1:A:253:MET:HE1	1.98	0.46
1:A:264:ARG:NH2	1:A:268:PRO:O	2.49	0.46
1:A:333:LEU:CD1	1:A:346:VAL:HG11	2.43	0.46
1:B:54:LEU:HD11	1:B:126:ASN:HB3	1.98	0.46
1:B:122:LEU:HB2	1:B:133:PHE:CD2	2.50	0.46
1:B:195:VAL:HB	1:B:252:SER:HB2	1.98	0.46
1:D:63:LYS:HD2	1:D:63:LYS:N	2.31	0.46
1:D:279:PHE:CE2	1:D:337:VAL:HG11	2.50	0.46
1:E:365:ASN:HA	1:E:370:LYS:HD3	1.98	0.46
1:A:344:GLU:O	1:A:348:LYS:HG2	2.16	0.46
1:B:234:MET:HE2	1:B:234:MET:HB3	1.81	0.46
1:F:72:PHE:CE1	1:F:91:HIS:HD2	2.34	0.46

Continued on next page...

For Manuscript Review

Continued from previous page...

Atom-1	Atom-2	Interatomic distance (Å)	Clash overlap (Å)
1:F:131:VAL:HG21	1:F:201:LEU:HD12	1.98	0.46
1:A:169:ILE:O	1:A:173:VAL:HG23	2.16	0.46
1:A:200:ILE:C	1:A:201:LEU:HD12	2.41	0.46
1:B:317:ALA:HB3	1:B:320:GLU:HG3	1.98	0.46
1:B:339:THR:O	1:B:343:GLN:HG3	2.16	0.46
1:E:103:ILE:HG12	1:E:105:LEU:HG	1.98	0.46
1:F:53:ARG:HD2	1:F:205:ARG:HD2	1.97	0.46
1:A:361:LYS:HG3	1:A:362:MET:N	2.31	0.45
1:C:53:ARG:HD3	1:C:207:GLU:OE2	2.16	0.45
4:C:403:5BM:S26	4:C:403:5BM:C02	3.04	0.45
1:E:111:ILE:O	1:E:115:ILE:HG13	2.15	0.45
1:C:129:TYR:O	1:C:130:ILE:HD13	2.16	0.45
1:F:169:ILE:O	1:F:173:VAL:HG23	2.16	0.45
1:F:344:GLU:HG3	1:F:348:LYS:HZ1	1.80	0.45
1:B:92:ARG:HB2	1:B:93:PRO:HD3	1.99	0.45
1:F:334:PRO:HD2	1:F:338:PHE:HE2	1.81	0.45
1:D:335:ASN:HA	1:D:343:GLN:OE1	2.17	0.45
1:F:318:ILE:HG22	1:F:321:LEU:CG	2.45	0.45
1:B:64:VAL:CG2	1:B:96:LEU:HD12	2.47	0.45
1:B:237:GLU:HG2	1:B:238:ARG:N	2.30	0.45
1:C:127:SER:OG	1:C:128:PRO:HD2	2.17	0.45
1:F:318:ILE:HG12	1:F:319:PHE:N	2.31	0.45
1:C:238:ARG:HD2	1:C:243:HIS:O	2.17	0.45
1:D:91:HIS:HB3	1:D:94:SER:HG	1.80	0.45
1:F:196:LYS:HE3	1:F:198:SER:HB2	1.99	0.45
1:A:92:ARG:HB2	1:A:93:PRO:HD3	1.99	0.45
1:A:201:LEU:HD21	3:A:402:ANP:C5	2.47	0.45
1:A:234:MET:HE3	1:A:238:ARG:NE	2.32	0.45
1:B:154:SER:HA	1:B:201:LEU:HD23	1.99	0.45
1:C:388:ARG:O	1:C:389:LEU:HB2	2.15	0.45
1:D:258:VAL:O	1:D:262:VAL:HG22	2.17	0.45
4:D:403:5BM:C22	4:D:403:5BM:HN05	2.29	0.45
1:B:165:ILE:HD12	1:B:260:LEU:HD22	1.99	0.45
1:B:255:LEU:CD1	1:B:266:PRO:HG2	2.47	0.45
1:C:175:ILE:HG12	1:C:369:ILE:HG23	1.99	0.45
1:C:214:GLY:HA2	4:C:403:5BM:N05	2.32	0.45
1:A:236:PRO:HD3	1:A:251:TRP:CD2	2.52	0.44
1:B:329:PRO:HA	1:B:330:PRO:HD3	1.91	0.44
1:C:173:VAL:O	1:C:177:VAL:HG23	2.16	0.44
1:D:175:ILE:HD13	1:D:373:GLU:HG2	1.99	0.44
1:D:365:ASN:HA	1:D:370:LYS:HE3	1.98	0.44

Continued on next page...

For Manuscript Review

Continued from previous page...

Atom-1	Atom-2	Interatomic distance (Å)	Clash overlap (Å)
1:E:267:ILE:HA	1:E:268:PRO:C	2.41	0.44
1:F:67:LEU:HD22	1:F:72:PHE:HZ	1.81	0.44
1:F:128:PRO:HG2	1:F:129:TYR:CE2	2.52	0.44
1:F:371:ARG:O	1:F:374:VAL:HG22	2.17	0.44
1:B:112:ARG:O	1:B:116:ILE:HG13	2.16	0.44
1:A:172:LYS:HD3	1:A:379:PHE:HB2	1.99	0.44
1:C:194:ASP:OD2	1:C:199:ASN:ND2	2.51	0.44
1:A:123:HIS:CD2	1:B:117:ARG:HG3	2.52	0.44
1:A:324:TYR:O	1:A:328:GLU:HB3	2.17	0.44
1:B:269:PRO:HA	1:B:270:PRO:HD3	1.84	0.44
1:A:164:ARG:NH1	1:A:279:PHE:CD1	2.85	0.44
1:A:243:HIS:CD2	1:A:243:HIS:H	2.36	0.44
1:E:255:LEU:HD12	1:E:266:PRO:HG2	2.00	0.44
1:F:265:TYR:HE2	1:F:267:ILE:HB	1.80	0.44
1:A:116:ILE:O	1:A:120:GLN:HG2	2.17	0.44
1:B:165:ILE:HD12	1:B:260:LEU:HB3	2.00	0.44
1:B:178:LEU:CD2	1:B:253:MET:HE1	2.47	0.44
1:B:88:LYS:HE3	1:B:97:ILE:HG21	1.99	0.44
1:C:240:GLN:OE1	1:C:326:VAL:HG13	2.18	0.44
1:D:216:SER:O	1:D:220:ILE:HG13	2.18	0.44
1:D:324:TYR:CD2	1:D:328:GLU:HG3	2.52	0.44
1:E:46:LEU:N	1:E:46:LEU:HD12	2.33	0.44
1:E:266:PRO:O	1:E:269:PRO:HA	2.18	0.44
1:A:371:ARG:HD2	5:A:502:HOH:O	2.18	0.44
1:C:100:ARG:HE	1:C:102:LEU:HD21	1.82	0.44
1:C:188:HIS:O	1:C:190:ILE:HG13	2.18	0.44
1:C:334:PRO:HD2	1:C:338:PHE:CE2	2.53	0.44
1:F:351:ILE:O	1:F:357:ARG:HD3	2.17	0.44
1:D:316:MET:HE3	1:D:321:LEU:HD13	1.99	0.43
1:E:317:ALA:HB3	1:E:320:GLU:CB	2.47	0.43
1:E:351:ILE:CG2	1:E:356:GLU:HB2	2.44	0.43
1:B:223:MET:HE3	4:B:403:5BM:H31	1.99	0.43
1:F:122:LEU:HD12	1:F:133:PHE:CD1	2.52	0.43
1:B:49:GLN:O	1:B:53:ARG:HG2	2.17	0.43
1:B:152:GLY:HA3	1:B:202:VAL:HG23	1.99	0.43
1:E:161:GLU:OE1	1:E:161:GLU:HA	2.17	0.43
1:A:185:ARG:HH12	1:A:360:LEU:CD1	2.28	0.43
1:D:98:MET:CE	1:D:134:TYR:CB	2.96	0.43
1:D:237:GLU:HG2	1:D:238:ARG:N	2.33	0.43
1:D:250:ILE:HG23	1:D:363:LEU:HD11	2.01	0.43
1:F:108:LYS:O	1:F:112:ARG:HG3	2.19	0.43

Continued on next page...

For Manuscript Review

Continued from previous page...

Atom-1	Atom-2	Interatomic distance (Å)	Clash overlap (Å)
1:F:250:ILE:O	1:F:251:TRP:C	2.61	0.43
1:F:250:ILE:HD11	1:F:360:LEU:CD2	2.48	0.43
1:A:54:LEU:HD11	1:A:126:ASN:HB3	1.99	0.43
1:B:46:LEU:O	1:B:51:LYS:HE3	2.19	0.43
1:B:167:GLU:OE1	1:B:339:THR:HG23	2.19	0.43
3:B:402:ANP:H5'1	3:B:402:ANP:H8	2.00	0.43
1:C:64:VAL:CG2	1:C:96:LEU:HD12	2.48	0.43
1:C:266:PRO:O	1:C:269:PRO:HA	2.19	0.43
1:D:267:ILE:HG21	1:D:321:LEU:CD1	2.46	0.43
1:D:267:ILE:O	1:D:269:PRO:HD3	2.19	0.43
1:E:192:HIS:HD2	1:E:194:ASP:C	2.27	0.43
1:F:147:MET:HE2	4:F:403:5BM:H32	1.99	0.43
1:A:117:ARG:O	1:A:120:GLN:HB2	2.18	0.43
1:A:192:HIS:ND1	1:A:213:PHE:HB3	2.33	0.43
1:A:247:GLN:CB	1:A:357:ARG:HH11	2.30	0.43
1:B:258:VAL:CG2	1:B:346:VAL:HG11	2.49	0.43
1:C:109:PRO:HA	1:C:112:ARG:HD3	2.01	0.43
1:D:57:PHE:CZ	1:D:132:GLY:HA3	2.53	0.43
1:E:60:GLN:HB3	1:E:96:LEU:HD11	1.99	0.43
1:A:267:ILE:HD12	1:A:325:ILE:HG12	2.00	0.43
1:B:169:ILE:O	1:B:173:VAL:HG23	2.18	0.43
1:B:358:ALA:HB3	1:B:363:LEU:HD21	2.01	0.43
1:D:62:ALA:HA	1:E:117:ARG:HH22	1.84	0.43
1:E:192:HIS:ND1	1:E:213:PHE:HB3	2.34	0.43
1:E:265:TYR:HD1	1:E:266:PRO:HD2	1.80	0.43
1:F:231:ARG:HB3	1:F:265:TYR:CE1	2.53	0.43
1:C:250:ILE:HD11	1:C:360:LEU:HD23	2.00	0.43
1:B:74:ARG:HA	1:B:89:VAL:HG12	1.99	0.43
1:B:239:LEU:HD13	1:B:322:LEU:HD22	2.01	0.43
1:C:268:PRO:HA	1:C:269:PRO:HD3	1.96	0.43
4:D:403:5BM:N05	4:D:403:5BM:C22	2.82	0.43
4:D:403:5BM:C02	4:D:403:5BM:S26	3.07	0.43
1:E:98:MET:HE3	1:E:134:TYR:HB2	2.01	0.43
1:E:269:PRO:HA	1:E:270:PRO:HD3	1.91	0.43
1:F:271:ASP:OD1	1:F:274:GLU:HB2	2.18	0.43
1:A:88:LYS:HE3	1:A:97:ILE:HG21	2.00	0.43
1:D:265:TYR:HE2	1:D:267:ILE:HB	1.83	0.43
1:F:269:PRO:HA	1:F:270:PRO:HD3	1.91	0.43
1:F:270:PRO:HB3	1:F:274:GLU:CD	2.44	0.43
1:A:256:SER:O	1:A:260:LEU:HG	2.19	0.42
1:C:169:ILE:HG23	1:C:379:PHE:HD1	1.84	0.42

Continued on next page...

For Manuscript Review

Continued from previous page...

Atom-1	Atom-2	Interatomic distance (Å)	Clash overlap (Å)
1:C:202:VAL:HG11	1:C:379:PHE:HZ	1.84	0.42
1:D:152:GLY:H	1:D:203:ASN:HA	1.83	0.42
1:E:131:VAL:HG22	1:E:209:LYS:HB2	2.01	0.42
1:B:175:ILE:HD13	1:B:373:GLU:HG2	2.00	0.42
1:C:269:PRO:HA	1:C:270:PRO:HD3	1.80	0.42
1:D:358:ALA:HB3	1:D:363:LEU:HD21	2.01	0.42
1:E:274:GLU:O	1:E:278:ILE:HG13	2.20	0.42
1:A:162:ALA:O	1:A:163:LYS:HB2	2.19	0.42
1:A:173:VAL:O	1:A:177:VAL:HG23	2.19	0.42
1:B:372:SER:HA	1:B:375:GLU:HG2	2.00	0.42
1:C:219:LEU:HD11	1:C:223:MET:CE	2.49	0.42
1:C:329:PRO:HA	1:C:330:PRO:HD3	1.93	0.42
1:D:48:GLU:HG2	1:D:52:LYS:NZ	2.34	0.42
1:D:102:LEU:N	1:D:102:LEU:HD12	2.34	0.42
1:E:334:PRO:HD2	1:E:338:PHE:CE2	2.54	0.42
1:A:72:PHE:HE2	1:A:137:PHE:HE1	1.68	0.42
1:B:60:GLN:HA	1:B:63:LYS:HE2	2.02	0.42
1:E:269:PRO:HG2	1:E:324:TYR:OH	2.19	0.42
1:E:328:GLU:HB3	1:E:329:PRO:CD	2.47	0.42
1:F:45:GLU:HG3	1:F:46:LEU:CD1	2.49	0.42
1:F:72:PHE:HE2	1:F:137:PHE:CE1	2.36	0.42
1:A:46:LEU:HD12	1:A:46:LEU:N	2.34	0.42
1:A:114:GLN:HG2	1:A:118:GLU:OE2	2.19	0.42
1:A:238:ARG:HG2	1:A:244:TYR:HD1	1.81	0.42
1:E:175:ILE:HG12	1:E:369:ILE:HG23	2.02	0.42
1:A:321:LEU:O	1:A:325:ILE:HG13	2.19	0.42
1:F:188:HIS:O	1:F:190:ILE:HG13	2.20	0.42
3:F:402:ANP:O2A	3:F:402:ANP:O1B	2.38	0.42
1:A:72:PHE:HE2	1:A:137:PHE:CE1	2.38	0.42
1:C:57:PHE:CZ	1:C:132:GLY:HA3	2.55	0.42
1:D:231:ARG:HB3	1:D:265:TYR:CE1	2.54	0.42
1:F:147:MET:HB3	4:F:403:5BM:C31	2.50	0.42
1:B:328:GLU:HB3	1:B:329:PRO:CD	2.46	0.42
1:C:199:ASN:OD1	1:C:212:ASP:HB2	2.19	0.42
1:C:264:ARG:NH2	1:C:270:PRO:HA	2.35	0.42
1:D:187:LYS:CG	1:D:188:HIS:CE1	3.02	0.42
1:A:102:LEU:HD12	1:A:102:LEU:N	2.35	0.42
4:A:403:5BM:S06	4:A:403:5BM:C22	3.08	0.42
1:C:255:LEU:HD23	1:C:255:LEU:HA	1.77	0.42
1:D:162:ALA:HB2	1:D:386:THR:CG2	2.49	0.42
1:D:169:ILE:HG23	1:D:379:PHE:CD1	2.53	0.42

Continued on next page...

For Manuscript Review

Continued from previous page...

Atom-1	Atom-2	Interatomic distance (Å)	Clash overlap (Å)
1:F:105:LEU:HD13	1:F:222:SER:CB	2.50	0.42
1:F:187:LYS:HB2	1:F:188:HIS:ND1	2.35	0.42
1:F:324:TYR:CD2	1:F:328:GLU:HG3	2.55	0.42
1:A:57:PHE:HE1	1:A:148:GLU:HB2	1.84	0.42
1:C:172:LYS:NZ	1:C:379:PHE:HB3	2.34	0.42
1:D:152:GLY:HA3	1:D:202:VAL:HG23	2.02	0.42
1:E:154:SER:HA	1:E:201:LEU:HD23	2.02	0.42
1:F:105:LEU:HB3	1:F:107:ILE:HD11	2.02	0.42
1:B:161:GLU:OE1	1:B:161:GLU:HA	2.20	0.41
1:A:57:PHE:CE1	1:A:148:GLU:HB2	2.56	0.41
1:F:235:ALA:HB1	1:F:236:PRO:HD2	2.02	0.41
1:F:354:PRO:HA	1:F:357:ARG:NE	2.33	0.41
1:A:131:VAL:HB	4:A:403:5BM:H10	2.02	0.41
1:B:237:GLU:HB2	1:B:243:HIS:CD2	2.55	0.41
1:B:265:TYR:HE2	1:B:267:ILE:HB	1.80	0.41
1:D:175:ILE:HG12	1:D:369:ILE:HG23	2.02	0.41
1:D:234:MET:HE2	1:D:238:ARG:NH1	2.36	0.41
1:E:319:PHE:HD2	1:F:240:GLN:HA	1.85	0.41
1:B:148:GLU:OE2	1:B:209:LYS:NZ	2.52	0.41
1:C:324:TYR:HA	1:C:328:GLU:HG3	2.01	0.41
1:E:184:LEU:HB3	1:E:190:ILE:HG13	2.02	0.41
1:E:185:ARG:HH21	1:E:246:VAL:HG23	1.85	0.41
1:A:112:ARG:O	1:A:116:ILE:HG13	2.21	0.41
1:C:86:VAL:HG11	3:C:402:ANP:C8	2.51	0.41
1:D:264:ARG:NE	1:D:274:GLU:OE2	2.53	0.41
1:E:74:ARG:HG3	1:E:74:ARG:O	2.20	0.41
1:F:220:ILE:HA	4:F:403:5BM:C10	2.50	0.41
1:D:134:TYR:HE2	1:D:148:GLU:HA	1.85	0.41
1:E:213:PHE:CE2	4:E:403:5BM:H09	2.56	0.41
1:E:323:ASP:O	1:E:323:ASP:OD1	2.39	0.41
1:E:324:TYR:CD2	1:E:328:GLU:HG3	2.55	0.41
1:F:107:ILE:HG21	1:F:111:ILE:HB	2.00	0.41
1:E:91:HIS:O	1:E:94:SER:O	2.39	0.41
1:E:195:VAL:HB	1:E:252:SER:HB2	2.02	0.41
1:F:271:ASP:OD1	1:F:274:GLU:CB	2.68	0.41
1:B:258:VAL:O	1:B:259:GLU:C	2.64	0.41
1:C:108:LYS:HG3	5:C:509:HOH:O	2.21	0.41
1:D:51:LYS:O	1:D:52:LYS:C	2.63	0.41
1:D:61:LYS:HE3	1:D:133:PHE:O	2.21	0.41
1:F:343:GLN:O	1:F:346:VAL:HG22	2.21	0.41
1:A:67:LEU:HD12	1:A:135:GLY:HA3	2.01	0.41

Continued on next page...

For Manuscript Review

Continued from previous page...

Atom-1	Atom-2	Interatomic distance (Å)	Clash overlap (Å)
1:A:164:ARG:HE	1:A:164:ARG:HB2	1.76	0.41
1:A:167:GLU:OE2	1:A:339:THR:CG2	2.69	0.41
1:A:220:ILE:N	1:A:220:ILE:CD1	2.84	0.41
1:B:193:ARG:HG2	1:B:193:ARG:HH11	1.86	0.41
1:E:264:ARG:NH1	1:E:274:GLU:HG3	2.36	0.41
1:E:270:PRO:HB2	1:E:275:LEU:CD2	2.51	0.41
1:C:234:MET:HE3	1:C:239:LEU:HD23	2.03	0.41
1:C:265:TYR:HA	1:C:266:PRO:HD3	1.92	0.41
1:E:53:ARG:NE	1:E:205:ARG:HD3	2.36	0.41
1:E:339:THR:OG1	1:E:340:PRO:HD2	2.20	0.41
1:F:91:HIS:HB3	1:F:94:SER:O	2.21	0.41
1:A:121:VAL:HG12	1:A:121:VAL:O	2.19	0.40
1:B:252:SER:O	1:B:253:MET:C	2.63	0.40
1:D:250:ILE:CG2	1:D:363:LEU:HD11	2.50	0.40
1:E:169:ILE:HG23	1:E:379:PHE:HD1	1.85	0.40
1:E:269:PRO:CG	1:E:324:TYR:OH	2.69	0.40
1:B:48:GLU:HG3	1:B:49:GLN:N	2.36	0.40
1:B:318:ILE:HG23	1:B:319:PHE:N	2.36	0.40
1:B:334:PRO:HD2	1:B:338:PHE:HE2	1.84	0.40
1:C:154:SER:OG	1:C:157:GLN:HG3	2.21	0.40
1:C:175:ILE:HD13	1:C:373:GLU:HG2	2.03	0.40
1:C:324:TYR:O	1:C:328:GLU:HB2	2.21	0.40
1:C:334:PRO:HD2	1:C:338:PHE:CD2	2.56	0.40
1:D:328:GLU:HB3	1:D:329:PRO:CD	2.51	0.40
1:F:332:LYS:HD2	1:F:347:ASN:CG	2.47	0.40
1:E:267:ILE:HD13	1:E:324:TYR:CD1	2.57	0.40
1:B:275:LEU:HD22	1:B:334:PRO:HG3	2.03	0.40
1:C:130:ILE:HG23	1:C:213:PHE:CE2	2.57	0.40
1:C:237:GLU:HG2	1:C:238:ARG:N	2.36	0.40
1:C:252:SER:O	1:C:253:MET:C	2.65	0.40
1:F:129:TYR:CZ	1:F:179:ARG:HD2	2.56	0.40
1:F:255:LEU:N	1:F:350:LEU:HD21	2.35	0.40
4:F:403:5BM:S26	4:F:403:5BM:C02	3.10	0.40
1:A:234:MET:CE	1:A:238:ARG:HB3	2.51	0.40
1:A:333:LEU:HD12	1:A:333:LEU:H	1.87	0.40
1:D:255:LEU:HD11	1:D:266:PRO:HG2	2.03	0.40
1:E:239:LEU:CD1	1:E:325:ILE:HD12	2.46	0.40
1:F:94:SER:O	1:F:96:LEU:N	2.52	0.40
1:F:261:ALA:HB1	1:F:338:PHE:CZ	2.57	0.40

There are no symmetry-related clashes.

For Manuscript Review

5.3 Torsion angles (i)

5.3.1 Protein backbone (i)

In the following table, the Percentiles column shows the percent Ramachandran outliers of the chain as a percentile score with respect to all X-ray entries followed by that with respect to entries of similar resolution.

The Analysed column shows the number of residues for which the backbone conformation was analysed, and the total number of residues.

Mol	Chain	Analysed	Favoured	Allowed	Outliers	Percentiles	
1	A	297/345 (86%)	291 (98%)	6 (2%)	0	100	100
1	B	298/345 (86%)	292 (98%)	6 (2%)	0	100	100
1	C	300/345 (87%)	296 (99%)	4 (1%)	0	100	100
1	D	300/345 (87%)	295 (98%)	5 (2%)	0	100	100
1	E	298/345 (86%)	296 (99%)	2 (1%)	0	100	100
1	F	292/345 (85%)	284 (97%)	8 (3%)	0	100	100
All	All	1785/2070 (86%)	1754 (98%)	31 (2%)	0	100	100

There are no Ramachandran outliers to report.

5.3.2 Protein sidechains (i)

In the following table, the Percentiles column shows the percent sidechain outliers of the chain as a percentile score with respect to all X-ray entries followed by that with respect to entries of similar resolution.

The Analysed column shows the number of residues for which the sidechain conformation was analysed, and the total number of residues.

Mol	Chain	Analysed	Rotameric	Outliers	Percentiles	
1	A	264/298 (89%)	264 (100%)	0	100	100
1	B	265/298 (89%)	265 (100%)	0	100	100
1	C	266/298 (89%)	266 (100%)	0	100	100
1	D	265/298 (89%)	265 (100%)	0	100	100
1	E	265/298 (89%)	265 (100%)	0	100	100
1	F	260/298 (87%)	260 (100%)	0	100	100
All	All	1585/1788 (89%)	1585 (100%)	0	100	100

For Manuscript Review

There are no protein residues with a non-rotameric sidechain to report.

Sometimes sidechains can be flipped to improve hydrogen bonding and reduce clashes. All (25) such sidechains are listed below:

Mol	Chain	Res	Type
1	A	192	HIS
1	A	335	ASN
1	A	343	GLN
1	B	104	HIS
1	B	126	ASN
1	B	243	HIS
1	B	343	GLN
1	C	49	GLN
1	C	113	ASN
1	C	327	ASN
1	C	343	GLN
1	D	49	GLN
1	D	104	HIS
1	D	120	GLN
1	D	188	HIS
1	D	218	GLN
1	D	343	GLN
1	E	104	HIS
1	E	113	ASN
1	E	123	HIS
1	E	335	ASN
1	E	343	GLN
1	E	366	HIS
1	F	114	GLN
1	F	343	GLN

5.3.3 RNA (i)

There are no RNA molecules in this entry.

5.4 Non-standard residues in protein, DNA, RNA chains (i)

There are no non-standard protein/DNA/RNA residues in this entry.

5.5 Carbohydrates (i)

There are no oligosaccharides in this entry.

For Manuscript Review

5.6 Ligand geometry (i)

Of 18 ligands modelled in this entry, 6 are monoatomic - leaving 12 for Mogul analysis.

In the following table, the Counts columns list the number of bonds (or angles) for which Mogul statistics could be retrieved, the number of bonds (or angles) that are observed in the model and the number of bonds (or angles) that are defined in the Chemical Component Dictionary. The Link column lists molecule types, if any, to which the group is linked. The Z score for a bond length (or angle) is the number of standard deviations the observed value is removed from the expected value. A bond length (or angle) with $|Z| > 2$ is considered an outlier worth inspection. RMSZ is the root-mean-square of all Z scores of the bond lengths (or angles).

Mol	Type	Chain	Res	Link	Bond lengths			Bond angles		
					Counts	RMSZ	$\#\|Z\| > 2$	Counts	RMSZ	$\#\|Z\| > 2$
3	ANP	F	402	2	32,33,33	2.04	5 (15%)	44,52,52	1.46	5 (11%)
3	ANP	B	402	2	32,33,33	1.97	4 (12%)	44,52,52	1.79	14 (31%)
3	ANP	A	402	2	32,33,33	2.19	6 (18%)	44,52,52	1.42	4 (9%)
4	5BM	C	403	-	23,27,27	2.64	8 (34%)	28,36,36	1.62	4 (14%)
4	5BM	A	403	-	23,27,27	2.77	7 (30%)	28,36,36	1.85	6 (21%)
3	ANP	E	402	2	32,33,33	2.01	4 (12%)	44,52,52	1.38	3 (6%)
4	5BM	B	403	-	23,27,27	2.58	8 (34%)	28,36,36	2.21	5 (17%)
3	ANP	C	402	2	32,33,33	2.04	5 (15%)	44,52,52	1.66	9 (20%)
4	5BM	F	403	-	23,27,27	2.68	8 (34%)	28,36,36	1.70	5 (17%)
4	5BM	E	403	-	23,27,27	2.63	8 (34%)	28,36,36	1.86	4 (14%)
4	5BM	D	403	-	23,27,27	2.72	8 (34%)	28,36,36	2.46	9 (32%)
3	ANP	D	402	2	32,33,33	2.05	6 (18%)	44,52,52	1.57	6 (13%)

In the following table, the Chirals column lists the number of chiral outliers, the number of chiral centers analysed, the number of these observed in the model and the number defined in the Chemical Component Dictionary. Similar counts are reported in the Torsion and Rings columns. '-' means no outliers of that kind were identified.

Mol	Type	Chain	Res	Link	Chirals	Torsions	Rings
3	ANP	F	402	2	-	4/18/38/38	0/3/3/3
3	ANP	B	402	2	-	5/18/38/38	0/3/3/3
3	ANP	A	402	2	-	4/18/38/38	0/3/3/3
4	5BM	C	403	-	-	2/14/24/24	0/2/2/2
4	5BM	A	403	-	-	2/14/24/24	0/2/2/2
3	ANP	E	402	2	-	2/18/38/38	0/3/3/3
4	5BM	B	403	-	-	2/14/24/24	0/2/2/2
3	ANP	C	402	2	-	4/18/38/38	0/3/3/3

Continued on next page...

For Manuscript Review

Continued from previous page...

Mol	Type	Chain	Res	Link	Chirals	Torsions	Rings
4	5BM	F	403	-	-	1/14/24/24	0/2/2/2
4	5BM	E	403	-	-	4/14/24/24	0/2/2/2
4	5BM	D	403	-	-	5/14/24/24	0/2/2/2
3	ANP	D	402	2	-	3/18/38/38	0/3/3/3

All (77) bond length outliers are listed below:

Mol	Chain	Res	Type	Atoms	Z	Observed(Å)	Ideal(Å)
3	A	402	ANP	PB-O3A	7.70	1.68	1.59
3	C	402	ANP	PB-O3A	7.45	1.68	1.59
3	F	402	ANP	PB-O3A	6.91	1.67	1.59
3	E	402	ANP	PB-O3A	6.85	1.67	1.59
3	D	402	ANP	PB-O3A	6.63	1.67	1.59
3	B	402	ANP	PB-O3A	6.05	1.66	1.59
4	F	403	5BM	C04-N05	6.01	1.46	1.35
4	E	403	5BM	C24-N25	5.94	1.45	1.35
4	A	403	5BM	C04-N05	5.92	1.45	1.35
4	C	403	5BM	C04-N05	5.91	1.45	1.35
4	A	403	5BM	C24-N25	5.88	1.45	1.35
4	D	403	5BM	C24-N25	5.86	1.45	1.35
3	F	402	ANP	PG-N3B	5.80	1.78	1.63
3	A	402	ANP	PG-N3B	5.78	1.78	1.63
4	B	403	5BM	C04-N05	5.69	1.45	1.35
3	E	402	ANP	PG-N3B	5.68	1.78	1.63
4	E	403	5BM	C04-N05	5.63	1.45	1.35
3	D	402	ANP	PG-N3B	5.59	1.78	1.63
4	B	403	5BM	C24-N25	5.59	1.45	1.35
4	D	403	5BM	C04-N05	5.58	1.45	1.35
4	C	403	5BM	C24-N25	5.58	1.45	1.35
4	F	403	5BM	C24-N25	5.57	1.45	1.35
3	C	402	ANP	PG-N3B	5.50	1.77	1.63
4	E	403	5BM	C22-C21	5.37	1.54	1.43
3	B	402	ANP	PG-N3B	5.33	1.77	1.63
3	B	402	ANP	PG-O1G	5.14	1.54	1.46
4	A	403	5BM	C22-C21	5.09	1.53	1.43
4	D	403	5BM	C22-C21	5.07	1.53	1.43
4	C	403	5BM	C02-C01	5.06	1.53	1.43
4	F	403	5BM	C22-C21	5.00	1.53	1.43
4	C	403	5BM	C22-C21	4.99	1.53	1.43
4	F	403	5BM	C02-C01	4.92	1.53	1.43
4	A	403	5BM	C27-S26	4.92	1.83	1.77
4	D	403	5BM	C02-C01	4.90	1.53	1.43

Continued on next page...

For Manuscript Review

Continued from previous page...

Mol	Chain	Res	Type	Atoms	Z	Observed(Å)	Ideal(Å)
4	B	403	5BM	C22-C21	4.83	1.52	1.43
4	B	403	5BM	C02-C01	4.82	1.52	1.43
4	E	403	5BM	C02-C01	4.80	1.52	1.43
4	D	403	5BM	C07-S06	4.74	1.83	1.77
3	E	402	ANP	PG-O1G	4.66	1.53	1.46
4	A	403	5BM	C02-C01	4.66	1.52	1.43
3	A	402	ANP	PG-O1G	4.66	1.53	1.46
3	C	402	ANP	PG-O1G	4.60	1.53	1.46
3	F	402	ANP	PG-O1G	4.59	1.53	1.46
3	D	402	ANP	PG-O1G	4.33	1.53	1.46
4	B	403	5BM	C27-S26	4.29	1.82	1.77
4	F	403	5BM	C27-S26	4.24	1.82	1.77
4	A	403	5BM	C07-S06	4.22	1.82	1.77
4	D	403	5BM	C27-S26	4.13	1.82	1.77
4	F	403	5BM	C07-S06	3.78	1.82	1.77
4	C	403	5BM	C07-S06	3.68	1.81	1.77
4	E	403	5BM	C27-S26	3.47	1.81	1.77
4	C	403	5BM	C27-S26	3.34	1.81	1.77
4	E	403	5BM	C07-S06	3.06	1.81	1.77
3	A	402	ANP	PB-O2B	-2.83	1.49	1.56
3	D	402	ANP	PB-O1B	2.69	1.50	1.46
4	E	403	5BM	C28-N33	2.69	1.46	1.37
4	A	403	5BM	C28-N33	2.66	1.46	1.37
3	A	402	ANP	PB-O1B	2.57	1.50	1.46
4	B	403	5BM	C07-S06	2.52	1.80	1.77
4	B	403	5BM	C08-N13	2.49	1.46	1.37
4	D	403	5BM	C28-N33	2.43	1.45	1.37
4	C	403	5BM	C28-N33	2.42	1.45	1.37
3	F	402	ANP	PB-O1B	2.38	1.49	1.46
3	B	402	ANP	PB-O2B	-2.38	1.50	1.56
3	D	402	ANP	PB-O2B	-2.34	1.50	1.56
4	F	403	5BM	C28-N33	2.33	1.45	1.37
4	C	403	5BM	C08-N13	2.25	1.45	1.37
4	F	403	5BM	C08-N13	2.25	1.45	1.37
4	D	403	5BM	C08-N13	2.25	1.45	1.37
3	F	402	ANP	PB-O2B	-2.22	1.50	1.56
4	E	403	5BM	C08-N13	2.20	1.45	1.37
3	E	402	ANP	PB-O2B	-2.18	1.50	1.56
4	B	403	5BM	C28-N33	2.16	1.45	1.37
3	D	402	ANP	O4'-C1'	-2.07	1.37	1.42
3	C	402	ANP	PB-O1B	2.06	1.49	1.46
3	C	402	ANP	PB-O2B	-2.04	1.51	1.56

Continued on next page...

For Manuscript Review

Continued from previous page...

Mol	Chain	Res	Type	Atoms	Z	Observed(Å)	Ideal(Å)
3	A	402	ANP	O3'-C3'	-2.03	1.38	1.43

All (74) bond angle outliers are listed below:

Mol	Chain	Res	Type	Atoms	Z	Observed(^{o})	Ideal(^{o})
4	D	403	5BM	C07-S06-C04	8.10	116.55	102.88
4	B	403	5BM	C22-C21-C01	-7.20	101.43	117.47
4	B	403	5BM	C07-S06-C04	6.15	113.27	102.88
4	E	403	5BM	C07-S06-C04	6.15	113.26	102.88
3	C	402	ANP	O2B-PB-O1B	5.57	121.61	109.92
3	D	402	ANP	O2B-PB-O1B	5.42	121.29	109.92
4	A	403	5BM	C22-C21-C01	-5.24	105.80	117.47
3	F	402	ANP	O2B-PB-O1B	5.21	120.84	109.92
4	C	403	5BM	C22-C21-C01	-5.03	106.26	117.47
4	F	403	5BM	C22-C21-C01	-4.92	106.52	117.47
3	E	402	ANP	O2B-PB-O1B	4.77	119.93	109.92
4	D	403	5BM	C22-C21-C01	-4.70	106.99	117.47
4	E	403	5BM	C22-C21-C01	-4.69	107.03	117.47
3	B	402	ANP	O2B-PB-O1B	4.65	119.66	109.92
4	A	403	5BM	C27-S26-C24	4.58	110.61	102.88
3	A	402	ANP	O1G-PG-N3B	-4.46	105.21	111.77
3	A	402	ANP	O2B-PB-O1B	4.45	119.26	109.92
3	E	402	ANP	O1G-PG-N3B	-4.45	105.22	111.77
4	C	403	5BM	C27-S26-C24	4.37	110.27	102.88
3	F	402	ANP	O1G-PG-N3B	-4.15	105.66	111.77
3	D	402	ANP	O1G-PG-N3B	-4.15	105.67	111.77
4	E	403	5BM	C01-C04-N05	4.10	127.63	119.49
4	D	403	5BM	C08-C07-S06	4.03	127.55	119.17
4	A	403	5BM	C02-C01-C21	-3.85	108.90	117.47
3	C	402	ANP	O1G-PG-N3B	-3.79	106.19	111.77
3	B	402	ANP	O3A-PB-N3B	-3.72	96.28	106.59
4	D	403	5BM	C07-C08-N13	3.70	125.89	121.34
4	B	403	5BM	C01-C04-N05	3.66	126.75	119.49
4	F	403	5BM	C27-S26-C24	3.62	109.00	102.88
4	D	403	5BM	C27-S26-C24	3.59	108.95	102.88
4	C	403	5BM	C07-S06-C04	3.58	108.93	102.88
3	B	402	ANP	C2'-C3'-C4'	-3.54	95.76	102.64
4	B	403	5BM	C02-C01-C21	-3.42	109.86	117.47
4	F	403	5BM	C01-C04-N05	3.41	126.25	119.49
4	E	403	5BM	C02-C01-C21	-3.37	109.96	117.47
3	B	402	ANP	O4'-C1'-N9	3.32	114.61	108.06
4	B	403	5BM	C27-S26-C24	3.25	108.37	102.88

Continued on next page...

For Manuscript Review

Continued from previous page...

Mol	Chain	Res	Type	Atoms	Z	Observed(°)	Ideal(°)
3	C	402	ANP	O2G-PG-O3G	3.16	116.05	107.64
4	F	403	5BM	C02-C01-C21	-3.13	110.51	117.47
3	B	402	ANP	O1G-PG-N3B	-3.07	107.25	111.77
4	D	403	5BM	C12-C07-S06	-2.96	114.00	120.22
4	D	403	5BM	C01-C04-N05	2.96	125.35	119.49
3	F	402	ANP	O2G-PG-O3G	2.95	115.49	107.64
4	A	403	5BM	C07-S06-C04	2.88	107.74	102.88
3	C	402	ANP	C5'-C4'-C3'	2.86	125.89	115.18
3	D	402	ANP	PB-O3A-PA	-2.83	122.65	132.62
4	D	403	5BM	C09-C08-N13	-2.82	114.67	120.13
4	A	403	5BM	C07-C08-N13	-2.80	117.90	121.34
3	C	402	ANP	O3'-C3'-C2'	2.80	120.87	111.82
3	C	402	ANP	PB-O3A-PA	-2.72	123.02	132.62
3	B	402	ANP	C4-N9-C1'	-2.69	120.19	126.59
3	B	402	ANP	O2'-C2'-C3'	-2.67	103.18	111.82
3	D	402	ANP	O2G-PG-O3G	2.65	114.68	107.64
3	C	402	ANP	O4'-C1'-N9	2.60	113.19	108.06
3	B	402	ANP	O2G-PG-O3G	2.48	114.23	107.64
3	F	402	ANP	PB-O3A-PA	-2.45	123.99	132.62
3	B	402	ANP	O4'-C4'-C3'	2.39	109.85	105.11
3	A	402	ANP	O2G-PG-O3G	2.34	113.88	107.64
3	B	402	ANP	O3'-C3'-C2'	2.28	119.20	111.82
3	E	402	ANP	O4'-C1'-N9	2.24	112.47	108.06
3	B	402	ANP	C1'-N9-C8	2.22	132.15	127.14
4	D	403	5BM	C02-C01-C21	-2.20	112.56	117.47
3	B	402	ANP	O4'-C1'-C2'	-2.20	101.84	106.64
3	C	402	ANP	C4-N9-C1'	-2.20	121.36	126.59
3	B	402	ANP	C5'-C4'-C3'	2.19	123.40	115.18
3	D	402	ANP	O3A-PB-N3B	-2.19	100.52	106.59
3	B	402	ANP	O4'-C4'-C5'	-2.18	102.19	109.37
4	F	403	5BM	C28-C27-S26	2.18	123.70	119.17
3	C	402	ANP	C1'-N9-C8	2.18	132.05	127.14
3	D	402	ANP	C4-N9-C1'	-2.17	121.42	126.59
3	A	402	ANP	O5'-PA-O1A	-2.12	100.80	109.07
4	C	403	5BM	C01-C04-N05	2.11	123.67	119.49
4	A	403	5BM	C01-C04-N05	2.07	123.60	119.49
3	F	402	ANP	O3A-PB-N3B	-2.04	100.94	106.59

There are no chirality outliers.

All (38) torsion outliers are listed below:

For Manuscript Review

Mol	Chain	Res	Type	Atoms
3	A	402	ANP	PB-N3B-PG-O1G
3	A	402	ANP	C3'-C4'-C5'-O5'
3	B	402	ANP	PB-N3B-PG-O1G
3	B	402	ANP	PG-N3B-PB-O1B
3	B	402	ANP	PG-N3B-PB-O3A
3	C	402	ANP	PB-N3B-PG-O1G
3	C	402	ANP	PA-O3A-PB-O1B
3	D	402	ANP	PB-N3B-PG-O1G
3	D	402	ANP	PG-N3B-PB-O1B
3	E	402	ANP	PB-N3B-PG-O1G
3	E	402	ANP	PG-N3B-PB-O1B
3	F	402	ANP	PB-N3B-PG-O1G
3	F	402	ANP	PG-N3B-PB-O1B
3	F	402	ANP	PA-O3A-PB-O1B
3	F	402	ANP	PA-O3A-PB-O2B
4	A	403	5BM	C04-C01-C21-C24
4	D	403	5BM	C08-C07-S06-C04
4	D	403	5BM	C12-C07-S06-C04
4	E	403	5BM	C04-C01-C21-C24
4	E	403	5BM	C08-C07-S06-C04
4	E	403	5BM	C12-C07-S06-C04
3	A	402	ANP	O4'-C4'-C5'-O5'
4	C	403	5BM	C04-C01-C21-C24
4	D	403	5BM	C01-C21-C22-N23
4	E	403	5BM	C01-C21-C22-N23
4	A	403	5BM	C28-C27-S26-C24
4	D	403	5BM	C04-C01-C21-C24
4	F	403	5BM	C04-C01-C21-C24
3	A	402	ANP	PG-N3B-PB-O1B
3	D	402	ANP	PB-O3A-PA-O2A
3	C	402	ANP	PA-O3A-PB-O2B
4	B	403	5BM	C21-C01-C02-N03
4	B	403	5BM	C02-C01-C21-C22
4	C	403	5BM	C28-C27-S26-C24
4	D	403	5BM	C28-C27-S26-C24
3	C	402	ANP	O4'-C4'-C5'-O5'
3	B	402	ANP	C5'-O5'-PA-O1A
3	B	402	ANP	O4'-C4'-C5'-O5'

There are no ring outliers.

11 monomers are involved in 48 short contacts:

For Manuscript Review

Mol	Chain	Res	Type	Clashes	Symm-Clashes
3	F	402	ANP	4	0
3	B	402	ANP	2	0
3	A	402	ANP	1	0
4	C	403	5BM	3	0
4	A	403	5BM	9	0
4	B	403	5BM	4	0
3	C	402	ANP	1	0
4	F	403	5BM	10	0
4	E	403	5BM	5	0
4	D	403	5BM	7	0
3	D	402	ANP	2	0

The following is a two-dimensional graphical depiction of Mogul quality analysis of bond lengths, bond angles, torsion angles, and ring geometry for all instances of the Ligand of Interest. In addition, ligands with molecular weight > 250 and outliers as shown on the validation Tables will also be included. For torsion angles, if less then 5% of the Mogul distribution of torsion angles is within 10 degrees of the torsion angle in question, then that torsion angle is considered an outlier. Any bond that is central to one or more torsion angles identified as an outlier by Mogul will be highlighted in the graph. For rings, the root-mean-square deviation (RMSD) between the ring in question and similar rings identified by Mogul is calculated over all ring torsion angles. If the average RMSD is greater than 60 degrees and the minimal RMSD between the ring in question and any Mogul-identified rings is also greater than 60 degrees, then that ring is considered an outlier. The outliers are highlighted in purple. The color gray indicates Mogul did not find sufficient equivalents in the CSD to analyse the geometry.

For Manuscript Review

Ligand ANP F 402

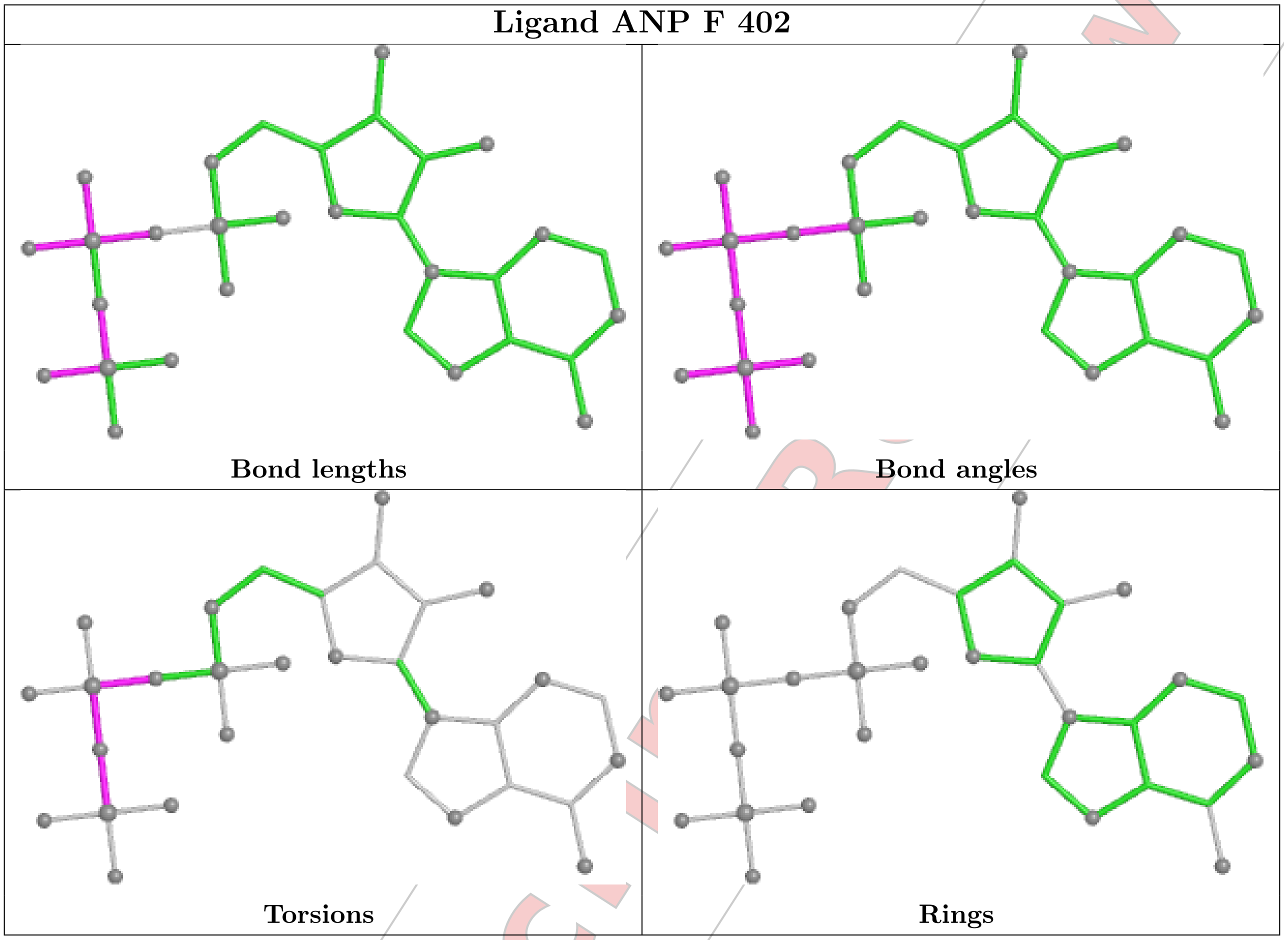

For Manuscript Review

Ligand ANP B 402

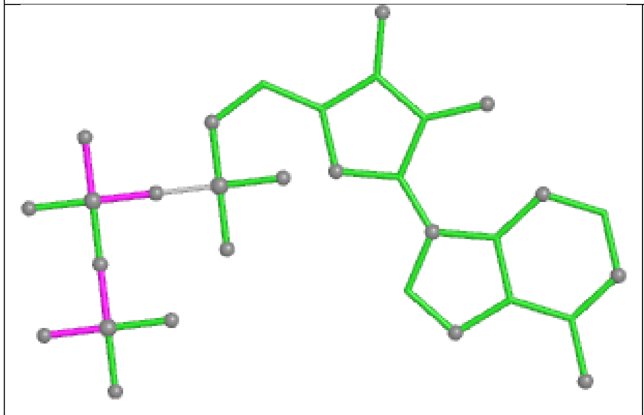

Bond lengths

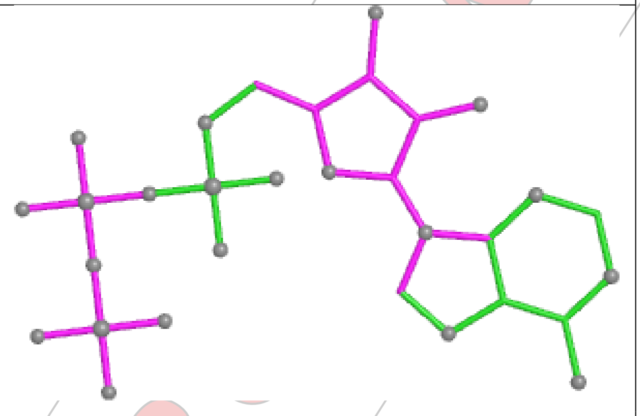

Bond angles

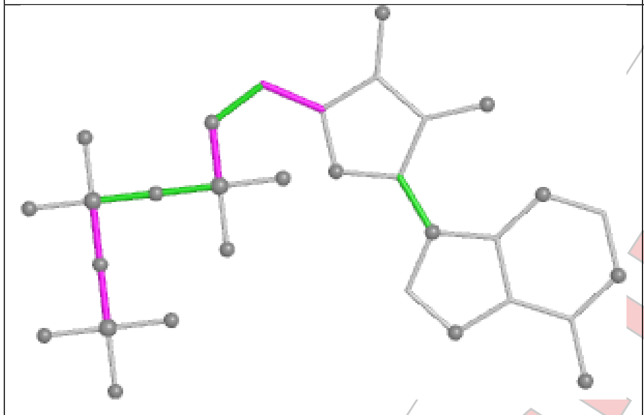

Torsions

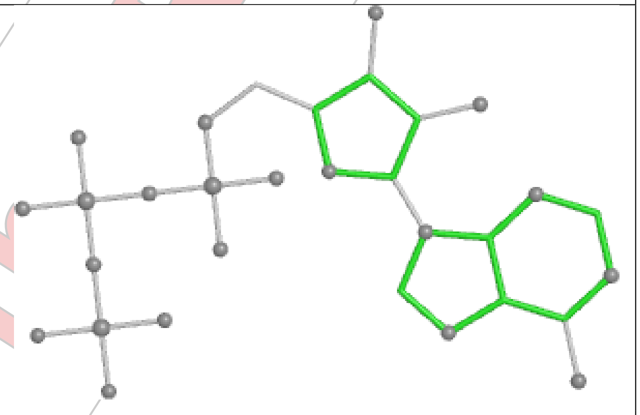

Rings

For Manuscript Review

Ligand ANP A 402

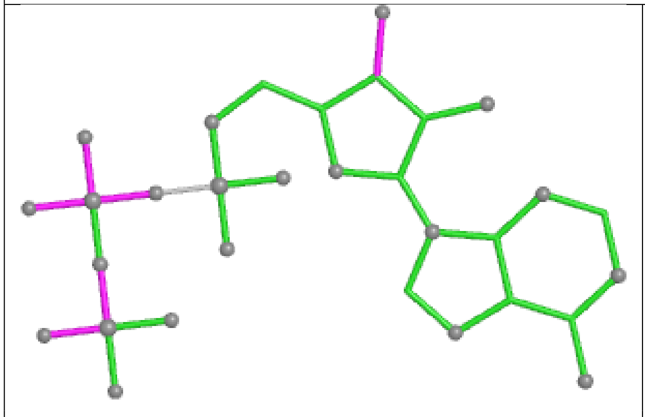
Bond lengths

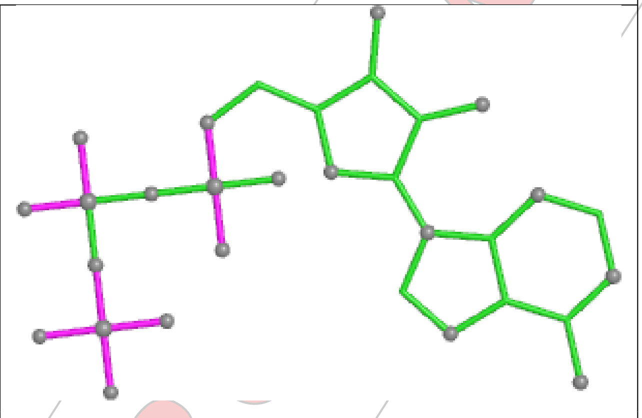
Bond angles

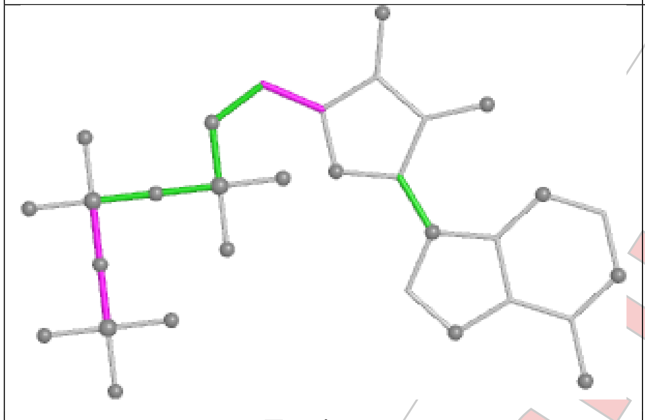
Torsions

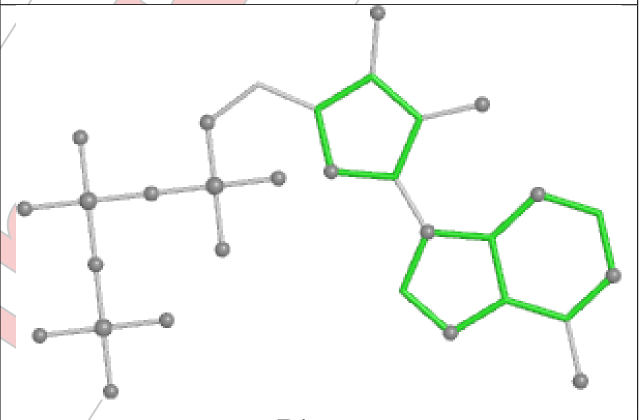
Rings

Ligand 5BM C 403

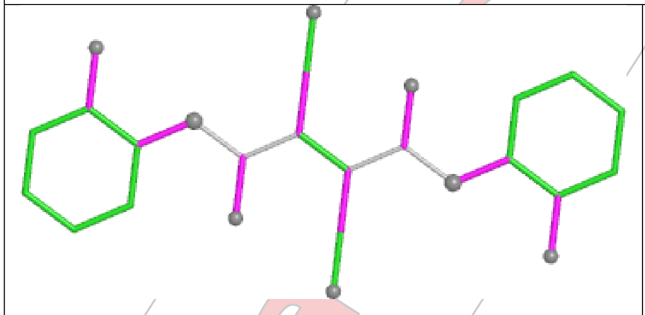
Bond lengths

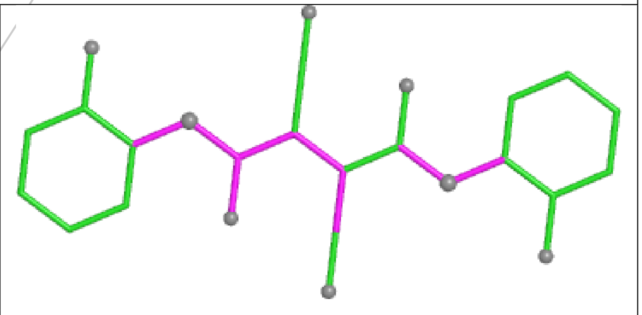
Bond angles

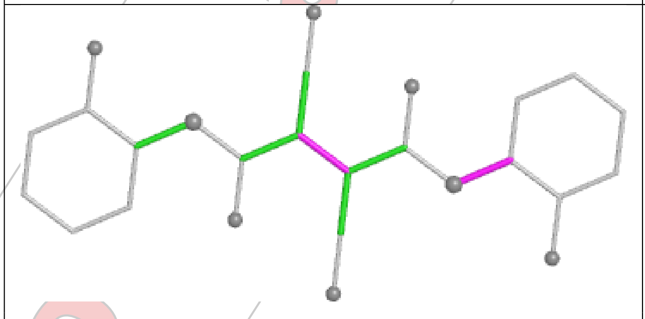
Torsions

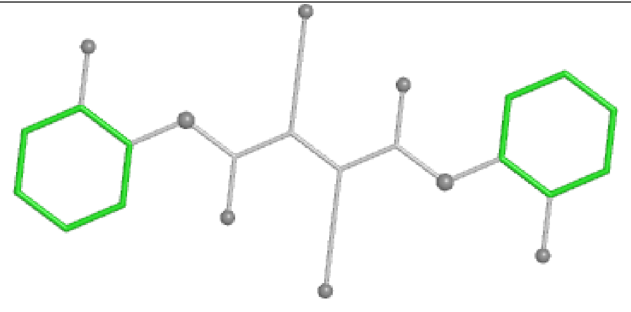
Rings

Ligand 5BM A 403

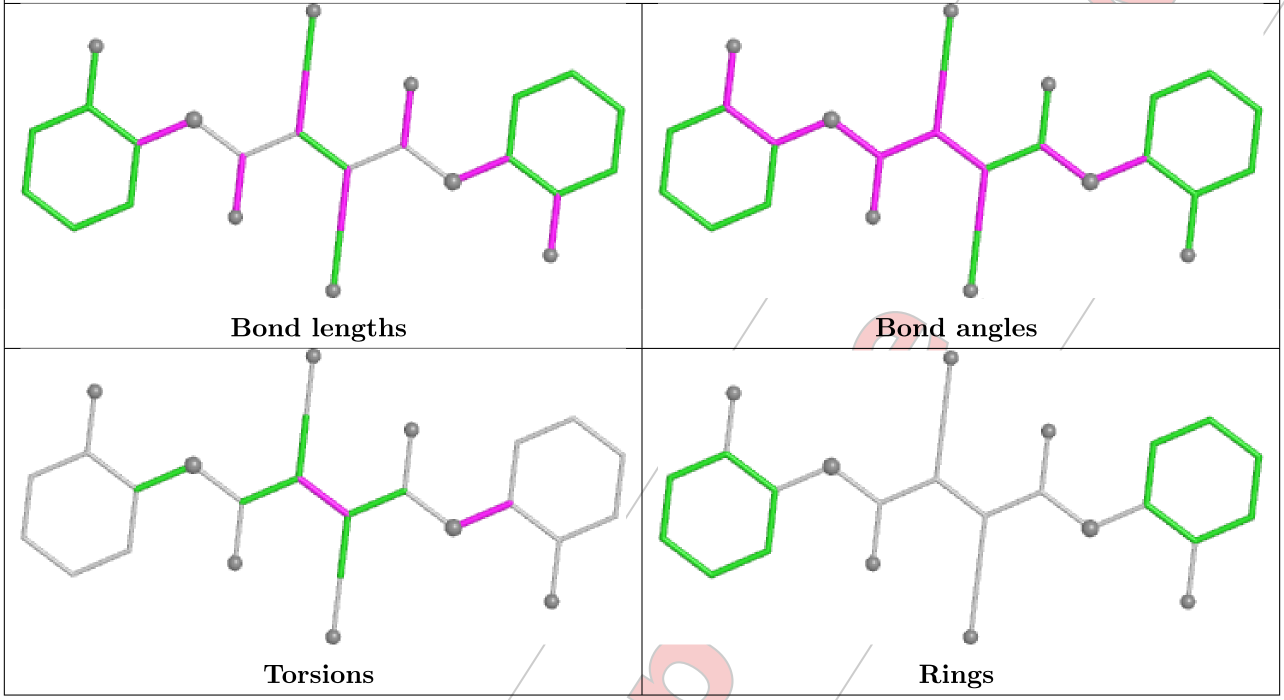

Ligand ANP E 402

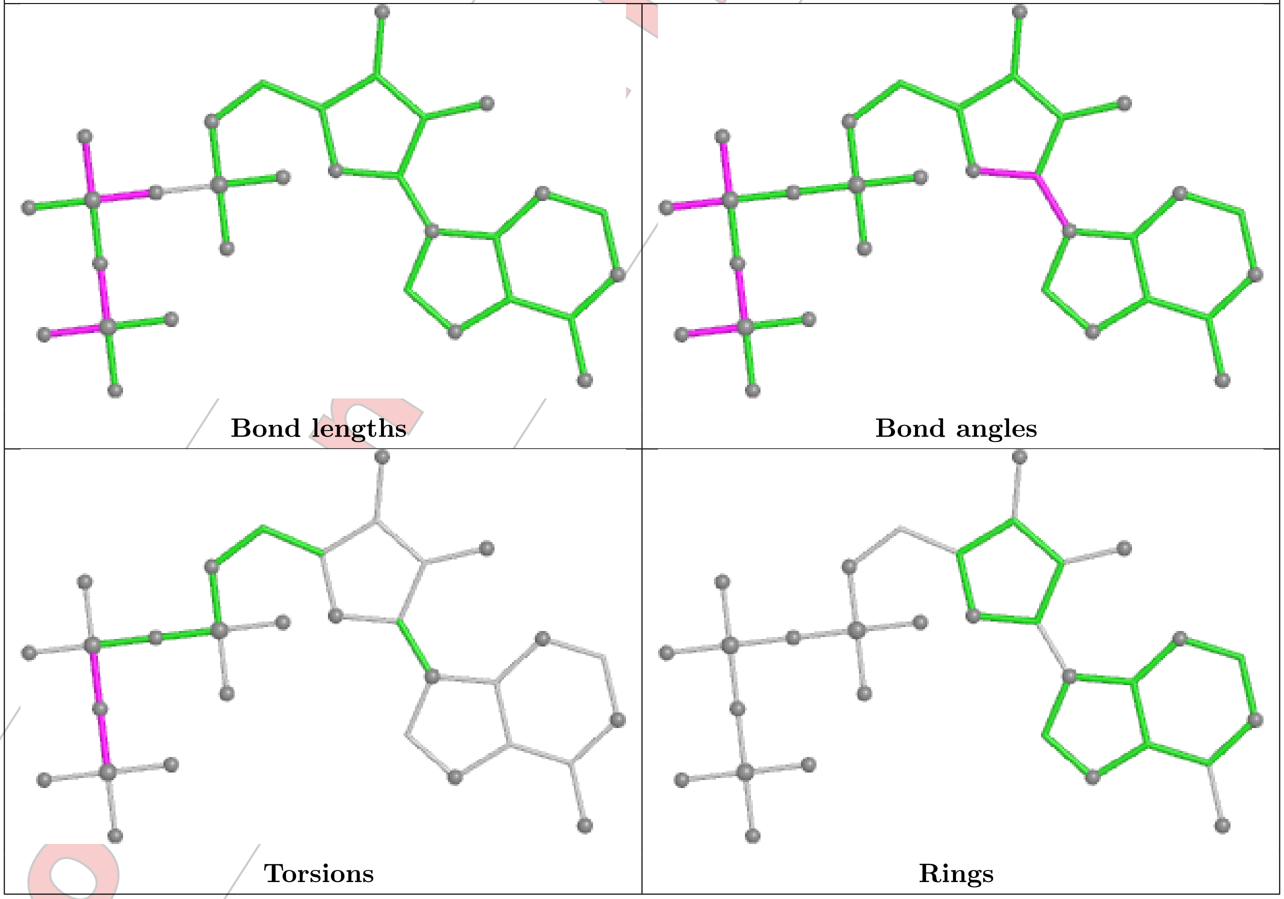

Ligand 5BM B 403

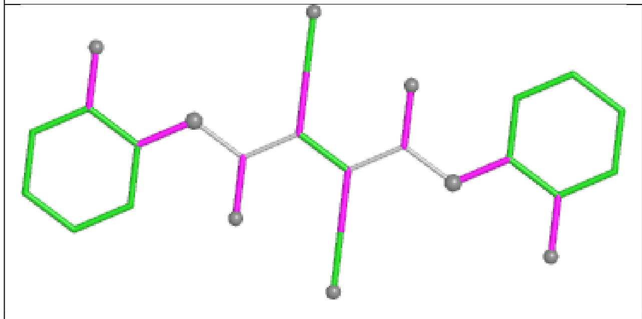
Bond lengths

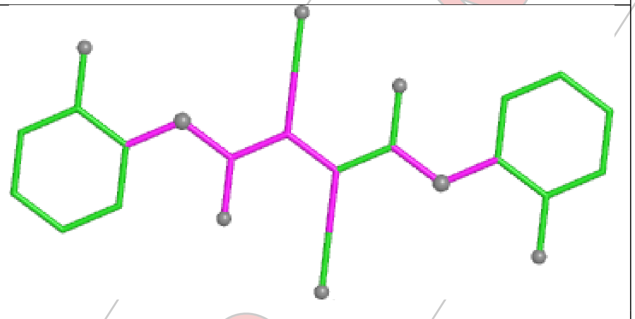
Bond angles

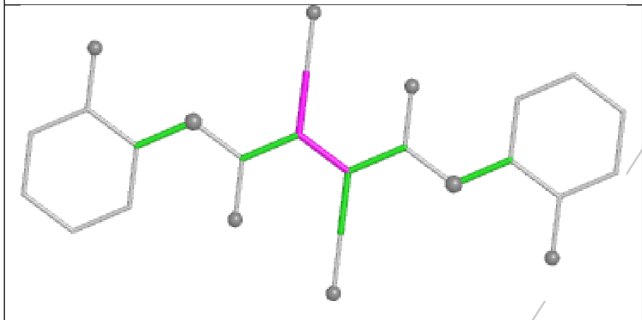
Torsions

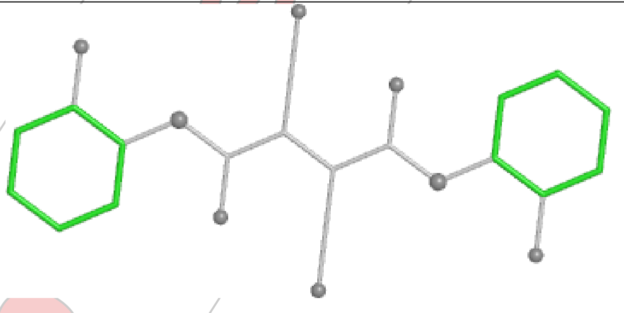
Rings

Ligand ANP C 402

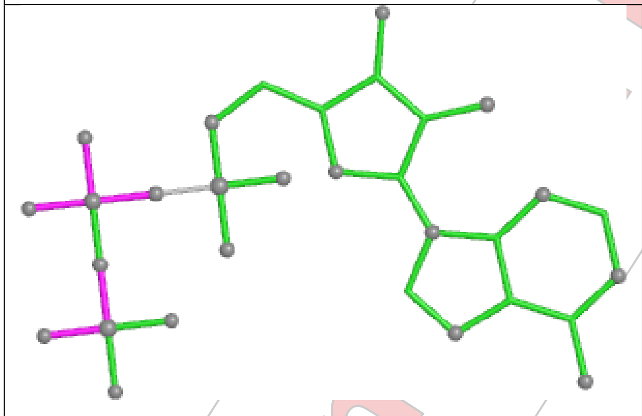
Bond lengths

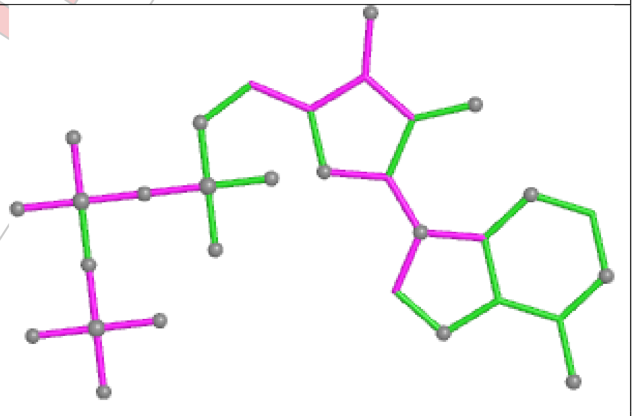
Bond angles

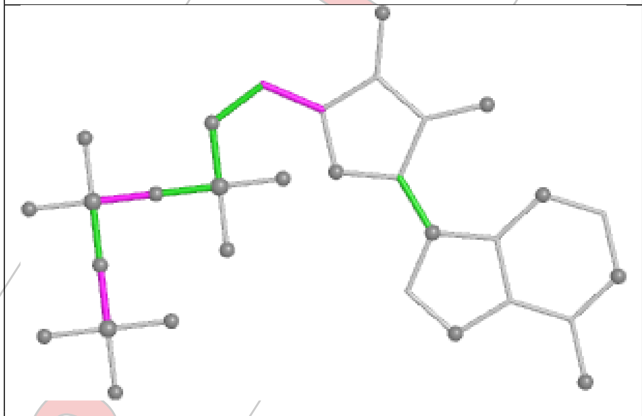
Torsions

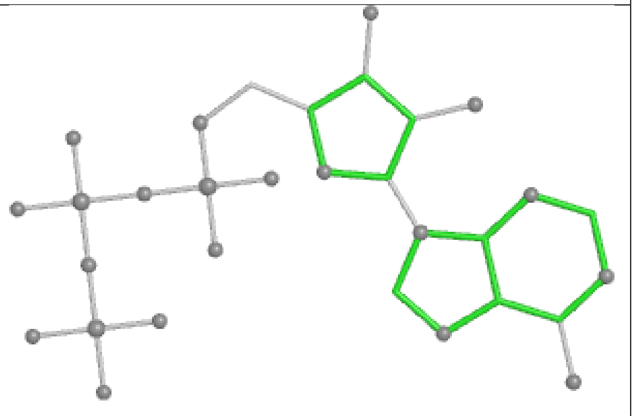
Rings

Ligand 5BM F 403

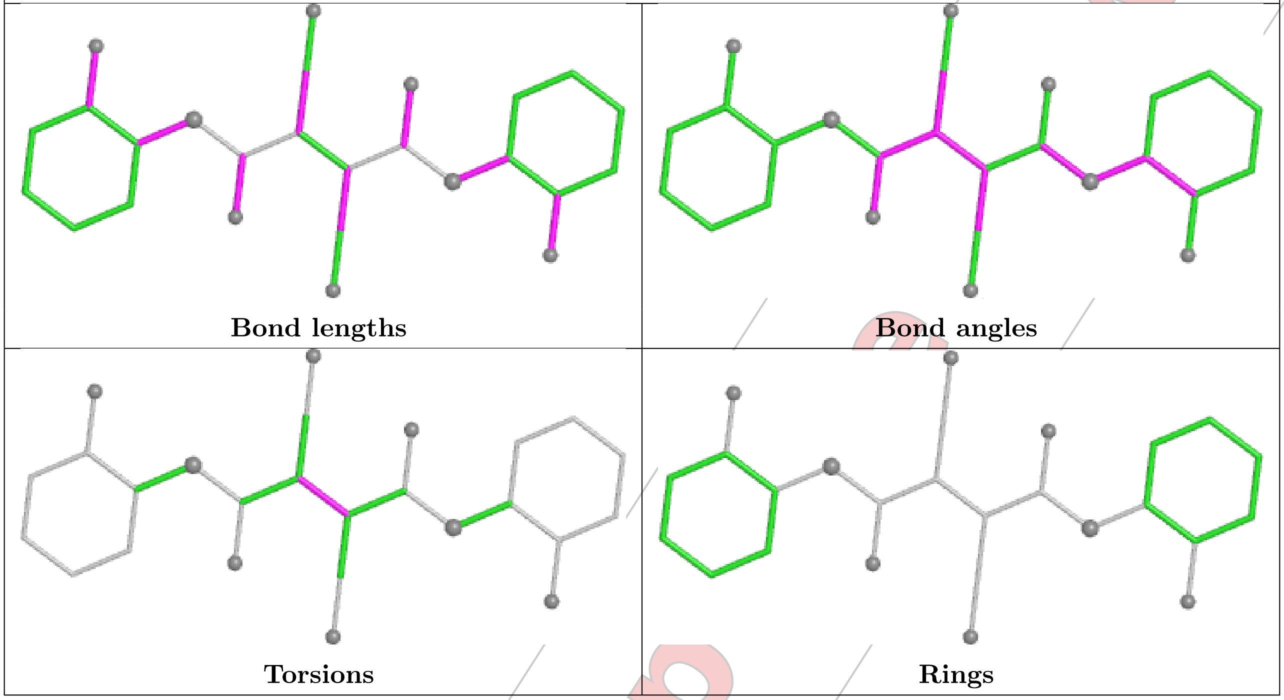

Ligand 5BM E 403

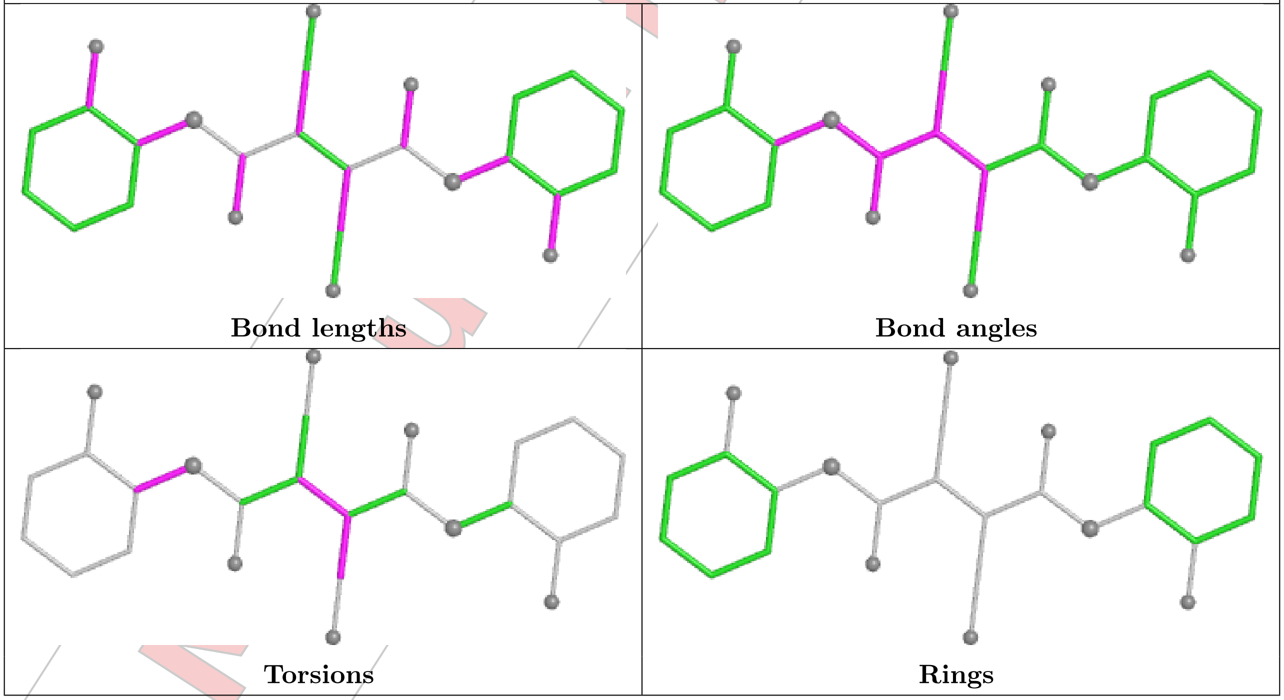

Ligand 5BM D 403

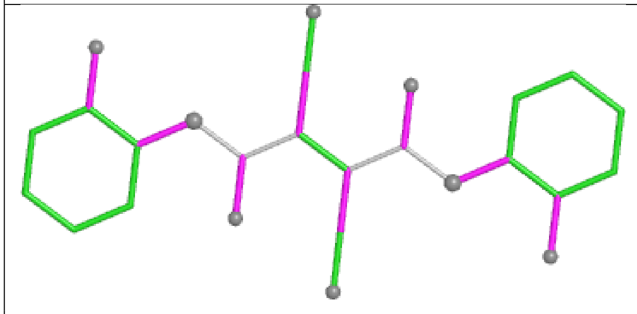

Bond lengths

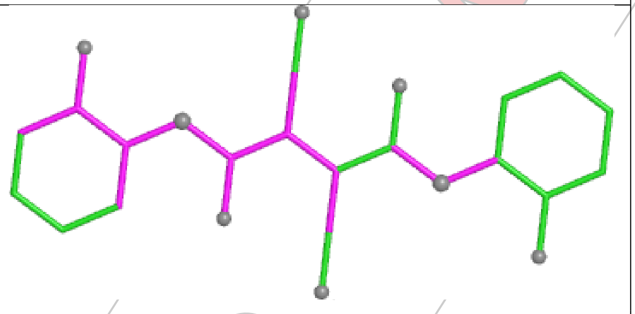

Bond angles

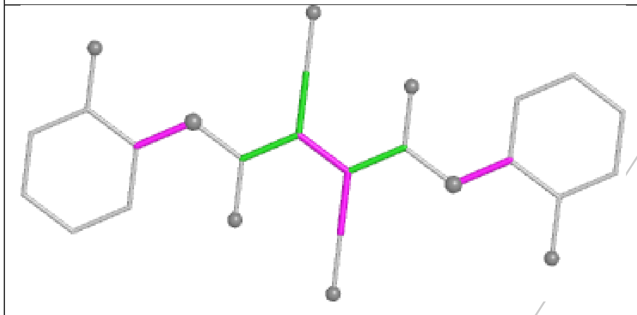

Torsions

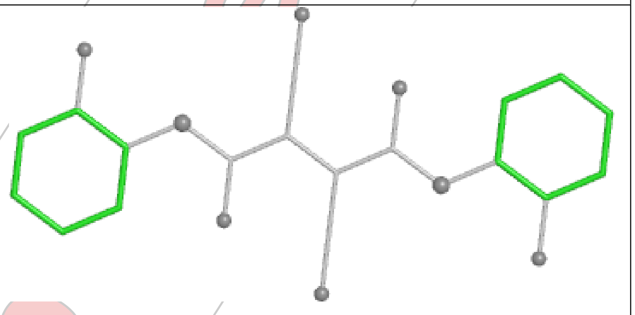

Rings

Ligand ANP D 402

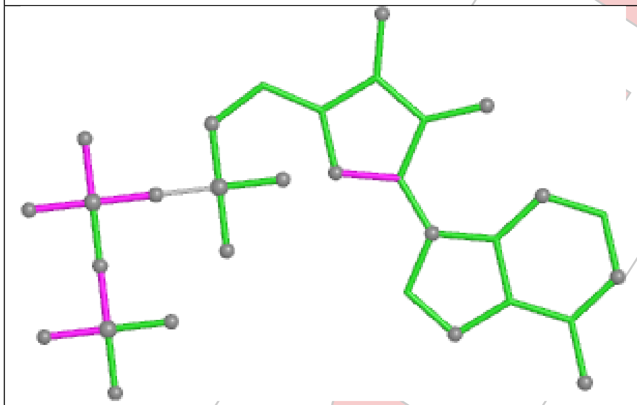

Bond lengths

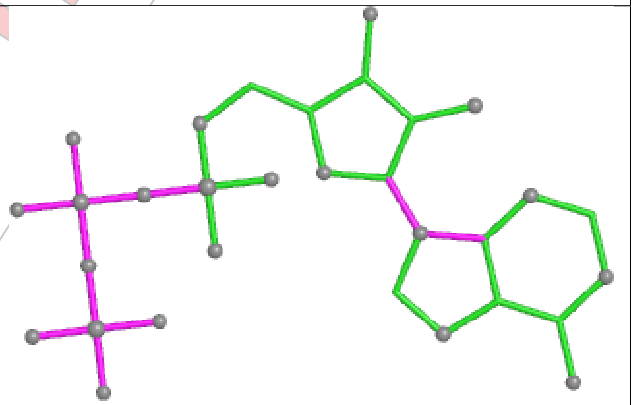

Bond angles

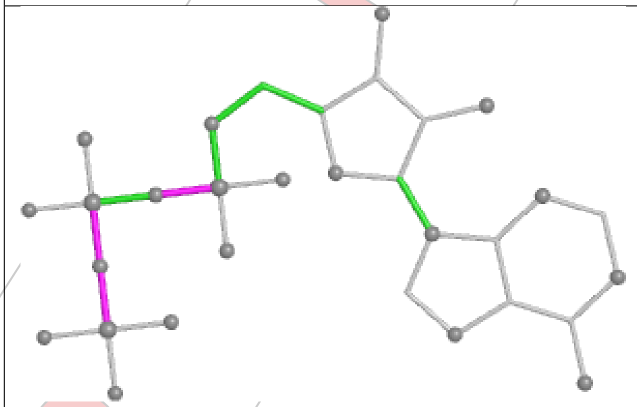

Torsions

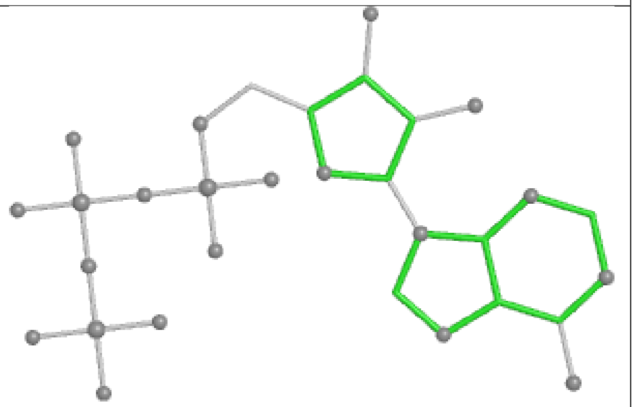

Rings

5.7 Other polymers (i)

There are no such residues in this entry.

5.8 Polymer linkage issues (i)

There are no chain breaks in this entry.

For Manuscript Review

6 Fit of model and data (i)

6.1 Protein, DNA and RNA chains (i)

In the following table, the column labelled '#RSRZ> 2' contains the number (and percentage) of RSRZ outliers, followed by percent RSRZ outliers for the chain as percentile scores relative to all X-ray entries and entries of similar resolution. The OWAB column contains the minimum, median, 95th percentile and maximum values of the occupancy-weighted average B-factor per residue. The column labelled 'Q< 0.9' lists the number of (and percentage) of residues with an average occupancy less than 0.9.

Mol	Chain	Analysed	<RSRZ>	#RSRZ>2			OWAB(Å^2)	Q<0.9
1	A	303/345 (87%)	-0.05	6 (1%)	65	44	21, 42, 72, 93	0
1	B	304/345 (88%)	-0.01	10 (3%)	49	29	21, 43, 73, 121	0
1	C	306/345 (88%)	0.04	8 (2%)	57	36	23, 46, 76, 98	0
1	D	306/345 (88%)	-0.04	2 (0%)	84	69	24, 43, 71, 97	0
1	E	304/345 (88%)	0.36	17 (5%)	30	17	21, 54, 90, 107	0
1	F	298/345 (86%)	0.33	17 (5%)	29	17	28, 55, 92, 116	0
All	All	1821/2070 (87%)	0.10	60 (3%)	49	29	21, 46, 82, 121	0

All (60) RSRZ outliers are listed below:

Mol	Chain	Res	Type	RSRZ
1	C	228	VAL	5.2
1	C	82	ASN	4.2
1	B	82	ASN	3.8
1	A	242	THR	3.7
1	E	326	VAL	3.6
1	B	267	ILE	3.6
1	F	318	ILE	3.6
1	F	82	ASN	3.5
1	F	323	ASP	3.4
1	E	319	PHE	3.4
1	E	318	ILE	3.3
1	B	244	TYR	3.3
1	E	82	ASN	3.3
1	C	389	LEU	3.2
1	C	271	ASP	3.2
1	B	280	GLY	3.2
1	E	322	LEU	3.1
1	B	83	GLY	3.1
1	E	230	THR	3.0

Continued on next page...

For Manuscript Review

Continued from previous page...

Mol	Chain	Res	Type	RSRZ
1	A	267	ILE	2.9
1	E	243	HIS	2.8
1	B	81	GLY	2.8
1	F	267	ILE	2.8
1	B	389	LEU	2.7
1	A	81	GLY	2.7
1	F	80	ALA	2.6
1	E	325	ILE	2.6
1	C	244	TYR	2.5
1	D	229	GLY	2.5
1	F	83	GLY	2.5
1	F	125	CYS	2.4
1	F	140	ASP	2.4
1	E	240	GLN	2.4
1	E	125	CYS	2.4
1	C	81	GLY	2.4
1	A	376	GLU	2.4
1	C	230	THR	2.3
1	F	104	HIS	2.3
1	A	125	CYS	2.3
1	F	320	GLU	2.3
1	E	239	LEU	2.3
1	C	243	HIS	2.3
1	F	326	VAL	2.3
1	F	321	LEU	2.2
1	F	319	PHE	2.2
1	F	168	GLU	2.2
1	E	80	ALA	2.2
1	E	321	LEU	2.1
1	E	320	GLU	2.1
1	B	268	PRO	2.1
1	B	271	ASP	2.1
1	F	325	ILE	2.1
1	A	244	TYR	2.1
1	F	81	GLY	2.1
1	E	268	PRO	2.1
1	F	375	GLU	2.1
1	E	104	HIS	2.0
1	B	230	THR	2.0
1	E	324	TYR	2.0
1	D	156	ASP	2.0

For Manuscript Review

6.2 Non-standard residues in protein, DNA, RNA chains (i)

There are no non-standard protein/DNA/RNA residues in this entry.

6.3 Carbohydrates (i)

There are no oligosaccharides in this entry.

6.4 Ligands (i)

In the following table, the Atoms column lists the number of modelled atoms in the group and the number defined in the chemical component dictionary. The B-factors column lists the minimum, median, 95th percentile and maximum values of B factors of atoms in the group. The column labelled 'Q< 0.9' lists the number of atoms with occupancy less than 0.9.

Mol	Type	Chain	Res	Atoms	RSCC	RSR	B-factors($Å^2$)	Q<0.9
4	5BM	A	403	26/26	0.85	0.19	36,62,82,93	0
4	5BM	B	403	26/26	0.86	0.19	39,58,76,79	0
4	5BM	C	403	26/26	0.90	0.16	41,59,86,89	0
4	5BM	E	403	26/26	0.90	0.15	37,56,85,102	0
4	5BM	D	403	26/26	0.91	0.15	38,53,67,71	0
2	MG	F	401	1/1	0.91	0.11	58,58,58,58	0
4	5BM	F	403	26/26	0.91	0.16	42,60,78,87	0
3	ANP	B	402	31/31	0.92	0.12	25,43,58,62	0
3	ANP	E	402	31/31	0.93	0.10	39,54,72,83	0
2	MG	D	401	1/1	0.94	0.08	41,41,41,41	0
3	ANP	F	402	31/31	0.94	0.10	36,56,73,100	0
3	ANP	C	402	31/31	0.94	0.09	36,47,58,63	0
3	ANP	D	402	31/31	0.94	0.09	34,49,61,68	0
2	MG	A	401	1/1	0.95	0.07	47,47,47,47	0
3	ANP	A	402	31/31	0.96	0.08	33,47,62,65	0
2	MG	E	401	1/1	0.96	0.07	36,36,36,36	0
2	MG	C	401	1/1	0.96	0.07	47,47,47,47	0
2	MG	B	401	1/1	0.97	0.15	35,35,35,35	0

The following is a graphical depiction of the model fit to experimental electron density of all instances of the Ligand of Interest. In addition, ligands with molecular weight > 250 and outliers as shown on the geometry validation Tables will also be included. Each fit is shown from different orientation to approximate a three-dimensional view.

For Manuscript Review

Electron density around 5BM A 403:

2mF$_o$-DF$_c$ (at 0.7 rmsd) in gray
mF$_o$-DF$_c$ (at 3 rmsd) in purple (negative)
and green (positive)

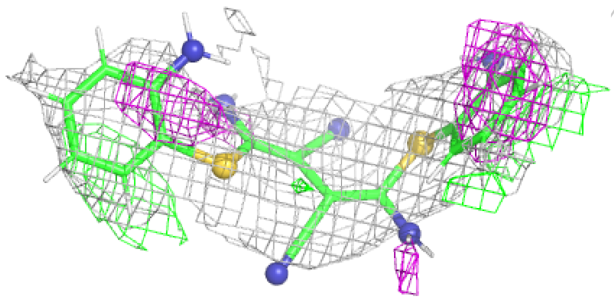

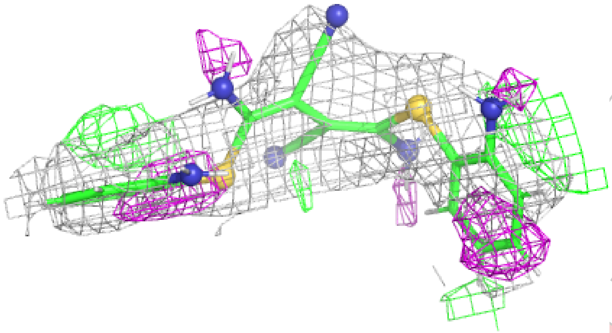

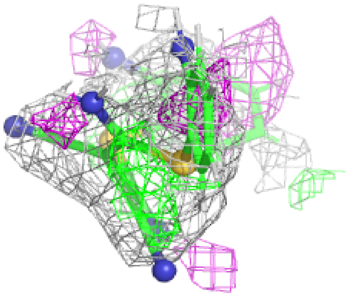

Electron density around 5BM B 403:

2mF$_o$-DF$_c$ (at 0.7 rmsd) in gray
mF$_o$-DF$_c$ (at 3 rmsd) in purple (negative)
and green (positive)

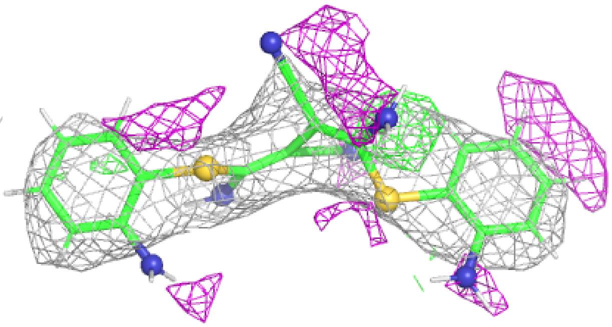

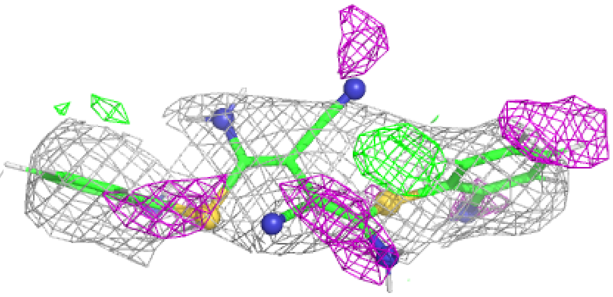

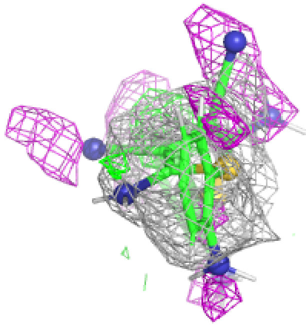

Electron density around 5BM C 403:

$2mF_o$-DF_c (at 0.7 rmsd) in gray
mF_o-DF_c (at 3 rmsd) in purple (negative)
and green (positive)

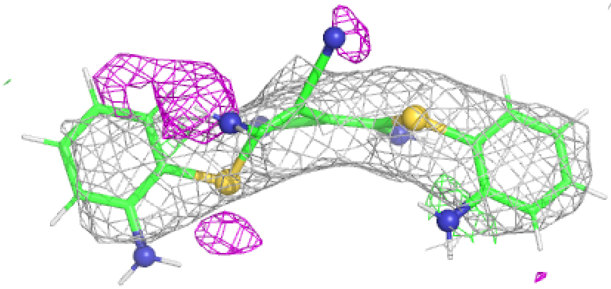

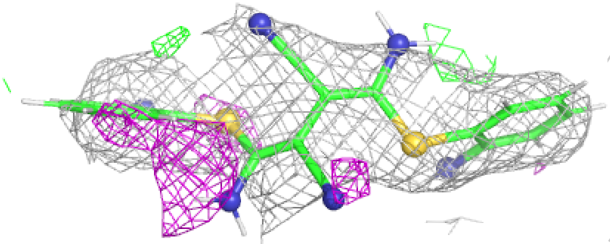

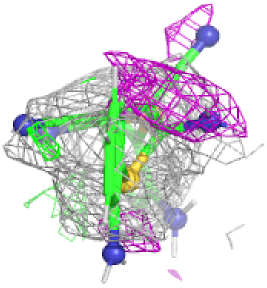

Electron density around 5BM E 403:

$2mF_o$-DF_c (at 0.7 rmsd) in gray
mF_o-DF_c (at 3 rmsd) in purple (negative)
and green (positive)

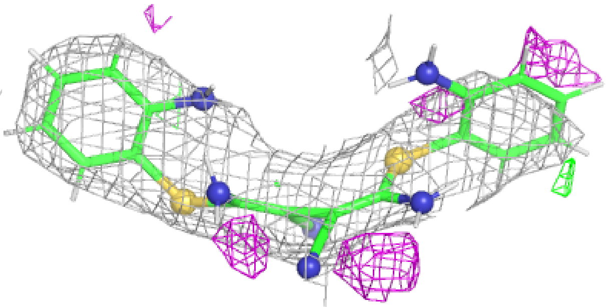

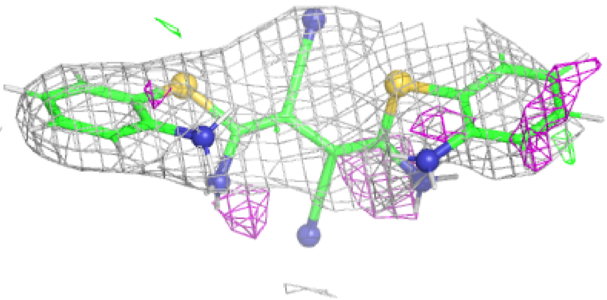

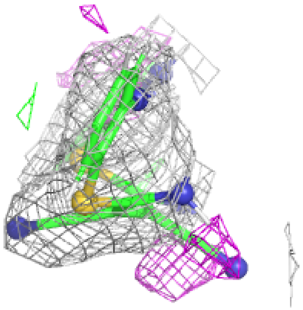

For Manuscript Review

Electron density around 5BM D 403:

$2mF_o$-DF_c (at 0.7 rmsd) in gray
mF_o-DF_c (at 3 rmsd) in purple (negative)
and green (positive)

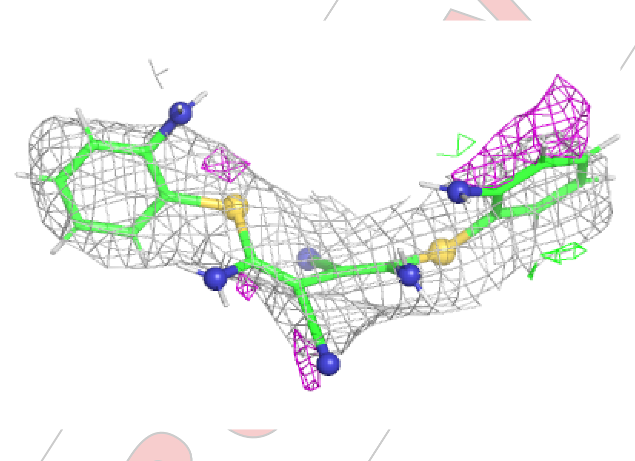

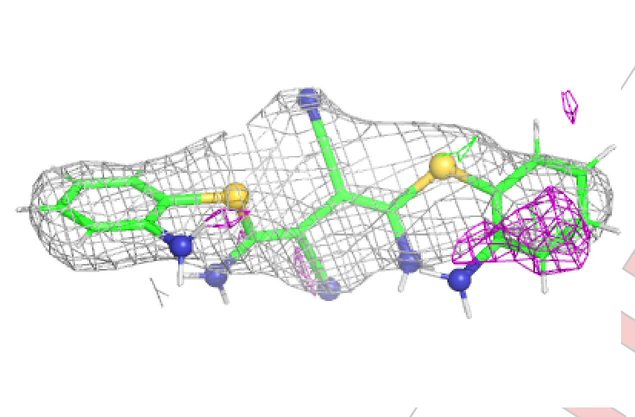

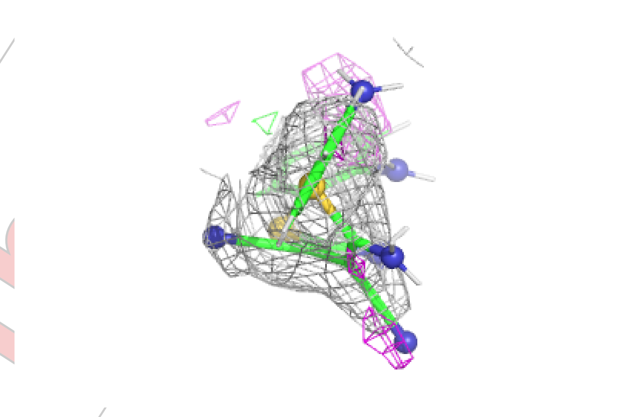

Electron density around 5BM F 403:

$2mF_o$-DF_c (at 0.7 rmsd) in gray
mF_o-DF_c (at 3 rmsd) in purple (negative)
and green (positive)

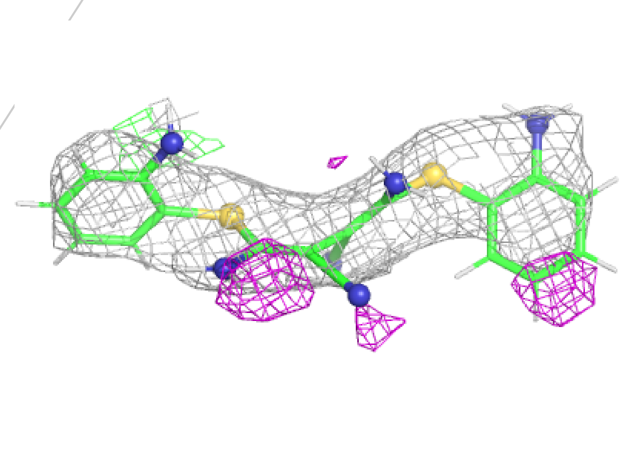

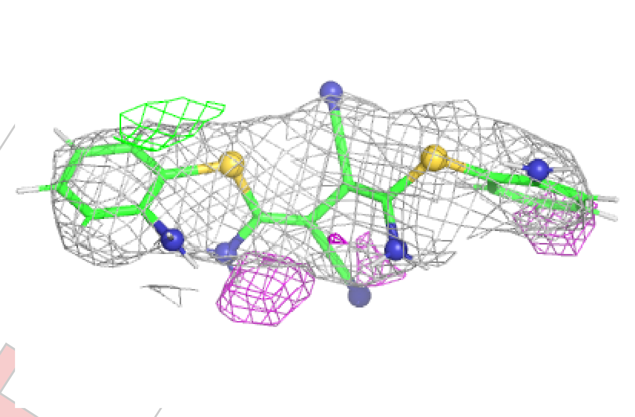

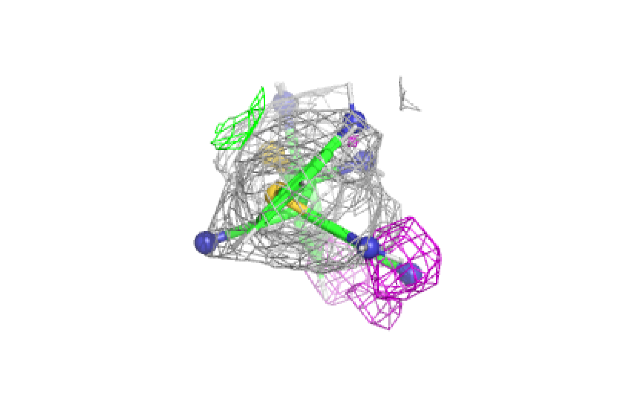

For Manuscript Review

Electron density around ANP B 402:

$2mF_o$-DF_c (at 0.7 rmsd) in gray
mF_o-DF_c (at 3 rmsd) in purple (negative)
and green (positive)

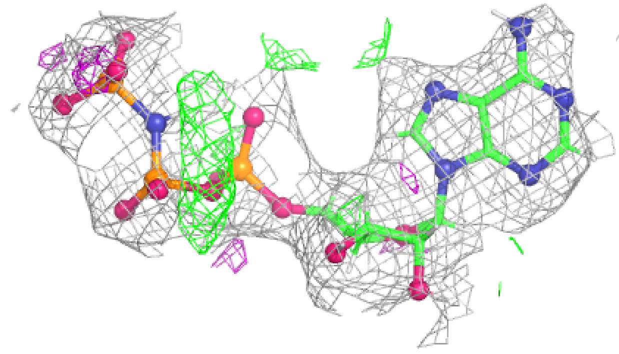

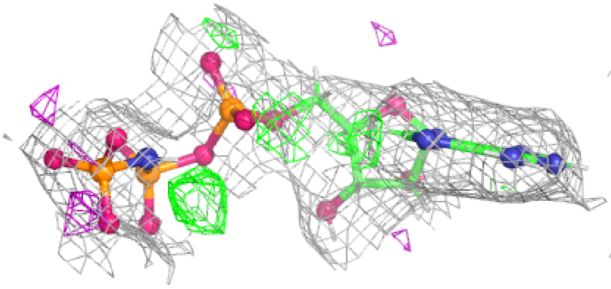

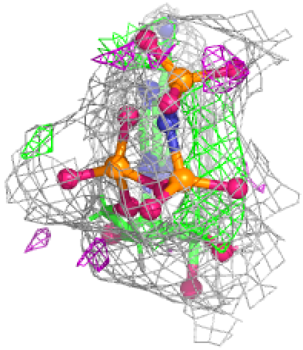

Electron density around ANP E 402:

$2mF_o$-DF_c (at 0.7 rmsd) in gray
mF_o-DF_c (at 3 rmsd) in purple (negative)
and green (positive)

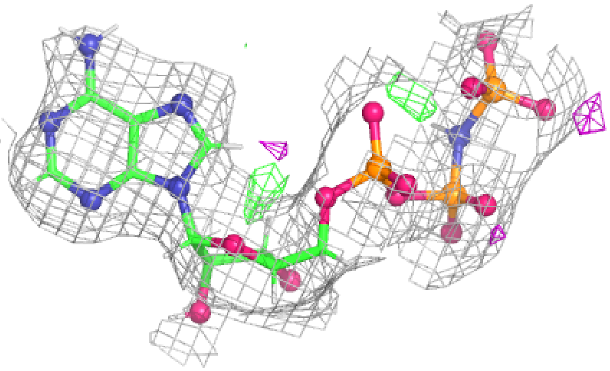

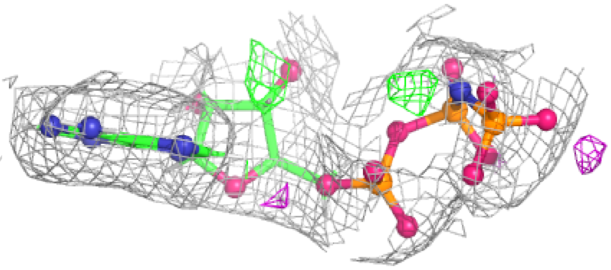

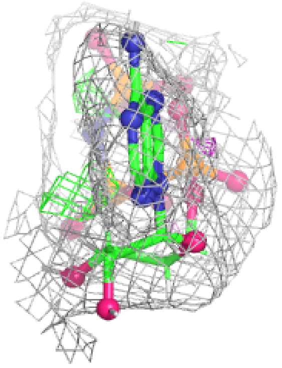

For Manuscript Review

Electron density around ANP F 402:

$2mF_o$-DF_c (at 0.7 rmsd) in gray
mF_o-DF_c (at 3 rmsd) in purple (negative)
and green (positive)

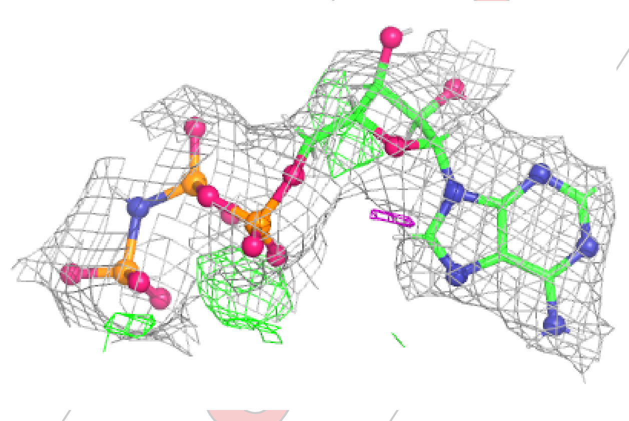

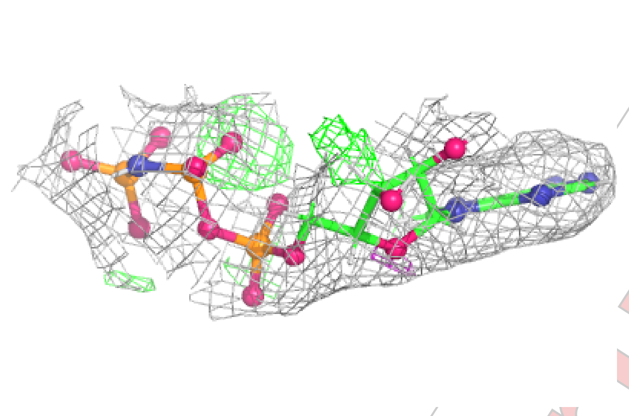

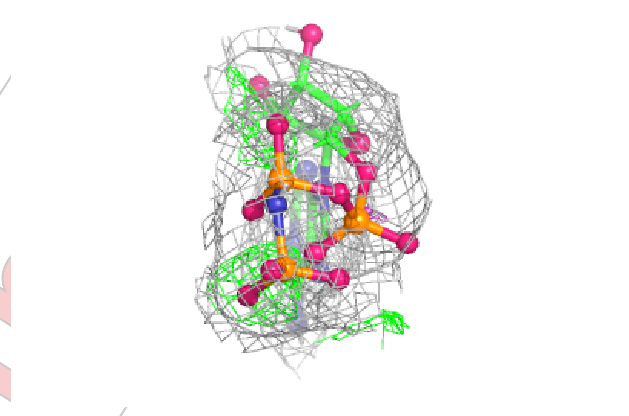

Electron density around ANP C 402:

$2mF_o$-DF_c (at 0.7 rmsd) in gray
mF_o-DF_c (at 3 rmsd) in purple (negative)
and green (positive)

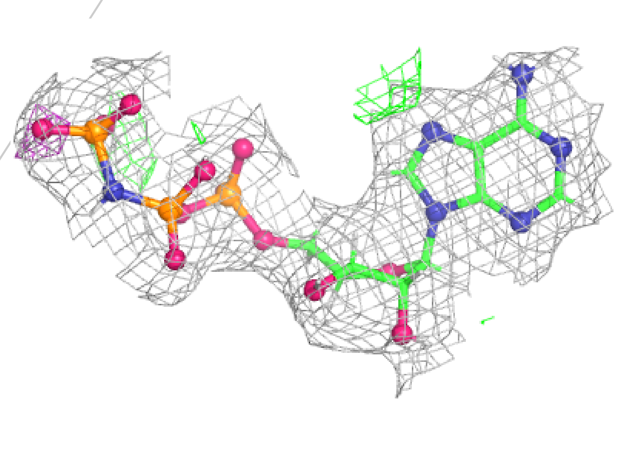

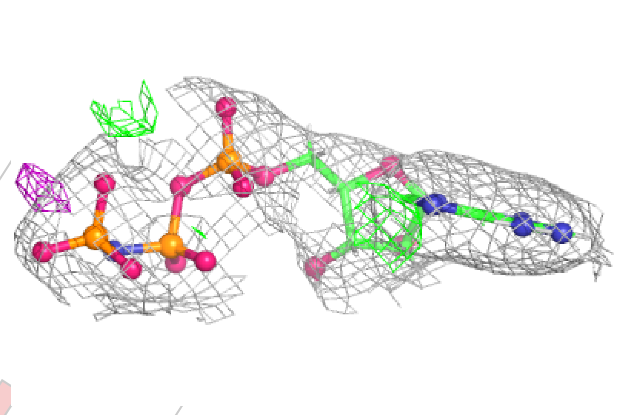

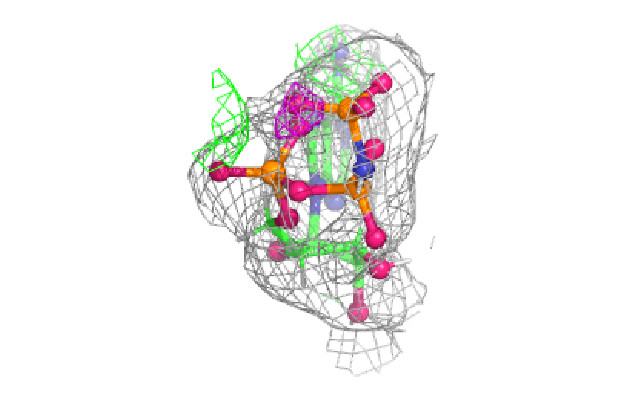

For Manuscript Review

Electron density around ANP D 402:

$2mF_o$-DF_c (at 0.7 rmsd) in gray
mF_o-DF_c (at 3 rmsd) in purple (negative)
and green (positive)

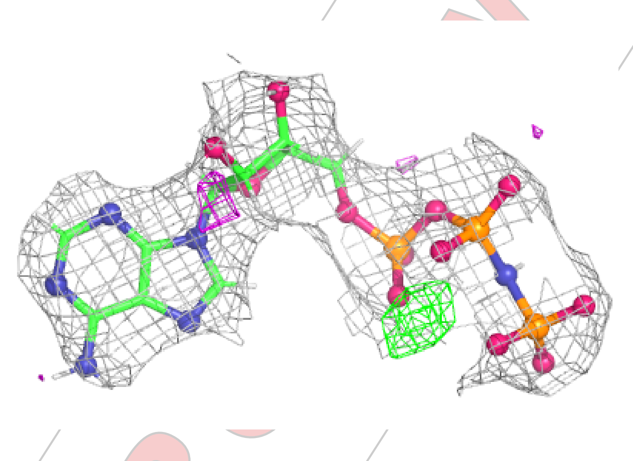

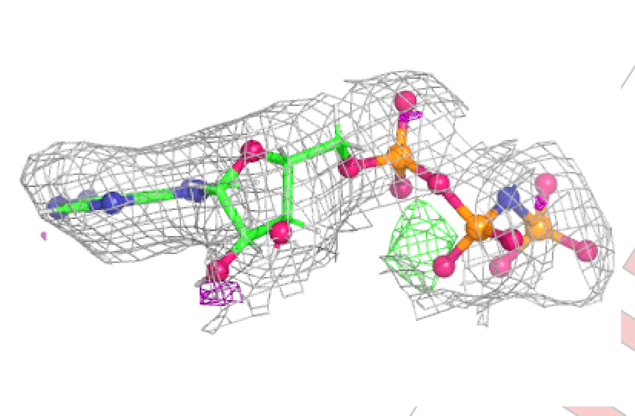

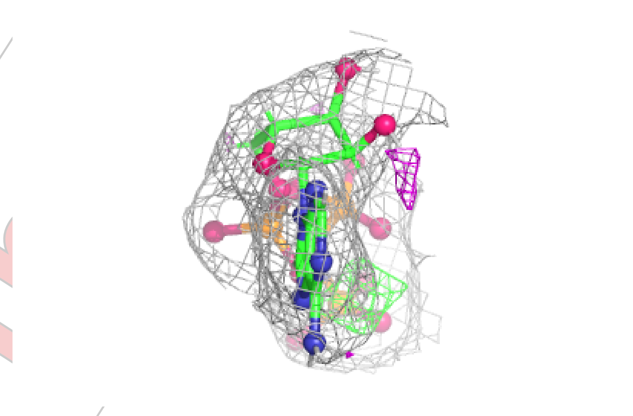

Electron density around ANP A 402:

$2mF_o$-DF_c (at 0.7 rmsd) in gray
mF_o-DF_c (at 3 rmsd) in purple (negative)
and green (positive)

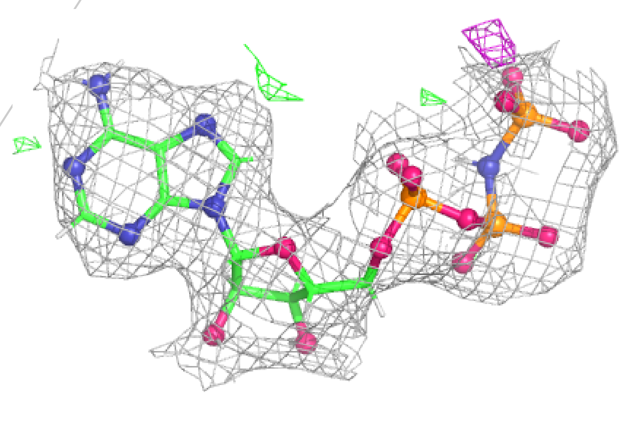

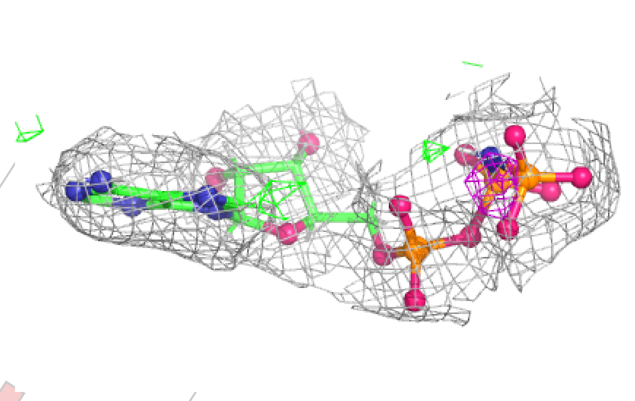

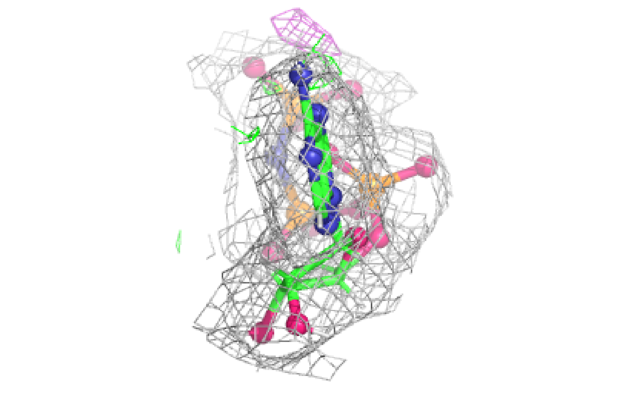

6.5 Other polymers (i)

There are no such residues in this entry.

For Manuscript Review
