# Supplementary material for: Structural and Biophysical Analyses of Human MEK2 in Complex with Two Inhibitors Reveal the Determinants of Isoform-Dependent Inhibitor Binding
Source: Int J Mol Sci. 2026 Jul 3;27(13):5992. doi: 10.3390/ijms27135992 (PMC13360787; doi:10.3390/ijms27135992)
Supplement: Supplementary file 1 [file ijms-27-05992-s001.zip › Supplementary_figs_revised_20260703.pdf]

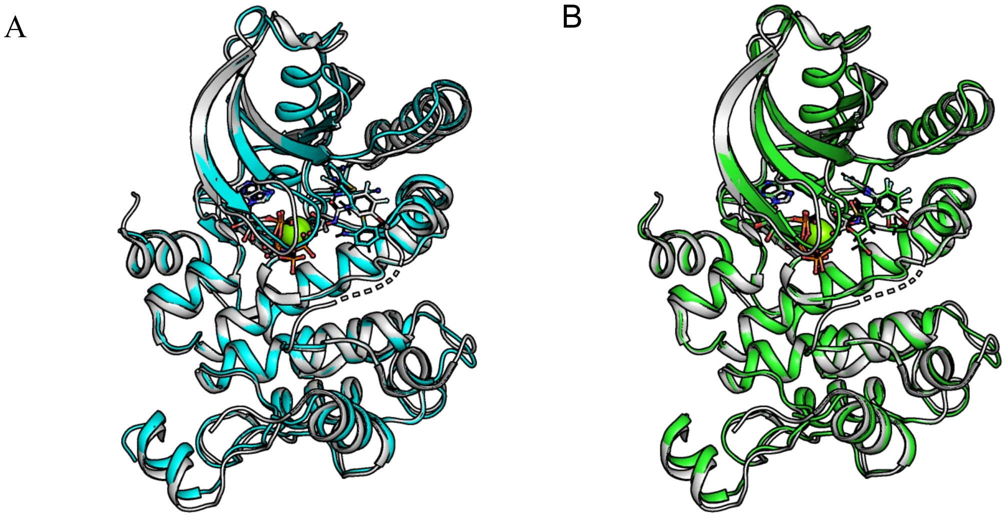


Figure S1. Structural comparison of the previously reported MEK2 structure (PDB ID: 1S9I) with the U0126- and refametinib-bound MEK2 structures determined in this study. (A) Overall structural alignment of the U0126-bound MEK2 structure with the previously reported MEK2 structure. The U0126-bound MEK2 is shown as a cyan cartoon, while the previously reported MEK2 structure is shown as a light gray cartoon. (B) Overall structural alignment of the refametinib-bound MEK2 structure with the previously reported MEK2 structure. The refametinib-bound MEK2 is shown as a green cartoon, while the previously reported MEK2 structure is shown as a light gray cartoon.

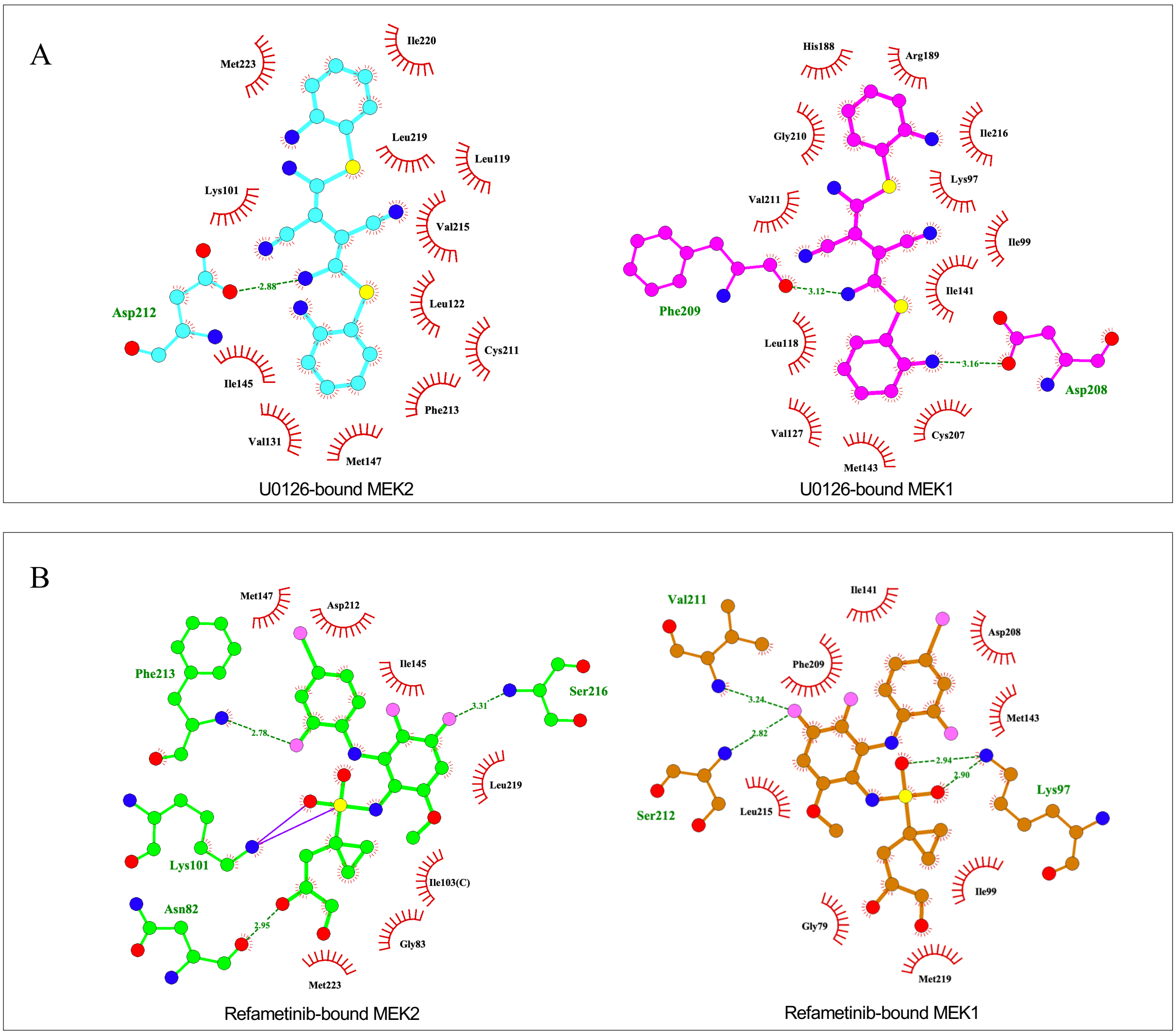


Figure S2. LigPlot-based comparison of residues involved in ligand interactions in MEK2 and MEK1 [32]. (A) Comparison of interaction residues in the U0126-bound MEK2 and U0126-bound MEK1 structures. MEK2 is shown as cyan sticks, and MEK1 is shown as magenta sticks. (B) Comparison of interaction residues in the refametinib-bound MEK2 and refametinib-bound MEK1 structures. MEK2 is shown as green sticks, and MEK1 is shown as orange sticks.

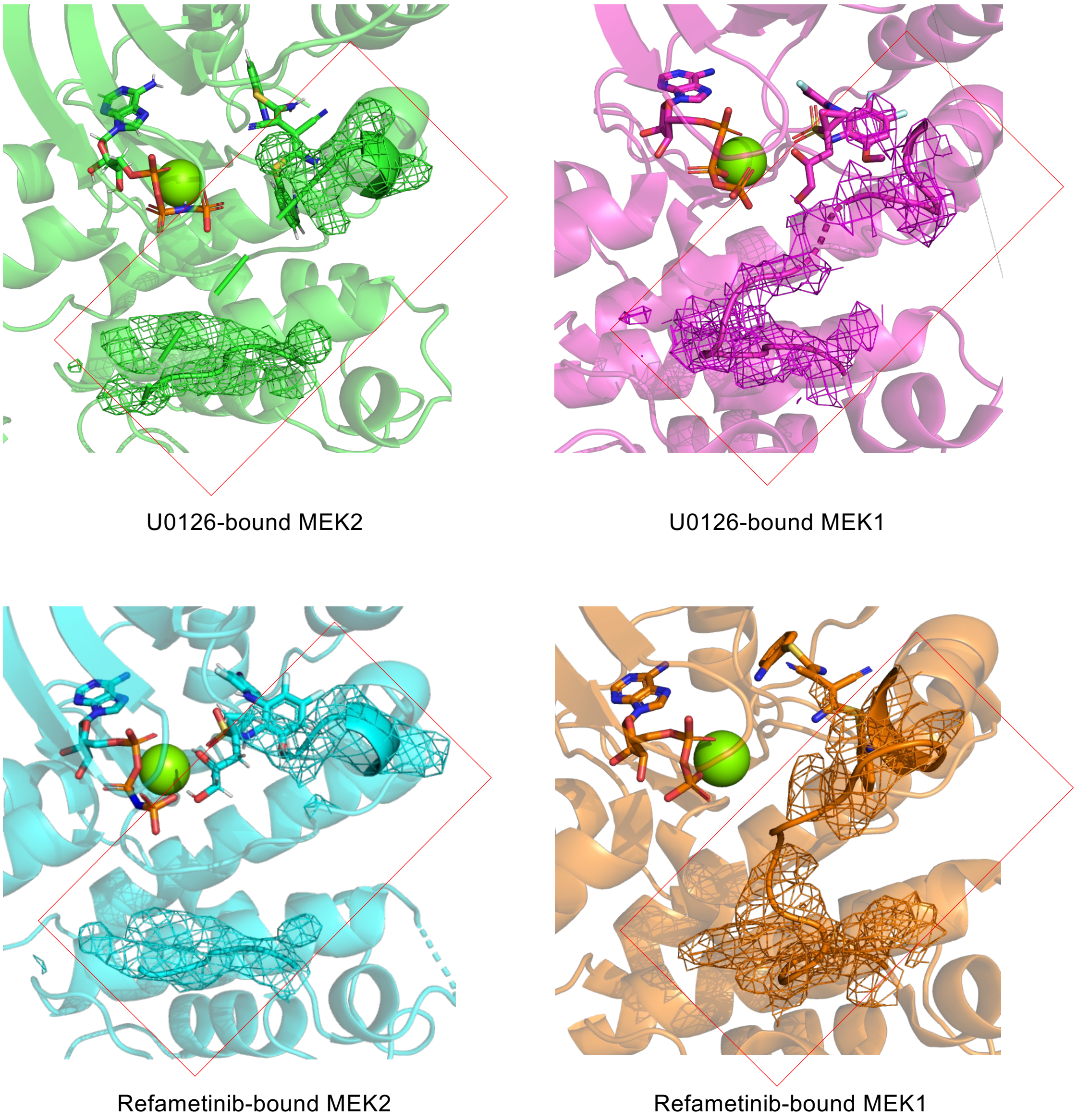


Figure S3. Comparison of activation loop electron density in the resolved MEK2 and MEK1 structures. The activation loop electron density is shown for the U0126- and refametinib-bound MEK2 and MEK1 structures. U0126-bound and refametinib-bound MEK2 are displayed as green and cyan cartoons, respectively, with the corresponding electron density maps shown as green and cyan meshes contoured at $\sigma$ = 1.0. U0126-bound and refametinib-bound MEK1 are displayed as magenta and orange cartoons, respectively, with the corresponding electron density maps shown as magenta and orange meshes contoured at $\sigma$ = 1.0.

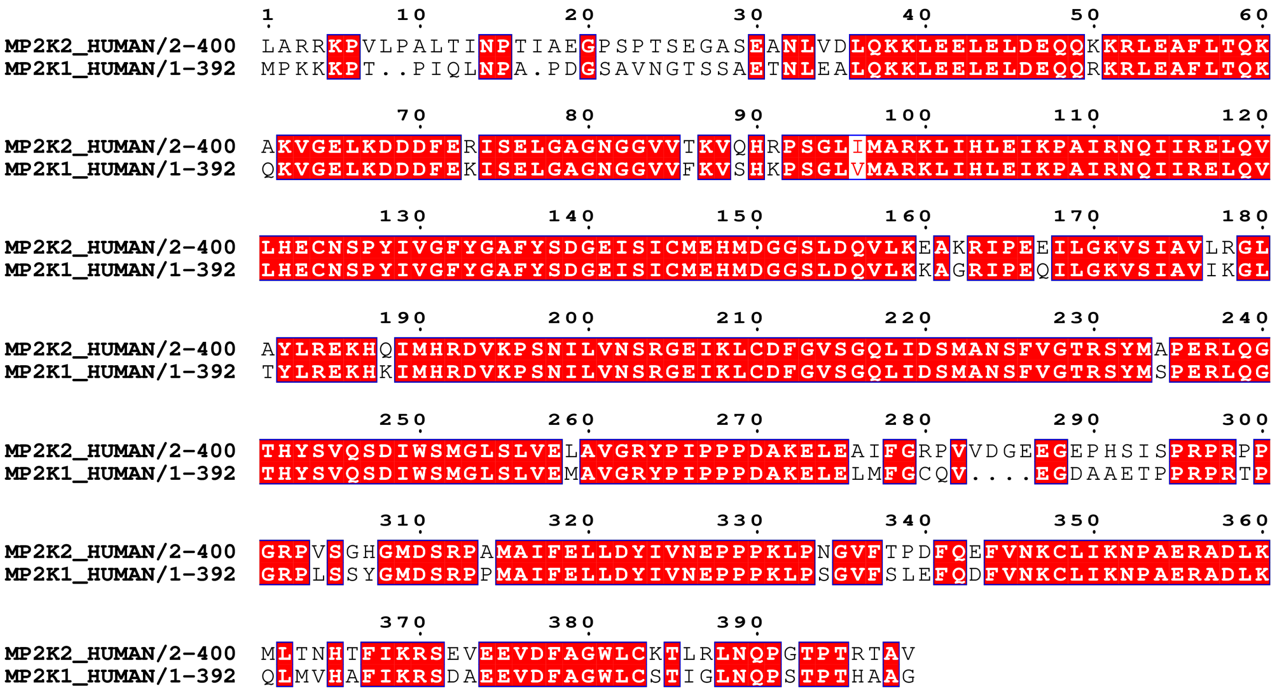


Figure S4. Full-length sequence alignment of MEK2 and MEK1 generated using Jalview [33]. The sequence alignment was performed using ClustalW. Identical residues are highlighted with red boxes, while non-identical residues are unboxed. The upper line corresponds to MEK2, and the lower line corresponds to MEK1.

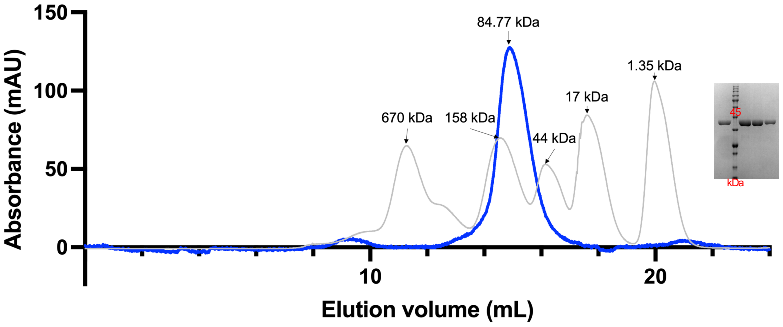


Figure S5. Size-exclusion chromatography analysis of human MEK1. The blue trace represents the A280 absorbance profile of purified human MEK1 (residues L37–Q383). The gray trace corresponds to molecular weight standards (Bio-Rad #1511901). The corresponding SDS-PAGE gel of the purified protein is shown on the right.

A

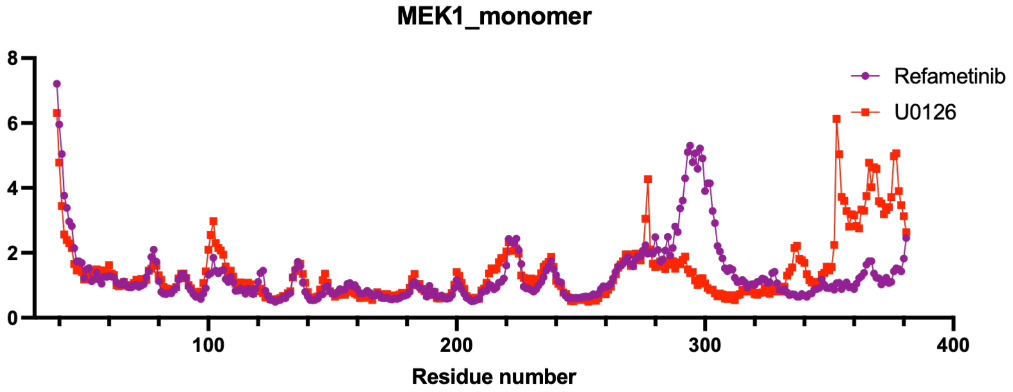


B

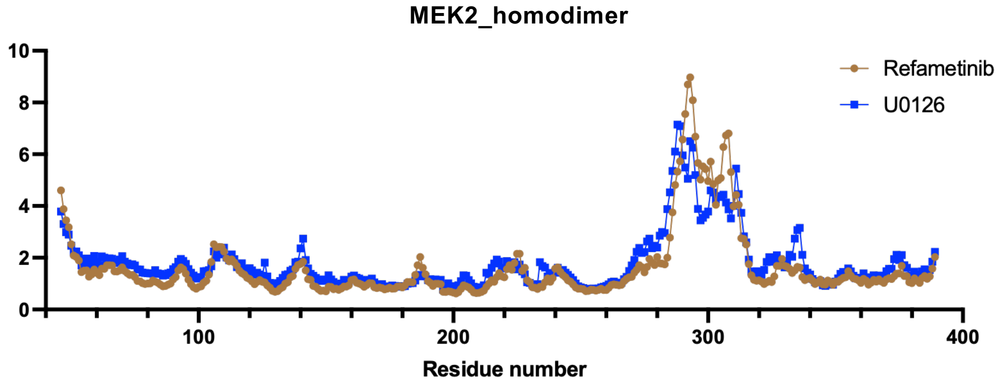


Figure S6. Molecular dynamics simulation analyses of homodimeric MEK2 and monomeric MEK1 in complex with refametinib and U0126. (A) RMSF profiles of monomeric MEK1 bound to refametinib or U0126. Refametinib is represented by a purple line with circular markers, whereas U0126 is represented by a red line with square markers. (B) RMSF profiles of homodimeric MEK2 bound to refametinib or U0126. Refametinib is represented by a brown line with circular markers, whereas U0126 is represented by a blue line with square markers.

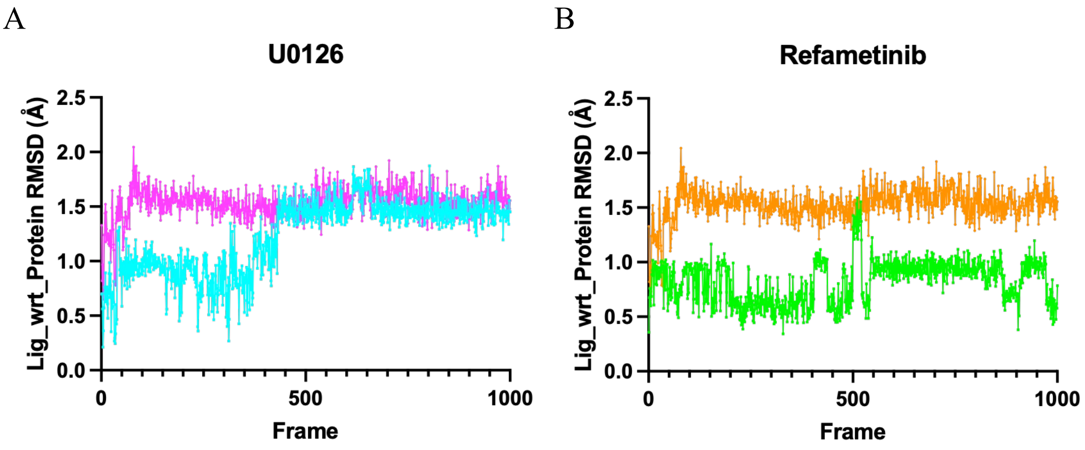


Figure S7. Ligand RMSD values relative to the protein during MD simulations of inhibitor-bound MEK1 and MEK2. Ligand RMSD values were calculated after alignment of the protein backbone. (A) U0126-bound complexes. MEK1 and MEK2 are shown in magenta and cyan, respectively. (B) Refametinib-bound complexes. MEK1 and MEK2 are shown in orange and green, respectively.
