# Supplementary material for: Structural and Biophysical Analyses of Human MEK2 in Complex with Two Inhibitors Reveal the Determinants of Isoform-Dependent Inhibitor Binding
Source: Int J Mol Sci. 2026 Jul 3;27(13):5992. doi: 10.3390/ijms27135992 (PMC13360787; doi:10.3390/ijms27135992)
Supplement: Supplementary file 1 [file ijms-27-05992-s001.zip › validation_report_21OA.pdf]

Full wwPDB X-ray Structure Validation Report (i)

Jun 19, 2026 – 04:58 PM JST

PDB ID	:	21OA / pdb_000021oa
Title	:	Crystal structure of human MEK2 in complex with Refametinib
Deposited on	:	2025-12-21
Resolution	:	3.30 Å(reported)

This wwPDB validation report is for manuscript review

This is a Full wwPDB X-ray Structure Validation Report.

This report is produced by the wwPDB biocuration pipeline after annotation of the structure.

We welcome your comments at *validation@mail.wwpdb.org*
A user guide is available at
https://www.wwpdb.org/validation/2017/XrayValidationReportHelp
with specific help available everywhere you see the (i) symbol.

The types of validation reports are described at
http://www.wwpdb.org/validation/2017/FAQs#types.

For Manuscript Review

The following versions of software and data (see references (i)) were used in the production of this report:

MolProbity	:	4-5-2 with Phenix2.0
Mogul	:	1.8.5 (274361), CSD as541be (2020)
Xtriage (Phenix)	:	2.0
EDS	:	3.0
Buster-report	:	wwPDB partial adaption of 1.1.7 (2018)
Percentile statistics	:	20250101.v01 (using entries in the PDB archive January 1st 2025)
CCP4	:	9.0.010 (Gargrove)
Density-Fitness	:	1.0.12
Ideal geometry (proteins)	:	Engh & Huber (2001)
Ideal geometry (DNA, RNA)	:	Parkinson et al. (1996)

1 Overall quality at a glance (i)

The following experimental techniques were used to determine the structure:
X-RAY DIFFRACTION

The reported resolution of this entry is 3.30 Å.

Percentile scores (ranging between 0-100) for global validation metrics of the entry are shown in the following graphic. The table shows the number of entries on which the scores are based.

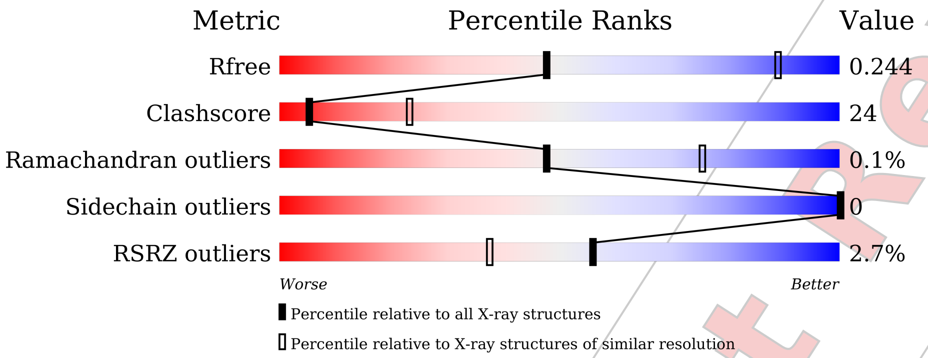

Metric	Whole archive (#Entries)	Similar resolution (#Entries, resolution range(Å))
R_{free}	180053	1169 (3.32-3.28)
Clashscore	190562	1209 (3.32-3.28)
Ramachandran outliers	187476	1188 (3.32-3.28)
Sidechain outliers	187428	1187 (3.32-3.28)
RSRZ outliers	180081	1169 (3.32-3.28)

The table below summarises the geometric issues observed across the polymeric chains and their fit to the electron density. The red, orange, yellow and green segments of the lower bar indicate the fraction of residues that contain outliers for >=3, 2, 1 and 0 types of geometric quality criteria respectively. A grey segment represents the fraction of residues that are not modelled. The numeric value for each fraction is indicated below the corresponding segment, with a dot representing fractions <=5% The upper red bar (where present) indicates the fraction of residues that have poor fit to the electron density. The numeric value is given above the bar.

Mol	Chain	Length	Quality of chain
1	A	346	% / 55% 34% 11%
1	B	346	2% / 51% 38% 11%
1	C	346	3% / 47% 40% 13%
1	D	346	3% / 49% 37% 13%

Continued on next page...

For Manuscript Review

Continued from previous page...

Mol	Chain	Length	Quality of chain
1	E	346	2% / 51% / 36% / 13%
1	F	346	4% / 47% / 40% / 13%

For Manuscript Review

2 Entry composition (i)

There are 5 unique types of molecules in this entry. The entry contains 15057 atoms, of which 198 are hydrogens and 0 are deuteriums.

In the tables below, the ZeroOcc column contains the number of atoms modelled with zero occupancy, the AltConf column contains the number of residues with at least one atom in alternate conformation and the Trace column contains the number of residues modelled with at most 2 atoms.

- Molecule 1 is a protein called Dual specificity mitogen-activated protein kinase kinase 2.

Mol	Chain	Residues	Atoms					ZeroOcc	AltConf	Trace
1	A	307	Total 2431	C 1547	N 420	O 450	S 14	0	0	0
1	B	307	Total 2430	C 1549	N 419	O 448	S 14	0	0	0
1	C	302	Total 2399	C 1530	N 414	O 441	S 14	0	0	0
1	D	300	Total 2382	C 1520	N 410	O 438	S 14	0	0	0
1	E	302	Total 2399	C 1530	N 414	O 441	S 14	0	0	0
1	F	302	Total 2399	C 1530	N 414	O 441	S 14	0	0	0

- Molecule 2 is MAGNESIUM ION (CCD ID: MG) (formula: Mg).

Mol	Chain	Residues	Atoms		ZeroOcc	AltConf
2	A	1	Total 1	Mg 1	0	0
2	B	1	Total 1	Mg 1	0	0
2	C	1	Total 1	Mg 1	0	0
2	D	1	Total 1	Mg 1	0	0
2	E	1	Total 1	Mg 1	0	0
2	F	1	Total 1	Mg 1	0	0

- Molecule 3 is N-{3,4-difluoro-2-[(2-fluoro-4-iodophenyl)amino]-6-methoxyphenyl}-1-[(2S)-2,3-dihydroxypropyl]cyclopropanesulfonamide (CCD ID: VRA) (formula: $C_{19}H_{20}F_3IN_2O_5S$) (labeled as "Ligand of Interest" by depositor).

For Manuscript Review

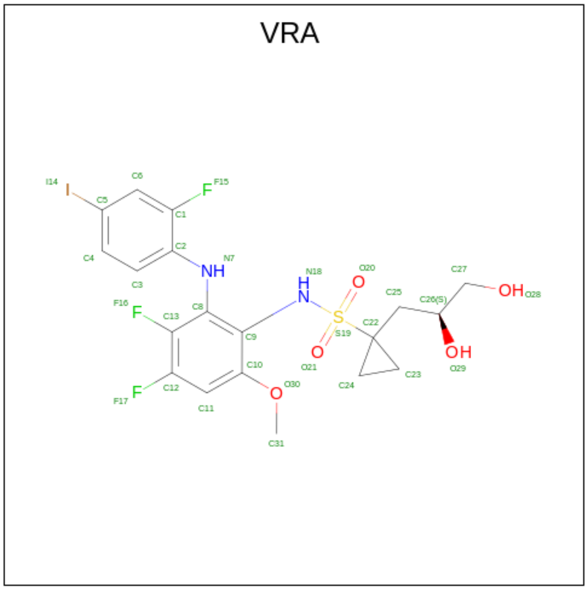

Mol	Chain	Residues	Atoms								ZeroOcc	AltConf
			Total	C	F	H	I	N	O	S		
3	A	1	51	19	3	20	1	2	5	1	0	0
3	B	1	51	19	3	20	1	2	5	1	0	0
3	C	1	51	19	3	20	1	2	5	1	0	0
3	D	1	51	19	3	20	1	2	5	1	0	0
3	E	1	51	19	3	20	1	2	5	1	0	0
3	F	1	51	19	3	20	1	2	5	1	0	0

- Molecule 4 is PHOSPHOAMINOPHOSPHONIC ACID-ADENYLATE ESTER (CCD ID: ANP) (formula: $C_{10}H_{17}N_6O_{12}P_3$) (labeled as "Ligand of Interest" by depositor).

For Manuscript Review

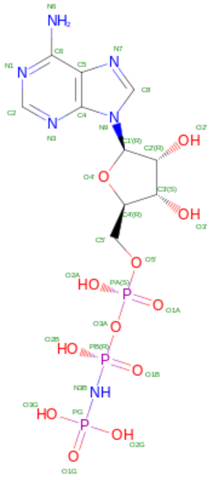

Mol	Chain	Residues	Atoms						ZeroOcc	AltConf
4	A	1	Total 44	C 10	H 13	N 6	O 12	P 3	0	0
4	B	1	Total 44	C 10	H 13	N 6	O 12	P 3	0	0
4	C	1	Total 44	C 10	H 13	N 6	O 12	P 3	0	0
4	D	1	Total 44	C 10	H 13	N 6	O 12	P 3	0	0
4	E	1	Total 44	C 10	H 13	N 6	O 12	P 3	0	0
4	F	1	Total 44	C 10	H 13	N 6	O 12	P 3	0	0

- Molecule 5 is water.

Mol	Chain	Residues	Atoms		ZeroOcc	AltConf
5	A	6	Total 6	O 6	0	0
5	B	5	Total 5	O 5	0	0
5	C	10	Total 10	O 10	0	0
5	D	3	Total 3	O 3	0	0
5	E	14	Total 14	O 14	0	0
5	F	3	Total 3	O 3	0	0

For Manuscript Review

3 Residue-property plots

These plots are drawn for all protein, RNA, DNA and oligosaccharide chains in the entry. The first graphic for a chain summarises the proportions of the various outlier classes displayed in the second graphic. The second graphic shows the sequence view annotated by issues in geometry and electron density. Residues are color-coded according to the number of geometric quality criteria for which they contain at least one outlier: green = 0, yellow = 1, orange = 2 and red = 3 or more. A red dot above a residue indicates a poor fit to the electron density (RSRZ > 2). Stretches of 2 or more consecutive residues without any outlier are shown as a green connector. Residues present in the sample, but not in the model, are shown in grey.

- Molecule 1: Dual specificity mitogen-activated protein kinase kinase 2

Chain A:

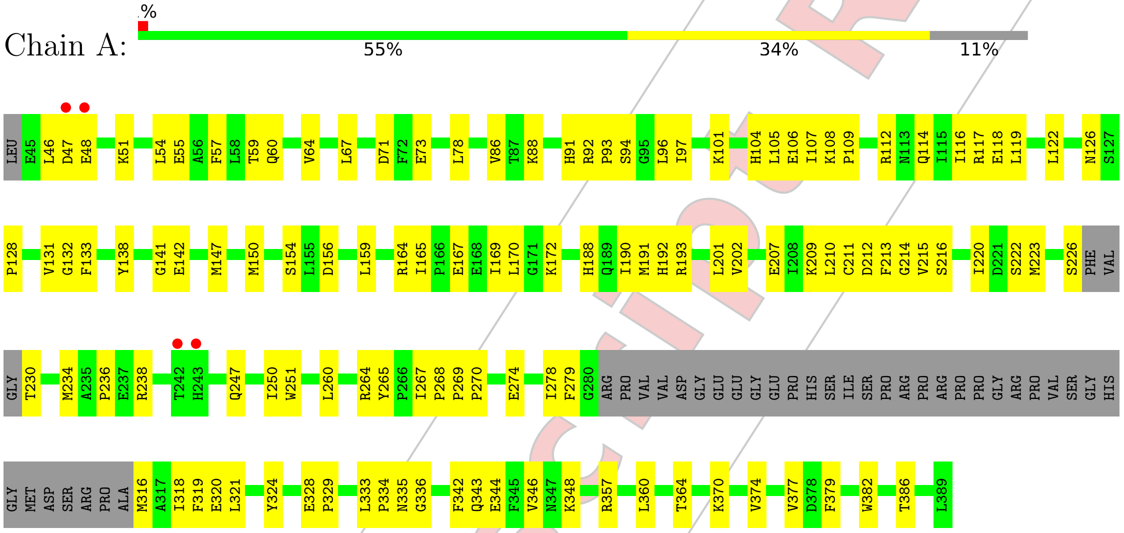

- Molecule 1: Dual specificity mitogen-activated protein kinase kinase 2

Chain B:

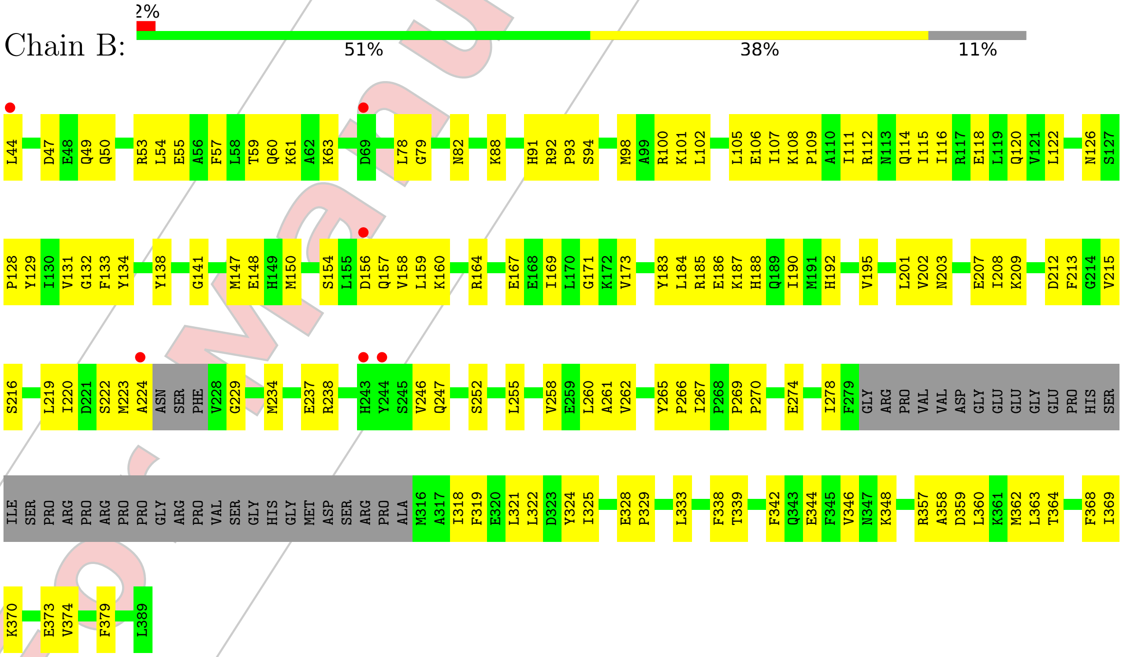

- Molecule 1: Dual specificity mitogen-activated protein kinase kinase 2

For Manuscript Review

Chain C:

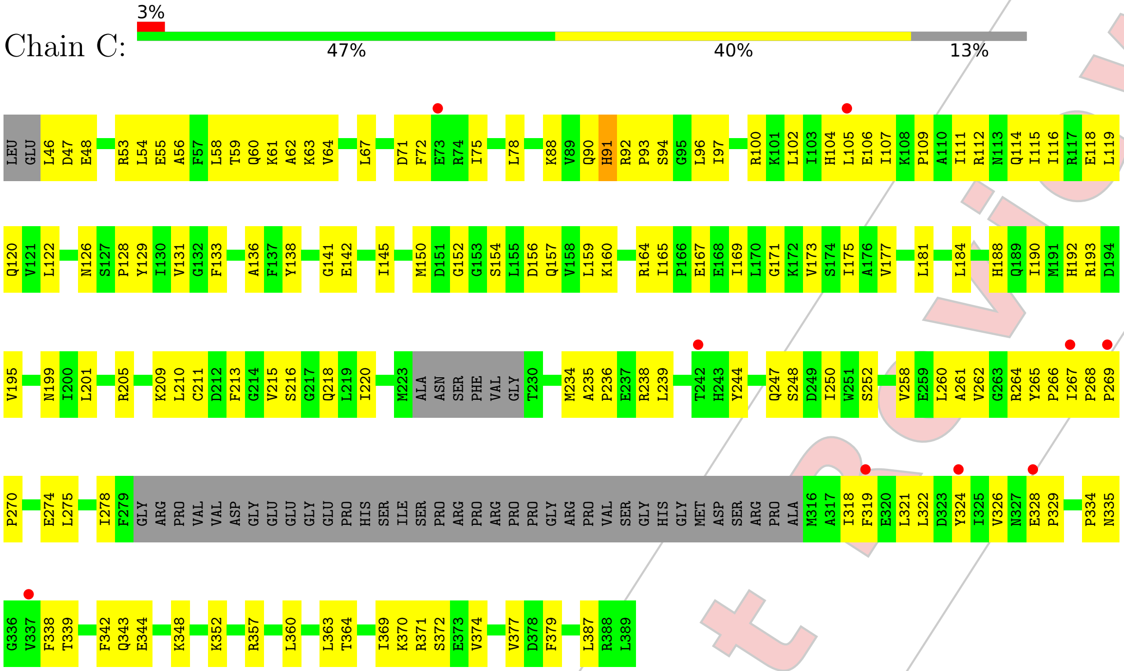

• Molecule 1: Dual specificity mitogen-activated protein kinase kinase 2

Chain D:

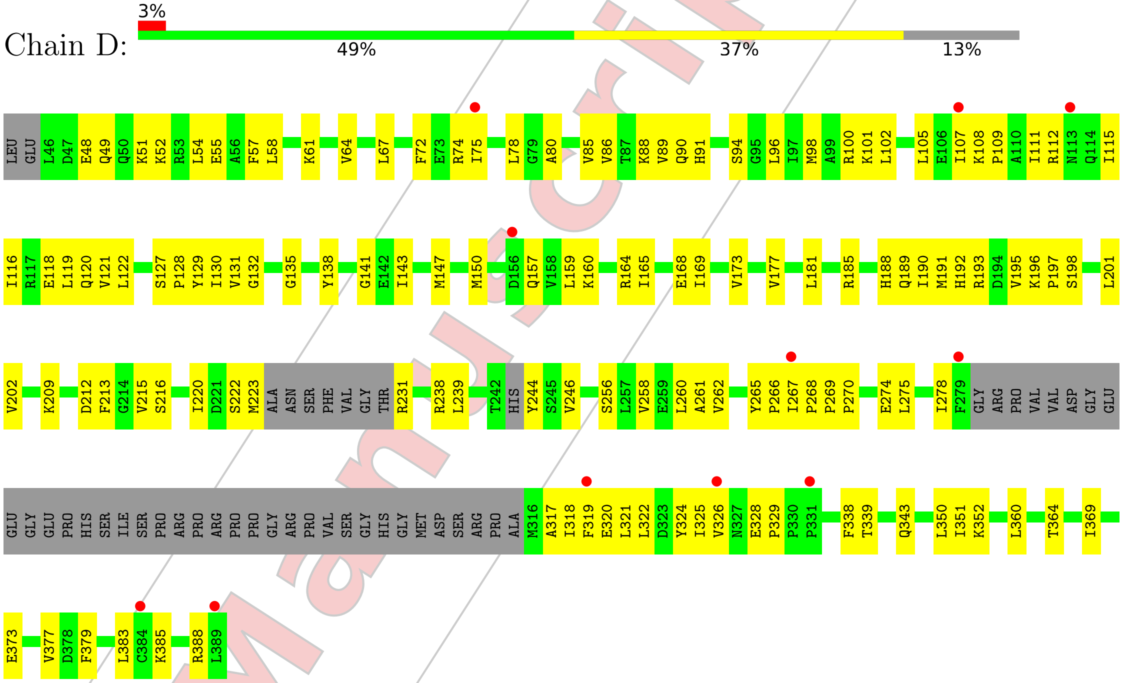

• Molecule 1: Dual specificity mitogen-activated protein kinase kinase 2

Chain E:

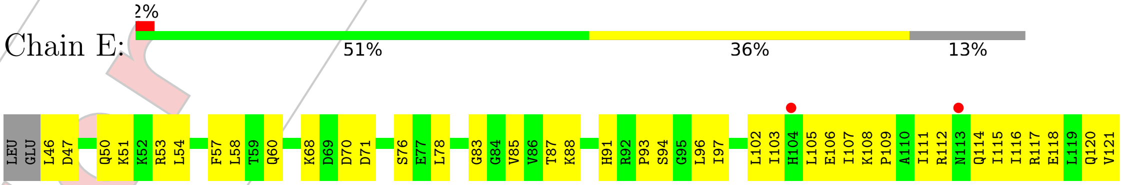

For Manuscript Review

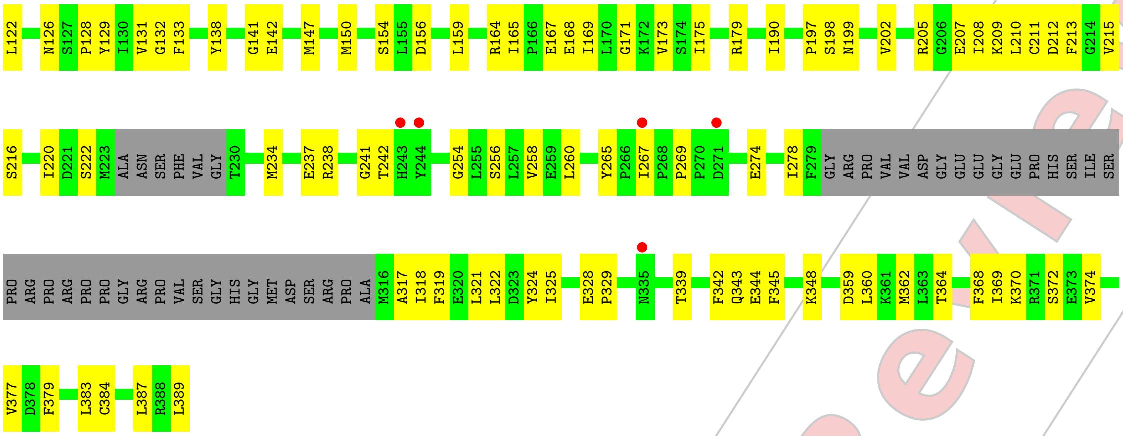

• Molecule 1: Dual specificity mitogen-activated protein kinase kinase 2

Chain F: 4% 47% 40% 13%

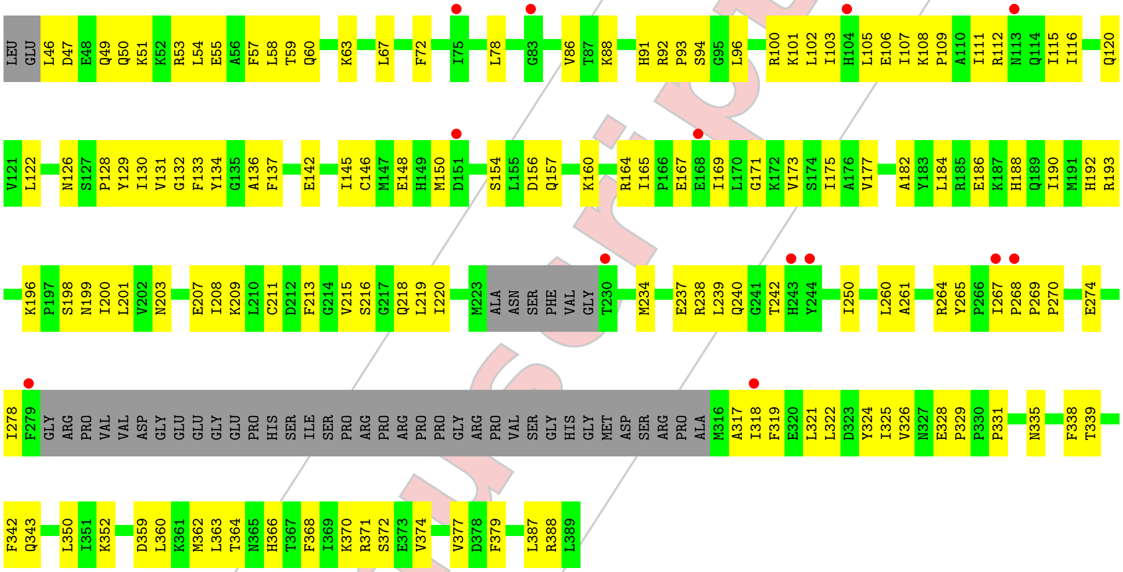

For Manuscript Review

4 Data and refinement statistics (i)

Property	Value	Source
Space group	P 21 21 2	Depositor
Cell constants<br>a, b, c, α, β, γ	149.35Å 261.67Å 75.37Å<br>90.00° 90.00° 90.00°	Depositor
Resolution (Å)	49.40 – 3.30<br>49.40 – 3.30	Depositor<br>EDS
% Data completeness<br>(in resolution range)	98.4 (49.40-3.30)<br>98.4 (49.40-3.30)	Depositor<br>EDS
R_{merge}	(Not available)	Depositor
R_{sym}	(Not available)	Depositor
$< I/\sigma(I) >$ [1]	2.84 (at 3.33Å)	Xtriage
Refinement program	PHENIX 2.0_5885	Depositor
R, R_{free}	0.208 , 0.242<br>0.210 , 0.244	Depositor<br>DCC
R_{free} test set	2000 reflections (4.42%)	wwPDB-VP
Wilson B-factor (Å^2)	61.3	Xtriage
Anisotropy	0.175	Xtriage
Bulk solvent k_{sol}(e/Å^3), B_{sol}(Å^2)	0.35 , 54.4	EDS
L-test for twinning[2]	$< \|L\| > = 0.49$, $< L^2 > = 0.32$	Xtriage
Estimated twinning fraction	No twinning to report.	Xtriage
F_o,F_c correlation	0.91	EDS
Total number of atoms	15057	wwPDB-VP
Average B, all atoms (Å^2)	56.0	wwPDB-VP

Xtriage's analysis on translational NCS is as follows: *The largest off-origin peak in the Patterson function is 11.73% of the height of the origin peak. No significant pseudotranslation is detected.*

[1] Intensities estimated from amplitudes.

[2] Theoretical values of $< |L| >$, $< L^2 >$ for acentric reflections are 0.5, 0.333 respectively for untwinned datasets, and 0.375, 0.2 for perfectly twinned datasets.

For Manuscript Review

5 Model quality (i)

5.1 Standard geometry (i)

Bond lengths and bond angles in the following residue types are not validated in this section: MG, VRA, ANP

The Z score for a bond length (or angle) is the number of standard deviations the observed value is removed from the expected value. A bond length (or angle) with $|Z| > 5$ is considered an outlier worth inspection. RMSZ is the root-mean-square of all Z scores of the bond lengths (or angles).

Mol	Chain	Bond lengths		Bond angles	
		RMSZ	#$\|Z\|$ >5	RMSZ	#$\|Z\|$ >5
1	A	0.18	0/2476	0.34	0/3336
1	B	0.18	0/2475	0.35	0/3335
1	C	0.18	0/2444	0.40	2/3293 (0.1%)
1	D	0.19	0/2425	0.35	0/3265
1	E	0.19	0/2444	0.36	0/3293
1	F	0.19	0/2444	0.36	0/3293
All	All	0.19	0/14708	0.36	2/19815 (0.0%)

There are no bond length outliers.

All (2) bond angle outliers are listed below:

Mol	Chain	Res	Type	Atoms	Z	Observed(^{o})	Ideal(^{o})
1	C	91	HIS	CA-C-N	6.67	128.05	119.78
1	C	91	HIS	C-N-CA	6.67	128.05	119.78

There are no chirality outliers.

There are no planarity outliers.

5.2 Too-close contacts (i)

In the following table, the Non-H and H(model) columns list the number of non-hydrogen atoms and hydrogen atoms in the chain respectively. The H(added) column lists the number of hydrogen atoms added and optimized by MolProbity. The Clashes column lists the number of clashes within the asymmetric unit, whereas Symm-Clashes lists symmetry-related clashes.

Mol	Chain	Non-H	H(model)	H(added)	Clashes	Symm-Clashes
1	A	2431	0	2462	99	0
1	B	2430	0	2464	118	0
1	C	2399	0	2437	127	0

Continued on next page...

For Manuscript Review

Continued from previous page...

Mol	Chain	Non-H	H(model)	H(added)	Clashes	Symm-Clashes
1	D	2382	0	2422	136	0
1	E	2399	0	2437	113	0
1	F	2399	0	2437	133	0
2	A	1	0	0	0	0
2	B	1	0	0	0	0
2	C	1	0	0	0	0
2	D	1	0	0	0	0
2	E	1	0	0	0	0
2	F	1	0	0	0	0
3	A	31	20	20	3	0
3	B	31	20	20	0	0
3	C	31	20	20	0	0
3	D	31	20	20	4	0
3	E	31	20	20	3	0
3	F	31	20	20	3	0
4	A	31	13	13	0	0
4	B	31	13	13	1	0
4	C	31	13	13	1	0
4	D	31	13	13	3	0
4	E	31	13	13	0	0
4	F	31	13	13	4	0
5	A	6	0	0	0	0
5	B	5	0	0	0	0
5	C	10	0	0	0	0
5	D	3	0	0	0	0
5	E	14	0	0	0	0
5	F	3	0	0	0	0
All	All	14859	198	14857	717	0

The all-atom clashscore is defined as the number of clashes found per 1000 atoms (including hydrogen atoms). The all-atom clashscore for this structure is 24.

All (717) close contacts within the same asymmetric unit are listed below, sorted by their clash magnitude.

Atom-1	Atom-2	Interatomic distance (Å)	Clash overlap (Å)
1:B:157:GLN:HA	1:B:160:LYS:HE2	1.38	1.04
1:D:157:GLN:HA	1:D:160:LYS:HE2	1.36	1.02
1:E:150:MET:HE1	1:E:209:LYS:HD2	1.37	1.00
1:D:267:ILE:HG22	1:D:268:PRO:HD3	1.46	0.94
1:F:150:MET:HE1	1:F:209:LYS:HD2	1.50	0.94
1:F:267:ILE:HG21	1:F:321:LEU:CD1	1.97	0.94

Continued on next page...

For Manuscript Review

Continued from previous page...

Atom-1	Atom-2	Interatomic distance (Å)	Clash overlap (Å)
1:C:267:ILE:HG21	1:C:321:LEU:HD12	1.49	0.94
1:C:238:ARG:HG2	1:C:244:TYR:HB3	1.51	0.93
1:B:150:MET:HE1	1:B:209:LYS:HD2	1.53	0.90
1:D:150:MET:HE1	1:D:209:LYS:HD2	1.54	0.89
1:D:267:ILE:CG2	1:D:268:PRO:HD3	2.02	0.89
1:E:267:ILE:HG21	1:E:321:LEU:HG	1.56	0.88
1:E:190:ILE:HD13	1:E:215:VAL:HG12	1.57	0.87
1:C:264:ARG:HH22	1:C:268:PRO:HG2	1.38	0.86
1:F:157:GLN:HA	1:F:160:LYS:HE2	1.59	0.84
1:A:165:ILE:HD12	1:A:260:LEU:HD22	1.59	0.84
1:E:384:CYS:HA	1:E:389:LEU:HD23	1.58	0.84
1:F:267:ILE:HG21	1:F:321:LEU:HD12	1.59	0.83
1:F:164:ARG:HD3	1:F:278:ILE:HG22	1.59	0.82
1:C:184:LEU:HD22	1:C:190:ILE:HD11	1.60	0.82
1:A:234:MET:CE	1:A:238:ARG:HB3	2.10	0.81
1:B:105:LEU:HG	1:B:107:ILE:HG23	1.62	0.80
1:E:54:LEU:HD11	1:E:126:ASN:HB3	1.64	0.80
1:B:328:GLU:HB3	1:B:329:PRO:HD2	1.62	0.79
1:B:173:VAL:HG22	1:B:208:ILE:HD13	1.63	0.79
1:C:150:MET:HE1	1:C:209:LYS:HD2	1.62	0.79
1:D:100:ARG:NE	1:D:102:LEU:HD21	1.96	0.79
1:A:335:ASN:HA	1:A:343:GLN:NE2	1.97	0.79
1:F:105:LEU:HG	1:F:107:ILE:HG23	1.63	0.79
1:A:335:ASN:HA	1:A:343:GLN:HE22	1.48	0.78
1:C:324:TYR:HA	1:C:328:GLU:HB2	1.63	0.78
1:B:138:TYR:OH	1:B:141:GLY:HA2	1.84	0.78
1:C:122:LEU:HD21	1:C:215:VAL:HG21	1.65	0.78
1:C:264:ARG:NH2	1:C:268:PRO:HG2	1.99	0.77
1:F:234:MET:CE	1:F:238:ARG:HB3	2.15	0.77
1:E:131:VAL:HG22	1:E:150:MET:HE3	1.65	0.77
1:E:105:LEU:HD21	1:E:222:SER:HB3	1.67	0.76
1:A:234:MET:HE2	1:A:238:ARG:HB3	1.66	0.76
1:E:138:TYR:OH	1:E:141:GLY:HA2	1.86	0.76
1:F:164:ARG:HD3	1:F:278:ILE:CG2	2.16	0.76
1:A:150:MET:HE1	1:A:209:LYS:HD2	1.69	0.75
1:E:173:VAL:HG22	1:E:208:ILE:HD13	1.68	0.75
1:D:100:ARG:HE	1:D:102:LEU:HD21	1.50	0.75
1:D:328:GLU:HB3	1:D:329:PRO:HD2	1.69	0.75
1:B:203:ASN:HD21	1:B:207:GLU:HB2	1.52	0.75
1:D:78:LEU:HD21	1:D:88:LYS:HB2	1.67	0.74
1:B:267:ILE:HD12	1:B:325:ILE:HG12	1.69	0.74

Continued on next page...

For Manuscript Review

Continued from previous page...

Atom-1	Atom-2	Interatomic distance (Å)	Clash overlap (Å)
1:B:105:LEU:HD23	1:B:107:ILE:HD13	1.68	0.74
1:C:150:MET:CE	1:C:209:LYS:HD2	2.17	0.74
1:D:164:ARG:HD3	1:D:278:ILE:HG22	1.70	0.73
1:D:107:ILE:HB	1:D:111:ILE:CD1	2.18	0.73
1:A:78:LEU:HD21	1:A:88:LYS:HB2	1.70	0.72
1:D:267:ILE:HG12	1:D:321:LEU:CD2	2.19	0.72
1:E:267:ILE:O	1:E:269:PRO:HD3	1.90	0.72
1:D:150:MET:CE	1:D:209:LYS:HD2	2.19	0.72
1:E:328:GLU:HB3	1:E:329:PRO:HD2	1.70	0.72
1:D:326:VAL:O	1:D:352:LYS:HE3	1.89	0.72
1:D:91:HIS:HB3	1:D:94:SER:OG	1.90	0.71
1:C:67:LEU:HD23	1:C:72:PHE:HZ	1.55	0.71
1:C:360:LEU:O	1:C:364:THR:HG23	1.90	0.71
1:C:267:ILE:HG21	1:C:321:LEU:CD1	2.19	0.70
1:F:54:LEU:HD11	1:F:126:ASN:HB3	1.72	0.70
1:B:78:LEU:HD21	1:B:88:LYS:HB2	1.72	0.70
1:C:111:ILE:HD12	1:C:218:GLN:NE2	2.06	0.70
1:C:159:LEU:HD22	1:C:260:LEU:HD23	1.74	0.70
1:D:80:ALA:HB2	1:D:85:VAL:HG23	1.74	0.70
1:C:193:ARG:HH12	1:C:244:TYR:HE2	1.40	0.70
1:E:190:ILE:CD1	1:E:215:VAL:HG12	2.22	0.69
1:F:150:MET:CE	1:F:209:LYS:HD2	2.22	0.69
1:B:150:MET:CE	1:B:209:LYS:HD2	2.21	0.69
1:C:175:ILE:HD11	1:C:372:SER:HB2	1.74	0.69
1:A:138:TYR:OH	1:A:141:GLY:HA2	1.92	0.69
1:B:234:MET:CE	1:B:238:ARG:HB3	2.23	0.69
1:D:196:LYS:HD2	1:D:198:SER:OG	1.93	0.68
1:E:234:MET:CE	1:E:238:ARG:HB3	2.23	0.68
1:A:268:PRO:HG3	1:A:316:MET:SD	2.33	0.68
1:E:269:PRO:HG2	1:E:324:TYR:OH	1.94	0.68
1:A:154:SER:HB2	1:A:156:ASP:OD1	1.92	0.68
1:C:131:VAL:HG22	1:C:150:MET:HE3	1.76	0.68
1:A:104:HIS:HA	1:A:142:GLU:HG2	1.74	0.68
1:D:112:ARG:O	1:D:116:ILE:HG12	1.94	0.67
1:E:150:MET:CE	1:E:209:LYS:HD2	2.19	0.67
1:A:226:SER:HB2	1:B:82:ASN:ND2	2.09	0.67
1:D:267:ILE:HG12	1:D:321:LEU:HD21	1.75	0.67
1:C:105:LEU:HG	1:C:107:ILE:HG23	1.76	0.67
1:A:78:LEU:HB2	1:A:86:VAL:HG12	1.75	0.67
1:D:48:GLU:HG3	1:D:49:GLN:H	1.60	0.67
1:C:75:ILE:HD11	1:C:90:GLN:HB2	1.76	0.66

Continued on next page...

For Manuscript Review

Continued from previous page...

Atom-1	Atom-2	Interatomic distance (Å)	Clash overlap (Å)
1:D:385:LYS:O	1:D:388:ARG:HD2	1.95	0.66
1:B:167:GLU:OE2	1:B:342:PHE:HB2	1.95	0.66
1:A:165:ILE:CD1	1:A:260:LEU:HD22	2.26	0.66
1:D:54:LEU:O	1:D:58:LEU:HD13	1.95	0.66
1:A:114:GLN:HG3	1:A:117:ARG:NH2	2.10	0.66
1:B:107:ILE:HB	1:B:111:ILE:CD1	2.25	0.66
1:B:107:ILE:HB	1:B:111:ILE:HD11	1.78	0.66
1:D:105:LEU:HD21	1:D:222:SER:HB3	1.76	0.66
1:C:169:ILE:HG23	1:C:379:PHE:HD1	1.59	0.66
1:F:173:VAL:HG22	1:F:208:ILE:HD13	1.78	0.66
1:E:112:ARG:O	1:E:116:ILE:HG12	1.95	0.66
1:B:265:TYR:CE2	1:B:267:ILE:HB	2.30	0.65
1:F:328:GLU:HB3	1:F:329:PRO:HD2	1.76	0.65
1:E:175:ILE:HD11	1:E:372:SER:HB2	1.78	0.65
1:A:230:THR:O	1:A:321:LEU:HD22	1.95	0.65
1:D:267:ILE:HD11	1:D:325:ILE:HG12	1.79	0.65
1:C:261:ALA:HB1	1:C:338:PHE:CZ	2.32	0.65
1:D:54:LEU:HD12	1:D:55:GLU:N	2.12	0.65
1:D:192:HIS:CD2	1:D:213:PHE:HB3	2.31	0.65
1:B:105:LEU:HG	1:B:107:ILE:CG2	2.27	0.65
1:F:133:PHE:HE1	1:F:145:ILE:HG23	1.61	0.65
1:A:344:GLU:O	1:A:348:LYS:HG3	1.97	0.65
1:F:234:MET:HE2	1:F:238:ARG:HB3	1.78	0.65
1:A:150:MET:CE	1:A:209:LYS:HD2	2.27	0.65
1:D:269:PRO:HG3	1:D:324:TYR:OH	1.97	0.65
1:F:131:VAL:HG22	1:F:150:MET:HE3	1.78	0.65
1:C:247:GLN:HB3	1:C:357:ARG:HH11	1.62	0.64
1:F:86:VAL:HB	4:F:401:ANP:H5'2	1.79	0.64
1:E:154:SER:HB2	1:E:156:ASP:OD1	1.97	0.64
1:A:71:ASP:HB3	1:A:93:PRO:CD	2.27	0.64
1:D:231:ARG:HA	1:D:265:TYR:CE2	2.32	0.64
1:F:100:ARG:NE	1:F:102:LEU:HD21	2.11	0.64
1:B:122:LEU:CD2	1:B:215:VAL:HG11	2.27	0.64
1:D:107:ILE:HB	1:D:111:ILE:HG13	1.79	0.64
1:B:274:GLU:O	1:B:278:ILE:HG13	1.98	0.64
1:D:108:LYS:H	1:D:111:ILE:HD11	1.62	0.64
1:E:370:LYS:O	1:E:374:VAL:HG13	1.99	0.63
1:F:234:MET:HE3	1:F:238:ARG:NE	2.13	0.63
1:D:159:LEU:HD22	1:D:260:LEU:HD23	1.80	0.63
1:F:196:LYS:HE2	1:F:198:SER:OG	1.99	0.63
1:C:114:GLN:O	1:C:118:GLU:HG3	1.98	0.63

Continued on next page...

For Manuscript Review

Continued from previous page...

Atom-1	Atom-2	Interatomic distance (Å)	Clash overlap (Å)
1:A:150:MET:HE1	1:A:209:LYS:CD	2.28	0.63
1:A:226:SER:HB2	1:B:82:ASN:HD21	1.63	0.63
1:B:164:ARG:HD3	1:B:278:ILE:CG2	2.29	0.62
1:C:46:LEU:CD1	1:C:128:PRO:HG3	2.28	0.62
1:E:154:SER:HB3	1:E:198:SER:HA	1.81	0.62
1:D:197:PRO:HG3	1:D:256:SER:HB3	1.80	0.62
1:F:107:ILE:HB	1:F:111:ILE:HG13	1.80	0.62
1:C:322:LEU:HD23	1:D:319:PHE:HB2	1.80	0.62
1:D:324:TYR:O	1:D:328:GLU:HB2	2.00	0.62
1:F:267:ILE:HG21	1:F:321:LEU:HD13	1.80	0.62
1:F:91:HIS:HB3	1:F:94:SER:OG	2.00	0.62
1:F:105:LEU:HD23	1:F:107:ILE:HD13	1.81	0.62
1:F:128:PRO:HG2	1:F:129:TYR:CE2	2.34	0.62
1:C:154:SER:HA	1:C:201:LEU:HD23	1.81	0.62
1:E:105:LEU:HG	1:E:107:ILE:HG23	1.81	0.62
1:F:274:GLU:O	1:F:278:ILE:HG12	1.99	0.62
1:F:203:ASN:HD21	1:F:207:GLU:HB2	1.65	0.62
1:C:55:GLU:O	1:C:59:THR:HG23	2.00	0.62
1:D:128:PRO:HG2	1:D:129:TYR:CE2	2.35	0.61
1:F:100:ARG:HE	1:F:102:LEU:HD21	1.65	0.61
1:F:133:PHE:HZ	1:F:136:ALA:HB2	1.65	0.61
1:B:122:LEU:HD21	1:B:215:VAL:HG11	1.82	0.61
1:F:60:GLN:NE2	1:F:96:LEU:HD21	2.14	0.61
1:B:131:VAL:HG22	1:B:150:MET:HE3	1.82	0.61
1:D:188:HIS:O	1:D:190:ILE:HG23	2.01	0.61
1:E:322:LEU:HB3	1:F:319:PHE:HD1	1.65	0.61
1:A:105:LEU:HG	1:A:107:ILE:HG23	1.83	0.61
1:A:131:VAL:HG23	1:A:210:LEU:O	1.99	0.61
1:B:107:ILE:HB	1:B:111:ILE:HG13	1.83	0.61
1:E:47:ASP:HB2	1:E:50:GLN:HG3	1.82	0.61
1:E:265:TYR:HE2	1:E:321:LEU:HD11	1.65	0.61
1:A:105:LEU:HD21	1:A:222:SER:HB3	1.83	0.60
1:F:51:LYS:O	1:F:55:GLU:HG3	2.01	0.60
1:B:203:ASN:ND2	1:B:207:GLU:HB2	2.16	0.60
1:C:322:LEU:HB3	1:D:319:PHE:HD1	1.66	0.60
1:D:75:ILE:HD11	1:D:90:GLN:HB2	1.82	0.60
1:D:318:ILE:O	1:D:322:LEU:HD13	2.00	0.60
1:B:157:GLN:HA	1:B:160:LYS:CE	2.25	0.60
1:D:169:ILE:HG23	1:D:379:PHE:HD1	1.65	0.60
1:F:318:ILE:O	1:F:322:LEU:HD13	2.02	0.60
1:D:80:ALA:CB	1:D:85:VAL:HG23	2.31	0.60

Continued on next page...

For Manuscript Review

Continued from previous page...

Atom-1	Atom-2	Interatomic distance (Å)	Clash overlap (Å)
1:F:157:GLN:HA	1:F:160:LYS:CE	2.29	0.60
1:B:234:MET:HE3	1:B:238:ARG:HB3	1.84	0.60
1:B:369:ILE:O	1:B:373:GLU:HG3	2.01	0.60
1:E:68:LYS:HG2	1:E:71:ASP:OD2	2.02	0.60
1:F:265:TYR:CE2	1:F:267:ILE:HB	2.37	0.60
1:A:328:GLU:HB3	1:A:329:PRO:HD2	1.84	0.59
1:E:344:GLU:O	1:E:348:LYS:HG3	2.02	0.59
1:F:46:LEU:HD12	1:F:50:GLN:OE1	2.02	0.59
1:F:67:LEU:HD23	1:F:72:PHE:HZ	1.67	0.59
1:B:91:HIS:HB3	1:B:94:SER:OG	2.02	0.59
1:E:167:GLU:OE1	1:E:339:THR:HG23	2.01	0.59
1:F:216:SER:O	1:F:220:ILE:HG13	2.01	0.59
1:D:339:THR:O	1:D:343:GLN:HG3	2.02	0.59
1:B:55:GLU:O	1:B:59:THR:HG23	2.03	0.59
1:C:61:LYS:HA	1:C:64:VAL:HG12	1.84	0.59
1:F:261:ALA:HB1	1:F:338:PHE:CZ	2.37	0.59
1:B:185:ARG:HH11	1:B:246:VAL:HG21	1.67	0.59
1:D:51:LYS:O	1:D:54:LEU:HG	2.03	0.59
1:C:328:GLU:HB3	1:C:329:PRO:HD2	1.84	0.59
1:D:48:GLU:HG3	1:D:49:GLN:N	2.18	0.59
1:D:61:LYS:O	1:D:64:VAL:HG22	2.02	0.59
1:C:250:ILE:HD12	1:C:363:LEU:HD12	1.84	0.59
1:F:264:ARG:HH22	1:F:268:PRO:HB2	1.68	0.59
1:C:61:LYS:HE2	1:C:133:PHE:O	2.02	0.59
1:D:78:LEU:CD2	1:D:88:LYS:HB2	2.32	0.59
1:E:318:ILE:O	1:E:322:LEU:HD13	2.02	0.59
1:D:169:ILE:HG23	1:D:379:PHE:CD1	2.38	0.58
1:E:106:GLU:HG3	1:E:106:GLU:O	2.01	0.58
1:E:108:LYS:HB3	1:E:109:PRO:HD2	1.85	0.58
1:F:57:PHE:CZ	1:F:132:GLY:HA3	2.37	0.58
1:F:105:LEU:HG	1:F:107:ILE:CG2	2.32	0.58
1:C:269:PRO:HG3	1:C:324:TYR:OH	2.02	0.58
1:F:47:ASP:HB2	1:F:50:GLN:HG3	1.85	0.58
1:C:46:LEU:HD11	1:C:128:PRO:HG3	1.85	0.58
1:D:122:LEU:HD21	1:D:215:VAL:HG11	1.86	0.58
1:F:267:ILE:HD12	1:F:325:ILE:HG12	1.85	0.58
1:D:122:LEU:CD2	1:D:215:VAL:HG11	2.34	0.58
1:F:387:LEU:O	1:F:388:ARG:HB2	2.04	0.58
1:A:269:PRO:HG3	1:A:324:TYR:OH	2.04	0.58
1:B:107:ILE:HB	1:B:111:ILE:CG1	2.33	0.58
1:E:116:ILE:O	1:E:120:GLN:HG2	2.04	0.57

Continued on next page...

For Manuscript Review

Continued from previous page...

Atom-1	Atom-2	Interatomic distance (Å)	Clash overlap (Å)
1:D:138:TYR:OH	1:D:141:GLY:HA2	2.04	0.57
1:A:94:SER:HB2	1:A:96:LEU:HG	1.85	0.57
1:C:238:ARG:CG	1:C:244:TYR:HB3	2.31	0.57
1:B:192:HIS:ND1	1:B:213:PHE:HB3	2.18	0.57
1:B:258:VAL:O	1:B:262:VAL:HG22	2.04	0.57
1:B:267:ILE:O	1:B:269:PRO:HD3	2.03	0.57
1:B:267:ILE:CD1	1:B:325:ILE:HG12	2.35	0.57
1:D:107:ILE:HB	1:D:111:ILE:CG1	2.35	0.56
1:D:212:ASP:HA	3:D:402:VRA:C3	2.34	0.56
1:F:46:LEU:CD1	1:F:128:PRO:HG3	2.35	0.56
1:F:154:SER:OG	4:F:401:ANP:H2’	2.04	0.56
1:B:171:GLY:HA2	1:B:368:PHE:CE2	2.40	0.56
1:E:167:GLU:CD	1:E:339:THR:HG23	2.30	0.56
3:E:402:VRA:C9	3:E:402:VRA:H24	2.36	0.56
1:D:78:LEU:HD13	4:D:401:ANP:C2	2.36	0.56
1:F:150:MET:HG3	1:F:201:LEU:HB3	1.87	0.56
1:A:214:GLY:HA3	1:A:220:ILE:HD11	1.88	0.56
1:B:164:ARG:HD3	1:B:278:ILE:HG22	1.86	0.56
1:F:46:LEU:HG	1:F:50:GLN:HB2	1.88	0.56
1:B:49:GLN:O	1:B:53:ARG:HG3	2.05	0.56
1:B:247:GLN:HB3	1:B:357:ARG:HH11	1.69	0.56
1:F:111:ILE:O	1:F:115:ILE:HG13	2.06	0.56
1:F:371:ARG:HA	1:F:374:VAL:HG22	1.87	0.56
1:C:106:GLU:O	1:C:106:GLU:HG2	2.06	0.56
1:E:324:TYR:O	1:E:328:GLU:HB2	2.05	0.56
1:B:54:LEU:HD11	1:B:126:ASN:HB3	1.87	0.55
1:B:111:ILE:O	1:B:115:ILE:HG13	2.06	0.55
1:F:269:PRO:HG3	1:F:324:TYR:OH	2.05	0.55
1:C:111:ILE:O	1:C:115:ILE:HG13	2.05	0.55
1:F:92:ARG:HB2	1:F:93:PRO:HD3	1.87	0.55
1:D:177:VAL:O	1:D:181:LEU:HG	2.05	0.55
1:D:246:VAL:HG22	1:D:360:LEU:HD11	1.88	0.55
1:E:274:GLU:O	1:E:278:ILE:HD13	2.07	0.55
1:C:122:LEU:CD2	1:C:215:VAL:HG11	2.36	0.55
1:C:54:LEU:O	1:C:58:LEU:HD13	2.06	0.55
1:C:138:TYR:OH	1:C:141:GLY:HA2	2.07	0.55
1:D:109:PRO:HA	1:D:112:ARG:HG2	1.88	0.55
1:F:133:PHE:CZ	1:F:136:ALA:HB2	2.41	0.55
1:C:164:ARG:HD3	1:C:278:ILE:HG22	1.88	0.55
1:C:109:PRO:HA	1:C:112:ARG:HG2	1.88	0.55
1:C:119:LEU:HD12	1:C:216:SER:HB2	1.89	0.55

Continued on next page...

For Manuscript Review

Continued from previous page...

Atom-1	Atom-2	Interatomic distance (Å)	Clash overlap (Å)
1:C:188:HIS:O	1:C:190:ILE:HG23	2.06	0.55
1:F:154:SER:HB2	1:F:156:ASP:OD1	2.06	0.55
1:E:102:LEU:HB3	1:E:142:GLU:OE1	2.07	0.55
1:F:107:ILE:HB	1:F:111:ILE:CD1	2.37	0.55
1:F:192:HIS:ND1	1:F:213:PHE:HB3	2.22	0.55
1:C:91:HIS:HB3	1:C:94:SER:OG	2.07	0.55
1:F:109:PRO:O	1:F:112:ARG:HG2	2.06	0.55
1:C:92:ARG:HB2	1:C:93:PRO:HD3	1.89	0.54
1:F:237:GLU:HB2	1:F:242:THR:O	2.06	0.54
1:D:267:ILE:HD11	1:D:325:ILE:CG1	2.37	0.54
1:A:169:ILE:HG23	1:A:379:PHE:CD1	2.43	0.54
1:F:54:LEU:O	1:F:58:LEU:HD13	2.07	0.54
1:B:101:LYS:HE3	1:B:212:ASP:OD1	2.07	0.54
1:B:267:ILE:HG21	1:B:321:LEU:HG	1.90	0.54
1:C:122:LEU:HD21	1:C:215:VAL:HG11	1.88	0.54
1:E:46:LEU:O	1:E:51:LYS:HE3	2.07	0.54
1:E:109:PRO:HA	1:E:112:ARG:HG2	1.88	0.54
1:F:108:LYS:O	1:F:111:ILE:HG12	2.06	0.54
1:B:169:ILE:HG23	1:B:379:PHE:HD1	1.71	0.54
1:D:274:GLU:O	1:D:278:ILE:HG13	2.07	0.54
1:E:199:ASN:HB3	1:E:211:CYS:O	2.07	0.54
1:E:318:ILE:HG23	1:E:319:PHE:N	2.22	0.54
3:E:402:VRA:H24	3:E:402:VRA:C10	2.37	0.54
1:A:78:LEU:CD2	1:A:88:LYS:HB2	2.36	0.54
1:D:105:LEU:HD21	1:D:222:SER:CB	2.36	0.54
1:D:193:ARG:NH1	1:D:220:ILE:HD13	2.22	0.54
1:F:107:ILE:HB	1:F:111:ILE:CG1	2.38	0.54
1:F:193:ARG:NH1	1:F:220:ILE:HG21	2.23	0.54
1:A:55:GLU:O	1:A:59:THR:HG23	2.08	0.54
1:D:91:HIS:HB3	1:D:94:SER:HG	1.72	0.54
1:D:105:LEU:HG	1:D:107:ILE:HG23	1.90	0.54
1:D:321:LEU:O	1:D:325:ILE:HG13	2.07	0.54
1:F:173:VAL:O	1:F:177:VAL:HG23	2.06	0.54
1:B:255:LEU:HD11	1:B:266:PRO:HG2	1.89	0.54
1:C:157:GLN:HA	1:C:160:LYS:HE2	1.90	0.54
1:D:51:LYS:HD2	1:D:54:LEU:HD21	1.90	0.54
1:F:112:ARG:O	1:F:116:ILE:HG12	2.08	0.54
1:B:150:MET:HE1	1:B:209:LYS:CD	2.31	0.54
1:C:133:PHE:HE1	1:C:145:ILE:HG23	1.73	0.54
1:C:128:PRO:HG2	1:C:129:TYR:CE2	2.43	0.54
1:C:67:LEU:HD23	1:C:72:PHE:CZ	2.39	0.53

Continued on next page...

For Manuscript Review

Continued from previous page...

Atom-1	Atom-2	Interatomic distance (Å)	Clash overlap (Å)
1:C:71:ASP:HB3	1:C:93:PRO:CD	2.38	0.53
1:D:324:TYR:HA	1:D:328:GLU:CG	2.38	0.53
1:A:382:TRP:O	1:A:386:THR:HG23	2.07	0.53
1:E:111:ILE:O	1:E:115:ILE:HG13	2.08	0.53
1:E:164:ARG:HD3	1:E:278:ILE:HG23	1.89	0.53
1:F:324:TYR:O	1:F:328:GLU:HB2	2.08	0.53
1:A:60:GLN:OE1	1:A:60:GLN:HA	2.08	0.53
1:C:116:ILE:O	1:C:120:GLN:HG2	2.09	0.53
1:D:100:ARG:HE	1:D:102:LEU:CD2	2.21	0.53
1:D:157:GLN:HA	1:D:160:LYS:CE	2.24	0.53
1:F:237:GLU:HG2	1:F:238:ARG:N	2.22	0.53
1:C:175:ILE:HG12	1:C:369:ILE:HG23	1.90	0.53
1:C:239:LEU:HD13	1:C:322:LEU:HG	1.90	0.53
1:C:250:ILE:HG23	1:C:363:LEU:HD11	1.90	0.53
1:C:322:LEU:HB3	1:D:319:PHE:CD1	2.43	0.53
1:A:164:ARG:HD3	1:A:278:ILE:CG2	2.38	0.53
1:C:169:ILE:HG23	1:C:379:PHE:CD1	2.41	0.53
1:E:68:LYS:HE3	1:E:70:ASP:HB2	1.91	0.53
1:A:92:ARG:HB2	1:A:93:PRO:HD3	1.91	0.53
1:B:269:PRO:HG2	1:B:324:TYR:OH	2.08	0.53
1:E:207:GLU:OE1	1:E:209:LYS:HE3	2.09	0.53
1:E:171:GLY:O	1:E:175:ILE:HG13	2.09	0.53
1:A:211:CYS:O	1:A:212:ASP:HB2	2.09	0.53
1:E:359:ASP:OD1	1:E:362:MET:HG3	2.09	0.53
1:B:154:SER:HB3	1:B:156:ASP:OD1	2.09	0.52
1:A:226:SER:CB	1:B:82:ASN:HD21	2.22	0.52
1:A:247:GLN:HB3	1:A:357:ARG:HH11	1.73	0.52
1:C:152:GLY:HA2	1:C:387:LEU:HD21	1.91	0.52
1:C:54:LEU:HD11	1:C:126:ASN:HB3	1.90	0.52
1:D:246:VAL:CG2	1:D:360:LEU:HD11	2.39	0.52
1:F:360:LEU:O	1:F:364:THR:HG23	2.10	0.52
1:C:326:VAL:O	1:C:352:LYS:HE3	2.10	0.52
1:E:122:LEU:HB2	1:E:133:PHE:CD2	2.45	0.52
1:E:267:ILE:HD12	1:E:325:ILE:HG12	1.92	0.52
1:C:112:ARG:O	1:C:116:ILE:HG12	2.08	0.52
1:C:370:LYS:O	1:C:374:VAL:HG13	2.10	0.52
1:B:344:GLU:O	1:B:348:LYS:HG3	2.10	0.52
1:F:101:LYS:HE2	3:F:402:VRA:O21	2.10	0.52
1:D:57:PHE:CZ	1:D:132:GLY:HA3	2.45	0.52
1:D:223:MET:HG3	3:D:402:VRA:H24A	1.90	0.52
1:E:319:PHE:HD1	1:F:322:LEU:HB3	1.75	0.52

Continued on next page...

For Manuscript Review

Continued from previous page...

Atom-1	Atom-2	Interatomic distance (Å)	Clash overlap (Å)
1:C:63:LYS:HE3	1:C:96:LEU:HD21	1.91	0.52
1:D:317:ALA:HB3	1:D:320:GLU:HB2	1.92	0.52
1:D:324:TYR:HA	1:D:328:GLU:HB2	1.90	0.52
1:B:358:ALA:HB3	1:B:363:LEU:HD21	1.92	0.52
1:D:138:TYR:HD2	1:D:143:ILE:HG12	1.75	0.51
1:E:387:LEU:HB2	1:E:389:LEU:HD22	1.91	0.51
1:B:265:TYR:OH	1:B:267:ILE:HG13	2.10	0.51
1:B:134:TYR:HE2	1:B:148:GLU:HA	1.75	0.51
1:D:164:ARG:HD3	1:D:278:ILE:CG2	2.39	0.51
1:E:169:ILE:HG23	1:E:379:PHE:HD1	1.75	0.51
1:F:46:LEU:HD11	1:F:128:PRO:HG3	1.91	0.51
1:D:64:VAL:CG1	1:D:96:LEU:HD12	2.40	0.51
1:B:61:LYS:HE2	1:B:133:PHE:O	2.11	0.51
1:A:267:ILE:O	1:A:269:PRO:HD3	2.11	0.51
1:A:319:PHE:HD1	1:B:322:LEU:HB3	1.75	0.51
1:C:56:ALA:O	1:C:60:GLN:HG2	2.10	0.51
1:E:114:GLN:HG3	1:E:117:ARG:NH2	2.25	0.51
1:E:216:SER:O	1:E:220:ILE:HG13	2.10	0.51
1:F:109:PRO:HA	1:F:112:ARG:HG2	1.93	0.51
1:B:57:PHE:CZ	1:B:132:GLY:HA3	2.46	0.51
1:A:265:TYR:HE2	1:A:321:LEU:HD11	1.76	0.51
1:C:111:ILE:HG23	1:C:218:GLN:HE21	1.74	0.51
1:C:324:TYR:HA	1:C:328:GLU:CB	2.37	0.51
1:D:270:PRO:HB2	1:D:275:LEU:HD21	1.92	0.51
1:F:78:LEU:HD21	1:F:88:LYS:HB2	1.93	0.51
1:C:258:VAL:O	1:C:262:VAL:HG22	2.11	0.50
1:D:111:ILE:O	1:D:115:ILE:HG13	2.11	0.50
1:F:60:GLN:NE2	1:F:63:LYS:HD2	2.26	0.50
1:B:131:VAL:HG21	1:B:201:LEU:HD12	1.92	0.50
1:B:154:SER:O	1:B:158:VAL:HG23	2.11	0.50
1:D:267:ILE:CG2	1:D:268:PRO:CD	2.84	0.50
1:E:105:LEU:HD21	1:E:222:SER:CB	2.40	0.50
1:F:175:ILE:HD11	1:F:372:SER:HB2	1.92	0.50
1:F:171:GLY:O	1:F:175:ILE:HG13	2.12	0.50
1:F:129:TYR:O	1:F:130:ILE:HD13	2.12	0.50
1:F:199:ASN:HB3	1:F:211:CYS:O	2.10	0.50
1:A:234:MET:HE3	1:A:238:ARG:NE	2.27	0.50
1:B:223:MET:O	1:B:224:ALA:HB3	2.12	0.50
1:C:111:ILE:HG23	1:C:218:GLN:CG	2.42	0.50
1:D:165:ILE:HD12	1:D:260:LEU:HB3	1.94	0.50
1:F:122:LEU:HD21	1:F:215:VAL:HG21	1.92	0.50

Continued on next page...

For Manuscript Review

Continued from previous page...

Atom-1	Atom-2	Interatomic distance (Å)	Clash overlap (Å)
1:C:371:ARG:O	1:C:374:VAL:HG22	2.11	0.50
1:A:71:ASP:HB3	1:A:93:PRO:HD2	1.93	0.50
1:F:167:GLU:OE2	1:F:342:PHE:HB2	2.12	0.49
1:B:114:GLN:O	1:B:118:GLU:HG3	2.12	0.49
1:D:258:VAL:O	1:D:262:VAL:HG22	2.12	0.49
1:A:122:LEU:HB2	1:A:133:PHE:CD2	2.47	0.49
1:E:175:ILE:HD11	1:E:372:SER:CB	2.42	0.49
1:E:237:GLU:HG2	1:E:238:ARG:N	2.27	0.49
1:E:387:LEU:HB2	1:E:389:LEU:CD2	2.41	0.49
1:B:100:ARG:HG2	1:B:102:LEU:CD1	2.42	0.49
1:C:119:LEU:CD1	1:C:216:SER:HB2	2.43	0.49
1:B:169:ILE:HG23	1:B:379:PHE:CD1	2.48	0.49
1:B:188:HIS:O	1:B:190:ILE:HG23	2.11	0.49
1:B:219:LEU:O	1:B:223:MET:HG2	2.13	0.49
1:D:49:GLN:HA	1:D:52:LYS:HG2	1.95	0.49
1:D:64:VAL:HG12	1:D:94:SER:CB	2.42	0.49
1:E:211:CYS:O	1:E:212:ASP:HB2	2.13	0.49
1:B:370:LYS:O	1:B:374:VAL:HG13	2.13	0.49
1:C:238:ARG:HG2	1:C:244:TYR:CB	2.33	0.49
1:D:169:ILE:O	1:D:173:VAL:HG23	2.12	0.49
1:E:78:LEU:HD21	1:E:88:LYS:HB2	1.95	0.49
1:F:188:HIS:O	1:F:190:ILE:HG23	2.13	0.49
1:F:240:GLN:OE1	1:F:326:VAL:HG13	2.12	0.49
1:A:105:LEU:HD21	1:A:222:SER:CB	2.43	0.49
1:A:274:GLU:O	1:A:278:ILE:HG12	2.13	0.49
1:C:274:GLU:O	1:C:278:ILE:HG13	2.13	0.49
1:D:131:VAL:HG21	1:D:201:LEU:HD12	1.95	0.49
1:E:171:GLY:HA2	1:E:368:PHE:CE2	2.48	0.49
1:F:370:LYS:O	1:F:374:VAL:HG13	2.13	0.49
1:F:78:LEU:HB2	1:F:86:VAL:HG12	1.95	0.49
1:C:105:LEU:HG	1:C:107:ILE:CG2	2.42	0.48
1:D:118:GLU:O	1:D:121:VAL:HG22	2.13	0.48
1:A:167:GLU:OE2	1:A:342:PHE:HB2	2.13	0.48
1:C:115:ILE:O	1:C:119:LEU:HD13	2.12	0.48
1:E:324:TYR:HA	1:E:328:GLU:HB2	1.95	0.48
1:C:169:ILE:HD13	1:C:377:VAL:HG11	1.95	0.48
1:D:265:TYR:CD1	1:D:266:PRO:HD2	2.47	0.48
1:D:107:ILE:HB	1:D:111:ILE:HD11	1.93	0.48
1:A:91:HIS:HB3	1:A:94:SER:OG	2.14	0.48
1:B:116:ILE:O	1:B:120:GLN:HG2	2.14	0.48
1:C:319:PHE:HB3	1:D:239:LEU:O	2.14	0.48

Continued on next page...

For Manuscript Review

Continued from previous page...

Atom-1	Atom-2	Interatomic distance (Å)	Clash overlap (Å)
1:D:191:MET:HE2	1:D:193:ARG:HG2	1.95	0.48
1:D:267:ILE:HG23	1:D:268:PRO:HD3	1.91	0.48
1:F:115:ILE:HG12	1:F:218:GLN:HB3	1.96	0.48
1:F:250:ILE:HD12	1:F:363:LEU:HD12	1.95	0.48
1:C:59:THR:O	1:C:62:ALA:HB3	2.14	0.48
1:F:46:LEU:HD11	1:F:50:GLN:HB3	1.95	0.48
1:D:265:TYR:HD1	1:D:266:PRO:HD2	1.78	0.48
1:E:91:HIS:HB3	1:E:94:SER:HB3	1.95	0.48
1:F:107:ILE:HB	1:F:111:ILE:HD11	1.94	0.48
1:A:114:GLN:O	1:A:118:GLU:HG3	2.14	0.48
1:B:109:PRO:O	1:B:112:ARG:HG2	2.13	0.48
1:B:195:VAL:HB	1:B:252:SER:HB2	1.95	0.48
1:B:216:SER:O	1:B:220:ILE:HG13	2.14	0.48
1:C:122:LEU:HD21	1:C:215:VAL:CG2	2.41	0.48
1:E:118:GLU:O	1:E:121:VAL:HG22	2.14	0.48
1:E:169:ILE:HG23	1:E:379:PHE:CD1	2.48	0.48
3:A:402:VRA:H31B	3:A:402:VRA:N18	2.29	0.48
1:B:92:ARG:HB3	1:B:93:PRO:HD3	1.94	0.47
1:E:318:ILE:HG23	1:E:319:PHE:H	1.79	0.47
1:A:122:LEU:CD2	1:A:215:VAL:HG11	2.44	0.47
1:B:109:PRO:HA	1:B:112:ARG:HG2	1.96	0.47
1:B:267:ILE:HG21	1:B:321:LEU:CD2	2.44	0.47
1:F:128:PRO:HG2	1:F:129:TYR:CD2	2.47	0.47
1:F:133:PHE:CE1	1:F:145:ILE:HG23	2.47	0.47
1:B:128:PRO:HG2	1:B:129:TYR:CD2	2.50	0.47
1:B:359:ASP:OD1	1:B:362:MET:HG3	2.15	0.47
1:C:97:ILE:O	1:C:97:ILE:HG13	2.13	0.47
1:D:321:LEU:CD2	1:D:325:ILE:HD11	2.44	0.47
1:F:318:ILE:HG23	1:F:319:PHE:N	2.30	0.47
1:A:267:ILE:CG2	1:A:321:LEU:HD12	2.44	0.47
1:A:318:ILE:HG23	1:A:319:PHE:N	2.29	0.47
1:D:127:SER:OG	1:D:128:PRO:HD2	2.14	0.47
1:D:150:MET:HE1	1:D:209:LYS:CD	2.37	0.47
1:D:191:MET:HG3	1:D:193:ARG:HG2	1.95	0.47
1:D:350:LEU:O	1:D:351:ILE:HD13	2.14	0.47
1:E:85:VAL:HG23	1:E:85:VAL:O	2.14	0.47
1:E:321:LEU:HD23	1:E:321:LEU:C	2.40	0.47
1:C:53:ARG:CZ	1:C:205:ARG:HG3	2.45	0.47
1:C:267:ILE:CG2	1:C:321:LEU:HD12	2.32	0.47
1:F:122:LEU:CD2	1:F:215:VAL:HG11	2.44	0.47
1:C:169:ILE:O	1:C:173:VAL:HG23	2.15	0.47

Continued on next page...

For Manuscript Review

Continued from previous page...

Atom-1	Atom-2	Interatomic distance (Å)	Clash overlap (Å)
1:E:105:LEU:HD23	1:E:107:ILE:HD13	1.96	0.47
1:E:164:ARG:HD3	1:E:278:ILE:CG2	2.45	0.47
1:A:223:MET:HE2	3:A:402:VRA:H23	1.97	0.47
1:B:112:ARG:O	1:B:116:ILE:HG12	2.14	0.47
1:B:247:GLN:CB	1:B:357:ARG:HH11	2.28	0.47
1:C:177:VAL:HG22	1:C:210:LEU:HD21	1.96	0.47
1:C:250:ILE:CG2	1:C:363:LEU:HD11	2.44	0.47
1:D:223:MET:HE2	3:D:402:VRA:H23	1.96	0.47
1:E:47:ASP:O	1:E:51:LYS:HG3	2.15	0.47
1:E:54:LEU:O	1:E:58:LEU:HD13	2.15	0.47
1:E:71:ASP:HB3	1:E:93:PRO:CD	2.44	0.47
1:E:83:GLY:O	1:E:103:ILE:HG23	2.14	0.47
1:A:164:ARG:HD3	1:A:278:ILE:HG22	1.97	0.47
1:C:267:ILE:O	1:C:269:PRO:HD3	2.15	0.47
4:F:401:ANP:H5'1	4:F:401:ANP:H8	1.97	0.47
1:B:185:ARG:NH1	1:B:246:VAL:HG21	2.30	0.47
1:D:360:LEU:O	1:D:364:THR:HG23	2.15	0.47
1:F:106:GLU:O	1:F:106:GLU:HG3	2.14	0.47
1:F:234:MET:HE3	1:F:238:ARG:CZ	2.45	0.47
1:A:67:LEU:HD12	1:A:67:LEU:N	2.30	0.47
1:F:165:ILE:HD12	1:F:260:LEU:HB3	1.97	0.47
1:A:267:ILE:HG21	1:A:321:LEU:HD12	1.96	0.46
1:A:333:LEU:HD11	1:A:346:VAL:HG11	1.97	0.46
1:C:78:LEU:HD13	4:C:402:ANP:C2	2.45	0.46
1:E:147:MET:HE2	3:E:402:VRA:H4	1.97	0.46
1:E:256:SER:O	1:E:260:LEU:HG	2.15	0.46
1:A:54:LEU:HD11	1:A:126:ASN:HB3	1.96	0.46
1:A:106:GLU:O	1:A:106:GLU:HG3	2.15	0.46
1:A:216:SER:O	1:A:220:ILE:HG13	2.16	0.46
1:C:171:GLY:O	1:C:175:ILE:HG13	2.15	0.46
1:F:339:THR:O	1:F:343:GLN:HG3	2.15	0.46
1:A:119:LEU:CD1	1:A:216:SER:HB2	2.45	0.46
1:A:188:HIS:O	1:A:190:ILE:HG23	2.15	0.46
1:B:150:MET:HB3	1:B:202:VAL:O	2.16	0.46
1:C:265:TYR:CZ	1:C:267:ILE:HG12	2.51	0.46
1:F:137:PHE:HE1	1:F:146:CYS:SG	2.37	0.46
1:A:109:PRO:HA	1:A:112:ARG:HG2	1.98	0.46
1:A:265:TYR:CE2	1:A:267:ILE:HB	2.50	0.46
1:A:279:PHE:CE2	1:A:334:PRO:HG3	2.50	0.46
1:F:134:TYR:HE2	1:F:148:GLU:HA	1.81	0.46
1:A:47:ASP:O	1:A:51:LYS:HG3	2.16	0.46

Continued on next page...

For Manuscript Review

Continued from previous page...

Atom-1	Atom-2	Interatomic distance (Å)	Clash overlap (Å)
1:B:261:ALA:HB1	1:B:338:PHE:CZ	2.50	0.46
1:E:167:GLU:OE2	1:E:339:THR:HG23	2.15	0.46
1:C:100:ARG:NE	1:C:102:LEU:HD21	2.30	0.46
1:D:74:ARG:HA	1:D:89:VAL:HG12	1.97	0.46
1:F:102:LEU:HB3	1:F:142:GLU:OE1	2.16	0.46
1:F:103:ILE:HD13	3:F:402:VRA:H23A	1.96	0.46
1:F:116:ILE:O	1:F:120:GLN:HG2	2.16	0.46
1:F:122:LEU:HD23	1:F:215:VAL:HG11	1.97	0.46
1:A:131:VAL:HG21	1:A:201:LEU:HD12	1.97	0.46
1:B:358:ALA:CB	1:B:363:LEU:HD21	2.46	0.46
1:D:116:ILE:O	1:D:120:GLN:HG2	2.15	0.46
1:B:91:HIS:HB3	1:B:94:SER:HG	1.80	0.46
1:C:192:HIS:ND1	1:C:213:PHE:HB3	2.31	0.46
1:E:169:ILE:HD13	1:E:377:VAL:HG11	1.98	0.46
1:A:370:LYS:O	1:A:374:VAL:HG13	2.16	0.46
1:E:202:VAL:HG23	1:E:383:LEU:HD21	1.98	0.46
1:F:234:MET:HE3	1:F:238:ARG:HB3	1.98	0.45
1:A:64:VAL:CG2	1:A:96:LEU:HD12	2.46	0.45
1:A:169:ILE:HG23	1:A:379:PHE:HD1	1.81	0.45
1:B:128:PRO:HG2	1:B:129:TYR:CE2	2.51	0.45
1:A:105:LEU:HD23	1:A:107:ILE:HD13	1.97	0.45
1:A:112:ARG:O	1:A:116:ILE:HG12	2.16	0.45
1:A:119:LEU:HD12	1:A:216:SER:HB2	1.97	0.45
1:B:108:LYS:O	1:B:111:ILE:HG12	2.16	0.45
1:B:195:VAL:O	1:B:252:SER:HB3	2.17	0.45
1:B:202:VAL:HG11	1:B:379:PHE:HZ	1.81	0.45
1:D:109:PRO:HA	1:D:112:ARG:NE	2.31	0.45
1:D:185:ARG:HH12	1:D:189:GLN:HG3	1.81	0.45
1:F:169:ILE:HD13	1:F:377:VAL:CG1	2.47	0.45
1:A:226:SER:HA	1:B:229:GLY:HA2	1.98	0.45
1:B:44:LEU:HD11	1:B:183:TYR:HD1	1.82	0.45
1:B:318:ILE:HG23	1:B:319:PHE:N	2.31	0.45
1:F:67:LEU:HD12	1:F:67:LEU:N	2.31	0.45
1:A:122:LEU:HD21	1:A:215:VAL:HG11	1.98	0.45
1:C:111:ILE:HG23	1:C:218:GLN:HG2	1.99	0.45
1:E:237:GLU:HB2	1:E:242:THR:O	2.17	0.45
1:E:328:GLU:HB3	1:E:329:PRO:CD	2.44	0.45
1:E:360:LEU:O	1:E:364:THR:HG23	2.16	0.45
1:A:267:ILE:HG21	1:A:321:LEU:CD1	2.46	0.45
1:B:100:ARG:HG2	1:B:102:LEU:HD11	1.99	0.45
1:B:192:HIS:CE1	1:B:213:PHE:HB3	2.51	0.45

Continued on next page...

For Manuscript Review

Continued from previous page...

Atom-1	Atom-2	Interatomic distance (Å)	Clash overlap (Å)
1:D:267:ILE:HG12	1:D:321:LEU:HG	1.99	0.45
1:A:202:VAL:HA	1:A:207:GLU:O	2.17	0.45
1:F:169:ILE:HG23	1:F:379:PHE:HD1	1.82	0.45
1:C:235:ALA:HB2	1:C:248:SER:HA	1.99	0.45
1:C:318:ILE:HG23	1:C:319:PHE:N	2.31	0.45
1:A:46:LEU:HD21	1:A:128:PRO:HD3	1.99	0.44
1:B:108:LYS:H	1:B:111:ILE:HD11	1.81	0.44
1:D:138:TYR:CZ	1:D:141:GLY:HA2	2.52	0.44
1:E:150:MET:HB3	1:E:202:VAL:O	2.17	0.44
1:E:169:ILE:O	1:E:173:VAL:HG23	2.17	0.44
1:B:159:LEU:HD22	1:B:260:LEU:HD23	1.99	0.44
1:F:182:ALA:O	1:F:186:GLU:HG2	2.17	0.44
1:B:47:ASP:HB3	1:B:50:GLN:HG3	1.99	0.44
1:C:318:ILE:HG23	1:C:319:PHE:H	1.82	0.44
1:C:319:PHE:HD1	1:D:322:LEU:HB3	1.82	0.44
1:A:192:HIS:CD2	1:A:213:PHE:HB3	2.52	0.44
1:B:269:PRO:HA	1:B:270:PRO:HD3	1.88	0.44
1:F:131:VAL:HG21	1:F:201:LEU:HD12	2.00	0.44
1:B:122:LEU:HD23	1:B:215:VAL:HG11	1.96	0.44
1:C:264:ARG:HH11	1:C:270:PRO:HG3	1.82	0.44
1:B:107:ILE:CB	1:B:111:ILE:HD11	2.44	0.44
1:D:267:ILE:HG12	1:D:321:LEU:CG	2.47	0.44
1:F:169:ILE:HG23	1:F:379:PHE:CD1	2.51	0.44
1:B:105:LEU:HD21	1:B:222:SER:HB3	2.00	0.44
1:B:138:TYR:CZ	1:B:141:GLY:HA2	2.52	0.44
1:B:255:LEU:CD1	1:B:266:PRO:HG2	2.46	0.44
1:C:133:PHE:HZ	1:C:136:ALA:HB2	1.83	0.44
1:C:344:GLU:O	1:C:348:LYS:HG3	2.17	0.44
1:D:54:LEU:HD12	1:D:54:LEU:C	2.42	0.44
1:D:128:PRO:HG2	1:D:129:TYR:CD2	2.53	0.44
1:D:147:MET:HE2	3:D:402:VRA:H6	2.00	0.44
1:D:168:GLU:HB3	1:D:377:VAL:HG21	1.99	0.44
1:E:131:VAL:HG23	1:E:210:LEU:O	2.18	0.44
1:B:167:GLU:OE1	1:B:339:THR:HG23	2.18	0.43
1:D:86:VAL:HG22	1:D:101:LYS:HB2	1.99	0.43
1:C:53:ARG:NH2	1:C:205:ARG:HG3	2.33	0.43
1:E:53:ARG:HD2	1:E:205:ARG:HH11	1.83	0.43
1:F:203:ASN:ND2	1:F:207:GLU:HB2	2.33	0.43
1:F:359:ASP:OD1	1:F:362:MET:HG3	2.18	0.43
1:B:185:ARG:HG3	1:B:186:GLU:N	2.33	0.43
1:C:339:THR:O	1:C:343:GLN:HG3	2.18	0.43

Continued on next page...

For Manuscript Review

Continued from previous page...

Atom-1	Atom-2	Interatomic distance (Å)	Clash overlap (Å)
1:D:231:ARG:HG2	1:D:265:TYR:CD2	2.53	0.43
1:D:328:GLU:HB3	1:D:329:PRO:CD	2.45	0.43
1:E:159:LEU:HD22	1:E:260:LEU:HD23	2.00	0.43
1:E:234:MET:HE2	1:E:234:MET:HB3	1.85	0.43
1:F:55:GLU:O	1:F:59:THR:HG23	2.17	0.43
1:F:165:ILE:HD12	1:F:260:LEU:HD22	1.99	0.43
1:F:207:GLU:OE1	1:F:209:LYS:HE3	2.18	0.43
1:A:316:MET:HE3	1:A:320:GLU:HG2	2.00	0.43
1:D:108:LYS:N	1:D:111:ILE:HD11	2.32	0.43
1:D:130:ILE:HG23	1:D:213:PHE:CE1	2.53	0.43
1:E:128:PRO:HG2	1:E:129:TYR:CE2	2.53	0.43
1:E:167:GLU:OE2	1:E:342:PHE:HB2	2.18	0.43
1:D:238:ARG:HG2	1:D:244:TYR:HB3	2.01	0.43
1:A:94:SER:CB	1:A:96:LEU:HG	2.49	0.43
1:D:261:ALA:HB1	1:D:338:PHE:CZ	2.53	0.43
1:E:122:LEU:CD2	1:E:215:VAL:HG11	2.48	0.43
1:E:265:TYR:CZ	1:E:267:ILE:HG12	2.53	0.43
1:B:328:GLU:HB3	1:B:329:PRO:CD	2.40	0.43
1:C:92:ARG:N	1:C:93:PRO:CD	2.81	0.43
1:C:216:SER:O	1:C:220:ILE:HG13	2.18	0.43
1:D:72:PHE:HE1	1:D:98:MET:SD	2.41	0.43
1:E:241:GLY:HA3	1:F:317:ALA:HB2	2.00	0.43
1:A:147:MET:HE2	3:A:402:VRA:H4	2.00	0.43
1:C:234:MET:CE	1:C:238:ARG:HB3	2.48	0.43
1:C:275:LEU:HD13	1:C:334:PRO:HB3	2.00	0.43
1:D:119:LEU:CD1	1:D:216:SER:HB2	2.48	0.43
1:E:197:PRO:HG3	1:E:256:SER:HB3	2.00	0.43
1:E:339:THR:O	1:E:343:GLN:HG3	2.19	0.43
1:B:105:LEU:HD21	1:B:222:SER:CB	2.49	0.43
1:C:104:HIS:HA	1:C:142:GLU:HG2	2.00	0.43
1:C:175:ILE:HD11	1:C:372:SER:CB	2.45	0.43
1:C:199:ASN:HB3	1:C:211:CYS:O	2.19	0.43
1:E:234:MET:HE3	1:E:238:ARG:NE	2.34	0.43
1:F:219:LEU:HD23	3:F:402:VRA:C12	2.48	0.43
1:C:195:VAL:O	1:C:252:SER:HB3	2.19	0.43
1:D:67:LEU:CD1	1:D:135:GLY:HA3	2.48	0.42
1:D:318:ILE:HG23	1:D:319:PHE:N	2.34	0.42
1:F:91:HIS:HB3	1:F:94:SER:HG	1.84	0.42
1:A:172:LYS:HD3	1:A:172:LYS:HA	1.75	0.42
1:A:191:MET:HG3	1:A:193:ARG:CG	2.49	0.42
1:C:164:ARG:HD3	1:C:278:ILE:CG2	2.50	0.42

Continued on next page...

For Manuscript Review

Continued from previous page...

Atom-1	Atom-2	Interatomic distance (Å)	Clash overlap (Å)
1:A:264:ARG:HH21	1:A:270:PRO:HG3	1.84	0.42
1:D:78:LEU:CG	1:D:88:LYS:HB2	2.48	0.42
1:A:57:PHE:CZ	1:A:132:GLY:HA3	2.54	0.42
1:A:236:PRO:HD3	1:A:251:TRP:CE2	2.54	0.42
1:E:168:GLU:H	1:E:168:GLU:HG3	1.66	0.42
1:B:187:LYS:HG3	1:B:187:LYS:O	2.20	0.42
1:C:47:ASP:OD1	1:C:48:GLU:N	2.53	0.42
1:E:57:PHE:CZ	1:E:132:GLY:HA3	2.54	0.42
1:E:129:TYR:CZ	1:E:179:ARG:HD3	2.55	0.42
1:E:254:GLY:O	1:E:258:VAL:HG23	2.19	0.42
1:F:167:GLU:HG3	1:F:342:PHE:CD2	2.55	0.42
1:F:169:ILE:HD13	1:F:377:VAL:HG11	2.02	0.42
1:C:61:LYS:O	1:C:64:VAL:HG12	2.20	0.42
1:C:165:ILE:HD12	1:C:260:LEU:HB3	2.01	0.42
1:E:197:PRO:HD3	1:E:256:SER:OG	2.18	0.42
1:F:154:SER:HA	1:F:200:ILE:O	2.20	0.42
1:F:169:ILE:O	1:F:173:VAL:HG23	2.19	0.42
1:F:366:HIS:HD1	1:F:368:PHE:H	1.68	0.42
1:D:216:SER:O	1:D:220:ILE:HG13	2.19	0.42
1:E:317:ALA:HB1	1:F:239:LEU:O	2.20	0.42
1:B:79:GLY:HA3	4:B:402:ANP:H4'	2.01	0.42
1:D:67:LEU:HD12	1:D:135:GLY:HA3	2.02	0.42
1:A:48:GLU:CD	1:A:48:GLU:H	2.28	0.42
1:B:60:GLN:O	1:B:63:LYS:HB2	2.20	0.42
1:C:111:ILE:CG2	1:C:218:GLN:HE21	2.33	0.42
1:D:169:ILE:HD13	1:D:377:VAL:HG11	2.01	0.42
1:D:324:TYR:HA	1:D:328:GLU:CB	2.50	0.42
1:F:331:PRO:HG2	1:F:350:LEU:HD13	2.02	0.42
1:A:360:LEU:O	1:A:364:THR:HG23	2.20	0.42
1:B:106:GLU:O	1:B:106:GLU:HG3	2.19	0.42
1:C:235:ALA:HB1	1:C:236:PRO:HD2	2.01	0.42
1:D:195:VAL:HG12	1:D:195:VAL:O	2.19	0.42
1:B:328:GLU:CB	1:B:329:PRO:HD2	2.42	0.41
1:C:78:LEU:HD21	1:C:88:LYS:HB2	2.02	0.41
1:C:267:ILE:HD13	1:C:267:ILE:HA	1.81	0.41
4:F:401:ANP:H5'1	4:F:401:ANP:C8	2.50	0.41
1:A:97:ILE:O	1:A:97:ILE:HG13	2.20	0.41
1:C:234:MET:HE2	1:C:234:MET:HB3	1.80	0.41
1:A:170:LEU:HD12	1:A:342:PHE:CE2	2.55	0.41
1:D:191:MET:HG3	1:D:193:ARG:CG	2.51	0.41
1:F:184:LEU:HD22	1:F:190:ILE:HD11	2.02	0.41

Continued on next page...

For Manuscript Review

Continued from previous page...

Atom-1	Atom-2	Interatomic distance (Å)	Clash overlap (Å)
1:A:73:GLU:HB2	1:A:92:ARG:HE	1.85	0.41
1:D:86:VAL:HB	4:D:401:ANP:H5'2	2.02	0.41
1:D:185:ARG:NH1	1:D:189:GLN:HG3	2.35	0.41
1:F:102:LEU:HD13	1:F:142:GLU:OE1	2.19	0.41
1:B:122:LEU:HD21	1:B:215:VAL:HG21	2.03	0.41
1:C:122:LEU:CD2	1:C:215:VAL:HG21	2.41	0.41
1:D:318:ILE:HG23	1:D:319:PHE:H	1.86	0.41
1:F:49:GLN:O	1:F:53:ARG:HD3	2.21	0.41
1:F:335:ASN:HA	1:F:343:GLN:OE1	2.20	0.41
1:C:265:TYR:CD1	1:C:266:PRO:HD2	2.56	0.41
1:D:265:TYR:CZ	1:D:267:ILE:HB	2.56	0.41
1:E:328:GLU:CB	1:E:329:PRO:HD2	2.45	0.41
1:A:159:LEU:HD22	1:A:260:LEU:HD23	2.03	0.41
1:A:172:LYS:HE3	1:A:377:VAL:HB	2.03	0.41
1:A:269:PRO:HA	1:A:270:PRO:HD3	1.90	0.41
1:B:333:LEU:HG	1:B:346:VAL:HG21	2.02	0.41
1:B:324:TYR:HA	1:B:328:GLU:HB2	2.02	0.41
1:C:335:ASN:HA	1:C:343:GLN:OE1	2.20	0.41
4:D:401:ANP:H8	4:D:401:ANP:H5'1	2.03	0.41
1:E:97:ILE:HG13	1:E:97:ILE:O	2.20	0.41
1:E:175:ILE:HG12	1:E:369:ILE:HG23	2.03	0.41
1:E:345:PHE:CZ	1:E:369:ILE:HD11	2.56	0.41
1:A:101:LYS:NZ	1:A:212:ASP:OD1	2.54	0.41
1:A:223:MET:HE3	1:A:223:MET:HB3	1.90	0.41
1:C:67:LEU:HD12	1:C:67:LEU:N	2.36	0.41
1:C:177:VAL:O	1:C:181:LEU:HG	2.21	0.41
1:D:369:ILE:O	1:D:373:GLU:HG3	2.21	0.41
1:E:265:TYR:CZ	1:E:267:ILE:CG1	3.04	0.41
1:E:322:LEU:HB3	1:F:319:PHE:CD1	2.50	0.41
1:F:60:GLN:NE2	1:F:60:GLN:HA	2.36	0.41
1:F:60:GLN:O	1:F:63:LYS:HB2	2.21	0.41
1:F:269:PRO:HA	1:F:270:PRO:HD3	1.92	0.41
1:A:108:LYS:HB3	1:A:109:PRO:HD2	2.03	0.41
1:B:184:LEU:HD22	1:B:190:ILE:HD11	2.03	0.41
1:B:267:ILE:HD13	1:B:267:ILE:HA	1.90	0.41
1:C:154:SER:CB	1:C:156:ASP:OD1	2.69	0.41
1:D:122:LEU:HD23	1:D:215:VAL:HG11	2.03	0.41
1:D:202:VAL:HG23	1:D:383:LEU:HD21	2.02	0.40
1:E:71:ASP:HB3	1:E:93:PRO:HD3	2.03	0.40
1:E:165:ILE:HD12	1:E:260:LEU:HB3	2.01	0.40
1:E:234:MET:HE3	1:E:238:ARG:HB3	2.01	0.40

Continued on next page...

For Manuscript Review

Continued from previous page...

Atom-1	Atom-2	Interatomic distance (Å)	Clash overlap (Å)
1:B:98:MET:HB2	1:B:147:MET:O	2.21	0.40
1:E:76:SER:O	1:E:87:THR:HG23	2.20	0.40
1:E:213:PHE:CD2	1:E:215:VAL:HG13	2.56	0.40
1:F:108:LYS:H	1:F:111:ILE:HD11	1.86	0.40
1:F:326:VAL:O	1:F:352:LYS:HE3	2.21	0.40
1:C:128:PRO:HG2	1:C:129:TYR:CD2	2.55	0.40
1:C:167:GLU:HG3	1:C:342:PHE:CD2	2.56	0.40
1:D:169:ILE:HD13	1:D:377:VAL:CG1	2.51	0.40
1:B:360:LEU:O	1:B:364:THR:HG23	2.21	0.40
1:C:236:PRO:HB3	1:C:326:VAL:HA	2.03	0.40
1:E:60:GLN:HB3	1:E:96:LEU:HD11	2.04	0.40
1:A:250:ILE:HD11	1:A:360:LEU:CD1	2.52	0.40
1:B:237:GLU:HG2	1:B:238:ARG:N	2.37	0.40
1:E:321:LEU:HD22	1:E:322:LEU:HD12	2.02	0.40

There are no symmetry-related clashes.

5.3 Torsion angles (i)

5.3.1 Protein backbone (i)

In the following table, the Percentiles column shows the percent Ramachandran outliers of the chain as a percentile score with respect to all X-ray entries followed by that with respect to entries of similar resolution.

The Analysed column shows the number of residues for which the backbone conformation was analysed, and the total number of residues.

Mol	Chain	Analysed	Favoured	Allowed	Outliers	Percentiles	
1	A	301/346 (87%)	290 (96%)	10 (3%)	1 (0%)	36	65
1	B	301/346 (87%)	297 (99%)	4 (1%)	0	100	100
1	C	296/346 (86%)	291 (98%)	5 (2%)	0	100	100
1	D	292/346 (84%)	286 (98%)	6 (2%)	0	100	100
1	E	296/346 (86%)	289 (98%)	7 (2%)	0	100	100
1	F	296/346 (86%)	292 (99%)	4 (1%)	0	100	100
All	All	1782/2076 (86%)	1745 (98%)	36 (2%)	1 (0%)	48	75

All (1) Ramachandran outliers are listed below:

For Manuscript Review

Mol	Chain	Res	Type
1	A	336	GLY

5.3.2 Protein sidechains (i)

In the following table, the Percentiles column shows the percent sidechain outliers of the chain as a percentile score with respect to all X-ray entries followed by that with respect to entries of similar resolution.

The Analysed column shows the number of residues for which the sidechain conformation was analysed, and the total number of residues.

Mol	Chain	Analysed	Rotameric	Outliers	Percentiles	
1	A	267/299 (89%)	267 (100%)	0	100	100
1	B	266/299 (89%)	266 (100%)	0	100	100
1	C	264/299 (88%)	264 (100%)	0	100	100
1	D	262/299 (88%)	262 (100%)	0	100	100
1	E	264/299 (88%)	264 (100%)	0	100	100
1	F	264/299 (88%)	264 (100%)	0	100	100
All	All	1587/1794 (88%)	1587 (100%)	0	100	100

There are no protein residues with a non-rotameric sidechain to report.

Sometimes sidechains can be flipped to improve hydrogen bonding and reduce clashes. All (18) such sidechains are listed below:

Mol	Chain	Res	Type
1	A	126	ASN
1	A	218	GLN
1	A	343	GLN
1	B	82	ASN
1	B	192	HIS
1	B	343	GLN
1	B	365	ASN
1	C	126	ASN
1	C	218	GLN
1	C	247	GLN
1	D	335	ASN
1	D	343	GLN
1	E	126	ASN
1	F	60	GLN
1	F	120	GLN
1	F	126	ASN

Continued on next page...

For Manuscript Review

Continued from previous page...

Mol	Chain	Res	Type
1	F	157	GLN
1	F	365	ASN

5.3.3 RNA (i)

There are no RNA molecules in this entry.

5.4 Non-standard residues in protein, DNA, RNA chains (i)

There are no non-standard protein/DNA/RNA residues in this entry.

5.5 Carbohydrates (i)

There are no oligosaccharides in this entry.

5.6 Ligand geometry (i)

Of 18 ligands modelled in this entry, 6 are monoatomic - leaving 12 for Mogul analysis.

In the following table, the Counts columns list the number of bonds (or angles) for which Mogul statistics could be retrieved, the number of bonds (or angles) that are observed in the model and the number of bonds (or angles) that are defined in the Chemical Component Dictionary. The Link column lists molecule types, if any, to which the group is linked. The Z score for a bond length (or angle) is the number of standard deviations the observed value is removed from the expected value. A bond length (or angle) with $|Z| > 2$ is considered an outlier worth inspection. RMSZ is the root-mean-square of all Z scores of the bond lengths (or angles).

Mol	Type	Chain	Res	Link	Bond lengths			Bond angles		
					Counts	RMSZ	$\#\|Z\| > 2$	Counts	RMSZ	$\#\|Z\| > 2$
3	VRA	E	402	-	30,33,33	2.51	8 (26%)	39,50,50	2.49	11 (28%)
3	VRA	D	402	-	30,33,33	2.47	6 (20%)	39,50,50	2.51	10 (25%)
3	VRA	F	402	-	30,33,33	2.56	7 (23%)	39,50,50	2.61	10 (25%)
3	VRA	C	403	-	30,33,33	2.51	8 (26%)	39,50,50	2.23	10 (25%)
3	VRA	B	403	-	30,33,33	2.43	7 (23%)	39,50,50	2.29	7 (17%)
3	VRA	A	402	-	30,33,33	2.47	8 (26%)	39,50,50	2.47	10 (25%)
4	ANP	A	403	2	32,33,33	2.22	5 (15%)	44,52,52	1.75	12 (27%)
4	ANP	F	401	2	32,33,33	2.18	5 (15%)	44,52,52	1.67	8 (18%)
4	ANP	B	402	2	32,33,33	2.21	5 (15%)	44,52,52	1.44	7 (15%)

For Manuscript Review

Mol	Type	Chain	Res	Link	Bond lengths			Bond angles		
					Counts	RMSZ	#|Z| > 2	Counts	RMSZ	#|Z| > 2
4	ANP	E	401	2	32,33,33	2.18	5 (15%)	44,52,52	1.44	5 (11%)
4	ANP	C	402	2	32,33,33	2.15	5 (15%)	44,52,52	1.38	5 (11%)
4	ANP	D	401	2	32,33,33	2.21	5 (15%)	44,52,52	1.66	9 (20%)

In the following table, the Chirals column lists the number of chiral outliers, the number of chiral centers analysed, the number of these observed in the model and the number defined in the Chemical Component Dictionary. Similar counts are reported in the Torsion and Rings columns. '-' means no outliers of that kind were identified.

Mol	Type	Chain	Res	Link	Chirals	Torsions	Rings
3	VRA	E	402	-	-	9/15/31/31	0/3/3/3
3	VRA	D	402	-	-	4/15/31/31	0/3/3/3
3	VRA	F	402	-	-	3/15/31/31	0/3/3/3
3	VRA	C	403	-	-	10/15/31/31	0/3/3/3
3	VRA	B	403	-	-	5/15/31/31	0/3/3/3
3	VRA	A	402	-	-	7/15/31/31	0/3/3/3
4	ANP	A	403	2	-	3/18/38/38	0/3/3/3
4	ANP	F	401	2	-	6/18/38/38	0/3/3/3
4	ANP	B	402	2	-	4/18/38/38	0/3/3/3
4	ANP	E	401	2	-	2/18/38/38	0/3/3/3
4	ANP	C	402	2	-	2/18/38/38	0/3/3/3
4	ANP	D	401	2	-	6/18/38/38	0/3/3/3

All (74) bond length outliers are listed below:

Mol	Chain	Res	Type	Atoms	Z	Observed(Å)	Ideal(Å)
3	E	402	VRA	S19-N18	10.79	1.74	1.59
3	F	402	VRA	S19-N18	10.75	1.74	1.59
3	D	402	VRA	S19-N18	10.65	1.74	1.59
3	B	403	VRA	S19-N18	10.22	1.73	1.59
3	C	403	VRA	S19-N18	10.12	1.73	1.59
3	A	402	VRA	S19-N18	9.91	1.73	1.59
4	D	401	ANP	PB-O3A	8.45	1.69	1.59
4	B	402	ANP	PB-O3A	8.30	1.69	1.59
4	A	403	ANP	PB-O3A	8.29	1.69	1.59
4	E	401	ANP	PB-O3A	8.20	1.69	1.59
4	F	401	ANP	PB-O3A	8.19	1.69	1.59
4	C	402	ANP	PB-O3A	8.03	1.69	1.59
4	B	402	ANP	PG-N3B	6.18	1.79	1.63

Continued on next page...

For Manuscript Review

Continued from previous page...

Mol	Chain	Res	Type	Atoms	Z	Observed(Å)	Ideal(Å)
4	A	403	ANP	PG-N3B	6.08	1.79	1.63
4	C	402	ANP	PG-N3B	6.07	1.79	1.63
4	E	401	ANP	PG-N3B	6.03	1.79	1.63
4	F	401	ANP	PG-N3B	6.02	1.79	1.63
4	D	401	ANP	PG-N3B	5.99	1.79	1.63
3	A	402	VRA	C22-S19	4.89	1.85	1.81
4	A	403	ANP	PG-O1G	4.55	1.53	1.46
3	C	403	VRA	C22-S19	4.39	1.85	1.81
3	F	402	VRA	C22-S19	4.36	1.85	1.81
4	E	401	ANP	PG-O1G	4.27	1.52	1.46
4	B	402	ANP	PG-O1G	4.20	1.52	1.46
4	C	402	ANP	PG-O1G	4.04	1.52	1.46
3	D	402	VRA	C22-S19	3.86	1.84	1.81
4	F	401	ANP	PG-O1G	3.85	1.52	1.46
4	D	401	ANP	PG-O1G	3.82	1.52	1.46
3	C	403	VRA	C2-N7	3.43	1.49	1.39
3	B	403	VRA	C2-N7	3.30	1.49	1.39
3	A	402	VRA	C2-N7	3.28	1.49	1.39
3	B	403	VRA	O30-C10	3.26	1.42	1.37
3	F	402	VRA	C2-N7	3.14	1.48	1.39
3	E	402	VRA	O30-C10	3.12	1.42	1.37
3	B	403	VRA	C22-S19	3.10	1.84	1.81
3	E	402	VRA	C2-N7	3.01	1.48	1.39
3	E	402	VRA	C22-S19	3.01	1.84	1.81
3	A	402	VRA	O30-C10	3.00	1.41	1.37
3	D	402	VRA	C2-N7	2.99	1.48	1.39
3	F	402	VRA	O30-C10	2.94	1.41	1.37
3	D	402	VRA	O30-C10	2.84	1.41	1.37
4	A	403	ANP	PB-O1B	2.76	1.50	1.46
3	C	403	VRA	O20-S19	2.69	1.48	1.43
4	F	401	ANP	PB-O2B	-2.60	1.49	1.56
4	F	401	ANP	PB-O1B	2.60	1.50	1.46
3	C	403	VRA	C8-N7	2.59	1.49	1.40
3	C	403	VRA	O30-C10	2.55	1.41	1.37
3	D	402	VRA	C9-N18	2.51	1.49	1.43
3	C	403	VRA	O21-S19	2.49	1.47	1.43
3	A	402	VRA	C8-N7	2.49	1.48	1.40
3	E	402	VRA	C8-N7	2.48	1.48	1.40
4	C	402	ANP	PB-O1B	2.40	1.50	1.46
3	E	402	VRA	C9-N18	2.38	1.48	1.43
3	B	403	VRA	C9-N18	2.38	1.48	1.43
4	D	401	ANP	PB-O2B	-2.37	1.50	1.56

Continued on next page...

For Manuscript Review

Continued from previous page...

Mol	Chain	Res	Type	Atoms	Z	Observed(Å)	Ideal(Å)
3	F	402	VRA	C9-N18	2.34	1.48	1.43
3	A	402	VRA	O20-S19	2.33	1.47	1.43
4	E	401	ANP	PB-O2B	-2.30	1.50	1.56
3	B	403	VRA	O21-S19	2.29	1.47	1.43
3	F	402	VRA	C8-N7	2.28	1.48	1.40
3	A	402	VRA	O21-S19	2.28	1.47	1.43
3	F	402	VRA	O21-S19	2.28	1.47	1.43
3	B	403	VRA	C8-N7	2.27	1.48	1.40
3	C	403	VRA	C9-N18	2.26	1.48	1.43
3	E	402	VRA	O20-S19	2.26	1.47	1.43
4	B	402	ANP	PB-O2B	-2.24	1.50	1.56
4	C	402	ANP	PB-O2B	-2.23	1.50	1.56
3	D	402	VRA	O20-S19	2.23	1.47	1.43
4	E	401	ANP	PB-O1B	2.20	1.49	1.46
4	A	403	ANP	PB-O2B	-2.18	1.50	1.56
4	D	401	ANP	PB-O1B	2.17	1.49	1.46
4	B	402	ANP	PB-O1B	2.12	1.49	1.46
3	E	402	VRA	O21-S19	2.08	1.47	1.43
3	A	402	VRA	C9-N18	2.06	1.48	1.43

All (104) bond angle outliers are listed below:

Mol	Chain	Res	Type	Atoms	Z	Observed(°)	Ideal(°)
3	F	402	VRA	O21-S19-O20	-11.02	98.20	120.57
3	D	402	VRA	O21-S19-O20	-10.55	99.15	120.57
3	E	402	VRA	O21-S19-O20	-9.90	100.47	120.57
3	B	403	VRA	O21-S19-O20	-9.54	101.21	120.57
3	A	402	VRA	O21-S19-O20	-9.53	101.22	120.57
3	C	403	VRA	O21-S19-O20	-8.64	103.04	120.57
3	F	402	VRA	O21-S19-C22	7.15	113.34	107.60
3	A	402	VRA	O30-C10-C9	6.21	120.83	114.96
3	E	402	VRA	O30-C10-C9	6.05	120.68	114.96
3	B	403	VRA	O30-C10-C9	5.88	120.52	114.96
4	F	401	ANP	O2B-PB-O1B	5.61	121.67	109.92
4	A	403	ANP	O2B-PB-O1B	5.44	121.33	109.92
3	B	403	VRA	O21-S19-C22	5.32	111.87	107.60
4	E	401	ANP	O2B-PB-O1B	5.23	120.89	109.92
3	E	402	VRA	C22-S19-N18	5.17	116.93	107.08
4	D	401	ANP	O2B-PB-O1B	5.09	120.58	109.92
3	C	403	VRA	O30-C10-C9	4.97	119.66	114.96
3	D	402	VRA	O30-C10-C9	4.97	119.65	114.96
3	D	402	VRA	O21-S19-C22	4.90	111.53	107.60

Continued on next page...

For Manuscript Review

Continued from previous page...

Mol	Chain	Res	Type	Atoms	Z	Observed(^{o})	Ideal(^{o})
3	F	402	VRA	O30-C10-C9	4.86	119.55	114.96
4	F	401	ANP	O1G-PG-N3B	-4.75	104.77	111.77
3	A	402	VRA	O21-S19-C22	4.72	111.39	107.60
4	B	402	ANP	O2B-PB-O1B	4.70	119.77	109.92
4	C	402	ANP	O2B-PB-O1B	4.69	119.76	109.92
3	D	402	VRA	O20-S19-C22	4.53	111.23	107.60
4	E	401	ANP	O1G-PG-N3B	-4.27	105.48	111.77
4	B	402	ANP	O1G-PG-N3B	-4.12	105.70	111.77
4	C	402	ANP	O1G-PG-N3B	-3.96	105.95	111.77
4	D	401	ANP	O5'-C5'-C4'	3.89	122.37	108.99
3	C	403	VRA	O21-S19-C22	3.74	110.60	107.60
3	C	403	VRA	O20-S19-C22	3.70	110.57	107.60
3	A	402	VRA	O20-S19-C22	3.69	110.56	107.60
4	F	401	ANP	O5'-C5'-C4'	3.58	121.30	108.99
4	A	403	ANP	O1G-PG-N3B	-3.56	106.53	111.77
4	D	401	ANP	O1G-PG-N3B	-3.54	106.55	111.77
3	D	402	VRA	C6-C1-C2	-3.51	120.16	123.50
3	C	403	VRA	C31-O30-C10	-3.39	112.41	117.53
3	F	402	VRA	C31-O30-C10	-3.38	112.42	117.53
3	A	402	VRA	C22-S19-N18	3.36	113.50	107.08
3	D	402	VRA	C31-O30-C10	-3.31	112.53	117.53
4	D	401	ANP	O2G-PG-O3G	3.23	116.25	107.64
4	E	401	ANP	O2G-PG-O3G	3.16	116.06	107.64
4	D	401	ANP	O3'-C3'-C2'	3.13	121.96	111.82
3	A	402	VRA	C23-C22-C25	-3.11	112.14	116.76
4	A	403	ANP	O3'-C3'-C2'	3.02	121.60	111.82
3	E	402	VRA	C6-C1-C2	-2.98	120.66	123.50
4	C	402	ANP	O2G-PG-O3G	2.98	115.56	107.64
4	A	403	ANP	O2G-PG-O3G	2.91	115.39	107.64
3	E	402	VRA	C31-O30-C10	-2.90	113.15	117.53
3	A	402	VRA	O30-C10-C11	-2.89	119.14	124.12
3	F	402	VRA	C22-S19-N18	2.88	112.58	107.08
4	A	403	ANP	O4'-C1'-N9	2.88	113.73	108.06
4	C	402	ANP	PB-O3A-PA	-2.87	122.52	132.62
4	A	403	ANP	PB-O3A-PA	-2.85	122.57	132.62
4	A	403	ANP	O4'-C4'-C5'	-2.85	100.00	109.37
4	D	401	ANP	O4'-C1'-N9	2.79	113.56	108.06
4	F	401	ANP	PB-O3A-PA	-2.76	122.88	132.62
4	E	401	ANP	PB-O3A-PA	-2.76	122.91	132.62
4	C	402	ANP	O3A-PB-N3B	-2.75	98.96	106.59
3	E	402	VRA	C10-C9-N18	2.73	123.71	118.38
3	E	402	VRA	F15-C1-C2	2.73	120.92	117.50

Continued on next page...

For Manuscript Review

Continued from previous page...

Mol	Chain	Res	Type	Atoms	Z	Observed(°)	Ideal(°)
3	B	403	VRA	O30-C10-C11	-2.73	119.43	124.12
3	C	403	VRA	O30-C10-C11	-2.70	119.48	124.12
3	A	402	VRA	C31-O30-C10	-2.61	113.59	117.53
4	B	402	ANP	O2'-C2'-C3'	-2.58	103.47	111.82
4	F	401	ANP	O3'-C3'-C2'	2.57	120.13	111.82
4	A	403	ANP	C5'-C4'-C3'	2.55	124.74	115.18
4	F	401	ANP	O2G-PG-O3G	2.55	114.42	107.64
3	B	403	VRA	C4-C5-C6	-2.53	118.11	121.09
3	D	402	VRA	O30-C10-C11	-2.51	119.81	124.12
4	A	403	ANP	O3'-C3'-C4'	-2.50	103.81	111.05
3	D	402	VRA	C22-S19-N18	2.49	111.83	107.08
3	C	403	VRA	F15-C1-C2	2.43	120.55	117.50
4	A	403	ANP	C2'-C3'-C4'	-2.42	97.94	102.64
3	D	402	VRA	C3-C2-C1	2.40	119.60	117.17
4	B	402	ANP	O2G-PG-O3G	2.39	114.01	107.64
3	E	402	VRA	O30-C10-C11	-2.36	120.06	124.12
3	F	402	VRA	C6-C1-C2	-2.34	121.27	123.50
3	E	402	VRA	C24-C22-C25	2.34	120.23	116.76
3	F	402	VRA	O30-C10-C11	-2.33	120.11	124.12
3	F	402	VRA	C4-C5-C6	-2.28	118.41	121.09
3	A	402	VRA	C4-C5-I14	2.25	123.08	119.68
3	E	402	VRA	C23-C22-C25	-2.25	113.42	116.76
3	F	402	VRA	O20-S19-N18	2.25	113.48	109.42
3	E	402	VRA	O21-S19-C22	2.24	109.40	107.60
3	C	403	VRA	C22-S19-N18	2.23	111.33	107.08
4	B	402	ANP	PB-O3A-PA	-2.22	124.79	132.62
3	C	403	VRA	C9-N18-S19	-2.20	119.74	125.40
3	D	402	VRA	F15-C1-C2	2.19	120.25	117.50
4	A	403	ANP	O3A-PB-N3B	-2.18	100.55	106.59
3	B	403	VRA	C4-C5-I14	2.14	122.92	119.68
3	B	403	VRA	C22-S19-N18	2.14	111.17	107.08
4	F	401	ANP	O3A-PB-N3B	-2.11	100.73	106.59
4	D	401	ANP	O3'-C3'-C4'	-2.11	104.96	111.05
3	A	402	VRA	C4-C5-C6	-2.10	118.62	121.09
3	F	402	VRA	F17-C12-C13	2.08	121.22	118.30
4	E	401	ANP	O3A-PB-N3B	-2.06	100.88	106.59
4	B	402	ANP	C1'-N9-C8	2.06	131.78	127.14
4	F	401	ANP	C1'-N9-C8	2.05	131.77	127.14
4	D	401	ANP	C1'-N9-C8	2.03	131.72	127.14
4	B	402	ANP	C4-N9-C1'	-2.03	121.76	126.59
3	C	403	VRA	F17-C12-C13	2.03	121.15	118.30
4	A	403	ANP	O4'-C4'-C3'	2.02	109.11	105.11

Continued on next page...

For Manuscript Review

Continued from previous page...

Mol	Chain	Res	Type	Atoms	Z	Observed(^{o})	Ideal(^{o})
4	D	401	ANP	O3A-PB-N3B	-2.00	101.03	106.59

There are no chirality outliers.

All (61) torsion outliers are listed below:

Mol	Chain	Res	Type	Atoms
3	A	402	VRA	C1-C2-N7-C8
3	A	402	VRA	C9-C10-O30-C31
3	A	402	VRA	C25-C26-C27-O28
3	C	403	VRA	C1-C2-N7-C8
3	C	403	VRA	C9-N18-S19-O20
3	E	402	VRA	C1-C2-N7-C8
3	E	402	VRA	C10-C9-N18-S19
3	E	402	VRA	C9-N18-S19-O21
3	E	402	VRA	C9-N18-S19-C22
4	A	403	ANP	PB-N3B-PG-O1G
4	B	402	ANP	PB-N3B-PG-O1G
4	B	402	ANP	PG-N3B-PB-O1B
4	C	402	ANP	PB-N3B-PG-O1G
4	C	402	ANP	PG-N3B-PB-O1B
4	D	401	ANP	PB-N3B-PG-O1G
4	D	401	ANP	PG-N3B-PB-O1B
4	D	401	ANP	PG-N3B-PB-O3A
4	E	401	ANP	PG-N3B-PB-O1B
4	F	401	ANP	PB-N3B-PG-O1G
4	F	401	ANP	PG-N3B-PB-O1B
4	F	401	ANP	PG-N3B-PB-O3A
3	C	403	VRA	C9-C10-O30-C31
3	D	402	VRA	C9-C10-O30-C31
3	E	402	VRA	C3-C2-N7-C8
3	B	403	VRA	C9-C10-O30-C31
3	F	402	VRA	C9-C10-O30-C31
3	E	402	VRA	C11-C10-O30-C31
3	D	402	VRA	C11-C10-O30-C31
3	E	402	VRA	C9-C10-O30-C31
3	C	403	VRA	C11-C10-O30-C31
3	A	402	VRA	C3-C2-N7-C8
3	A	402	VRA	C11-C10-O30-C31
3	F	402	VRA	C11-C10-O30-C31
3	B	403	VRA	C11-C10-O30-C31
3	C	403	VRA	C3-C2-N7-C8
3	E	402	VRA	C8-C9-N18-S19

Continued on next page...

For Manuscript Review

Continued from previous page...

Mol	Chain	Res	Type	Atoms
4	D	401	ANP	C4'-C5'-O5'-PA
4	B	402	ANP	O4'-C4'-C5'-O5'
4	F	401	ANP	C4'-C5'-O5'-PA
3	A	402	VRA	O29-C26-C27-O28
3	C	403	VRA	O29-C26-C27-O28
4	E	401	ANP	PB-O3A-PA-O5'
3	D	402	VRA	C9-N18-S19-O21
3	A	402	VRA	C9-C8-N7-C2
3	E	402	VRA	C9-C8-N7-C2
3	C	403	VRA	C22-C25-C26-O29
4	D	401	ANP	O4'-C4'-C5'-O5'
4	F	401	ANP	O4'-C4'-C5'-O5'
3	B	403	VRA	C9-C8-N7-C2
3	D	402	VRA	C9-C8-N7-C2
3	C	403	VRA	C9-N18-S19-C22
4	A	403	ANP	PG-N3B-PB-O1B
3	B	403	VRA	O29-C26-C27-O28
3	C	403	VRA	C9-C8-N7-C2
3	F	402	VRA	C9-C8-N7-C2
3	C	403	VRA	C25-C26-C27-O28
4	D	401	ANP	C3'-C4'-C5'-O5'
4	F	401	ANP	C3'-C4'-C5'-O5'
3	B	403	VRA	C9-N18-S19-O20
4	A	403	ANP	O4'-C4'-C5'-O5'
4	B	402	ANP	C5'-O5'-PA-O1A

There are no ring outliers.

8 monomers are involved in 22 short contacts:

Mol	Chain	Res	Type	Clashes	Symm-Clashes
3	E	402	VRA	3	0
3	D	402	VRA	4	0
3	F	402	VRA	3	0
3	A	402	VRA	3	0
4	F	401	ANP	4	0
4	B	402	ANP	1	0
4	C	402	ANP	1	0
4	D	401	ANP	3	0

The following is a two-dimensional graphical depiction of Mogul quality analysis of bond lengths, bond angles, torsion angles, and ring geometry for all instances of the Ligand of Interest. In addition, ligands with molecular weight > 250 and outliers as shown on the validation Tables will

For Manuscript Review

also be included. For torsion angles, if less then 5% of the Mogul distribution of torsion angles is within 10 degrees of the torsion angle in question, then that torsion angle is considered an outlier. Any bond that is central to one or more torsion angles identified as an outlier by Mogul will be highlighted in the graph. For rings, the root-mean-square deviation (RMSD) between the ring in question and similar rings identified by Mogul is calculated over all ring torsion angles. If the average RMSD is greater than 60 degrees and the minimal RMSD between the ring in question and any Mogul-identified rings is also greater than 60 degrees, then that ring is considered an outlier. The outliers are highlighted in purple. The color gray indicates Mogul did not find sufficient equivalents in the CSD to analyse the geometry.

**Ligand VRA E 402**	
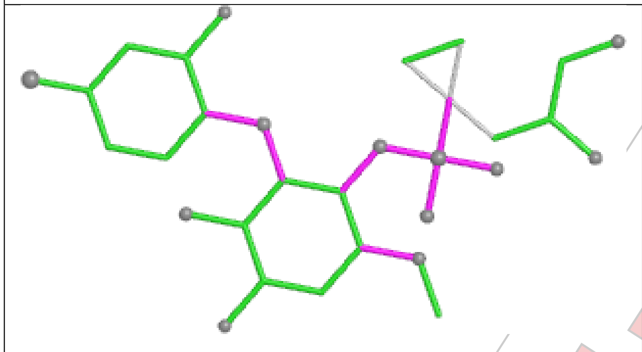 **Bond lengths**	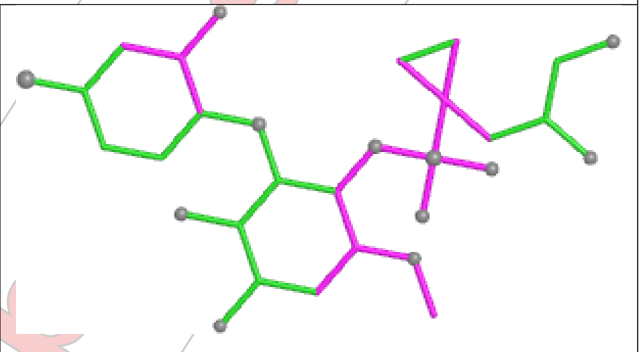 **Bond angles**
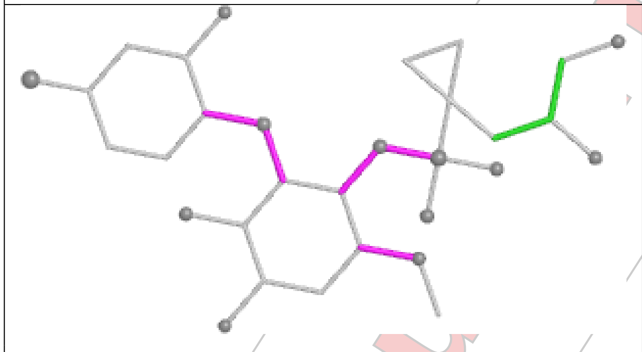 **Torsions**	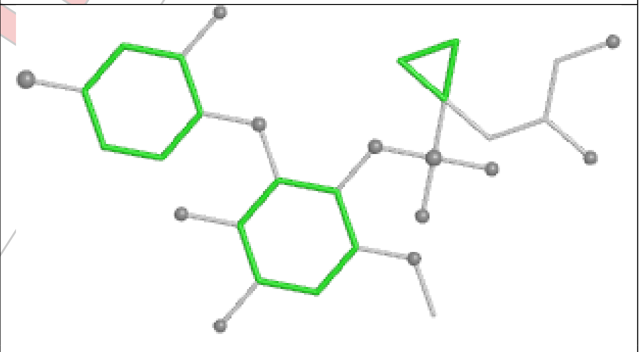 **Rings**

For Manuscript Review

Ligand VRA D 402

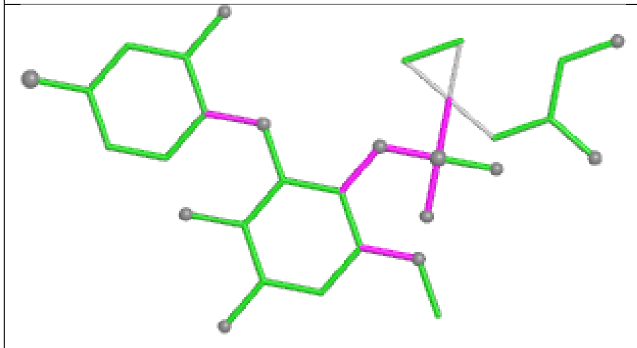

Bond lengths

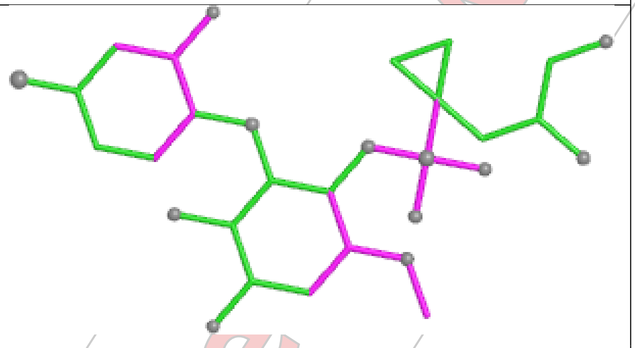

Bond angles

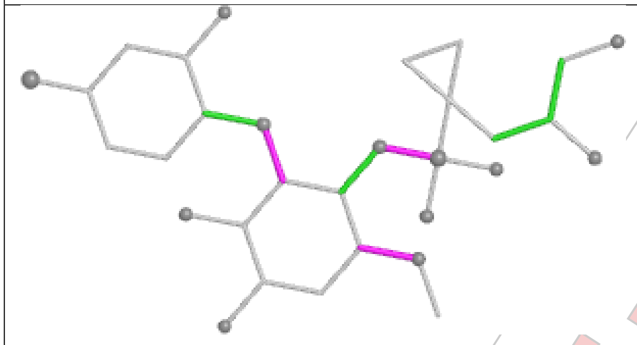

Torsions

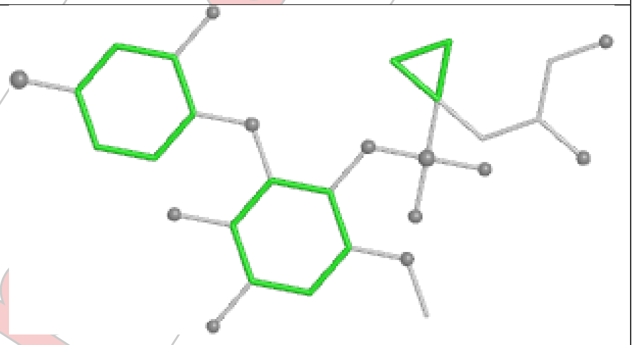

Rings

Ligand VRA F 402

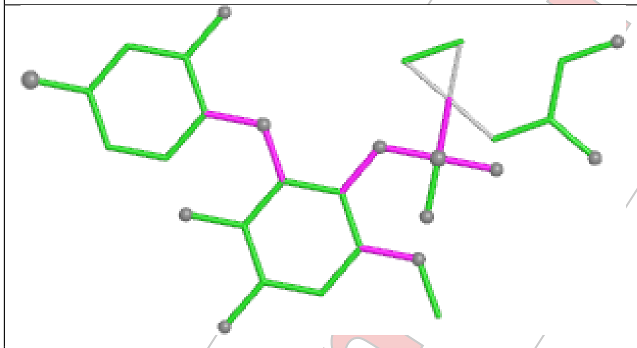

Bond lengths

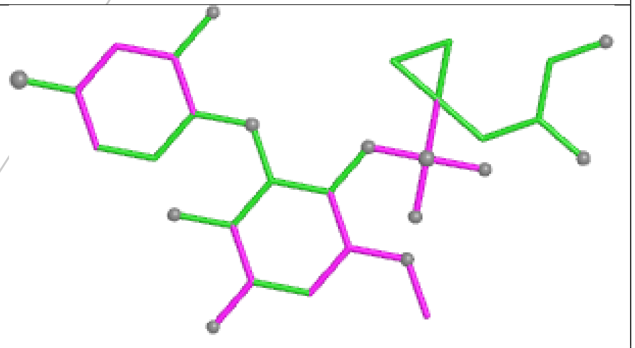

Bond angles

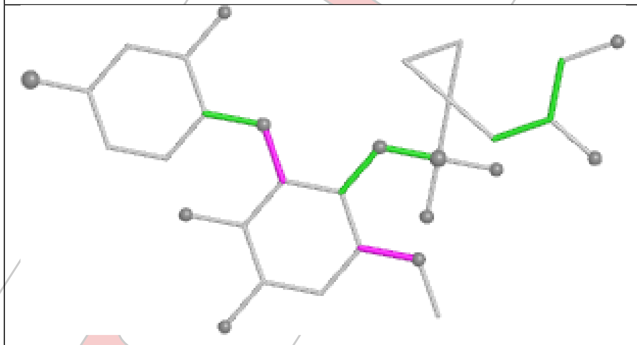

Torsions

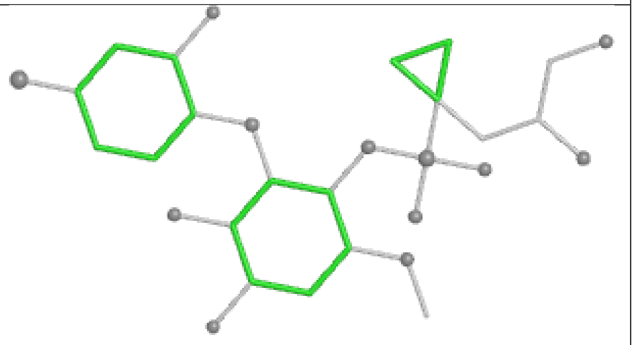

Rings

Ligand VRA C 403

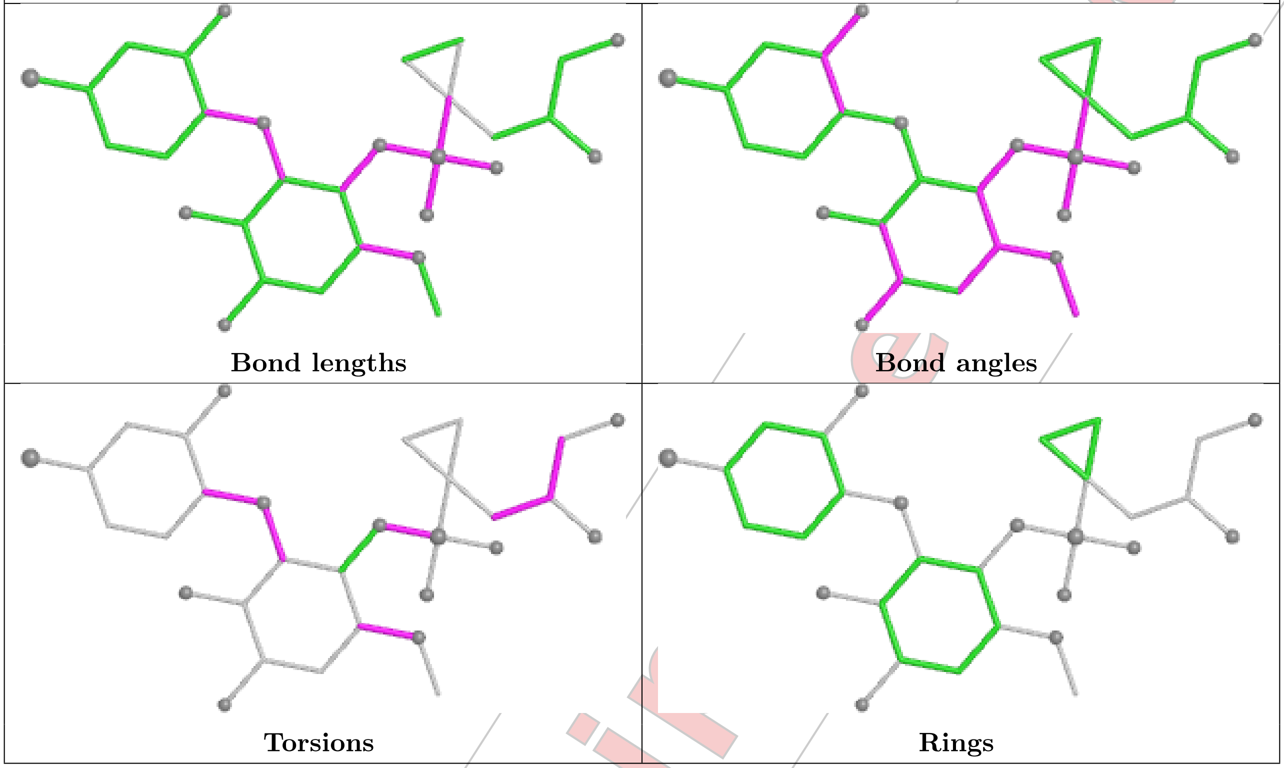

Ligand VRA B 403

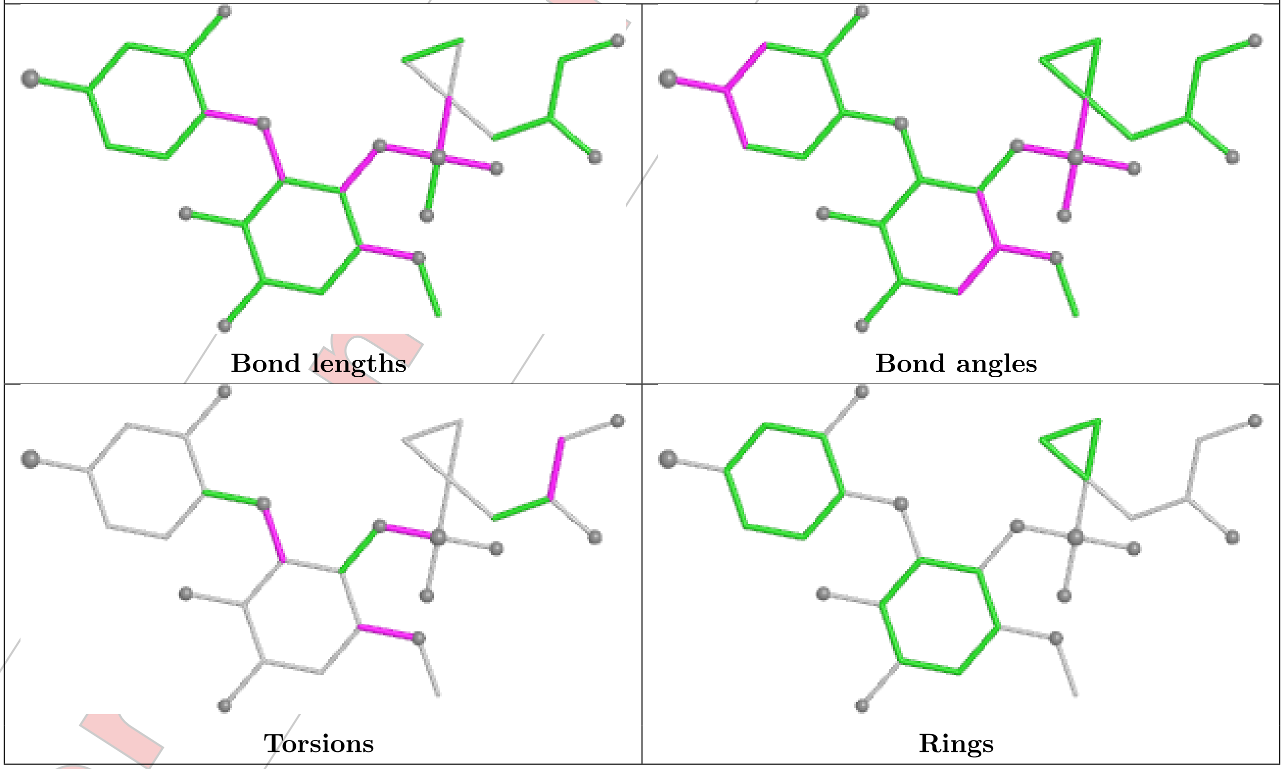

Ligand VRA A 402

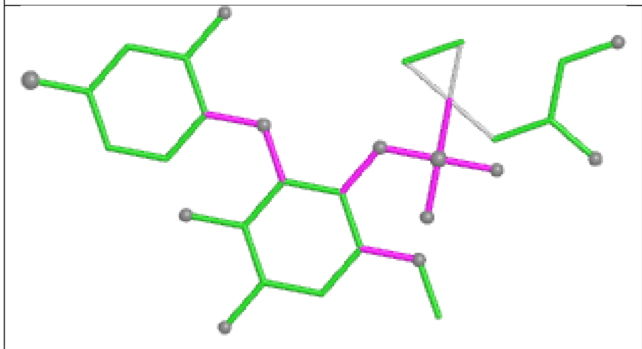

Bond lengths

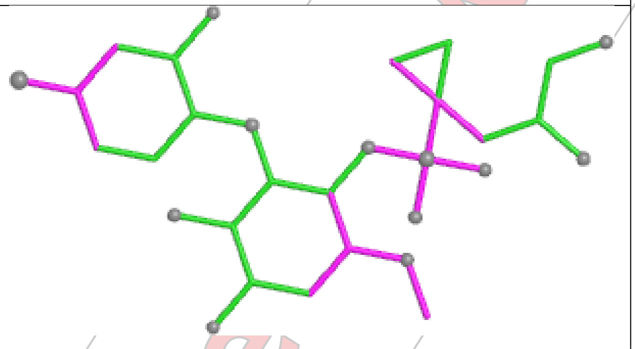

Bond angles

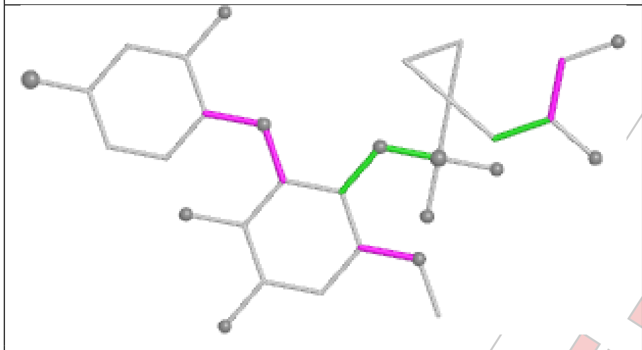

Torsions

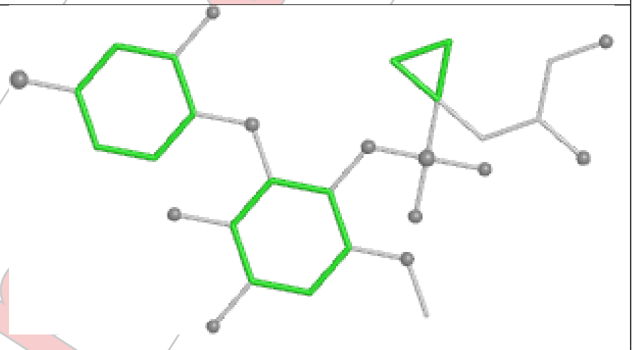

Rings

Ligand ANP A 403

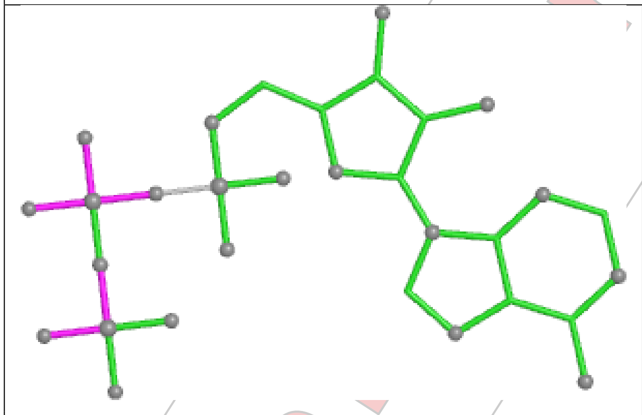

Bond lengths

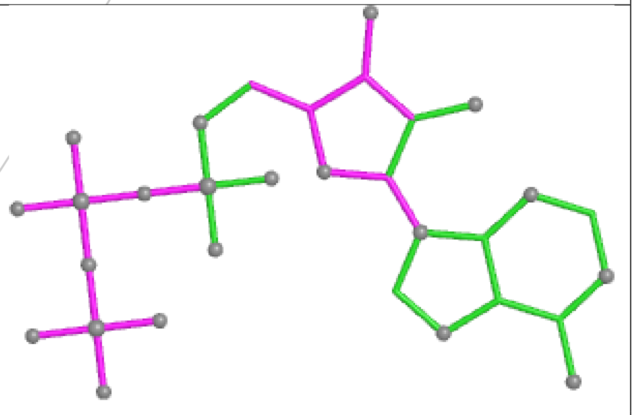

Bond angles

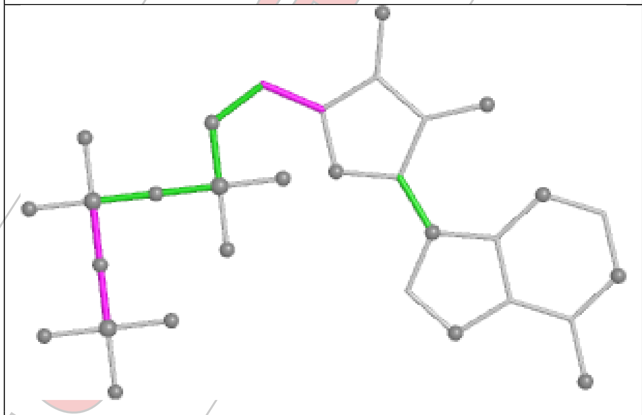

Torsions

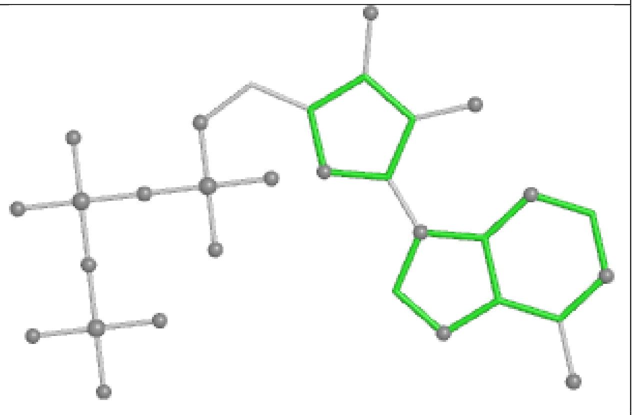

Rings

Ligand ANP F 401

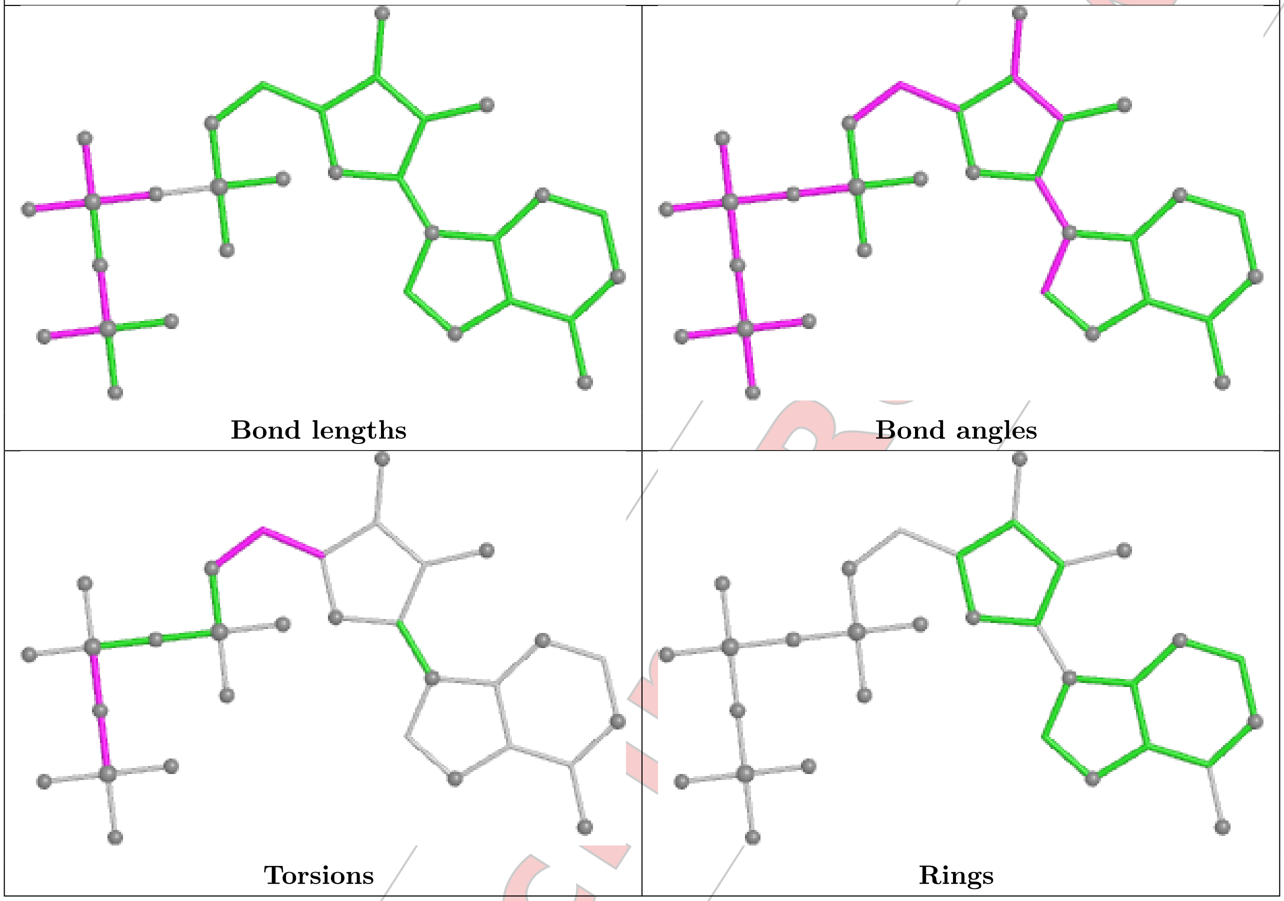

Bond lengths | Bond angles | Torsions | Rings

For Manuscript Review

Ligand ANP B 402

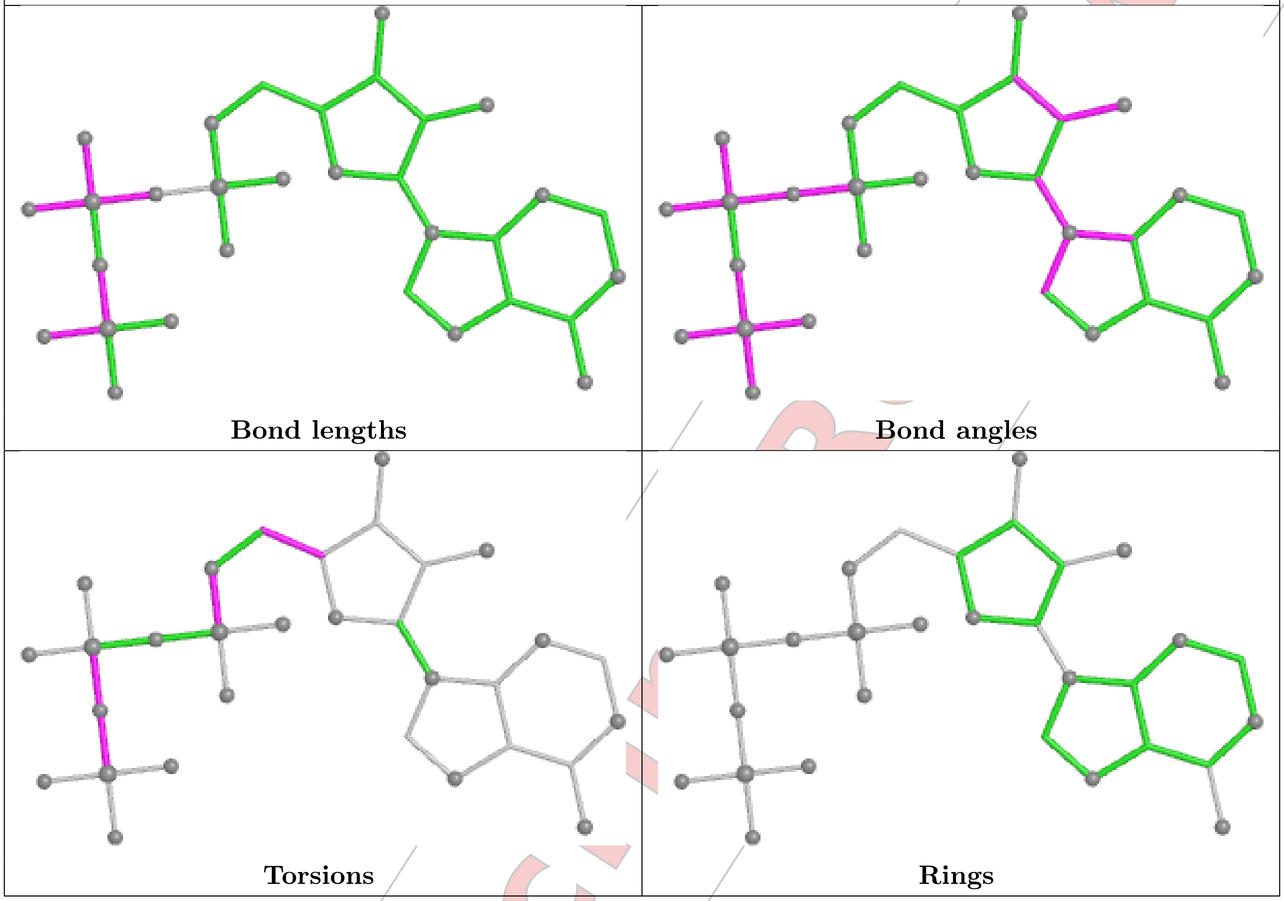

For Manuscript Review

Ligand ANP E 401

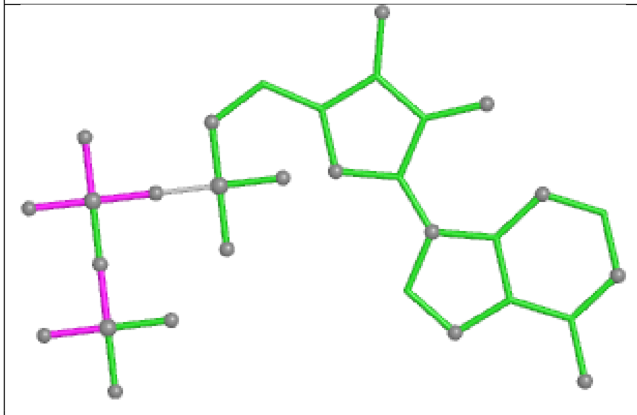

Bond lengths

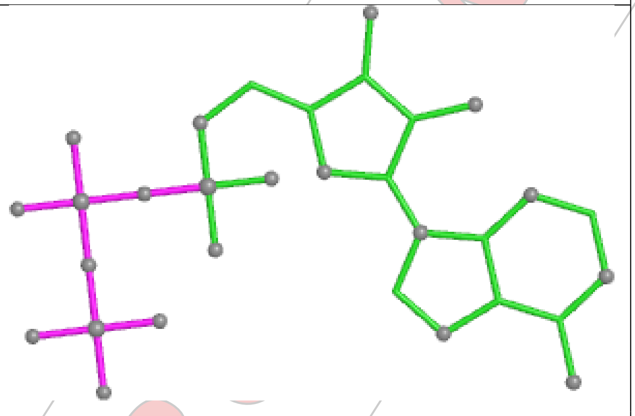

Bond angles

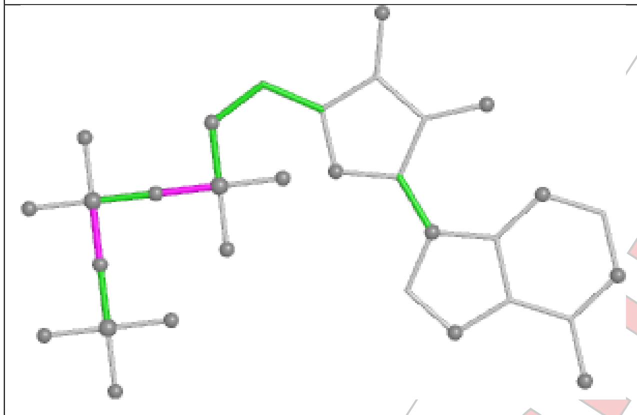

Torsions

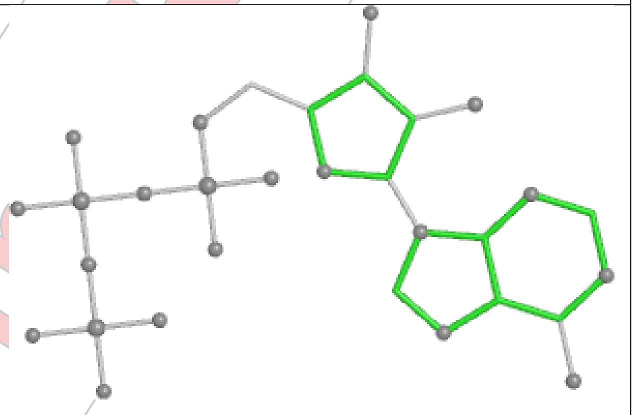

Rings

For Manuscript Review

Ligand ANP C 402

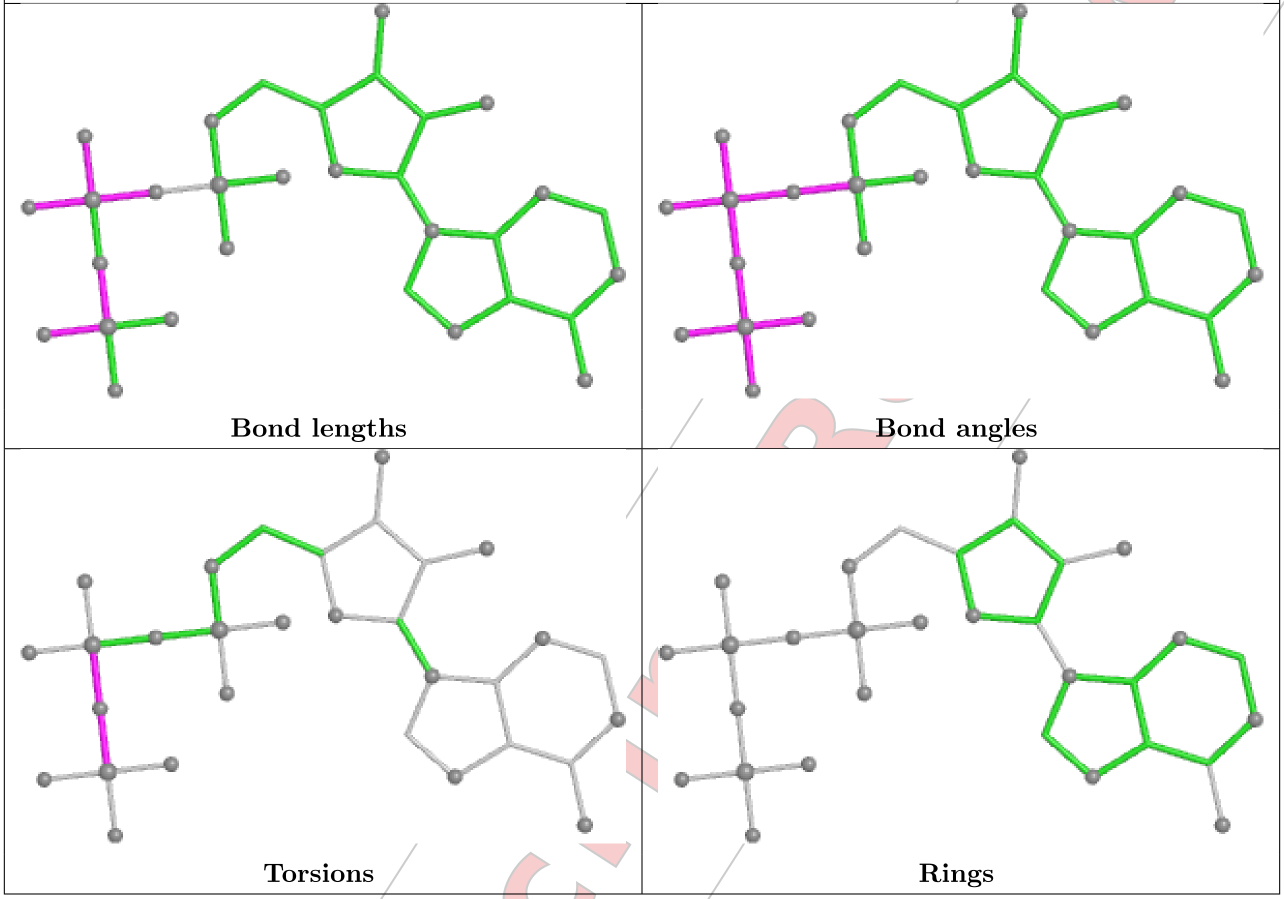

For Manuscript Review

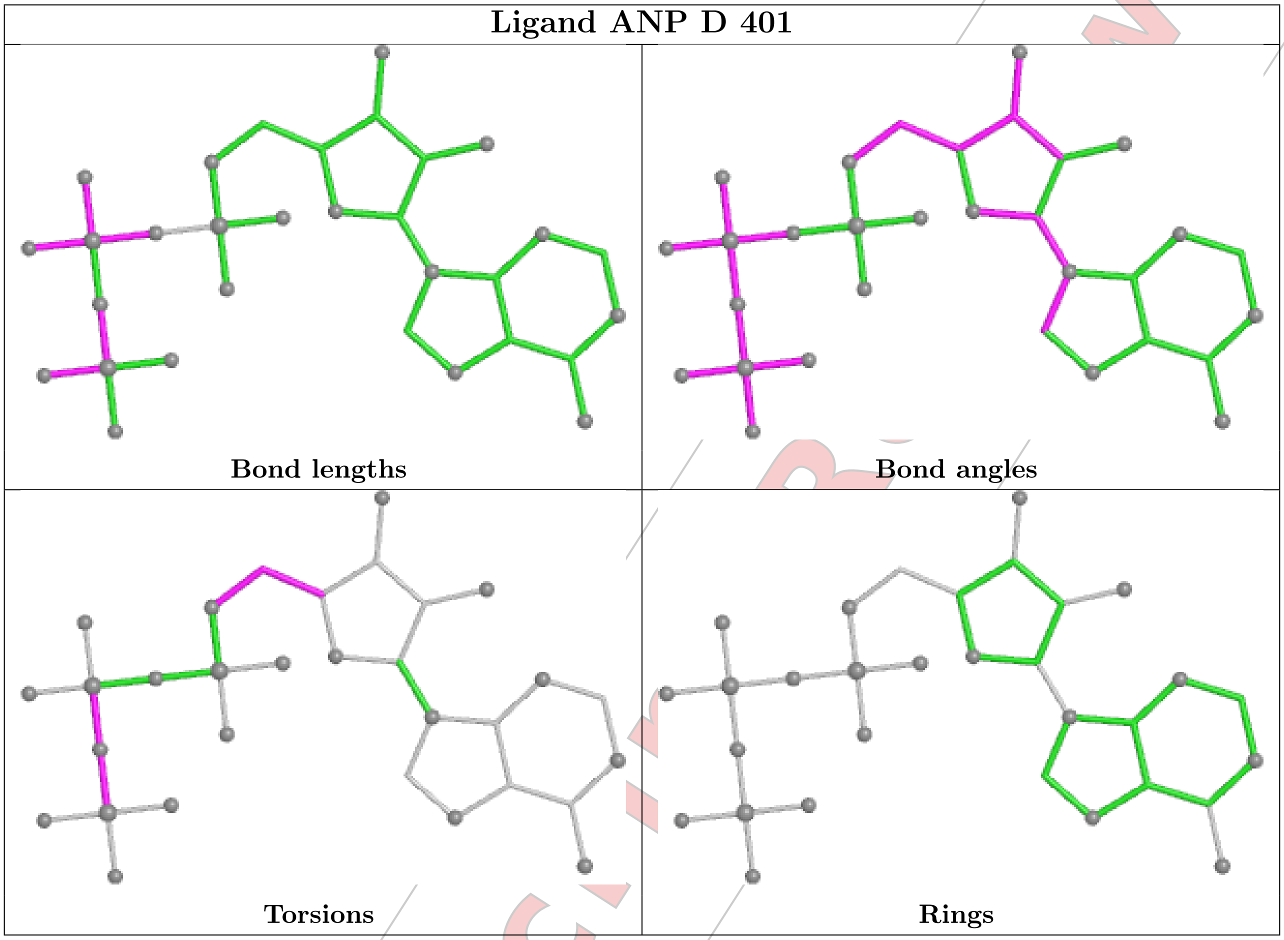

5.7 Other polymers (i)

There are no such residues in this entry.

5.8 Polymer linkage issues (i)

There are no chain breaks in this entry.

For Manuscript Review

6 Fit of model and data (i)

6.1 Protein, DNA and RNA chains (i)

In the following table, the column labelled '#RSRZ> 2' contains the number (and percentage) of RSRZ outliers, followed by percent RSRZ outliers for the chain as percentile scores relative to all X-ray entries and entries of similar resolution. The OWAB column contains the minimum, median, 95th percentile and maximum values of the occupancy-weighted average B-factor per residue. The column labelled 'Q< 0.9' lists the number of (and percentage) of residues with an average occupancy less than 0.9.

Mol	Chain	Analysed	<RSRZ>	#RSRZ>2			OWAB(Å^2)	Q<0.9
1	A	307/346 (88%)	0.10	4 (1%)	75	56	29, 48, 74, 111	0
1	B	307/346 (88%)	0.24	6 (1%)	65	46	31, 52, 77, 99	0
1	C	302/346 (87%)	0.41	9 (2%)	52	35	28, 59, 90, 109	0
1	D	300/346 (86%)	0.47	11 (3%)	45	31	33, 59, 87, 103	0
1	E	302/346 (87%)	0.30	7 (2%)	61	42	29, 52, 77, 104	0
1	F	302/346 (87%)	0.41	13 (4%)	40	25	34, 55, 83, 99	0
All	All	1820/2076 (87%)	0.32	50 (2%)	56	37	28, 53, 84, 111	0

All (50) RSRZ outliers are listed below:

Mol	Chain	Res	Type	RSRZ
1	D	279	PHE	3.9
1	E	113	ASN	3.3
1	D	326	VAL	3.3
1	E	244	TYR	3.3
1	F	267	ILE	3.3
1	D	113	ASN	3.2
1	F	279	PHE	3.1
1	B	156	ASP	2.9
1	C	319	PHE	2.9
1	F	243	HIS	2.9
1	A	243	HIS	2.8
1	E	104	HIS	2.8
1	C	73	GLU	2.8
1	A	242	THR	2.8
1	E	267	ILE	2.8
1	B	44	LEU	2.7
1	F	230	THR	2.6
1	D	156	ASP	2.5
1	F	113	ASN	2.5

Continued on next page...

For Manuscript Review

Continued from previous page...

Mol	Chain	Res	Type	RSRZ
1	F	151	ASP	2.5
1	C	324	TYR	2.5
1	D	267	ILE	2.4
1	F	318	ILE	2.4
1	E	271	ASP	2.4
1	F	268	PRO	2.4
1	B	244	TYR	2.4
1	B	224	ALA	2.3
1	C	242	THR	2.3
1	E	243	HIS	2.3
1	F	104	HIS	2.3
1	D	384	CYS	2.3
1	E	335	ASN	2.3
1	D	107	ILE	2.2
1	D	331	PRO	2.2
1	B	243	HIS	2.2
1	F	244	TYR	2.2
1	D	319	PHE	2.2
1	F	83	GLY	2.2
1	C	337	VAL	2.2
1	A	48	GLU	2.2
1	F	168	GLU	2.2
1	C	267	ILE	2.2
1	B	69	ASP	2.2
1	D	389	LEU	2.1
1	C	328	GLU	2.1
1	C	105	LEU	2.0
1	A	47	ASP	2.0
1	F	75	ILE	2.0
1	C	269	PRO	2.0
1	D	75	ILE	2.0

6.2 Non-standard residues in protein, DNA, RNA chains (i)

There are no non-standard protein/DNA/RNA residues in this entry.

6.3 Carbohydrates (i)

There are no oligosaccharides in this entry.

For Manuscript Review

6.4 Ligands ⓘ

In the following table, the Atoms column lists the number of modelled atoms in the group and the number defined in the chemical component dictionary. The B-factors column lists the minimum, median, 95th percentile and maximum values of B factors of atoms in the group. The column labelled 'Q< 0.9' lists the number of atoms with occupancy less than 0.9.

Mol	Type	Chain	Res	Atoms	RSCC	RSR	B-factors($Å^2$)	Q<0.9
2	MG	E	403	1/1	0.85	0.21	49,49,49,49	0
2	MG	D	403	1/1	0.91	0.21	46,46,46,46	0
4	ANP	D	401	31/31	0.92	0.11	42,65,79,86	0
4	ANP	F	401	31/31	0.92	0.10	44,54,65,70	0
4	ANP	E	401	31/31	0.93	0.10	44,56,68,77	0
4	ANP	B	402	31/31	0.93	0.11	41,52,65,83	0
4	ANP	C	402	31/31	0.94	0.10	44,61,73,77	0
2	MG	A	401	1/1	0.94	0.09	37,37,37,37	0
4	ANP	A	403	31/31	0.95	0.10	35,45,57,64	0
2	MG	F	403	1/1	0.95	0.09	52,52,52,52	0
3	VRA	E	402	31/31	0.95	0.12	48,71,91,109	0
3	VRA	C	403	31/31	0.96	0.13	49,75,101,108	0
3	VRA	A	402	31/31	0.96	0.11	41,58,74,90	0
3	VRA	F	402	31/31	0.96	0.12	47,65,81,89	0
2	MG	C	401	1/1	0.97	0.10	59,59,59,59	0
3	VRA	B	403	31/31	0.97	0.10	44,60,83,85	0
2	MG	B	401	1/1	0.97	0.15	41,41,41,41	0
3	VRA	D	402	31/31	0.97	0.09	49,68,85,88	0

The following is a graphical depiction of the model fit to experimental electron density of all instances of the Ligand of Interest. In addition, ligands with molecular weight > 250 and outliers as shown on the geometry validation Tables will also be included. Each fit is shown from different orientation to approximate a three-dimensional view.

For Manuscript Review

Electron density around ANP D 401:

$2mF_o$-DF_c (at 0.7 rmsd) in gray
mF_o-DF_c (at 3 rmsd) in purple (negative)
and green (positive)

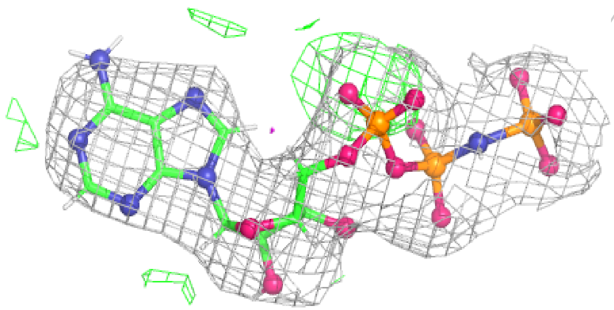

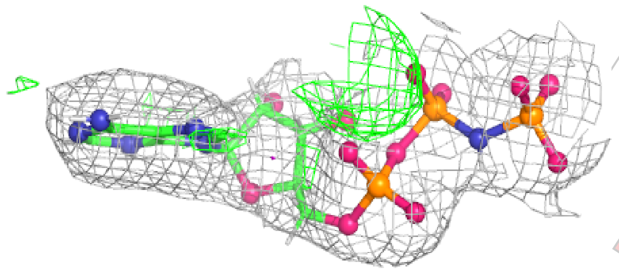

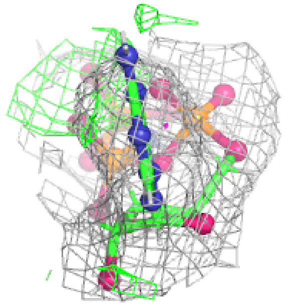

Electron density around ANP F 401:

$2mF_o$-DF_c (at 0.7 rmsd) in gray
mF_o-DF_c (at 3 rmsd) in purple (negative)
and green (positive)

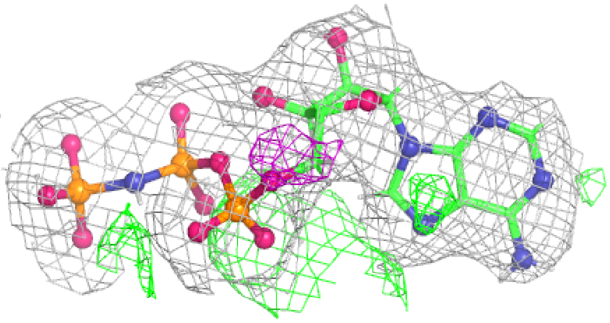

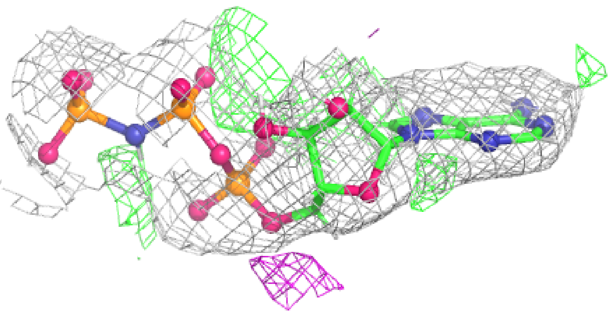

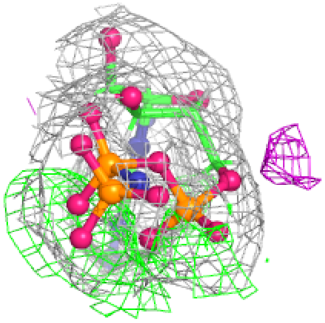

For Manuscript Review

Electron density around ANP E 401:

2mF$_o$-DF$_c$ (at 0.7 rmsd) in gray
mF$_o$-DF$_c$ (at 3 rmsd) in purple (negative)
and green (positive)

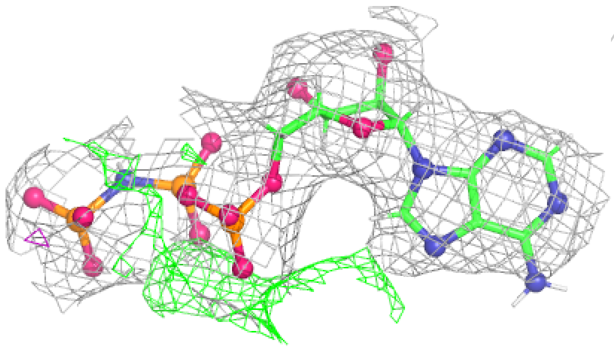

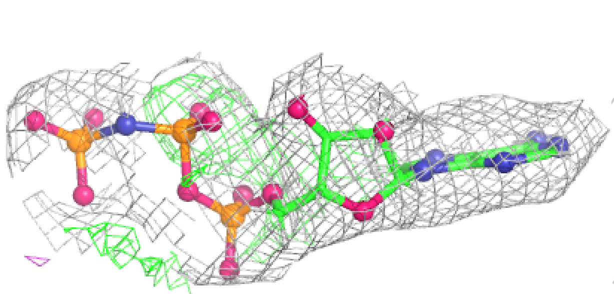

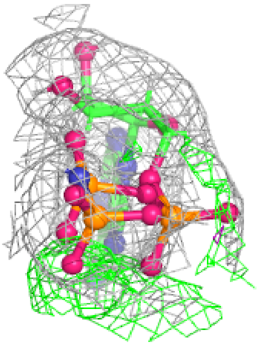

Electron density around ANP B 402:

2mF$_o$-DF$_c$ (at 0.7 rmsd) in gray
mF$_o$-DF$_c$ (at 3 rmsd) in purple (negative)
and green (positive)

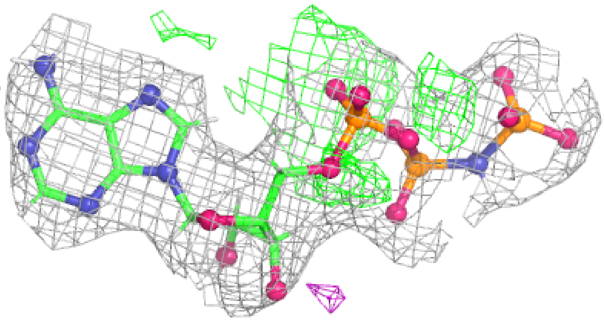

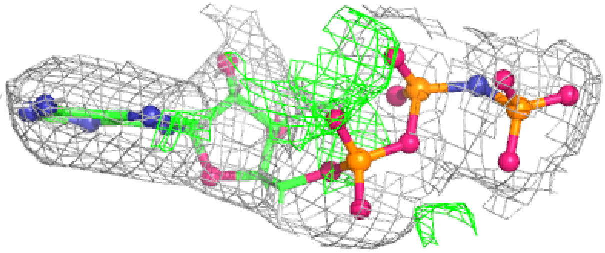

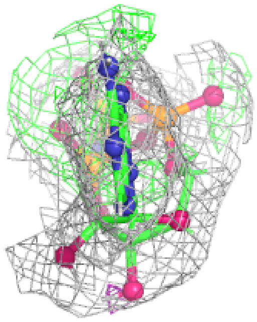

For Manuscript Review

Electron density around ANP C 402:

2mF$_o$-DF$_c$ (at 0.7 rmsd) in gray
mF$_o$-DF$_c$ (at 3 rmsd) in purple (negative)
and green (positive)

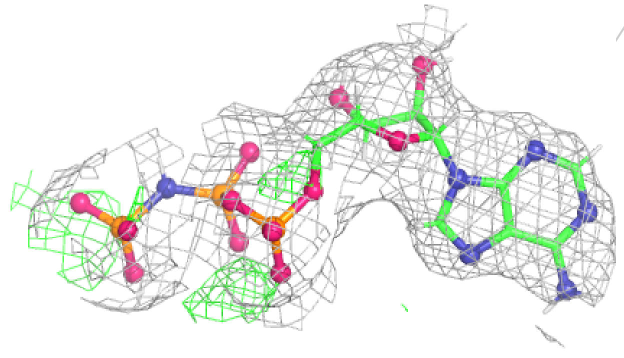

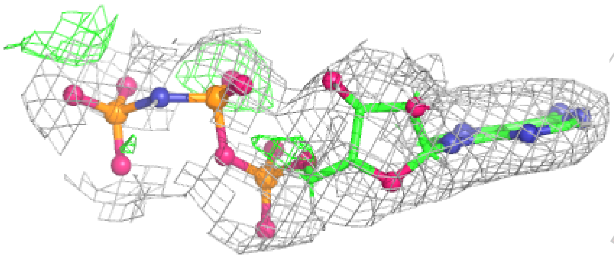

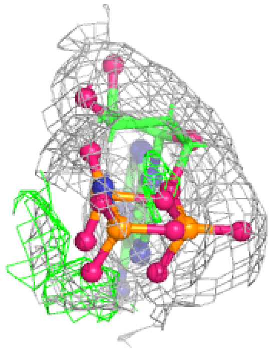

Electron density around ANP A 403:

2mF$_o$-DF$_c$ (at 0.7 rmsd) in gray
mF$_o$-DF$_c$ (at 3 rmsd) in purple (negative)
and green (positive)

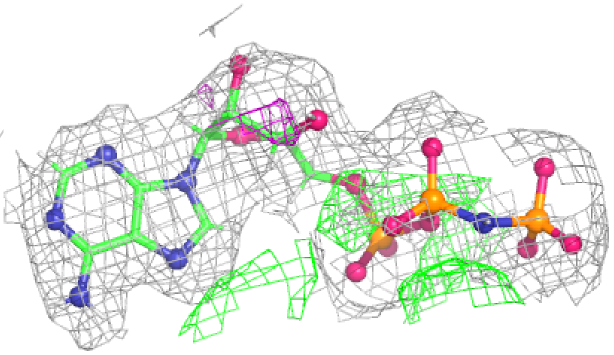

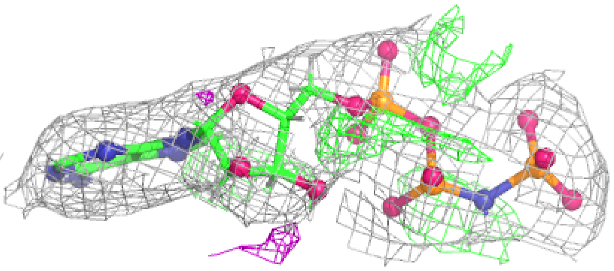

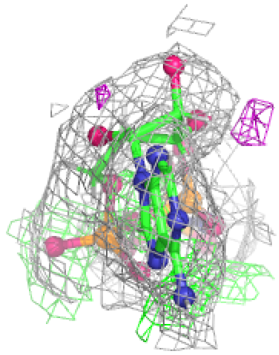

Electron density around VRA E 402:

$2mF_o$-DF_c (at 0.7 rmsd) in gray
mF_o-DF_c (at 3 rmsd) in purple (negative)
and green (positive)

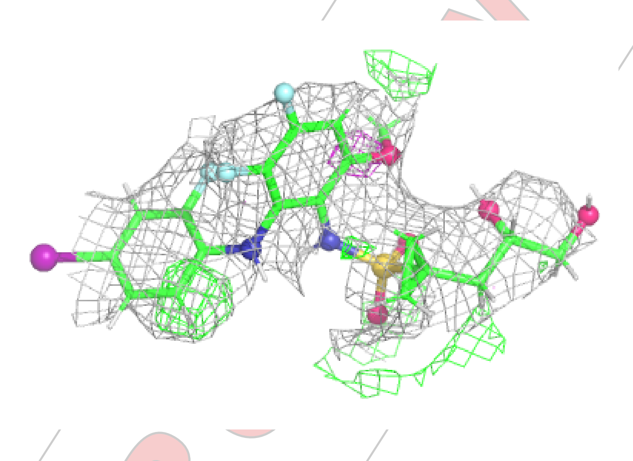

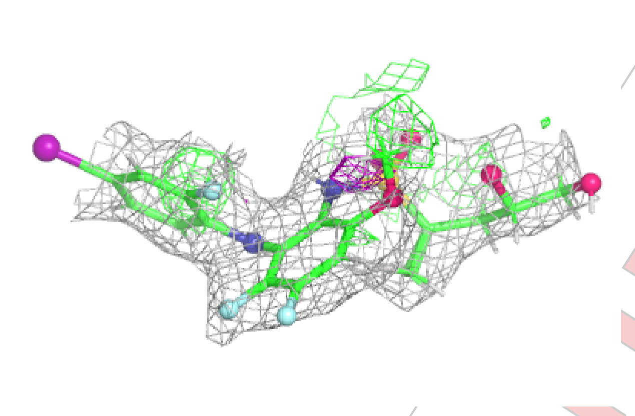

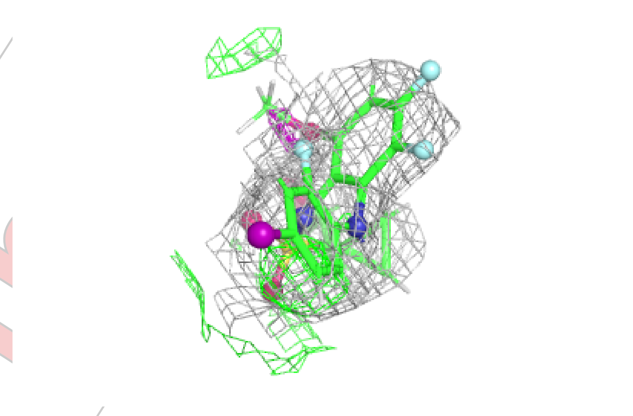

Electron density around VRA C 403:

$2mF_o$-DF_c (at 0.7 rmsd) in gray
mF_o-DF_c (at 3 rmsd) in purple (negative)
and green (positive)

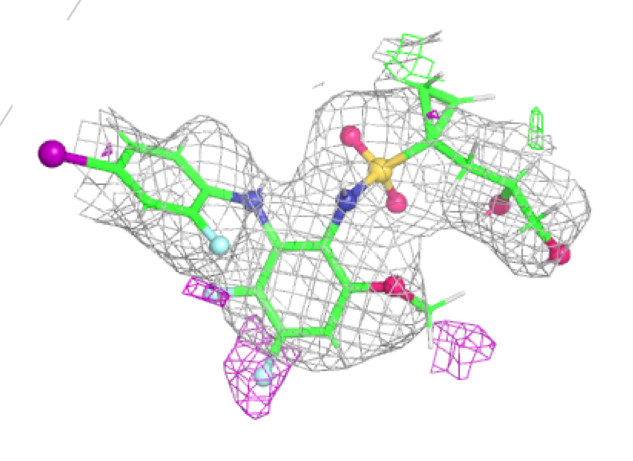

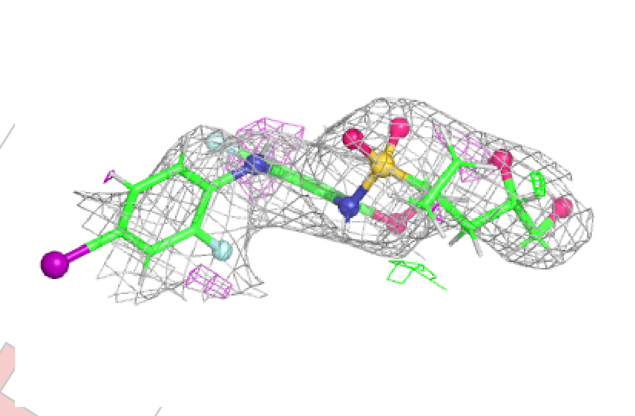

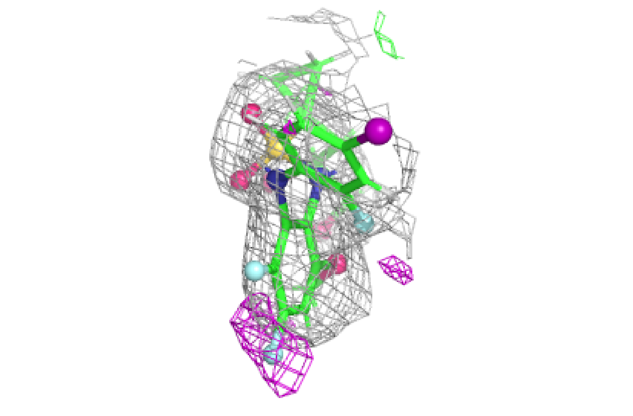

Electron density around VRA A 402:

$2mF_o$-DF_c (at 0.7 rmsd) in gray
mF_o-DF_c (at 3 rmsd) in purple (negative)
and green (positive)

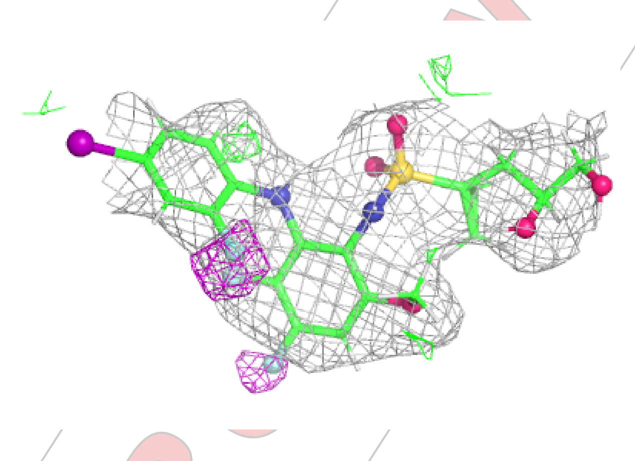

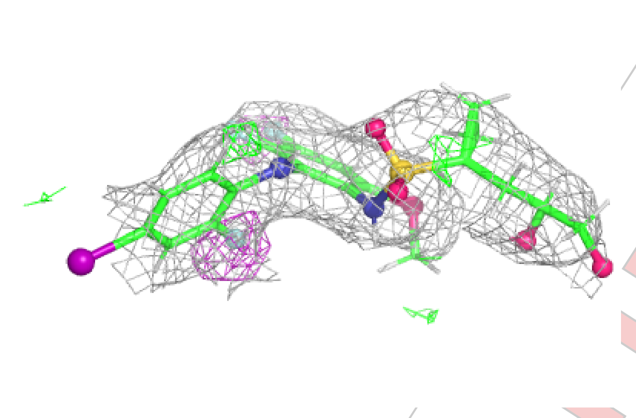

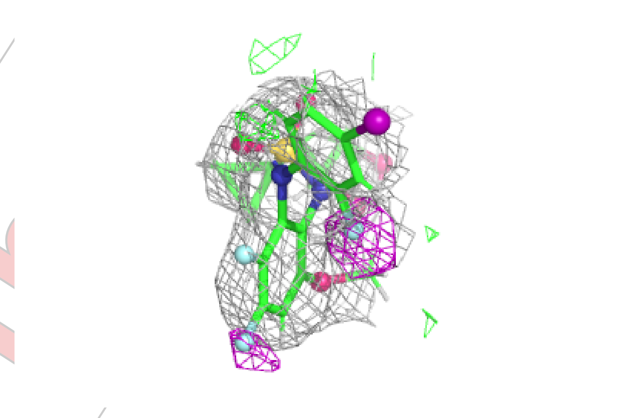

Electron density around VRA F 402:

$2mF_o$-DF_c (at 0.7 rmsd) in gray
mF_o-DF_c (at 3 rmsd) in purple (negative)
and green (positive)

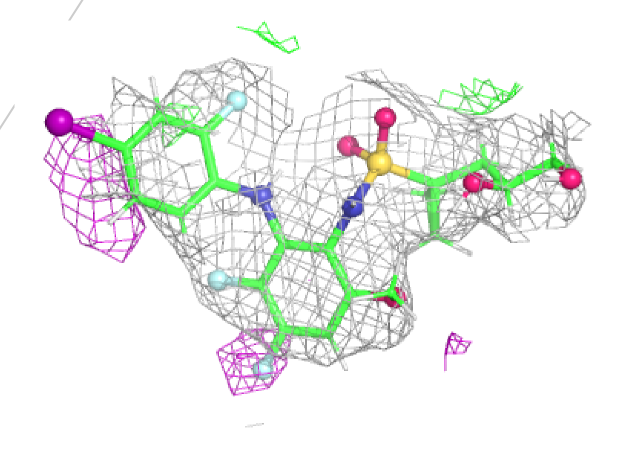

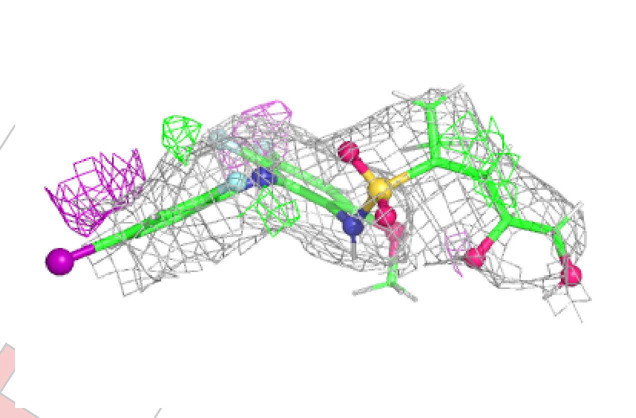

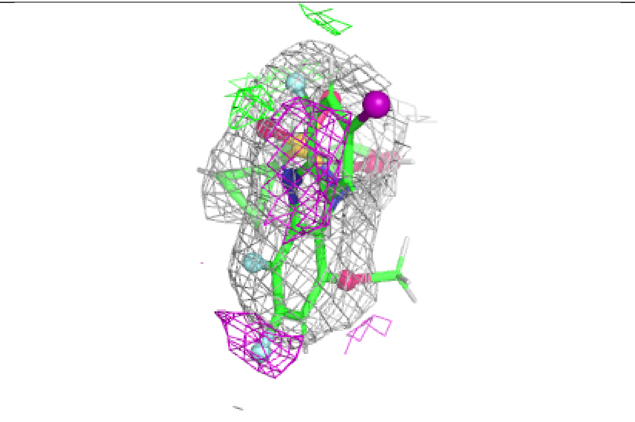

Electron density around VRA B 403:

2mF$_o$-DF$_c$ (at 0.7 rmsd) in gray
mF$_o$-DF$_c$ (at 3 rmsd) in purple (negative)
and green (positive)

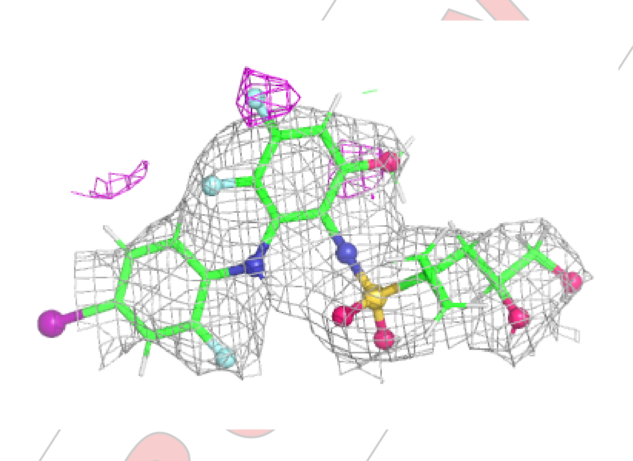

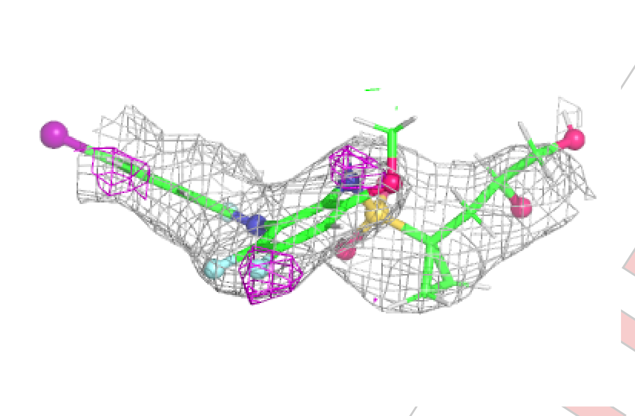

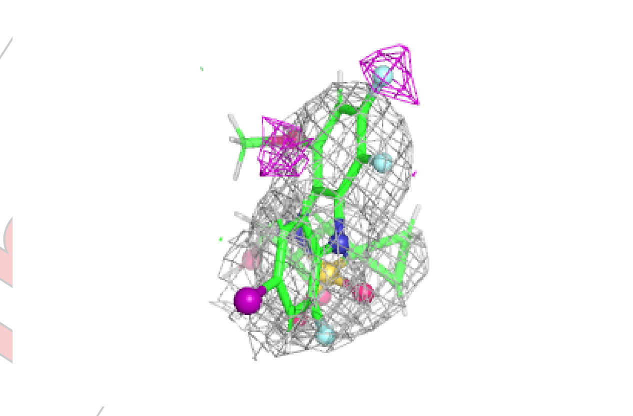

Electron density around VRA D 402:

2mF$_o$-DF$_c$ (at 0.7 rmsd) in gray
mF$_o$-DF$_c$ (at 3 rmsd) in purple (negative)
and green (positive)

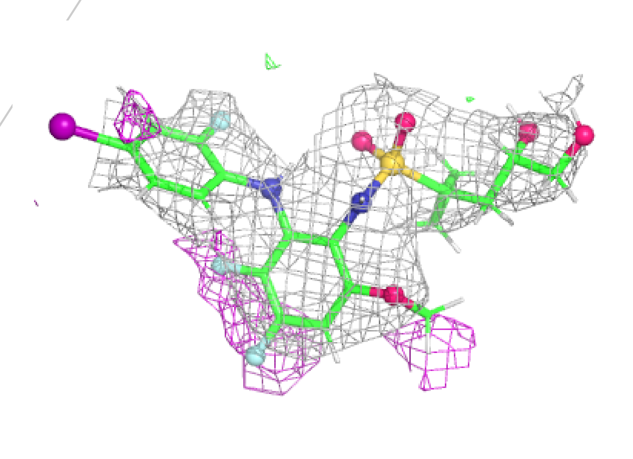

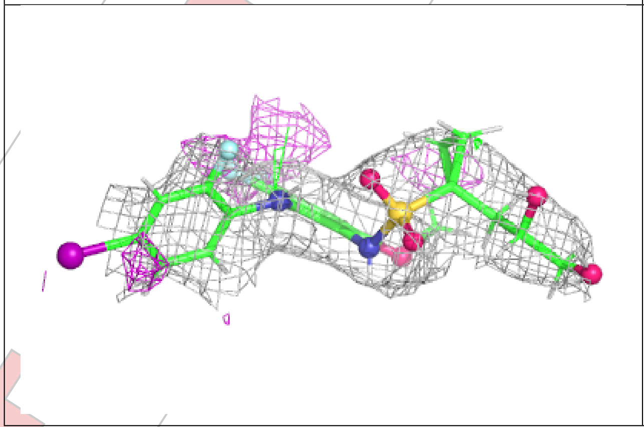

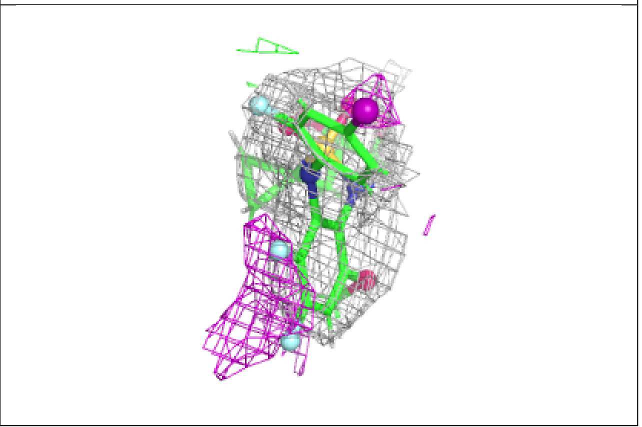

6.5 Other polymers (i)

There are no such residues in this entry.

For Manuscript Review
